# Development and validation of a risk assessment model for predicting the failure of early medical abortions: A clinical prediction model study based on a systematic review and meta-analysis

**DOI:** 10.1371/journal.pone.0315025

**Published:** 2024-12-20

**Authors:** An-Hao Liu, Bin Xu, Xiu-Wen Li, Yue-Wen Yu, Hui-Xin Guan, Xiao-Lu Sun, Yan-Zhen Lin, Li-Li Zhang, Xian-Di Zhuo, Jia Li, Wen-Qun Chen, Wen-Feng Hu, Ming-Zhu Ye, Xiu-Min Huang, Xun Chen

**Affiliations:** 1 Department of Obstetrics and Gynecology, Zhongshan Hospital of Xiamen University, School of Medicine, Xiamen University, Xiamen, China; 2 Department of Basic Medicine, School of Medicine, Xiamen University, Xiamen, China; 3 Department of Ultrasound, Shanghai Changzheng Hospital, Naval Medical University, Shanghai, China; 4 Department of Obstetrics and Gynecology, Jianning General Hospital, Sanming, China; University of Foggia: Universita degli Studi di Foggia, ITALY

## Abstract

**Objective:**

As the first model in predicting the failure of early medical abortion (EMA) was inefficient, this study aims to develop and validate a risk assessment model for predicting the failure of EMAs more accurately in a clinical setting.

**Methods:**

The derivation cohort was obtained from a comprehensive systematic review and meta-analysis. The clinically significant risk factors were identified and combined with their corresponding odds ratios to establish a risk assessment model. The risk factors were assigned scores based on their respective weightings. The model’s performance was evaluated by an external validation cohort obtained from a tertiary hospital. The outcome was defined as the incidence of EMA failure.

**Results:**

A total of 126,420 patients who had undergone medical abortions were included in the systematic review and meta-analysis, and the pooled failure rate was 6.7%. The final risk factors consisted of gestational age, maternal age, parity, previous termination of pregnancy, marital status, type of residence, and differences between gestational age calculated using the last menstrual period and that measured via ultrasound. The risk factors were assigned scores based on their respective weightings, with a maximum score of 19. The clinical prediction model exhibited a good discrimination, as validated by external verification (402 patients) with an area under the curve of 0.857 (95% confidence interval 0.804–0.910). The optimal cutoff value was determined to be 13.5 points, yielding a sensitivity of 83.3% and specificity of 75.4%.

**Conclusion:**

This study effectively establishes a simple risk assessment model including seven routinely available clinical parameters for predicting EMA failure. In preliminary validation, this model demonstrates good performance in terms of predictive efficiency, accuracy, calibration, and clinical benefit. However, more research and validation are warranted for future application.

**Trial registration number:**

CRD42023485388.

## 1. Introduction

Medical abortions emerged as an alternative to surgical abortions for 1st trimester pregnancies with the availability of prostaglandins in the 1970s and anti-progesterones in the 1980s [1–4]. Currently, an estimated 73 million induced abortions are performed annually worldwide [5, 6]. In the United States in 2021, medical abortions accounting for a substantial proportion (56%) of all induced abortions [7]. In England and Wales in 2021, medical abortions constituted approximately 87% of the total number of abortions [8]. Although misoprostol has demonstrated high efficacy in medical abortion regimens containing mifepristone, with success rates exceeding 90% [9–11], the pooled failure rate of medical abortions was determined to be 6.7% (95% CI 5.3%, 8.3%) based on a meta-analysis of the included studies and this study (**Table 1**) [11–19]. Based on the aforementioned data, a roughly estimated 3 million pregnant women experience medical abortion failure annually worldwide. Therefore, revealing and summarizing the risk factors associated with early medical abortion (EMA) failure is of paramount importance in clinical practices.

**Table 1:** Results from meta-analysis to estimate the pooled failure rate of early medical abortions.

Study	No. of medical abortions	No. of failed abortions	Failure rate (%)	95% CI (%)
Ashok 2002 [14]	4131	94	2.3	(1.9, 2.8)
Bartley 2000 [15]	2839	102	3.6	(3.0, 4.3)
Chien 2009 [11]	879	82	9.3	(7.6, 11.4)
Gluck 2023 [16]	778	196	25.2	(22.3, 28.4)
Lefebvre 2008 [17]	1850	54	2.9	(2.2, 3.8)
Meaidi 2019 [12]	86437	5320	6.2	(6.0, 6.3)
Niinimaki 2004 [18]	316	29	9.2	(6.5, 12.9)
Niinimaki 2011 [13]	27030	1447	5.4	(5.1, 5.6)
Reeves 2015 [19]	2160	75	3.5	(2.8, 4.3)
This study	402	48	11.9	(9.1, 15.5)
Pooled analysis*	126822	7447	6.7	(5.3, 8.3)

*Using a random-effects model.

At the molecular level, the expression of insulin-like growth factor 1 protein (IGF-1) and vascular endothelial growth factor protein (VEGF) in chorionic villi, the expression of PGF2α receptor splice variant 2 (FP-V2) in human decidua, and the G2014G genotype in the estrogen receptor 1 gene has been reported to be associated with EMA failure [20–22]. Two primary factors (endometrial thickness and serum β-hCG level) have been identified as predictors of EMA failure after patients undergo medical abortions [23–26]. Although peripheral blood α1-acid glycoprotein has been linked to incomplete EMAs, its clinical validation and application still require further investigation [27].

Furthermore, it is noteworthy that a multitude of factors have been identified as being associated with the risk of EMA failure before patients undergoing medical abortions [11–19, 28–36]. These factors can be categorized into six main groups: different drugs [28–31], different drug dosages [30–32], different administration methods (routes and time intervals) for drugs [30, 31, 33], different settings (home or hospital) [34, 35], different periods [13, 36], and other epidemiological and clinical risk factors [11–19]. According to the specified clinical guidelines from different countries or regions, clinicians are typically unable to modify the former five categories for specific patients and cannot employ them to assess the risk of EMA failure in clinical settings [37–41]. However, risk factors of the 6th category generally exhibit variations for different patients and can be easily applied in clinical practice.

In 2011, Heikinheimo et al. identified five epidemiological and clinical risk factors associated with medical abortion failure (surgical evacuation performed) comprehensively [13]. In 2019, Meaidi et al. developed and validated a risk assessment model for predicting early medical abortion failure (surgical intervention performed) based on epidemiological and clinical data; however, the model was deemed inefficient as indicated by an area under the curve (AUC) of 0.63 [12]. Therefore, the objective of this study is to develop and validate a more accurate risk assessment model for clinically predicting EMA failure, building upon the foundation established by Meaidi and other researchers.

## 2. Methods

### 2.1 Source of data

The derivation cohort was established based on a comprehensive systematic review and meta-analysis of 9 high-quality cohort studies, including 2 prospective and 7 retrospective cohort studies (**Table 2**) [11–19]. This systematic review was registered in the International Prospective Register of Systematic Reviews (PROSPERO), with registration number: CRD42024505032. The electronic databases, including Medline, Embase, Scopus, Web of Science, and Cochrane Library, were systematically searched from their inception to January 19th, 2024. During the search process, specific terms such as "surgical or failed or incomplete or unsuccessful or failure", "medical abortion" and "surgical abortion" were employed (**S1 Appendix**).

**Table 2:** Selected risk factors associated with the failure of medical abortions.

Study	Selected risk factors	Definition of the success of medical abortion	Definition of the failure of medical abortion	Design
Ashok 2002 [14]	PT	Complete uterine evacuation without the need for SGI	ICA, missed abortion, CTP, or the need for SGI	PCS
Bartley 2000 [15]	PA, PT	Complete abortion with no need for surgical evacuation of the uterus	OGP, ICA, or the need for surgical evacuation of the uterus	RCS
Chien 2009 [11]	PA	An empty uterus observed by ultrasonography at follow-up and no SGI	SGI performed after a medical regimen for reasons: vaginal bleeding, lower abdominal pain, septic abortion, ICA, or OGP	RCS
Gluck 2023 [16]	LU	Medical treatment without the requirement of additional surgical treatment	Medical treatment with the requirement of additional surgical treatment	RCS
Lefebvre 2008 [17]	PA	Complete abortion not requiring any additional treatment	CTP, the residual products require aspiration, repeat administration of misoprostol, or need for a curettage	RCS
Meaidi 2019 [12]	GA, MA, VD, CS, MR, PM, PS	A medical abortion without being surgically intervened during the follow-up	A medical abortion being surgically intervened during the follow-up	RCS
Niinimaki 2004 [18]	PA	Without need for curettage for any reason after medical abortion	The need for curettage for any reason after medical abortion	RCS
Niinimaki 2011 [13]	TR, MS, GA	Not receiving surgical evacuation for any reason after medical abortion	Receiving surgical evacuation for any reason after medical abortion	RCS
Reeves 2015 [19]	PA, PT	Not undergoing uterine evacuation for any reason after medical abortion	Undergoing uterine evacuation for any reason after medical abortion	PCS
This study	GA, MA, PA, PT, TR, MS, LU	Complete expulsion without requiring surgical intervention	Requirement for surgical evacuation to complete the abortion due to any reason	RCS

PT, previous termination of pregnancy. PA, parity. VD, only vaginal deliveries and spontaneous delivery of placenta. CS, ≥1 caesarean section. MR, ≥1 manual removal of placenta. GA, gestational age. MA, maternal age. PM, previous medical abortions. PS, previous surgical abortions. MS, marital status. TR, type of residence. LU, differences between gestational age calculated using the last menstrual period and gestational age calculated via ultrasound. SGI, surgical intervention. ICA, incomplete abortion. OGP, Ongoing pregnancy. CTP, continuing pregnancy. RCS, retrospective cohort study. PCS, prospective cohort study.

The validation cohort consisted of EMA patients from Zhongshan Hospital of Xiamen University, whose hospital admission dates were between January 1st, 2019 and May 13rd, 2024. Access to the relevant data was granted for research purposes starting from May 13rd, 2024. The authors ensured that no personally identifiable information of individual participants was accessible during or after data collection. Patient anonymity was maintained throughout this study.

Additionally, we declared that the method section was written according to TRIPOD development and validation writing standard [42].

### 2.2 Study participants

**Fig 1:**
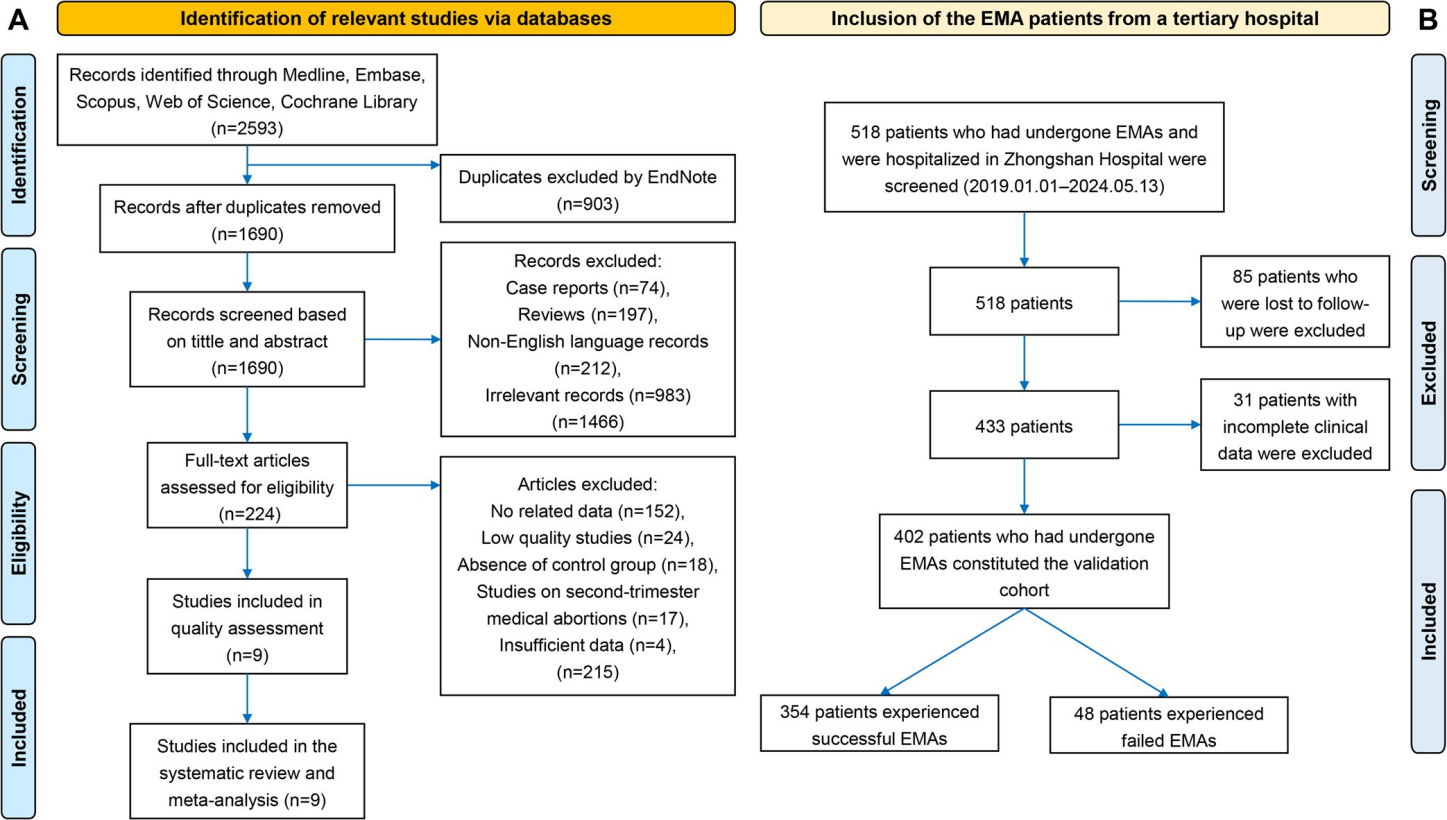
Two flow charts of this study. (A) PRISMA flow chart of the literature selection process. (B) Process for the selection of patients in the validation cohort. PRISMA, preferred reporting items for systematic reviews and meta-analyses. EMA, early medical abortion.

A total of 126,420 patients who had undergone medical abortions were included in our derivation cohort, and they were from Europe, Asia and North America. The range of time of the included studies was from 1994 to 2020. The flowchart of screening studies is presented in **Fig 1A**. The original information of the literature selection process is presented in **S2 Appendix**. The inclusion criteria, exclusion criteria, data extraction, and quality assessment are detailed in **S3 Appendix** [43]. All data extracted from the primary research sources are presented in **S4 Appendix**. The certainty assessment of meta-analysis is presented in **Table 3**.

**Table 3:** GRADE certainty rating of meta-analysis.

Risk factors	Study design	Initial certainty	Reasons for certainty up or down	Final certainty
Parity	OS×5	Low	A (+), B (+)	High*
Previous termination of pregnancy	OS×3	Low	A (+), B (+)	High*

*GRADE rating showing high or moderate certainty. OS, observational study. A, upgraded one level due to large magnitude of effect. B, upgraded one level due to all plausible confounding would reduce a demonstrated effect or suggest a spurious effect when results show no effect. C, downgraded one level due to imprecision of results: wide confidence intervals. D, downgraded one level due to serious inconsistency: heterogeneity in interventions (I^2^> 50%). GRADE: High, we are very confident that the true effect lies close to that of the estimate of the effect. Moderate, we are moderately confident in the effect estimate; the true effect is likely to be close to the estimate of the effect, but there is a possibility that it is substantially different. Low, our confidence in the effect estimate is limited; the true effect may be substantially different from the estimate of the effect. Very low, we have very little confidence in the effect estimate; the true effect is likely to be substantially different from the estimate of effect. (+), yes. (-), no.

The study participants of validation cohort were aged between 16 and 43 years, who received oral administration of mifepristone (150 mg) upon hospitalization, and were followed by oral administration of misoprostol (600 mcg) after a time interval of 72 hours according to the medical abortion guidelines in China [37]. Women were discharged home following medical abortions, which was confirmed through visualization of the products of conception or by ultrasound scanning if necessary. All participants were scheduled to attend a follow-up visit, which included sonographic examination, approximately 6–8 and 13–15 days or longer after the administration of mifepristone.

### 2.3 Study outcomes

In derivation cohort, the success and failure of medical abortions had been defined clearly among included studies (**Table 2**) [44–47].

The outcomes of EMAs in validation cohort were classified as follows: (1) Success: Complete abortion without the need for surgical intervention; (2) Failure: Requirement for surgical evacuation to complete the abortion for any reason.

### 2.4 Clinical predictors

**Fig 2:**
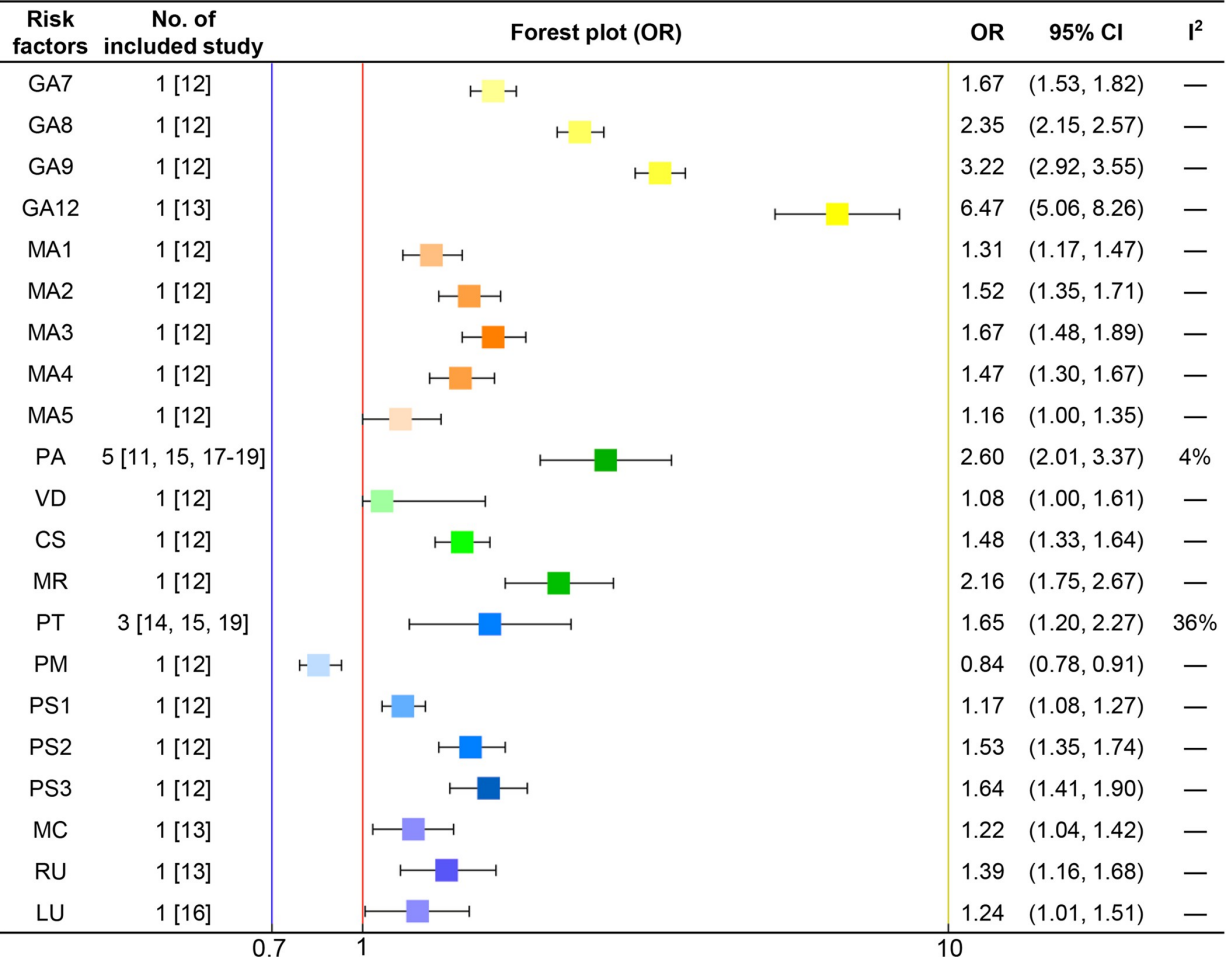
Forest plot of risk factors associated with the failure of early medical abortions. GA7, gestational age of 42–48 days. GA8, gestational age of 49–55 days. GA9, gestational age of 56–62 days. GA12, gestational age of 9–12 weeks. MA1, maternal age of 20–24 years. MA2, maternal age of 25–29 years. MA3, maternal age of 30–34 years. MA4, maternal age of 35–39 years. MA5, maternal age of 40–49 years. PA, parity. VD, only vaginal deliveries and spontaneous delivery of placenta. CS, ≥1 caesarean section. MR, ≥1 manual removal of placenta. PT, previous termination of pregnancy. PM, previous medical abortions. PS1, ≥1 previous surgical abortion: ≥56 days of gestation. PS2, ≥1 previous surgical abortion: <56 days of gestation. PS3, ≥2 previous surgical abortion: both <56 and ≥56 days of gestation. MC, married or cohabiting. RU, rural areas. LU, differences between gestational age calculated using the last menstrual period and gestational age calculated via ultrasound.

**Fig 3:**
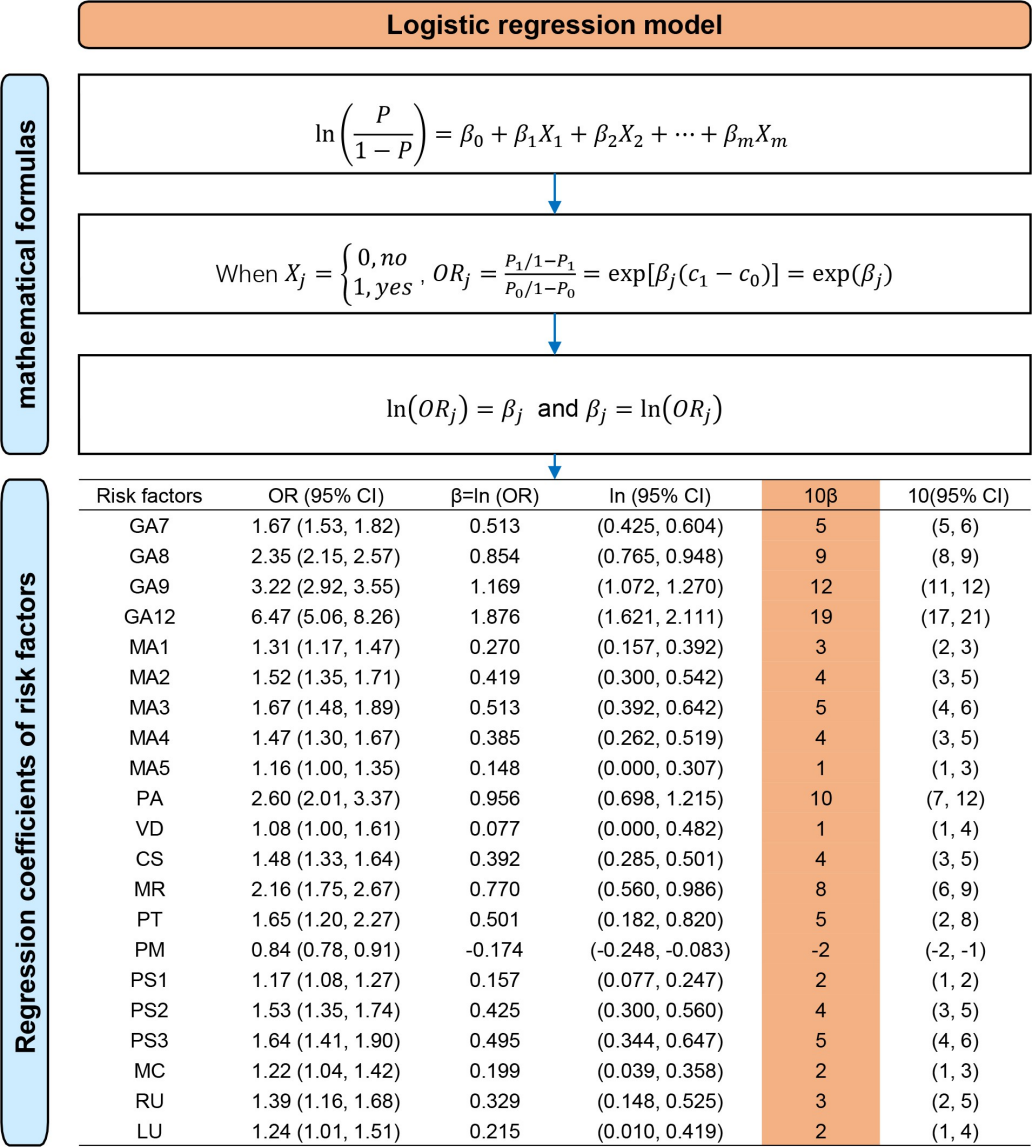
Regression coefficients of risk factors calculated in logistic regression model. P, probability of occurrence of an event (the failure of early medical abortion). X, independent variables (risk factors). β, regression coefficients of risk factors. OR, odds ratio. GA7, gestational age of 42–48 days. GA8, gestational age of 49–55 days. GA9, gestational age of 56–62 days. GA12, gestational age of 9–12 weeks. MA2, maternal age of 25–29 years. MA3, maternal age of 30–34 years. MA4, maternal age of 35–39 years. MA5, maternal age of 40–49 years. PA, parity. VD, only vaginal deliveries and spontaneous delivery of placenta. CS, ≥1 caesarean section. MR, ≥1 manual removal of placenta. PT, previous termination of pregnancy. PM, previous medical abortions. PS1, ≥1 previous surgical abortion: ≥56 days of gestation. PS2, ≥1 previous surgical abortion: <56 days of gestation. PS3, ≥2 previous surgical abortion: both <56 and ≥56 days of gestation. MC, married or cohabiting. RU, rural areas. LU, differences between gestational age calculated using the last menstrual period and gestational age calculated via ultrasound.

According to the systematic review, all clinical risk factors associated with EMA failure were identified and incorporated into the risk assessment model. We conducted a meta-analysis to combine odds ratios (ORs) and 95% confidence intervals (CIs) of risk factors, or alternatively, we directly extracted ORs and 95% CIs from a high-quality study with a large sample size. Subsequently, we calculated the corresponding β-coefficients (**Fig 2**). Related mathematical principles are presented in **Fig 3** [48]. The scores of risk factors were determined by multiplying the β-coefficients by 10 and rounding them to the nearest whole number [49].All risk factors in the risk assessment model were categorized and assigned scores to establish a risk scoring system based on the systematic review and meta-analysis [50]. The scores of each risk factor were summarized to calculate the total score [51]. The higher the cumulative score, the greater the risk of EMA failure.

### 2.5 Sample size

As the derivation cohort was established based on a systematic review, the study participants were included according to the inclusion and exclusion criteria in development cohort.

In validation cohort, based on the anticipated outcome proportion (6.7%) and the desired margin of error (≤0.05), the required sample size was calculated as n = 96.1 [52].


$$n={\left(\frac{1.96}{\delta }\right)}^{2}\hat{\emptyset }(1-\hat{\emptyset })={\left(\frac{1.96}{0.05}\right)}^{2}0.067(1-0.067)=96.1$$

In order to better test the model, we decided to set the sample size to be four times of the required sample size (96.1⨯4 = 384.4).

### 2.6 Missing data

Patients who were lost to follow-up were contacted via telephone and queried regarding their medical condition. Patients with incomplete clinical information were additionally contacted via telephone and requested to provide further details. Relevant patients who were still lost to follow-up or had incomplete clinical data were excluded from the study following our efforts in establishing telephone contact.

### 2.7 Statistical analysis methods

The data obtained from the derivation cohort were utilized for the development of our clinical prediction model. The OR along with its corresponding 95% CI for each risk factor was extracted. The heterogeneity was assessed using the Q-test and I^2^ statistics. Due to the existence of heterogeneity among included studies in term of population, study design and sampling, the random-effects model was utilized. The statistical analysis of the data was conducted using Review Manager 5.4. A level of P < 0.05 was adopted, unless otherwise specified, to determine statistical significance.

The data obtained from the validation cohort were used for the evaluation of our risk assessment model. A risk score system was employed to compute the total score of clinical variables for each patient within the cohort. The total score was utilized to construct a receiver operating characteristic (ROC) curve. Then, the sensitivity, specificity, optimal cutoff point, and AUC were computed. The AUC represents the predictive performance, with a range of values between 0.5 and 1.0. The greater the value, the higher the level of prediction accuracy. Based on the optimal cutoff point, patients were stratified into low-risk and high-risk groups. The model performance was assessed using a calibration plot and OE ratio [53]. Additionally, the decision curve analysis was used to measure risk threshold and net benefit of the risk prediction model [53]. Moreover, a series of subgroup analyses were conducted within the validation cohort, in order to assess the model performance across different clinical risk parameters. The statistical analysis was conducted using SPSS 22.0, GraphPad Prism 9.5 and R version 4.4.1.

### 2.8 Ethics statement

This study was completed in accordance with the ethical principles originating from the Declaration of Helsinki and was approved by the Medical Ethics Committee of Zhongshan Hospital of Xiamen University (No. XMZSYYKY-2024-042).

## 3. Results

### 3.1 Description of the cohorts

#### 3.1.1 Derivation cohort

A total of 126,420 patients who underwent medical abortions were included in the derivation cohort, with the majority having gestational ages of less than 14 weeks and maternal ages ranging between 15–49 years old. These patients had been administered mifepristone and/or prostaglandin analogs for the purpose of medical termination of pregnancy. The majority of patients in the cohort (at least 97%) had a gestational age of less than 12 weeks, while their ages mainly ranged from 15 to 49 years old. The included cohort studies all possessed complete baseline data (**Table 4**). They were evaluated using the Newcastle-Ottawa Scale (**Table 5**) [54]. During the follow-up time following medical abortion, approximately 5.9% (7,399 patients) experienced medical abortion failure. The main baseline characteristics of the derivation cohort are presented in **Table 4**. According to the Newcastle-Ottawa scale, nine included studies were all deemed to be of high quality (**Table 5**). Seven categories of risk factors were gathered, including gestational age (GA), maternal age (MA), parity (PA) and its type, previous termination of pregnancy (PT) and its type, marital status (MS), type of residence (TR), and differences between gestational age calculated using the last menstrual period and that measured via ultrasound (LU). The details of the included studies and corresponding cohorts are provided in **Tables 1–5**.

**Table 4:** Main baseline characteristics of the studies included in this analysis.

Study	Region	Period	No. of medical abortions	No. of failed abortions (%)	Procedure of medical abortions (dose and route)	Gestational age	Maternal age (mean±SD or range)
Ashok 2002 [14]	Scotland	1994–2001	4131	94 (2.3)	Mife (200mg orally) + Miso (0.8/1.2mg vaginally)	≤9 weeks	26±6 years
Bartley 2000 [15]	Scotland	1995–1999	2839	102 (3.6)	Mife (200mg orally) + Geme (0.5mg vaginally)	<9 weeks	26±6 years
Chien 2009 [11]	Taiwan	2002–2007	879	82 (9.3)	Mife (600mg orally) + Miso (0.6mg orally)	≤8 weeks	32±6 years
Gluck 2023 [16]	Israel	2015–2020	778	196 (25.1)	Miso (0.8/1.6mg vaginally)	<11 weeks	33±6 years
Lefebvre 2008 [17]	France	2001–2005	1850	54 (2.9)	Mife (600mg orally) + Miso (0.4/0.8mg orally)	≤7 weeks	17–47 years (100%)
Meaidi 2019 [12]	Denmark	2005–2015	86437	5320 (6.2)	Mife (200mg orally) + Miso (0.8mg vaginally)	≤9 weeks	20–39 years (80%)
Niinimaki 2004 [18]	Finland	~2004	316	29 (9.2)	Mife (200mg orally) + Miso (0.8mg vaginally)	≤9 weeks	≤39 years (96%)
Niinimaki 2011 [13]	Finland	2000–2006	27030	1447 (5.4)	Mife (-) or Mife + Miso (-) or Mife + other PGs (-)	≤12 weeks (86%)	26 years (range: 13–50)
Reeves 2015 [19]	USA	~2015	2160	75 (3.5)	Mife (200mg orally) + Miso (0.8mg vaginally)	≤9 weeks	≤34 years (90%)
This study	China	2019–2024	402	48 (11.9)	Mife (150mg orally) + Miso (0.6mg orally)	≤11 weeks	27±6 years

Mife, mifepristone. Miso, misoprostol. Geme, gemeprost. PGs, prostaglandin analogs.

**Table 5:** Quality assessment of the included studies based on the Newcastle-Ottawa scale.

Study/Assessment	Item 1	Item 2	Item 3	Item 4	Item 5	Item 6	Item 7	Item 8	Total scores
RCS/PCS	A*	B	C	D	E	F	G	H	NOS scores for cohort studies
Ashok 2002 PCS	1	1	1	0	1	1	1	1	**7** ^**†**^
Bartley 2000 RCS	1	1	1	0	1	1	1	1	**7** ^**†**^
Chien 2009 RCS	1	1	1	0	2	1	1	1	**8** ^**†**^
Gluck 2023 RCS	1	1	1	0	2	1	1	1	**8** ^**†**^
Lefebvre 2008 RCS	1	1	1	0	1	1	1	1	**7** ^**†**^
Meaidi 2019 RCS	1	1	1	0	2	1	1	1	**8** ^**†**^
Niinimaki 2004 RCS	1	1	1	0	2	1	1	1	**8** ^**†**^
Niinimaki 2011 RCS	1	1	1	0	2	1	1	1	**8** ^**†**^
Reeves 2015 PCS	1	1	1	0	2	1	1	1	**8** ^**†**^

*Items of quality assessment methods. A, representativeness of the exposed cohort. B, selection of the non-exposed cohort. C, ascertainment of exposure. D, demonstration that outcome of interest was not present at start of study. E, comparability of cohorts on the basis of the design or analysis. F, assessment of outcome. G, was follow-up long enough for outcomes to occur. H, adequacy of follow up of cohorts. NOS, the Newcastle-Ottawa scale. RCS, retrospective cohort study. PCS, prospective cohort study. 1 star, was awarded when the respective information was available. 0 star, was awarded when the respective information was unavailable. †Studies receiving 6 stars or more were considered to be of high quality in NOS.

#### 3.1.2 Validation cohort

A total of 518 patients who had undergone EMAs and were admitted to Zhongshan Hospital were included in our study; however, 85 patients lost to follow-up and 31 patients with incomplete clinical data were excluded. Ultimately, 402 patients were included in the validation cohort (**Fig 1B**). Among the 402 patients who had undergone EMAs, 48 cases (11.9%) experienced early medical abortion failure and 354 cases (88.1%) experienced early medical abortion success. The mean maternal age with the standard deviation (SD) was 27.7±19.1 years, while the median gestational age was 6 0/7 weeks (interquartile range [IQR] = 5 2/7 to 6 2/7 weeks). Among the patients, 30% had exclusively undergo vaginal deliveries with spontaneous placental delivery, and 14% had experienced one or more caesarean sections. About 29% had undergone one or more previous surgical abortions without exact information about gestational age, and 11% had only experienced medical abortions. Additionally, 41% of the patients were married and 32% were from rural areas. The detailed information of the validation cohort patients is provided in **S5 Appendix**.

### 3.2 Model derivation

**Fig 4:**
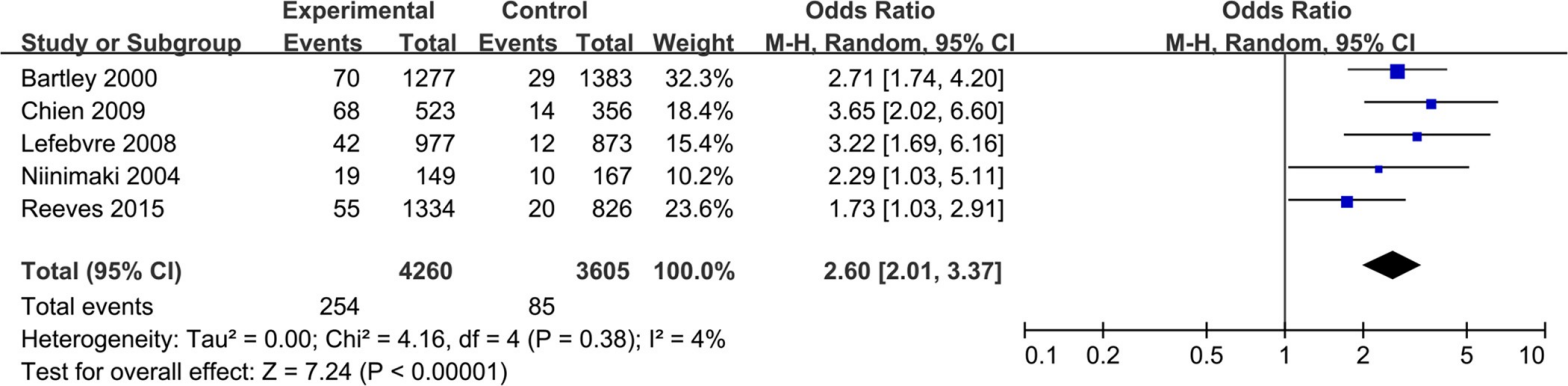
Forest plot meta-analysis of EMA failure risk of patients with parity ≥ 1 versus those with parity = 0.

**Fig 5:**
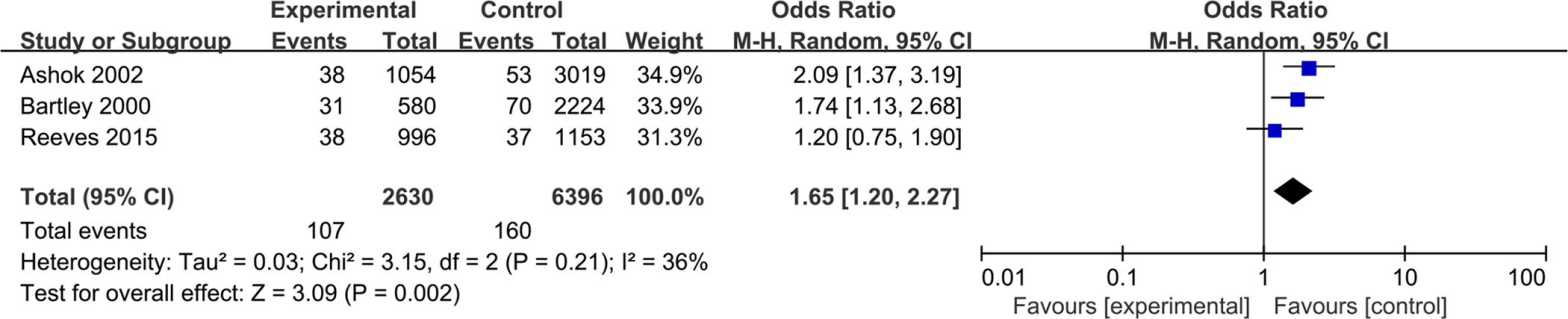
Forest plot meta-analysis of EMA failure risk of patients with previous termination of pregnancy ≥ 1 versus those with previous termination of pregnancy = 0.

The 7 categories of clinical risk factors identified in the systematic review and meta-analysis were included in the risk assessment model. All related 22 risk factors in our model were as follows: gestational age of 42–48 days (OR = 1.67, 95%CI 1.53–1.82), gestational age of 49–55 days (OR = 2.35, 95%CI 2.15–2.57), gestational age of 56–62 days (OR = 3.22, 95%CI 2.92–3.55), gestational age of 9–12 weeks (OR = 6.47, 95%CI 5.06–8.26), maternal age of 20–24 years (OR = 1.31, 95%CI 1.17–1.47), maternal age of 25–29 years (OR = 1.52, 95%CI 1.35–1.71), maternal age of 30–34 years (OR = 1.67, 95%CI 1.48–1.89), maternal age of 35–39 years (OR = 1.47, 95%CI 1.30–1.67), maternal age of 40–49 years (OR = 1.16, 95%CI 1.00–1.35), parity (PA, OR = 2.60, 95%CI 2.01–3.37), only vaginal deliveries and spontaneous delivery of placenta (the placenta was delivered spontaneously without the need of manual removal at the third stage of labor) (VD, OR = 1.08, 95%CI 1.00–1.61), ≥1 caesarean section (CS, OR = 1.48, 95%CI 1.33–1.64), ≥1 manual removal of placenta (MR, OR = 2.16, 95%CI 1.75–2.67), previous termination of pregnancy (PT, OR = 1.65, 95%CI 1.20–2.27), previous medical abortions (PM, OR = 0.84, 95%CI 0.78–0.91), ≥1 previous surgical abortion: ≥56 days of gestation (PS1, OR = 1.17, 95%CI 1.08–1.27), ≥1 previous surgical abortion: <56 days of gestation (PS2, OR = 1.53, 95%CI 1.35–1.74), ≥2 previous surgical abortion: both <56 and ≥56 days of gestation (PS3, OR = 1.64, 95%CI 1.41–1.90), married or cohabiting (MC, OR = 1.22, 95%CI 1.04–1.42), rural areas (RU, OR = 1.39, 95%CI 1.16–1.68), and differences between gestational age calculated using the last menstrual period and that measured via ultrasound (LU, OR = 1.24, 95%CI 1.01–1.51). The forest plot of risk factors is shown in **Fig 2**, and the meta-analysis results are presented in **Figs 4 and 5**. The ORs (95% CI), β-coefficients, and risk scores of the included risk factors are presented in **Table 6**. The mathematical formula of the risk prediction model is as follows:

$${When\;X}_{j}=\left\{ \begin{array}{c} 0,no\\ 1,yes \end{array}\right.$$

$$\ln\left(\frac{P_{EMAfailrue}}{1-P_{EMAfailrue}}\right)=0.513{GA}_{7}+0.854{GA}_{8}+1.169{GA}_{9}+1.876{GA}_{12}+0.270{MA}_{1}+0.419{MA}_{2}+0.513{MA}_{3}+0.385{MA}_{4}+0.148{MA}_{5}+0.956PA+0.077VD+0.392CS+0.770MR+0.501PT-0.174PM+0.157{PS}_{1}+0.425{PS}_{2}+0.495{PS}_{3}+0.199MC+0.329RU+0.215LU+\beta_{0}$$

**Table 6:** ORs, β-coefficients, and scores of clinical risk factors included in the risk assessment model for predicting the failure of EMAs.

Risk factors for EMA	Pooled ORs	95% CIs	β-coefficients	Scores
GA7 (+/-)	1.67	1.53–1.82	0.513	5
GA8 (+/-)	2.35	2.15–2.57	0.854	9
GA9 (+/-)	3.22	2.92–3.55	1.169	12
GA12 (+/-)	6.47	5.06–8.26	1.867	19
MA1 (+/-)	1.31	1.17–1.47	0.270	3
MA2 (+/-)	1.52	1.35–1.71	0.419	4
MA3 (+/-)	1.67	1.48–1.89	0.513	5
MA4 (+/-)	1.47	1.30–1.67	0.385	4
MA5 (+/-)	1.16	1.00–1.35	0.148	1
PA (+/-)	2.60	2.01–3.37	0.956	10
VD (+/-)	1.08	1.00–1.61	0.077	1
CS (+/-)	1.48	1.33–1.64	0.392	4
MR (+/-)	2.16	1.75–2.67	0.770	8
PT (+/-)	1.65	1.20–2.27	0.501	5
PM (+/-)	0.84	0.78–0.91	-0.174	-2
PS1 (+/-)	1.17	1.08–1.27	0.157	2
PS2 (+/-)	1.53	1.35–1.74	0.425	4
PS3 (+/-)	1.64	1.41–1.90	0.495	5
MC (+/-)	1.22	1.04–1.42	0.199	2
RU (+/-)	1.39	1.16–1.68	0.329	3
LU (≥1/<1 week)	1.24	1.01–1.51	0.215	2

EMA, early medical abortion. β, regression coefficients of risk factors. OR, odds ratio. GA7, gestational age of 42–48 days. GA8, gestational age of 49–55 days. GA9, gestational age of 56–62 days. GA12, gestational age of 9–12 weeks. MA1, maternal age of 20–24 years. MA2, maternal age of 25–29 years. MA3, maternal age of 30–34 years. MA4, maternal age of 35–39 years. MA5, maternal age of 40–49 years. PA, parity. VD, only vaginal deliveries and spontaneous delivery of placenta. CS, ≥1 caesarean section. MR, ≥1 manual removal of placenta. PT, previous termination of pregnancy. PM, previous medical abortions. PS1, ≥1 previous surgical abortion: ≥56 days of gestation. PS2, ≥1 previous surgical abortion: <56 days of gestation. PS3, ≥2 previous surgical abortion: both <56 and ≥56 days of gestation. MC, married or cohabiting. RU, rural areas. LU, differences between gestational age calculated using the last menstrual period and gestational age calculated via ultrasound. (+), yes. (-), no.

### 3.3 Model validation

**Fig 6:**
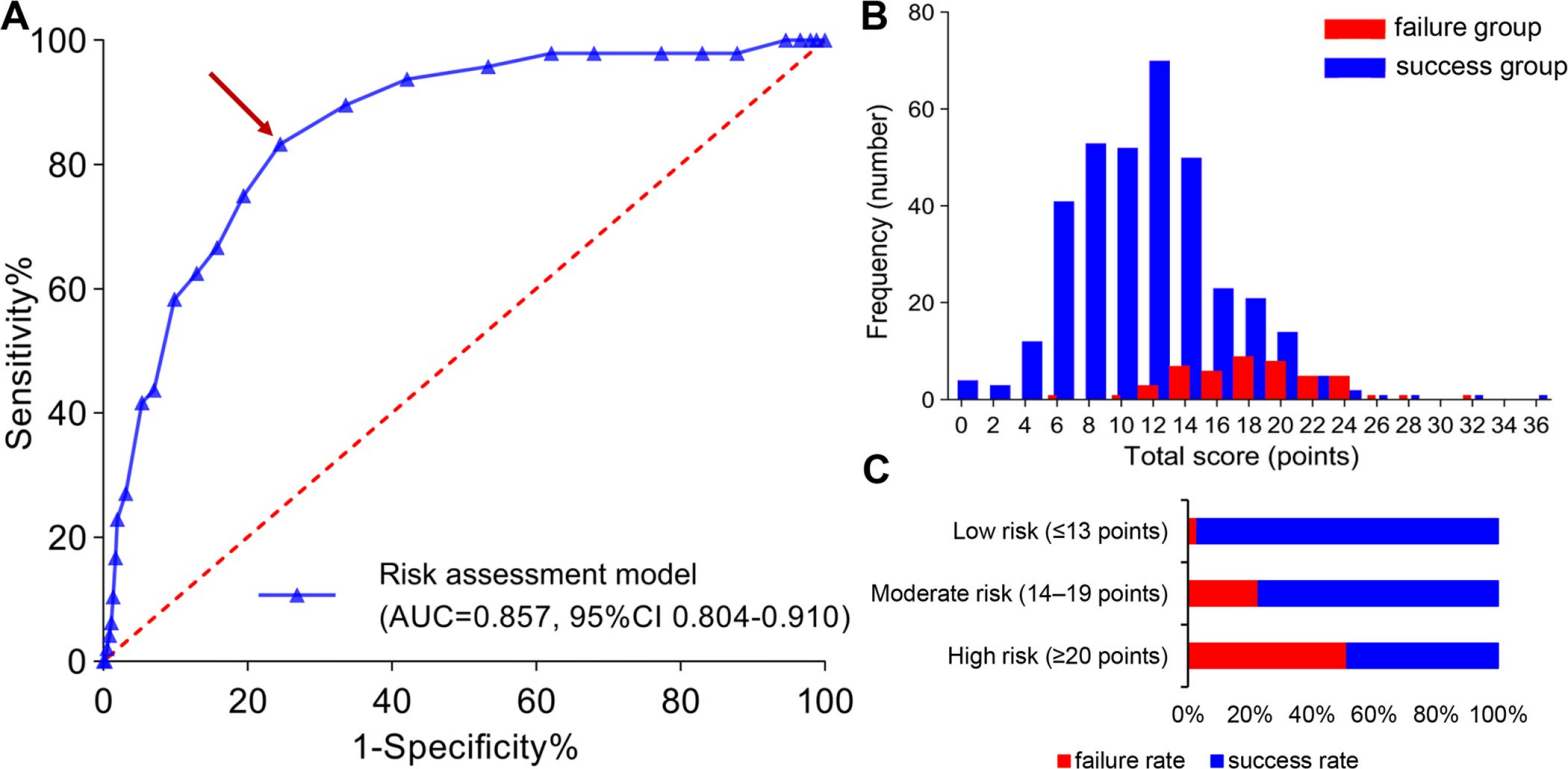
Three kinds of analyses exhibited the performance of the risk prediction model. (A) The receiver operator characteristic (ROC) curve analysis for predicting the failure of EMA. The area under the curve (AUC) was 0.857 (95% CI 0.804–0.910, P < 0.0001). (B) Frequency distribution histogram of the success and failure groups of EMA. (C) Failure and success rate of EMA in three risk groups stratified by risk score in the validation cohort. Low risk: ≤13 points. Moderate risk: 14–19 points. High risk: ≥20 points. Brown arrow: the best cutoff value (13.5) with the highest Youden index (0.588).

The AUC value of the validation cohort was 0.857 (95% CI 0.804–0.910), and the ROC curve is showed in **Fig 6A**. The optimal cutoff risk score was determined to be 13.5 points, based on the maximum Youden index (0.588), yielding a sensitivity of 83.3% and specificity of 75.4%. The sensitivity, specificity, and Youden indexes of different critical risk scores are provided in **Table 7**. The frequency distribution histogram of the success and failure groups is presented in **Fig 6B**. According to the optimal cutoff value and the frequency distribution, a total of 402 patients who had undergone EMAs were stratified into three groups: low-risk (n = 275), moderate-risk (n = 88) and high-risk (n = 39). The corresponding risk scores were ≤13, 14–19 and ≥20, respectively. Compared with the low-risk group (3%), the failure rates of EMAs in the moderate-risk group and high-risk group were 23% and 51% respectively (**Fig 6C**).

**Table 7:** Sensitivity, specificity, and Youden indexes of different cutoff values.

Cutoff value (points)	Sensitivity	Specificity	Youden index
-1	1.000	0.000	0.000
1	1.000	0.011	0.011
2.5	1.000	0.019	0.019
3.5	1.000	0.033	0.033
4.5	1.000	0.053	0.053
5.5	0.979	0.121	0.100
6.5	0.979	0.169	0.148
7.5	0.979	0.225	0.204
8.5	0.979	0.319	0.298
9.5	0.979	0.378	0.357
10.5	0.958	0.466	0.424
11.5	0.938	0.579	0.517
12.5	0.896	0.663	0.559
**13.5** *	**0.833**	**0.754**	**0.588**
14.5	0.750	0.805	0.555
15.5	0.667	0.841	0.508
16.5	0.625	0.870	0.495
17.5	0.583	0.901	0.484
18.5	0.438	0.929	0.367
19.5	0.417	0.946	0.363
20.5	0.271	0.968	0.239
21.5	0.229	0.980	0.209
22.5	0.167	0.983	0.149
23.5	0.104	0.985	0.089
24.5	0.063	0.988	0.051
25.5	0.042	0.991	0.033
26.5	0.021	0.994	0.015
29	0.000	0.997	-0.003
33	0.000	1.000	0.000
36	1.000	0.000	0.000

*The optimal cutoff risk score (13.5) based on the maximum Youden index.

**Fig 7:**
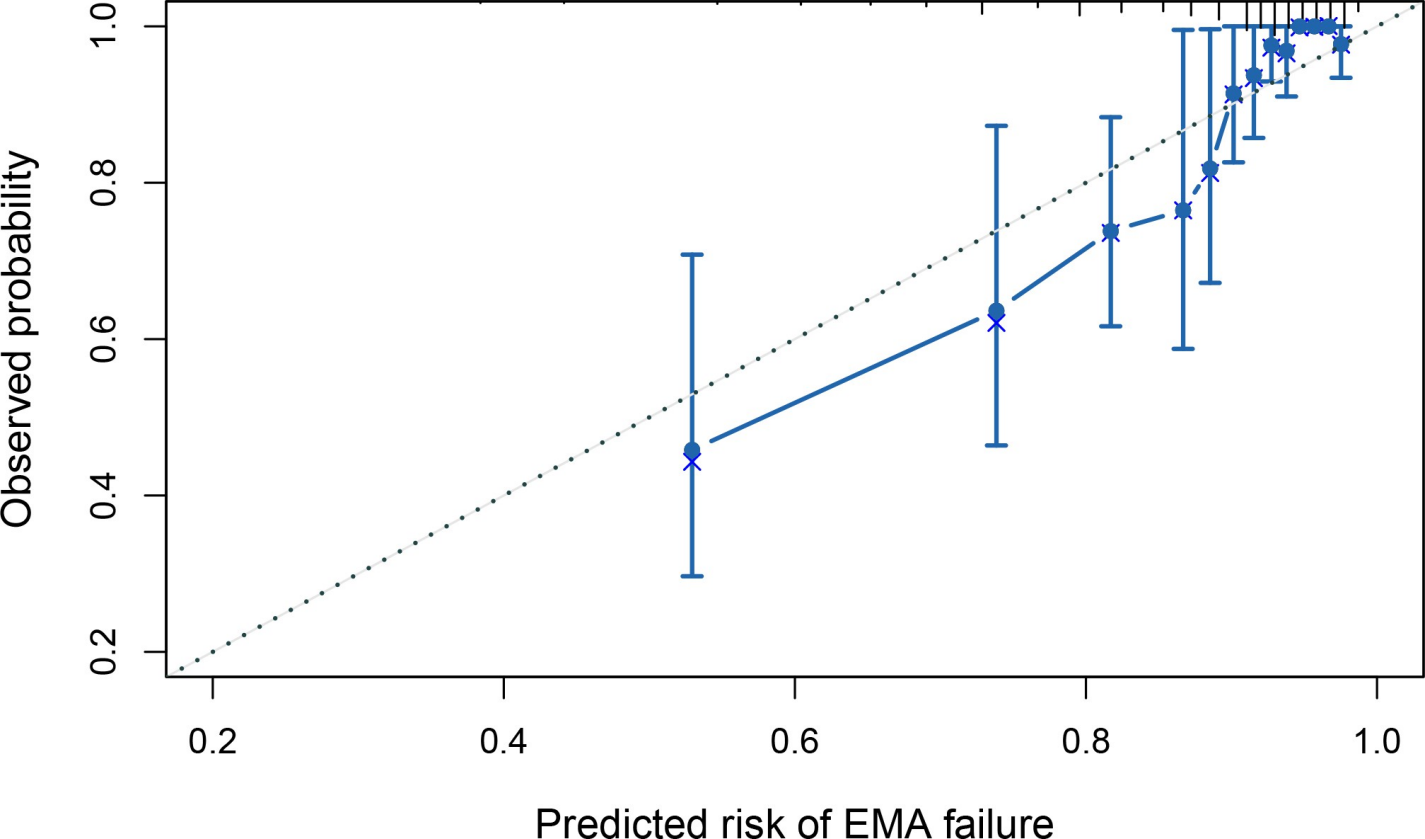
Calibration plot of the prediction model in the validation cohort. The long-dotted line represents a perfect prediction, and the solid line represent the predictive performance of the model. The closer the solid line fits the long-dotted line, the better the predictive accuracy of the model.

**Fig 8:**
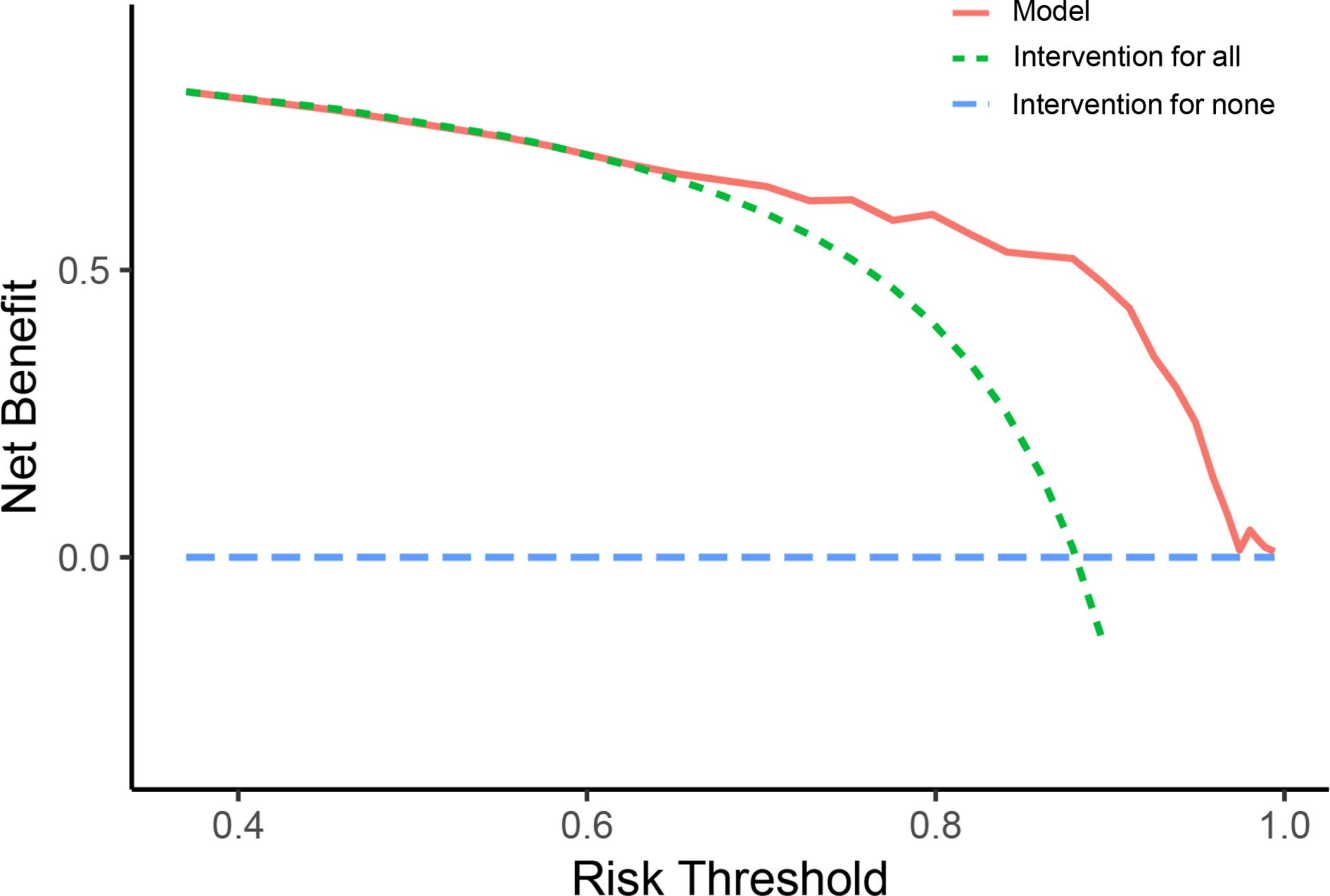
Decision curve analysis of the risk prediction model in the validation cohort. The red line represents the risk prediction model. The green line represents the intervention for all patients. The blue line represents the intervention for none patients. When the risk threshold of EMA failure is higher than 0.6, this model yields a net benefit.

In addition, the calibration plot of the prediction model in the validation cohort revealed the goodness of fit of the predicted risk and observed probability of EMA failure (**Fig 7**). Based on the risk threshold of high-risk group (≥20 points), the OE ratio was calculated as 1.231 (95%CI 0.904–1.558), which indicated that this model might exhibit a tendency towards underprediction. Moreover, the decision curve analysis showed that this model yielded a net benefit when the risk threshold of EMA failure was higher than 0.6 (**Fig 8**).

**Fig 9:**
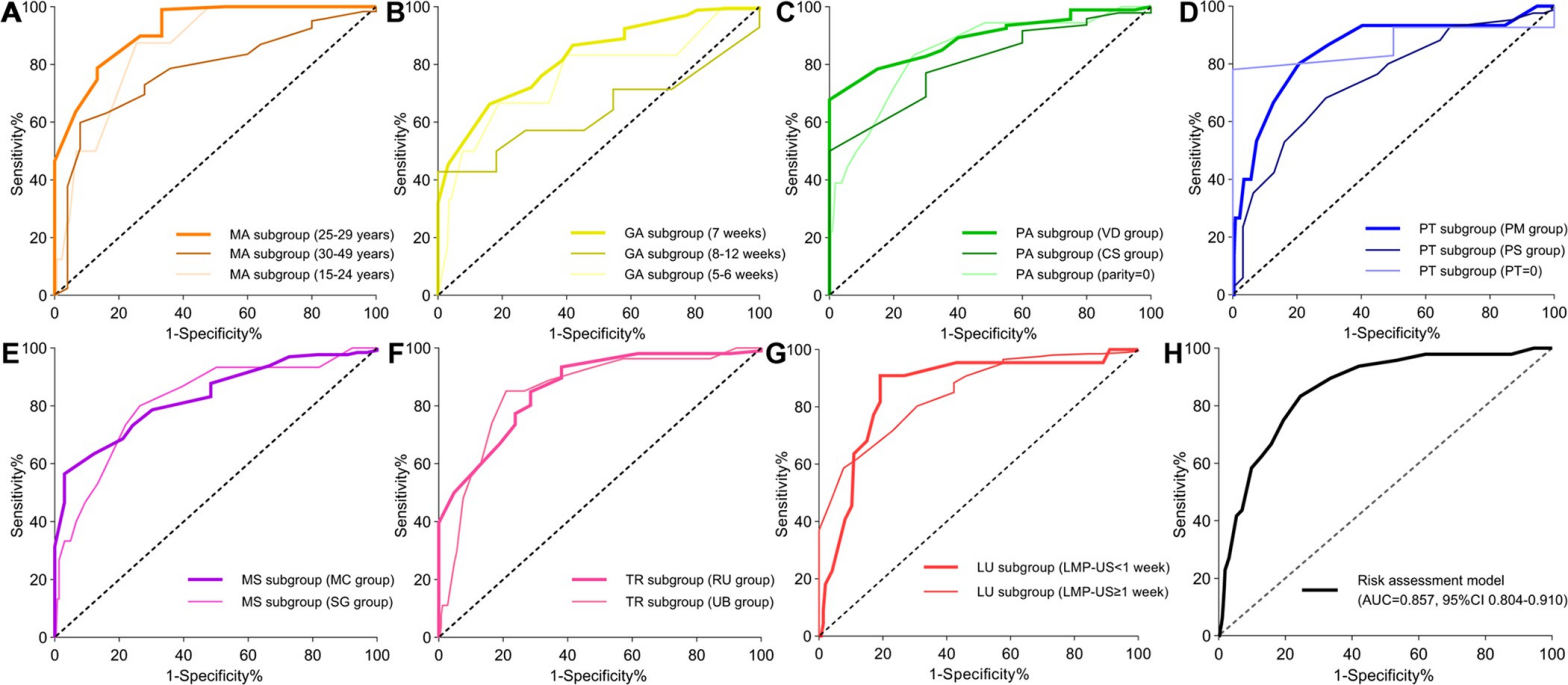
Subgroup analyses of 7 clinical risk parameters within the validation cohort. (A) The receiver operator characteristic (ROC) curve analysis of three MA subgroups. (B) The ROC curve analysis of three GA subgroups. (C) The ROC curve analysis of three PA subgroups. (D) The ROC curve analysis of three PT subgroups. (E) The ROC curve analysis of two MS subgroups. (F) The ROC curve analysis of two TR subgroups. (G) The ROC curve analysis of two LU subgroups. (H) The ROC curve analysis of the risk assessment model.

In subgroup analysis, the ROC curve analyses of 7 clinical risk parameters were conducted within the validation cohort (**Fig 9**). All the AUC values of three MA subgroups were more than 0.750. The AUC values of one GA subgroup (8–12 weeks) was 0.640 (95%CI 0.417–0.862), and the other two subgroups exhibited that the AUC values were > 0.750. Three PA subgroups, two MS subgroups, two TR subgroups and two LU subgroups all exhibited that the AUC values were more than 0.800. All the AUC values of three PT subgroups were more than 0.740 (**Table 8**).

**Table 8:** Subgroup analyses of 7 clinical risk parameters within the validation cohort.

Clinical risk parameters	Subgroups	AUC values	95% CI
MA	15–24 years	0.857	(0.757, 0.956)
	25–29 years*	0.921	(0.849, 0.993)
	30–49 years	0.774	(0.680, 0.869)
GA	5–6 weeks	0.759	(0.529, 0.989)
	7 weeks*	0.824	(0.756, 0.893)
	8–12 weeks	0.640	(0.417, 0.862)
PA	Parity = 0 group	0.842	(0.742, 0.942)
	VD group*	0.889	(0.829, 0.950)
	CS group	0.805	(0.682, 0.929)
PT	PT = 0 group	0.850	(0.735, 0.966)
	PM group*	0.866	(0.737, 0.994)
	PS group	0.774	(0.645, 0.842)
MS	MC*	0.831	(0.764, 0.897)
	SG	0.817	(0.701, 0.934)
TR	RU	0.861	(0.780, 0.943)
	UB	0.848	(0.773, 0.923)
LU	LMP-US<1 week*	0.858	(0.773, 0.943)
	LMP-US≥1 week	0.848	(0.779, 0.916)

*This group exhibited a highest AUC value in subgroup analysis. GA, gestational age. MA, maternal age. LU, differences between gestational age calculated using the last menstrual period (LMP) and gestational age calculated via ultrasound (US). PA, parity. VD, only vaginal deliveries and spontaneous delivery of placenta. CS, ≥1 caesarean section. PT, previous termination of pregnancy. PM, previous medical abortions. PS, ≥1 previous surgical abortion. MS, marital status. SG, single. MC, married or cohabiting. TR, type of residence. UB, urban areas. RU, rural areas. AUC, an area under the receiver operating characteristic curve.

### 3.4 Risk prediction scale

According to the risk prediction model, a simple risk scale was also developed (points): GA (days: 28–41 = 0, 42–48 = 5, 49–55 = 9, 56–62 = 12, weeks: 9–12 = 19), MA (years: 15–19 = 0, 20–24 = 3, 25–29 = 4, 30–34 = 5, 35–39 = 4, 40–49 = 1), PA (times: 0 = 0, ≥1 = 10; types: VD = 1, CS = 4, MR = 8), PT (times: 0 = 0, ≥1 = 5; types: PM = -2, PS1 = 2, PS2 = 4, PS3 = 5), MS (types: single = 0, MC = 2), TP (types: urban = 0, RU = 3), LU (weeks: <1 = 0, ≥1 = 2). This model was recommended to be used for patients aged 15–49 years in order to predict the risk of EMA failure before undergoing EMAs (**Table 9**). The mathematical formula of the risk prediction scale is as follows:

Totalscore=GA+MA+LU+PA+PT+MS+TR

**Table 9:** Risk assessment scale for clinically predicting the failure of EMAs.

Risk factors for EMA	Points	Risk factors for EMA	Points
**GA**		**PA (≥1)**	**10**
GA5–6 (+)	0	VD (+)	1
GA7 (+)	5	CS (+)	4
GA8 (+)	9	MR (+)	8
GA9 (+)	12	**PT (0)**	**0**
GA12 (+)	19	**PT (≥1)**	**5**
**MA**		PM (+)	-2
MA0 (+)	0	PS1 (+)	2
MA1 (+)	3	PS2 (+)	4
MA2 (+)	4	PS3 (+)	5
MA3 (+)	5	**MS**	
MA4 (+)	4	SG (+)	0
MA5 (+)	1	MC (+)	2
**LU**		**TR**	
LU (LMP–US <1 week)	0	UB (+)	0
LU (LMP–US ≥1 week)	2	RU (+)	3
**PA (0)**	**0**	**Total score** = GA+MA+LU+PA+PT+MS+TR =	

EMA, early medical abortion. GA, gestational age. GA5–6, gestational age of 28–41 days. GA7, gestational age of 42–48 days. GA8, gestational age of 49–55 days. GA9, gestational age of 56–62 days. GA12, gestational age of 9–12 weeks. MA, maternal age. MA0, maternal age of 15–19 years. MA1, maternal age of 20–24 years. MA2, maternal age of 25–29 years. MA3, maternal age of 30–34 years. MA4, maternal age of 35–39 years. MA5, maternal age of 40–49 years. LU, differences between gestational age calculated using the last menstrual period (LMP) and gestational age calculated via ultrasound (US). PA, parity. VD, only vaginal deliveries and spontaneous delivery of placenta. CS, ≥1 caesarean section. MR, ≥1 manual removal of placenta. PT, previous termination of pregnancy. PM, previous medical abortions. PS1, ≥1 previous surgical abortion:≥56 days of gestation. PS2, ≥1 previous surgical abortion: <56 days of gestation. PS3, ≥2 previous surgical abortion: both <56 and ≥56 days of gestation. MS, marital status. SG, single. MC, married or cohabiting. TR, type of residence. UB, urban areas. RU, rural areas. (+), yes. (-), no.

## 4. Discussion

To date, an effective risk assessment model for clinical prediction of EMA failure has not been established by previous studies [55]. To the best of our knowledge, this is the second risk prediction model with a relatively good performance [12].

The first prediction model was developed and validated by Meaidi et al. in 2019, based on a high-quality cohort of more than 80 thousand EMA patients, which included 6 clinical parameters (GA, MA, PA, PM, PS and calendar time) with an AUC of 0.63 [12]. Based on the solid foundation established by Meaidi, Heikinheimo and other researchers, we developed and validated a more comprehensive model with 3 additional clinical parameters (MS, TP, and LU), which exhibited a good discrimination. Although the risk of surgical intervention following medical abortions decreased over time from 2008 to 2012 [36], our risk prediction model cannot incorporate this factor due to its current inapplicability. In addition, our risk assessment model was refined into a simplified risk score scale based on rigorous mathematical principles, facilitating its integration into clinical practices for the convenience of clinicians [56].

Moreover, as the study conducted by Meaidi et al. did not provide an assessment of the overall risk ratios for PA or PT [12], we performed a meta-analysis to extract and combine the OR values from high-quality cohort studies [11, 14, 15, 17–19]. Given the frequent lack of precise information about PA (number and type) and PT (type and gestational age) due to the forgetfulness or other factors, our pooled findings on PA and PT can offer doctors a more convenient reference for real-world clinical applications.

According to a high-quality cohort study comprising over 20,000 patients undergoing medical abortions, Heikinheimo et al. (2011) identified single status and residing in rural areas as risk factors associated with medical abortion failure [13]. Although the gestational age of patients was less than 20 weeks, a majority of patients (at least 86%) had undergone EMAs (≤12 weeks) [13]. Consequently, our risk prediction model assimilated these risk factors and transformed them into parameters [50].

In 2023, Gluck et al. reported that the differences between gestational age calculated using the last menstrual period (LMP) and that measured via ultrasound (US) were correlated with EMA failure [16]. Despite the inherent limitations of the cohort study design, a multivariate regression analysis revealed a direct association between differences in LMP-US and the risk of EMA failure [16]. Therefore, this risk factor was also incorporated into our predictive model.

In addition, there were no unified clear definitions of success or failure of medical abortions (**Table 2**) [44–47]. Winikoff et al. proposed that the definition of failures in medical abortion should encompass any surgical intervention performed for any reason [44]. Fiala et al. concluded that success may be defined as the termination of pregnancy without requiring additional intervention, while failure may encompass ongoing viable pregnancy, the necessity for additional treatment, or expectant management [45]. Zwerling et al. defined a successful abortion as the complete expulsion of the pregnancy through the intended medical method without any additional intervention, and they also defined a failed abortion as the counterpart to their defined successful abortion, for the purpose of their guideline [46]. Kruse et al. considered that the occurrence of medical abortion failure could be defined as the need for a surgical evacuation to complete the abortion for any reason [47]. According to the aforementioned definitions, we defined a successful abortion as complete expulsion without requiring surgical intervention, while the EMA failure was defined as the requirement for surgical evacuation to complete the abortion due to any reason.

Some important confounders were also considered to have a significant impact on the occurrence of EMA failure, such as socioeconomic factors, geographic region-specific practices, and access to healthcare [11–19, 28–33, 37–41]. Several studies reported that the occurrence rates of EMA failure among developed European countries (2–9%) were lower than those in developing countries/regions (9–11%) [11–19]. This exhibited that socioeconomic factors may significantly affect the failure of EMAs. According to the specified clinical guidelines, medical abortion drugs, dosages, and administration methods were different in different countries [37–41]. Recently, some studies elucidated that the incidence of EMA failure was apparently affected by these specific medical practices [28–33]. In 2011, Heikinheimo et al. identified rural areas as a risk factor associated with medical abortion failure, which may reveal the impact of access to healthcare [13].

There are three key strengths in our study. First, this is the second risk assessment model for clinically predicting the risk of EMA failure, with a good discrimination (AUC = 0.857). Second, this study conducts a more comprehensive analysis of the available clinical risk factors, surpassing previous studies in terms of summarization. Third, the risk score scale (**Table 9**) can be readily employed by clinicians to assess the risk of EMA failure in clinical settings.

Conversely, there are several limitations. Firstly, the presence of heterogeneity among the included studies may impact the reliability of the risk prediction model to a certain extent. Secondly, the validated cohort in this study had a relatively smaller sample size compared to the derivation cohort, which may limit the generalizability of the results across different populations and healthcare settings. Thirdly, the participants of our validation cohort were from a single-center tertiary hospital. Despite these limitations, the risk assessment model derived and validated from this study still potentially provides an effective clinical prediction method for evaluating the risk of EMA failure prior to undergoing medical abortions.

It is recommended that EMAs could be considered for pregnant women classified as low-risk (≤13 points) or moderate-risk (14–19 points). Furthermore, pregnant women in the moderate-risk group should receive comprehensive information regarding the potential occurrence of EMA failure, and undergo close monitoring. Although medical abortion is not recommended for patients in the high-risk group (≥20 points) due to the high failure rate (51%), it is essential to respect and value the patient’s choice of abortion method. Undoubtedly, this prediction model still necessitates rigorous testing, meticulous refinement, and further research prior to its practical implementation.

## 5. Conclusion

Based on a systematic review and meta-analysis, this study effectively establishes a simple risk assessment model including seven routinely available clinical parameters for predicting EMA failure. In preliminary validation, this model demonstrates good performance in terms of predictive efficiency, accuracy, calibration, and clinical benefit. However, more research and validation are warranted for future application.

## Supporting information

S1 Checklist PRISMA 2020 checklist. (DOCX)

S2 Checklist Human participants research checklist. (PDF)

S3 Checklist TRIPOD checklist: Prediction model development and validation. (DOCX)

S1 Appendix Search strategy of systematic review. (DOCX)

S2 Appendix Original information of the literature selection process. (DOCX)

S3 Appendix The inclusion criteria, exclusion criteria, data extraction, and quality assessment of systematic review. (DOCX)

S4 Appendix All data extracted from the primary research sources for the systematic review and meta-analysis. (DOCX)

S5 Appendix Original data of the validation cohort patients. (DOCX)

S1 Fig PRISMA flow chart of the literature selection process. PRISMA, preferred reporting items for systematic reviews and meta-analyses.
(PPTX)

## Abbreviations

EMA: early medical abortion
OR: odds ratio
IGF-1: insulin-like growth factor 1 protein
VEGF: vascular endothelial growth factor protein
FP-V2 PGF2α: receptor splice variant 2
AUC: an area under the receiver operating characteristic curve
ROC: receiver operating characteristic curve
PROSPERO: the International Prospective Register of Systematic Reviews
CI: confidence interval
GA: gestational age
MA: maternal age
PA: parity
PT: previous termination of pregnancy
MS: marital status
TR: type of residence
LU: differences between gestational age calculated using the last menstrual period and that measured via ultrasound
VD: only vaginal deliveries and spontaneous delivery of placenta
CS ≥1: caesarean section
MR ≥1: manual removal of placenta
PT: previous termination of pregnancy
PM: previous medical abortions
PS1 ≥1: previous surgical abortion: ≥56 days of gestation
PS2 ≥1: previous surgical abortion: <56 days of gestation
PS3 ≥2: previous surgical abortion: both <56 and ≥56 days of gestation
MC: married or cohabiting
RU: rural areas
SD: standard deviation
IQR: interquartile range
LMP: gestational age calculated using the last menstrual period
US: gestational age measured via ultrasound
