# Supplementary material for: Development and validation of a risk assessment model for predicting the failure of early medical abortions: A clinical prediction model study based on a systematic review and meta-analysis
Source: PLoS One. 2024 Dec 20;19(12):e0315025. doi: 10.1371/journal.pone.0315025 (PMC11661585; doi:10.1371/journal.pone.0315025)
Supplement: S1 Appendix — (DOCX) [file pone.0315025.s004.docx]

**S1 Appendix. Search strategy of systematic review.**

**Search strategy**

**1. PubMed** (from the inception to January 19th, 2024)

#1 surgical OR failed OR incomplete OR unsuccessful OR failure [Title/Abstract]

#2 medical abortion [Title/Abstract]

#3 surgical abortion [Title/Abstract]

#4 (#1 AND #2 NOT #3) **= 412**

**2. Embase** (from the inception to January 19th, 2024)

#1 surgical OR failed OR incomplete OR unsuccessful OR failure [Title/Abstract]

#2 medical abortion [Title/Abstract]

#3 surgical abortion [Title/Abstract]

#4 (#1 AND #2 NOT #3) **= 541**

**3. Scopus** (from the inception to January 19th, 2024)

#1 surgical OR failed OR incomplete OR unsuccessful OR failure [Title/Abstract]

#2 medical abortion [Title/Abstract]

#3 surgical abortion [Title/Abstract]

#4 (#1 AND #2 NOT #3) **= 887**

**4. Web of Science** (from the inception to January 19th, 2024)

#1 surgical OR failed OR incomplete OR unsuccessful OR failure [Title/Abstract]

#2 medical abortion [Title/Abstract]

#3 surgical abortion [Title/Abstract]

#4 (#1 AND #2 NOT #3) **= 744**

**5. Cochrane library** (from the inception to January 19th, 2024)

#1 surgical OR failed OR incomplete OR unsuccessful OR failure [Title/Abstract]

#2 medical abortion [Title/Abstract]

#3 surgical abortion [Title/Abstract]

#4 (#1 AND #2 NOT #3) **= 9**

**Total = 2593**
