# Supplementary material for: Development and validation of a risk assessment model for predicting the failure of early medical abortions: A clinical prediction model study based on a systematic review and meta-analysis
Source: PLoS One. 2024 Dec 20;19(12):e0315025. doi: 10.1371/journal.pone.0315025 (PMC11661585; doi:10.1371/journal.pone.0315025)
Supplement: S2 Appendix — (DOCX) [file pone.0315025.s005.docx]

**S2 Appendix. Original information of the literature selection process.**

**1. Records identified through Medline, Embase, Scopus, Web of Science, Cochrane Library (n=2593)**

**1.1 Records identified through Medline (n=412)**

1. Impact of the Covid-19 Pandemic on the Prevalence of MTP Cases and Their Clinicodemographic Profile in India: A Retrospective Multicentric Study
2. Abortion medical management between 14-16 weeks' amenorrhea after French legislation deadline extension
3. Oncologic and Fertility Outcomes after Simple Trachelectomy in Women with Early Cervical Cancer
4. Medical abortion in the second trimester - an update
5. Failure Rate of Medical Treatment for Miscarriage Correlated with the Difference between Gestational Age According to Last Menstrual Period and Gestational Size Calculated via Ultrasound
6. Abortion in the First Trimester. Guideline of the DGGG (S2k-Level, AWMF Registry No. 015-094, December 2022) - Part 1 with Recommendations on Care Structures, Information and Advice on Decision-Making, Measures Before Abortion and Medical Abortion
7. A comparison of misoprostol with and without methylergometrine and oxytocin in outpatient medical abortion: a phase III randomized controlled trial
8. Using telemedicine to improve early medical abortion at home (UTAH): a randomised controlled trial to compare telemedicine with in-person consultation for early medical abortion
9. Misoprostol-Induced Fever and Unnecessary Antibiotic Prescribing: A Retrospective Study
10. "I will never wish this pain to even my worst enemy": Lived experiences of pain associated with manual vacuum aspiration during post-abortion care in Kenya
11. The contraceptive choice at the time of a surgical and pharmacological abortion: a possible and effective option thanks to a dedicate counselling
12. Telephone follow-up after early medical abortion using Australia's first low sensitivity urine pregnancy test
13. Cognitive Bias in the Management of a Critically Ill 29-Year-Old Patient
14. Parsing the Q-Markers of Baoyin Jian to Treat Abnormal Uterine Bleeding by High-Throughput Chinmedomics Strategy
15. Diagnosis of medulloblastoma in pregnant women: To continue with the pregnancy or not? A case report and literature review
16. Abortion services during the COVID-19 pandemic: a systematic review
17. Structural barriers or patient preference? A mixed methods appraisal of medical abortion use in England and Wales
18. Comparison of mifepristone plus misoprostol with misoprostol alone for first trimester medical abortion: A systematic review and meta-analysis
19. Development and pilot testing of the 2019 Canadian Abortion Provider Survey
20. Improving safe post-abortion care practices: A study on interventions implemented by Ipas Pakistan
21. Pharmacy dispensing of abortion pills in Ghana: experiences of pharmacy workers and users
22. Effectiveness and acceptability of home use of misoprostol for medical abortion up to 10 weeks of pregnancy
23. A prospective, comparative study of clinical outcomes following clinic-based versus self-use of medical abortion
24. An examination of loss to follow-up and potential bias in outcome ascertainment in a study of direct-to-patient telemedicine abortion in the United States
25. Accessing abortion in a highly restrictive legal regime: characteristics of women and pregnant people in Malta self-managing their abortion through online telemedicine
26. Acute uterine inversion following an induced abortion
27. Integrative transcriptomics and proteomics analyses to reveal the therapeutic effect and mechanism of Buxue Yimu Pills in medical-induced incomplete abortion rats
28. Management and treatment of brain tumors during pregnancy: an Italian survey
29. Ultrasonographic features of the endometrium following successful medical termination of early pregnancy
30. Profile of Women receiving Second-trimester Safe Abortion Service at Paropakar Maternity and Women's Hospital
31. Myometritis with pelvic septic vein thrombophlebitis secondary to Fusobacterium necrophorum sepsis
32. Trend of serum beta-human chorionic gonadotropin levels after medical abortion in the early first trimester of pregnancy
33. Willing but not able: A survey of New Zealand health practitioners' interests in providing second trimester abortion care and the obstacles they face
34. Toward a Standard Measure of Abortion Service Quality-A Stakeholder First Approach
35. The efficacy of Shenghua Decoction supplementation after early medical abortion: A meta-analysis of randomized controlled trials
36. Validation of a Spanish-language scale for evaluating perceived quality of care of medical abortions before 9 weeks gestation
37. Medical methods for first trimester abortion
38. Abortion policy implementation in Ireland: Lessons from the community model of care
39. The impact of provider restrictions on abortion-related outcomes: a synthesis of legal and health evidence
40. Buxue Yimu Pills improve angiogenesis and blood flow in experimental zebrafish and rat models
41. Analysis of Complications and Management After Self-Administration of Medical Termination of Pregnancy Pills
42. Subsequent placenta accreta after previous mifepristone-induced abortion: A case report
43. [Evaluation of the success of medical abortion by a plasma hCG control threshold]
44. Effectiveness and acceptability of "at home" versus "at hospital" early medical abortion - A lesson from the COVID-19 pandemic: A retrospective cohort study
45. Combined vesicouterine rupture during second-trimester medical abortion for fetal abnormality after prior cesarean delivery: A case report
46. It's a small bit of advice, but actually on the day, made such a difference…: perceptions of quality in abortion care in England and Wales
47. Cervical Twin Heterotopic Pregnancy: Overview of Ectopic Pregnancies and Scanning Detection Algorithm
48. Predictive value of peripheral blood α1-acid glycoprotein in medical abortion outcomes with mifepristone and relativity of concentration
49. Client perspectives on choice of abortion method in England and Wales
50. Accuracy of anemia screening by point-of-care hemoglobin testing in patients seeking abortion
51. Prevalence of Self Induced Abortion by Self-Administration of Abortive Pills among Abortion-related Admissions in a Tertiary Care Centre
52. Clinical analysis of second-trimester pregnancy termination after previous caesarean delivery in 51 patients with placenta previa and placenta accreta spectrum: a retrospective study
53. UTAH: Using Telemedicine to improve early medical Abortion at Home: a protocol for a randomised controlled trial comparing face-to-face with telephone consultations for women seeking early medical abortion
54. Termination of pregnancy services in Irish general practice from January 2019 to June 2019
55. Medical abortion offered in pharmacy versus clinic-based settings
56. 10-year evaluation of the use of medical abortion through telemedicine: a retrospective cohort study
57. Declining risk of surgical intervention following early medical abortion: A time trend analysis
58. PXR mediates mifepristone-induced hepatomegaly in mice
59. Medical management of induced and incomplete first-trimester abortion by non-physicians in low- and middle-income countries: A systematic review and meta-analysis of randomized controlled trials
60. Second trimester medical abortion in a primigravida with lupus nephritis and rapidly progressive renal failure: challenges and outcome
61. Telemedicine medical abortion at home under 12 weeks' gestation: a prospective observational cohort study during the COVID-19 pandemic
62. Two prophylactic pain management regimens for medical abortion ≤63 days' gestation with mifepristone and misoprostol: A multicenter, randomized, placebo-controlled trial
63. Advantages and Disadvantages of Medical Abortion, According to Brazilian Residents in Obstetrics and Gynaecology
64. Chemical Fingerprint Analysis and Ultra-Performance Liquid Chromatography Quadrupole Time-of-Flight Mass Spectrometry-Based Metabolomics Study of the Protective Effect of Buxue Yimu Granule in Medical-Induced Incomplete Abortion Rats
65. [If you were to have another abortion, would you choose the same method? A study on 1032 patients' level of satisfaction]
66. Inequalities in access to and quality of abortion services in Mexico: Can task-sharing be an opportunity to increase legal and safe abortion care?
67. How the coronavirus disease 2019 pandemic is impacting sexual and reproductive health and rights and response: Results from a global survey of providers, researchers, and policy-makers
68. Expulsion at home for early medical abortion: A systematic review with meta-analyses
69. Comparison of the efficacy and safety of two combined misoprostol regimens for second trimester medical abortion
70. Systematic Review of the Effectiveness, Safety, and Acceptability of Mifepristone and Misoprostol for Medical Abortion in Low- and Middle-Income Countries
71. Medical termination for pregnancy in early first trimester (≤ 63 days) using combination of mifepristone and misoprostol or misoprostol alone: a systematic review
72. Cesarean Scar Ectopic Pregnancy: The Lurking Danger in Post Cesarean Failed Medical Abortion
73. Risk of surgical evacuation and risk of major surgery following second-trimester medical abortion in Denmark: A nationwide cohort study
74. 2017-19 governmental decisions to allow home use of misoprostol for early medical abortion in the UK
75. A repeat dose of misoprostol 800 mcg following mifepristone for outpatient medical abortion at 64-70 and 71-77 days of gestation: A retrospective chart review
76. Medical abortion
77. Women's self-reported experiences using misoprostol obtained from drug sellers: a prospective cohort study in Lagos State, Nigeria
78. Women's voices and medical abortions: A review of the literature
79. Surgical or medical abortion of pregnancies between 13(+0) and 23(+6) weeks' gestation? A systematic review and new NICE national guidelines
80. Self-administered versus provider-administered medical abortion
81. Simultaneous compared to interval administration of mifepristone and misoprostol for medical abortion up to 10(+0) weeks' gestation: a systematic review with meta-analyses
82. Understanding of changes in abortion rate following liberalization of abortion law among Brazilian residents in obstetrics and gynecology
83. Modern methods to induce abortion: Safety, efficacy and choice
84. A non-inferiority study of outpatient mifepristone-misoprostol medical abortion at 64-70 days and 71-77 days of gestation
85. Follow-up strategies to confirm the success of medical abortion of pregnancies up to 10 weeks' gestation: a systematic review with meta-analyses
86. General practitioner knowledge and practice in relation to unintended pregnancy in the Grampians region of Victoria, Australia
87. [Conflicting recommendations between the French national authority for health (HAS) and clinical practice guidelines (CNGOF); focus on 200 late medical abortions, conducted outside marketing authorizations]
88. Surgical Management of Incomplete Abortion by Manual Vacuum Aspiration (MVA)
89. Feasibility of a hospital outpatient day procedure for medication abortion at 13-18 weeks gestation: Findings from Nepal()
90. Management of pain associated with up-to-9-weeks medical termination of pregnancy (MToP) using mifepristone-misoprostol regimens: expert consensus based on a systematic literature review
91. First trimester termination of pregnancy
92. Determinants and Outcome of Safe Second Trimester Medical Abortion at Jimma University Medical Center, Southwest Ethiopia
93. Current and potential methods for second trimester abortion
94. A prospective study of mifepristone and unlimited dosing of sublingual misoprostol for termination of second-trimester pregnancy in Uzbekistan and Ukraine
95. Medical abortion with mifepristone and vaginal misoprostol between 64 and 70 days' gestation
96. Outcomes During Early Implementation of Mifepristone-Buccal Misoprostol Abortions up to 63 Days of Gestation in a Canadian Clinical Setting
97. Abortion providers' experiences and views on self-managed medication abortion: an exploratory study
98. Follow-up Rates and Contraceptive Choices after Medical Abortion in Adolescents at Cook County Hospital
99. Telemedicine for medical abortion: a systematic review
100. Early abortion with buccal versus sublingual misoprostol alone: a multicenter, randomized trial
101. Expanding a woman's options to include home use of misoprostol for medical abortion up until 76 days: an observational study of efficacy and safety
102. Outcome of first trimester medical termination of pregnancy: definitions and management
103. Efficacy of Misoprostol Alone for First-Trimester Medical Abortion: A Systematic Review
104. Complications related to induced abortion: a combined retrospective and longitudinal follow-up study
105. Does supportive legislation guarantee access to pregnancy termination and postabortion care services? Findings from a facility census in Central Province, Zambia
106. Acceptability and feasibility of outpatient medical abortion with mifepristone and misoprostol up to 70 days gestation in Singapore
107. Efficacy and Safety of Intravaginal Misoprostol for Mid-trimester Medical Termination of Pregnancy
108. Early medical abortion with self-administered low-dose mifepristone in combination with misoprostol
109. Changes in abortion service provision in Bihar and Jharkhand states, India between 2004 and 2013
110. Abortion education in Canadian family medicine residency programs
111. Providing accessible medical abortion services in a Victorian rural community: A description and audit of service delivery and contraception follow up
112. Protective Effect of Taohong Siwu Decoction on Abnormal Uterine Bleeding Induced by Incomplete Medical Abortion in Rats during Early Pregnancy
113. A direct-to-patient telemedicine abortion service in Australia: Retrospective analysis of the first 18 months
114. Effectiveness and safety of sublingual misoprostol in medical treatment of the 1st trimester miscarriage: experience of off-label use in Korea
115. Risk factors and the choice of long-acting reversible contraception following medical abortion: effect on subsequent induced abortion and unwanted pregnancy
116. Simultaneous Compared With Interval Medical Abortion Regimens Where Home Use Is Restricted
117. Self-assessment of medical abortion outcome using symptoms and home pregnancy testing
118. Comparison of two low-sensitivity urine pregnancy tests for confirming the success of early medical abortion
119. Simplified medical abortion screening: a demonstration project
120. Experiences of women living in Hungary seeking a medical abortion online
121. Provision of medical abortion by midlevel healthcare providers in Kyrgyzstan: testing an intervention to expand safe abortion services to underserved rural and periurban areas
122. Evaluation of effect of letrozole prior to misoprostol in comparison with misoprostol alone in success rate of induced abortion
123. A maternal death from self-induced medical abortion: a call for action
124. Serum β-hCG concentration is a predictive factor for successful early medical abortion with vaginal misoprostol within 24 hours
125. Experience of clandestine use of medical abortion among university students in Chile: a qualitative study
126. Efficacy of medical abortion prior to 6 gestational weeks: a systematic review
127. The role of medical abortion in the implementation of the law on voluntary termination of pregnancy in Uruguay
128. Is self-assessment of medical abortion using a low-sensitivity pregnancy test combined with a checklist and phone text messages feasible in South African primary healthcare settings? A randomized trial
129. Self reported outcomes and adverse events after medical abortion through online telemedicine: population based study in the Republic of Ireland and Northern Ireland
130. [Retrospective assessment of the influence of gestational age on the first trimester medical abortion efficiency]
131. Follow-up after early medical abortion: Comparing clinical assessment with self-assessment in a rural hospital in northern Norway
132. Efficacy and safety of mifepristone-buccal misoprostol for early medical abortion in an Australian clinical setting
133. Sequential use of Foley catheter with misoprostol for second trimester pregnancy termination in women with and without caesarean scars: a prospective cohort study
134. Intrauterine contraception after medical abortion: factors affecting success of early insertion
135. [Abortion law in France]
136. Comparative Study of Mifepristone with Vaginal Misoprostol for First Trimester Termination of Pregnancy at Different Gestational Ages
137. Efficacy of concurrent administration of mifepristone and misoprostol for termination of pregnancy
138. Immediate versus delayed insertion of an etonogestrel releasing implant at medical abortion-a randomized controlled equivalence trial
139. Factors related to completeness of medical abortion with mifepristone and misoprostol
140. The role of medical abortion in the implementation of the law on voluntary termination of pregnancy in Uruguay
141. Self-administered multi-level pregnancy tests in simplified follow-up of medical abortion in Tunisia
142. '…a one stop shop in their own community': Medical abortion and the role of general practice
143. Medical Abortion Provided by Nurse-Midwives or Physicians in a High Resource Setting: A Cost-Effectiveness Analysis
144. Prospective study of home use of mifepristone and misoprostol for medical abortion up to 10weeks of pregnancy in Kazakhstan
145. Uncomplicated abortion with mifepristone and misoprostol in a hemophilia A carrier
146. Effect of Immediate Compared With Delayed Insertion of Etonogestrel Implants on Medical Abortion Efficacy and Repeat Pregnancy: A Randomized Controlled Trial
147. Elevated mRNA expression of PGF2α receptor splice variant 2(FP-V2) in human decidua is associated with incomplete mifepristone-misoprostol-induced early medical abortion by regulation of interleukin-8
148. Effect of Wujia Shenghua capsule on uterine bleeding following medically-induced incomplete abortion in rats during early pregnancy
149. Factors influencing the abortion interval of second trimester pregnancy termination using misoprostol
150. Self-assessment of the outcome of early medical abortion versus clinic follow-up in India: a randomised, controlled, non-inferiority trial
151. Mifepristone With Buccal Misoprostol for Medical Abortion: A Systematic Review
152. Nurse versus physician-provision of early medical abortion in Mexico: a randomized controlled non-inferiority trial
153. Expulsion and continuation rates after postabortion insertion of framed IUDs versus frameless IUDs - review of the literature
154. Medical students' intentions to seek abortion training and to provide abortion services in future practice
155. Early versus late misoprostol administration after mifepristone for medical abortion
156. Association between ultrasonographic parameters of Cesarean scar defect and outcome of early termination of pregnancy
157. A randomized trial of hospital vs home self administration of vaginal misoprostol for medical abortion
158. [Medical abortion provided by telemedicine to women in Latin America: complications and their treatment]
159. Is It Safe to Provide Abortion Pills over the Counter? A Study on Outcome Following Self-Medication with Abortion Pills
160. Pharmacy workers in Nepal can provide the correct information about using mifepristone and misoprostol to women seeking medication to induce abortion
161. The role of auxiliary nurse-midwives and community health volunteers in expanding access to medical abortion in rural Nepal
162. Assessment of completion of early medical abortion using a text questionnaire on mobile phones compared to a self-administered paper questionnaire among women attending four clinics, Cape Town, South Africa
163. The introduction of first trimester medical abortion in Armenia
164. Safety of induced abortions at less than 12 weeks of pregnancy in Japan
165. Administration of depot medroxyprogesterone acetate on the day of mifepristone for medical abortion: a pilot study
166. Clinical follow-up compared with self-assessment of outcome after medical abortion: a multicentre, non-inferiority, randomised, controlled trial
167. Can women determine the success of early medical termination of pregnancy themselves?
168. Change in abortion services after implementation of a restrictive law in Texas
169. Integrating mobile phones into medical abortion provision: intervention development, use, and lessons learned from a randomized controlled trial
170. Efficacy and acceptability of a mifepristone-misoprostol combined regimen for early induced abortion among women in Mexico City
171. Situation analysis of patients attending TU Teaching Hospital after medical abortion with problems and complications
172. Medical abortion with mifepristone and home administration of misoprostol up to 63 days' gestation
173. Replacement of dilation and curettage/evacuation by manual vacuum aspiration and medical abortion, and the introduction of postabortion contraception in Pakistan
174. Achievements of the FIGO Initiative for the Prevention of Unsafe Abortion and its Consequences in South-Southeast Asia
175. Triangular assessment of the etiology of induced abortion in iran: a qualitative study
176. [Clinical study on 39 cases with caesarean scar pregnancy with sonographic mass]
177. Practice bulletin no. 143: medical management of first-trimester abortion
178. Misoprostol Abortion: Ultrasonography versus Beta-hCG Testing for Verification of Effectiveness
179. [Medical abortion for first-trimester termination of the pregnancy]
180. Minimal effective dose of mifepristone for medical abortion
181. [Misoprostol: off-label use in the first trimester of pregnancy (spontaneous abortion, and voluntary medical termination of pregnancy)]
182. Ectopic pregnancy in the era of medical abortion: are we ready for it? Spectrum of sonographic findings and our experience in a tertiary care service hospital of India
183. A Prospective Trial Using Mifepristone and Vaginal Misoprostol in Termination of Pregnancies up to 63 Days of Gestation
184. Medical termination of pregnancy during the second versus the first trimester and its effects on subsequent pregnancy
185. A two-pill sublingual misoprostol outpatient regimen following mifepristone for medical abortion through 70 days' LMP: a prospective comparative open-label trial
186. Provision of medical abortion using telemedicine in Brazil
187. Uterine preservation surgery for placental polyp
188. Prophylactic compared with therapeutic ibuprofen analgesia in first-trimester medical abortion: a randomized controlled trial
189. Comprehensive abortion care: evidence of improvements in hospital-level indicators in Tigray, Ethiopia
190. Interruption of nonviable pregnancies of 24-28 weeks' gestation using medical methods: release date June 2013 SFP guideline #20133
191. [Update in current care guidelines: induced abortion]
192. Misoprostol-induced termination of second-trimester pregnancy in women with a history of cesarean section: a retrospective analysis of 56 cases
193. Medical abortion follow-up with serum human chorionic gonadotropin compared with ultrasonography: a randomized controlled trial
194. Survey of the attitude to, the knowledge and the practice of contraception and medical abortion in women who attended a family planning clinic
195. Sonographic patterns of the endometrium in assessment of medical abortion outcomes
196. Effectiveness of early medical abortion using low-dose mifepristone and buccal misoprostol in women with no defined intrauterine gestational sac
197. Extending outpatient medical abortion services through 70 days of gestational age
198. Unsuccessful prior attempts to terminate pregnancy among women seeking first trimester abortion at registered facilities in Bihar and Jharkhand, India
199. Early medical abortion using low-dose mifepristone followed by buccal misoprostol: a large Australian observational study
200. [Cervical pregnancy: a rare case of reimplantation after abortion. A case report]
201. First-trimester medical abortion with mifepristone 200 mg and misoprostol: a systematic review
202. Introducing medical abortion in Turkey: perspectives of physicians
203. Prospective study of medical abortion in Nepal Medical College Teaching Hospital (NMCTH). A one year experience
204. A pilot study on the use of a 7-day course of letrozole followed by misoprostol for the termination of early pregnancy up to 63 days
205. Comparison of misoprostol-only and combined mifepristone-misoprostol regimens for home-based early medical abortion in Tunisia and Vietnam
206. Failed early medical abortion: beware of the uterine scar!--Case report
207. Clinical, surgical, and histopathologic outcomes following failed medical abortion
208. Clinical diagnosis of completeness of medical abortion by nurses: a reliability study in Mozambique
209. Does congenital heart disease severely jeopardise family life and pregnancies? Obstetrical history of women with congenital heart disease in a single tertiary centre
210. [Effects of taking estrogen and progestogen after medical abortion on reducing vaginal hemorrhage time: a randomized-controlled trial]
211. Effect of letrozole on uterine artery Doppler flow indices prior to first-trimester termination of pregnancy: a randomized controlled trial
212. Pre-emptive effect of ibuprofen versus placebo on pain relief and success rates of medical abortion: a double-blind, randomized, controlled study
213. Women's decision making regarding choice of second trimester termination method for pregnancy complications
214. Efficacy of misoprostol administration 24 hours after mifepristone for termination of early pregnancy
215. Can women accurately assess the outcome of medical abortion based on symptoms alone?
216. Effects of leonurine hydrochloride on medically induced incomplete abortion in early pregnancy rats
217. Efficacy and safety of mifepristone and buccal misoprostol versus buccal misoprostol alone for medical abortion
218. Has mifepristone medical abortion expanded abortion access in New Mexico? A survey of OB-GYN and Family Medicine physicians
219. A randomized comparative study on vaginal administration of acetic acid-moistened versus dry misoprostol for mid-trimester pregnancy termination
220. Adolescent girls undergoing medical abortion have lower risk of haemorrhage, incomplete evacuation or surgical evacuation than women above 18 years old
221. Methotrexate and misoprostol teratogenicity: further expansion of the clinical manifestations
222. Surgical intervention after medical abortion
223. Comparative effectiveness, safety and acceptability of medical abortion at home and in a clinic: a systematic review
224. Comparison of rates of adverse events in adolescent and adult women undergoing medical abortion: population register based study
225. Can midlevel health-care providers administer early medical abortion as safely and effectively as doctors? A randomised controlled equivalence trial in Nepal
226. Simultaneous administration of mifepristone and misoprostol for early termination of pregnancy: a randomized controlled trial
227. Placenta accreta: a cause of failed medical abortion
228. Mifepristone followed by home administration of buccal misoprostol for medical abortion up to 70 days of amenorrhoea in a general practice in Curaçao
229. Medical methods for mid-trimester termination of pregnancy
230. Evaluation of a network of medical abortion providers in two districts of Maharashtra, India
231. A comparative study on sublingual versus oral and vaginal administration of misoprostol for late first and early second trimester abortion
232. Randomized trial of oral versus sublingual misoprostol 24 h after mifepristone for medical abortion
233. Misoprostol dose and route after mifepristone for early medical abortion: a randomised controlled noninferiority trial
234. Medical abortion at 63 to 90 days of gestation
235. Issues in second trimester induced abortion (medical/surgical methods)
236. Home self-administration of vaginal misoprostol for medical abortion at 50-63 days compared with gestation of below 50 days
237. Early pregnancy termination with a simplified mifepristone: Medical abortion outpatient regimen
238. Association of the G2014G genotype in estrogen receptor 1 gene with failure of the mifepristone-induced termination of early pregnancy
239. Is mifepristone 100mg an effective alternative to standard dose for medical abortion
240. Timing and indication for curettage after medical abortion in early pregnant women with prior uterine incision
241. [Medical abortion at home and at hospital: a trial of efficacy and acceptability]
242. Increasing women's choices in medical abortion: a study of misoprostol 400 microg swallowed immediately or held sublingually following 200 mg mifepristone
243. Exploring the costs and economic consequences of unsafe abortion in Mexico City before legalisation
244. Non-physician clinicians can safely provide first trimester medical abortion
245. Endometrial thickness following medical abortion is not predictive of subsequent surgical intervention
246. Medication abortion
247. Frequency and risk factors for repeat abortions after surgical compared with medical termination of pregnancy
248. Effect of previous live birth and prior route of delivery on the outcome of early medical abortion
249. Setting up a medical abortion service for failure
250. Mifepristone plus vaginal misoprostol vs vaginal misoprostol alone for medical abortion in gestation 63 days or less in Nepalese women: a quasi-randomized controlled trial
251. Two mifepristone doses and two intervals of misoprostol administration for termination of early pregnancy: a randomised factorial controlled equivalence trial
252. [Management of pheochromocytoma during pregnancy: about three cases]
253. High-dose misoprostol as an alternative therapy after failed medical abortion
254. The role of parity in medical abortion up to 49 days of amenorrhoea
255. Endometrial thickness and serum beta-hCG as predictors of the effectiveness of oral misoprostol in early pregnancy failure
256. Comparison of vaginal and sublingual misoprostol for second trimester abortion: randomized controlled equivalence trial
257. Complications after second trimester surgical and medical abortion
258. Second trimester medical abortion with mifepristone-misoprostol and misoprostol alone: a review of methods and management
259. Second trimester abortions in India
260. High failure rates of medical termination of pregnancy after introduction to a large teaching hospital
261. Age, parity, history of abortion and contraceptive choices affect the risk of repeat abortion
262. Misoprostol use as a method of medical abortion
263. Integrating medical abortion into safe abortion services: experience from three pilot sites in South Africa
264. First trimester medical termination of pregnancy: The Nottingham experience
265. Misoprostol and pregnancy: risk of malformations
266. Medical treatment with misoprostol for early failure of pregnancies after assisted reproductive technology: a promising treatment option
267. A randomised comparative study on sublingual versus vaginal administration of misoprostol for termination of pregnancy between 13 to 20 weeks
268. Ibuprofen and paracetamol for pain relief during medical abortion: a double-blind randomized controlled study
269. Termination of early pregnancy using flexible, low-dose mifepristone-misoprostol regimens
270. [Clinical study of terminating biochemical pregnancy and early clinical pregnancy with mifepristone and misoprostol]
271. Toxic shock associated with Clostridium sordellii and Clostridium perfringens after medical and spontaneous abortion
272. Pathopharmacology of excessive hemorrhage in mifepristone abortions
273. Reproductive choice for women and men living with HIV: contraception, abortion and fertility
274. Risk factors for unsuccessful medical abortion with mifepristone and misoprostol
275. Does methotrexate confer a significant advantage over misoprostol alone for early medical abortion? A retrospective analysis of 8678 abortions
276. Mifepristone and misoprostol administered simultaneously versus 24 hours apart for abortion: a randomized controlled trial
277. Randomised controlled trial comparing the efficacy of same-day administration of mifepristone and misoprostol for termination of pregnancy with the standard 36 to 48 hour protocol
278. Alternatives to mifepristone for early medical abortion
279. Monitoring medical abortion using mifepristone/misoprostol combination with ultrasonogram and serum human chorionic gonadotropin
280. Vaginal misoprostol for salvage therapy after failed medical abortion
281. Use of mifepristone and sublingual misoprostol for early medical abortion
282. Clinical applications of mifepristone
283. Development and evaluation of the abortion attributes questionnaire
284. [Ambulatory medical abortion performed in a family planning center]
285. [Antiprogesterone for medical abortion]
286. Evidence for shortening the time interval of prostaglandin after mifepristone for medical abortion
287. Analysis of severe adverse events related to the use of mifepristone as an abortifacient
288. Consistency of medical abortion efficacy from 5 through 14 weeks' gestation
289. Medical abortion in rural Tamil Nadu, South India: a quiet transformation
290. Randomized trial of mifepristone and buccal or vaginal misoprostol for abortion through 56 days of last menstrual period
291. Randomised controlled trial comparing efficacy of same day administration of mifepristone and misoprostol for termination of pregnancy with the standard 36- to 48-hour protocol
292. Home self-administration of misoprostol for medical abortion up to 56 days' gestation
293. The effect of non-steroidal anti-inflammatory drugs on medical abortion with mifepristone and misoprostol at 13-22 weeks gestation
294. Pathophysiology of mifepristone-induced septic shock due to Clostridium sordellii
295. A randomised controlled trial of mifepristone in combination with misoprostol administered sublingually or vaginally for medical abortion up to 13 weeks of gestation
296. Introduction of early medical abortion in New Zealand: an audit of the first 67 cases
297. [Medical abortion using methotrexate and misoprostol. Efficacy and tolerability]
298. [Mifepristone 100 mg for early medical abortion]
299. [The efficacy and acceptability of mifepristone medical abortion with home administration misoprostol provided by private providers linked with the hospital: a prospective study of 433 patients]
300. A randomized trial of mifepristone in combination with misoprostol administered sublingually or vaginally for medical abortion at 13-20 weeks gestation
301. A pilot study of mifepristone and misoprostol administered at the same time for abortion up to 49 days gestation
302. Medical abortion at 9-13 weeks' gestation: a review of 1076 consecutive cases
303. [Side effects of induced abortion: results from a population-based survey]
304. Failed medical termination of twin pregnancy with mifepristone: a case report
305. Multicenter trial of a simplified mifepristone medical abortion regimen
306. Accounting for time: insights from a life-table analysis of the efficacy of medical abortion
307. A fatal case of Clostridium sordellii septic shock syndrome associated with medical abortion
308. Results and lessons learned from a small medical abortion clinical study in Turkey
309. Acceptability of home-use of misoprostol in medical abortion
310. Increased risk for medical abortion failure for multiparous women
311. Randomized, double-blind, controlled trial of mifepristone in capsule versus tablet form followed by misoprostol for early medical abortion
312. Misoprostol as the primary agent for medical abortion in a low-income urban setting
313. Methods for induced abortion
314. Ultrasound evaluation of the endometrium after medical termination of pregnancy
315. Role of routine ultrasonography in monitoring the outcome of medical abortion in a clinical setting
316. Midtrimester medical termination of pregnancy: a review of 1002 consecutive cases
317. Prediction of late failure after medical abortion from serial beta-hCG measurements and ultrasonography
318. Current medical abortion care
319. Medical abortion. defining success and categorizing failures
320. [Medical termination of pregnancy with mifepristone--initial experience at the Sheba Medical Center]
321. Methotrexate/misoprostol embryopathy: report of four cases resulting from failed medical abortion
322. Successful medical abortion of a pregnancy within a noncommunicating rudimentary uterine horn
323. WHO multinational study of three misoprostol regimens after mifepristone for early medical abortion. I: Efficacy
324. Medical abortion in family practice: a case series
325. [Large variation in clinical regimens use to induce medical abortion in Denmark]
326. Medical abortion at 64 to 91 days of gestation: a review of 483 consecutive cases
327. A viable alternative to surgical vacuum aspiration: repeated doses of intravaginal misoprostol over 9 hours for medical termination of pregnancies up to eight weeks
328. Factors affecting the outcome of early medical abortion: a review of 4132 consecutive cases
329. Options for early therapeutic abortion: a comparative review
330. Randomized trial of oral versus vaginal misoprostol 2 days after mifepristone 200 mg for abortion up to 63 days of pregnancy
331. Suggested FDA restrictions might curtail access to mifepristone
332. Clinical observation on termination of early pregnancy of 213 cases after caesarian section with repeated use of mifepristone and misoprostol
333. Heads Up! Potential legal obstacles to medical abortion
334. Manual vacuum aspiration technique draws interest
335. The political challenges and educational opportunities around very early abortion
336. Cutting unsafe abortions
337. Mifepristone-misoprostol abortion: a trial in rural and urban Maharashtra, India
338. Medical abortion with mifepristone and misoprostol: a clinical trial in Taiwanese women
339. [Use of misoprostol in gynecology and obstetrics]
340. A prospective randomized, double-blinded, placebo-controlled trial comparing mifepristone and vaginal misoprostol to vaginal misoprostol alone for elective termination of early pregnancy
341. Medical abortion in the first trimester
342. Could American women use mifepristone-misoprostol pills safely with less medical supervision?
343. Medical abortion as an alternative to vacuum aspiration: first experiences with the 'abortion pill' in The Netherlands
344. Mifepristone abortion in minors
345. Randomized trial of oral versus vaginal misoprostol at one day after mifepristone for early medical abortion
346. Early pregnancy termination with mifepristone and misoprostol in Norway
347. Double-blind randomized trial of mifepristone in combination with vaginal gemeprost or misoprostol for induction of abortion up to 63 days gestation
348. Second trimester medical abortion with mifepristone and gemeprost: a review of 956 cases
349. Lowering the doses of mifepristone and gameprost for early abortion: a randomised controlled trial. World Health Organization Task Force on Post-ovulatory Methods for Fertility Regulation
350. First trimester abortion with mifepristone and vaginal misoprostol
351. Medical abortion at 57 to 63 days' gestation with a lower dose of mifepristone and gemeprost. A randomized controlled trial
352. [Clinical study of four cases with malignant gestation trophoblastic tumor after mifepristone abortion]
353. Parity is a major determinant of success rate in medical abortion: a retrospective analysis of 3161 consecutive cases of early medical abortion treated with reduced doses of mifepristone and vaginal gemeprost
354. Vaginal misoprostol administered 1, 2, or 3 days after mifepristone for early medical abortion: A randomized trial
355. Legal issues in the provision of medical abortion
356. Medical abortion: public health and private lives
357. Abortion reporting in the era of medical procedures: why is it important?
358. Medical abortion in China
359. Misoprostol alone--a new method of medical abortion?
360. Advanced practice clinicians and medical abortion: increasing access to care
361. The counseling component of medical abortion
362. Are US health professionals likely to prescribe mifepristone or methotrexate?
363. Vaginal misoprostol alone for medical abortion up to 9 weeks of gestation: efficacy and acceptability
364. Comparison of two doses of mifepristone in combination with misoprostol for early medical abortion: a randomised trial
365. Low-dose mifepristone followed by vaginal misoprostol at 48 hours for abortion up to 63 days
366. The efficacy of medical abortion: a meta-analysis
367. Methotrexate and misoprostol used alone or in combination for early abortion
368. [The first 100 early medical abortions]
369. Tamoxifen compared to methotrexate when used with misoprostol for abortion
370. Methotrexate and misoprostol for early abortion in adolescent women
371. Providing mifepristone-misoprostol medical abortion: the view from the clinic
372. The influence of abortion legislation on maternal mortality
373. Abortion incidence and services in the United States, 1995-1996
374. Blood loss with mifepristone--misoprostol abortion: measures from a trial in China, Cuba and India
375. An effective regimen for early medical abortion: a report of 2000 consecutive cases
376. A pilot study of the effect of methotrexate or combined oral contraceptive on bleeding patterns after induction of abortion with mifepristone and a prostaglandin pessary
377. Acceptability and feasibility of early pregnancy termination by mifepristone-misoprostol. Results of a large multicenter trial in the United States. Mifepristone Clinical Trials Group
378. Mifepristone (RU 486): current knowledge and future prospects
379. Oral methotrexate and vaginal misoprostol for early abortion
380. Early pregnancy termination with mifepristone and misoprostol in the United States
381. Assessing strength of preference for abortion method using 'willingness to pay': a useful research technique for measuring values
382. Medical abortion with methotrexate 75 mg intramuscularly and vaginal misoprostol
383. [Should antiprogesterone be used in pregnancy termination?]
384. Abortion induced with methotrexate and misoprostol: a comparison of various protocols
385. A randomised study of two doses of gemeprost in combination with mifepristone for induction of abortion in the second trimester of pregnancy
386. [A phase III multicentre study on medical termination of early pregnancy with two regimens of mifepristone followed by PG05]
387. Change in serum beta-human chorionic gonadotropin after abortion with methotrexate and misoprostol
388. Medical abortion: what does the research tell us?
389. When is "emergency" contraception the right name for postcoital treatment?
390. Factors hindering access to abortion services
391. [Termination of early pregnancy by two regimens of mifepristone with misoprostol: a multicentre clinical trial]
392. Termination of early pregnancy by two regimens of mifepristone with misoprostol and mifepristone with PG05--a multicentre randomized clinical trial in China
393. A comparison of medical abortion (using mifepristone and gemeprost) with surgical vacuum aspiration: efficacy and early medical sequelae
394. Psychological responses following medical abortion (using mifepristone and gemeprost) and surgical vacuum aspiration. A patient-centered, partially randomised prospective study
395. Methotrexate and misoprostol vs misoprostol alone for early abortion. A randomized controlled trial
396. Comparison of medical abortion with surgical vacuum aspiration: women's preferences and acceptability of treatment
397. Abortion
398. The effect of dose of mifepristone and gestation on the efficacy of medical abortion with mifepristone and misoprostol
399. Further acceptability evaluation of RU486 and ONO 802 as abortifacient agents in a Chinese population
400. [Non-surgical female sterilization using quinacrine: efficacy of two insertions of quinacrine pellets]
401. [Clinical study of termination of early pregnancy by combination of dl-15-methyl-prostaglandin F2 alpha and RU 486]
402. Medical abortion in women of less than or equal to 56 days amenorrhoea: a comparison between gemeprost (a PGE1 analogue) alone and mifepristone and gemeprost
403. Psychiatric morbidity and acceptability following medical and surgical methods of induced abortion
404. Future direction of abortion technology
405. Medical methods to terminate early pregnancy
406. The use of prostaglandins and antiprogestins for pregnancy termination
407. [Craniorachischisis in conjoined "diprosopus" twins. Case report and review of the literature]
408. [Experiences with the antigestagen mifepristone (RU 486) in the interruption of early pregnancy]
409. Anti-progesterones for the interruption of pregnancy
410. The unwanted child
411. Medical abortion complications. An epidemiologic study at a mid-Missouri clinic
412. [The gynecologist and the problem of therapeutic abortion]

**1.2 Records identified through Embase (n=541)**

1. Abortion medical management between 14−16 weeks’ amenorrhea after French legislation deadline extension
2. Development and pilot testing of the 2019 Canadian Abortion Provider Survey
3. Telephone follow-up after early medical abortion using Australia's first low sensitivity urine pregnancy test
4. Impact of the Covid-19 Pandemic on the Prevalence of MTP Cases and Their Clinicodemographic Profile in India: A Retrospective Multicentric Study
5. Medical abortion in the second trimester - an update
6. Pain assessment during medical abortion up to 14 weeks: A 1-year prospective comparative study
7. A comparison of misoprostol with and without methylergometrine and oxytocin in outpatient medical abortion: a phase III randomized controlled trial
8. Pharmacy dispensing of abortion pills in Ghana: Experiences of pharmacy workers and users
9. Failure Rate of Medical Treatment for Miscarriage Correlated with the Difference between Gestational Age According to Last Menstrual Period and Gestational Size Calculated via Ultrasound
10. Using telemedicine to improve early medical abortion at home (UTAH): A randomised controlled trial to compare telemedicine with in-person consultation for early medical abortion
11. Misoprostol-Induced Fever and Unnecessary Antibiotic Prescribing: A Retrospective Study
12. "I will never wish this pain to even my worst enemy": Lived experiences of pain associated with manual vacuum aspiration during postabortion care in Kenya
13. Accessing abortion in a highly restrictive legal regime: Characteristics of women and pregnant people in Malta self-managing their abortion through online telemedicine
14. Outcome of First Trimester Induced Abortions Using Misoprostol by Buccal and Vaginal Routes
15. TRENDS IN ELECTIVE MEDICAL AND SURGICAL ABORTIONS IN THE UNITED STATES DURING THE COVID-19 PANDEMIC
16. An examination of loss to follow-up and potential bias in outcome ascertainment in a study of direct-to-patient telemedicine abortion in the United States
17. Structural barriers or patient preference? A mixed methods appraisal of medical abortion use in England and Wales
18. Medical abortion: a practical quick reference algorithm to support the gynecologists
19. Effectiveness and acceptability of home use of misoprostol for medical abortion up to 10 weeks of pregnancy
20. Parsing the Q-Markers of Baoyin Jian to Treat Abnormal Uterine Bleeding by High-Throughput Chinmedomics Strategy
21. Diagnosis of medulloblastoma in pregnant women: To continue with the pregnancy or not? A case report and literature review
22. Acute uterine inversion following an induced abortion
23. Comparing the Effects of Misoprostol/Letrozole and Misoprostol/Placebo on Medical Abortion Success Rate: A Randomized Clinical Trial
24. Self-administration of Abortion Pills and its Maternal Outcome in Tertiary Care Center
25. The effect of misoprostol with and without letrozole in inducing successful medical abortion
26. Trend of serum beta-human chorionic gonadotropin levels after medical abortion in the early first trimester of pregnancy
27. Management and treatment of brain tumors during pregnancy: an Italian survey
28. Ultrasonographic features of the endometrium following successful medical termination of early pregnancy
29. Retrospective Observational Assessment of the Hazards of OTC Availability of MTP Pills
30. The contraceptive choice at the time of a surgical and pharmacological abortion: a possible and effective option thanks to a dedicate counselling
31. Comparative study of regimen mifepristone and misoprostol with misoprostol alone at first trimester termination of pregnancy
32. Oncologic and Fertility Outcomes after Simple Trachelectomy in Women with Early Cervical Cancer
33. Which evacuation method for missed abortion is preferred by women with recurrent pregnancy loss?
34. The impact of provider restrictions on abortion-related outcomes: a synthesis of legal and health evidence
35. Validation of a Spanish-language scale for evaluating perceived quality of care of medical abortions before 9 weeks gestation
36. A Retrospective Service Evaluation of Early Medical Abortion at Home by a District General Hospital during the COVID-19 Pandemic -2020 to 2022
37. Medical Abortion Self Use in Kenya: Results from a process evaluation of women's experiences
38. Accessing abortion in a highly restrictive legal regime: characteristics of women and pregnant people in Malta self-managing their abortion through online telemedicine
39. Myometritis with pelvic septic vein thrombophlebitis secondary to Fusobacterium necrophorum sepsis
40. Willing but not able: A survey of New Zealand health practitioners' interests in providing second trimester abortion care and the obstacles they face
41. The efficacy of Shenghua Decoction supplementation after early medical abortion: A meta-analysis of randomized controlled trials
42. Prevalence of Anaemia in Women with Unsupervised Medical Abortion- An Observational Study
43. A Study of Fertility and Pregnancy Outcomes in Women after Surgery for Valvular Heart Disease
44. Client perspectives on choice of abortion method in England and Wales
45. Predictive value of peripheral blood α1-acid glycoprotein in medical abortion outcomes with mifepristone and relativity of concentration
46. Medical methods for first trimester abortion
47. Evaluation of the success of medical abortion by a plasma hCG control threshold
48. Abortion policy implementation in Ireland: Lessons from the community model of care
49. Barriers and Enablers to Nurse Practitioner Provision of Medication Abortion in Canada: Results from a National Survey
50. 346 Efficacy and safety of mifepristone and sublingual misoprostol for second trimester medical abortion. A prospective study
51. A case of fetal methotrexate syndrome-a rare cause of multiple congenital anomalies
52. PXR mediates mifepristone-induced hepatomegaly in mice
53. 10-year evaluation of the use of medical abortion through telemedicine: a retrospective cohort study
54. Accuracy of anemia screening by point-of-care hemoglobin testing in patients seeking abortion
55. FIRST TRIMESTER MEDICAL ABORTION PRACTICE IN NORTH EAST INDIA
56. Profile of Women receiving Second-trimester Safe Abortion Service at Paropakar Maternity and Women’s Hospital
57. Self administration versus supervised use of Medical Abortion pills: Impact on women’s health
58. Clinical analysis of second-trimester pregnancy termination after previous caesarean delivery in 51 patients with placenta previa and placenta accreta spectrum: a retrospective study
59. It’s a small bit of advice, but actually on the day, made such a difference…: perceptions of quality in abortion care in England and Wales
60. Effectiveness and acceptability of “at home” versus “at hospital” early medical abortion – A lesson from the COVID-19 pandemic: A retrospective cohort study
61. Subsequent placenta accreta after previous mifepristone-induced abortion: A case report
62. Declining risk of surgical intervention following early medical abortion: A time trend analysis
63. Termination of pregnancy services in Irish general practice from January 2019 to June 2019
64. Combined vesicouterine rupture during second-trimester medical abortion for fetal abnormality after prior cesarean delivery: A case report
65. Telemedicine medical abortion at home under 12 weeks' gestation: A prospective observational cohort study during the COVID-19 pandemic
66. Cervical Twin Heterotopic Pregnancy: Overview of Ectopic Pregnancies and Scanning Detection Algorithm
67. Surgical or medical abortion of pregnancies between 13 +0 and 23 +6 weeks' gestation? A systematic review and new NICE national guidelines
68. UTAH: Using Telemedicine to improve early medical Abortion at Home: A protocol for a randomised controlled trial comparing face-to-face with telephone consultations for women seeking early medical abortion
69. Medical abortion offered in pharmacy versus clinic-based settings
70. If you were to have another abortion, would you choose the same method? A study on 1032 patients’ level of satisfaction
71. Abstract #1002287: A Case of Diabetic Ketoacidosis in Medical Management of Miscarriage
72. Clinician-led transformation of sexual and reproductive healthcare in NI post-decriminalisation of abortion
73. User-centred design and development of a patient decision aid for choice of first trimester abortion method
74. Expulsion at home for early medical abortion: A systematic review with meta-analyses
75. How the coronavirus disease 2019 pandemic is impacting sexual and reproductive health and rights and response: Results from a global survey of providers, researchers, and policy-makers
76. Medical management of induced and incomplete first-trimester abortion by non-physicians in low- and middle-income countries: A systematic review and meta-analysis of randomized controlled trials
77. Two prophylactic pain management regimens for medical abortion ≤63 days' gestation with mifepristone and misoprostol: A multicenter, randomized, placebo-controlled trial
78. Comparison of the efficacy and safety of two combined misoprostol regimens for second trimester medical abortion
79. Second trimester medical abortion in a primigravida with lupus nephritis and rapidly progressive renal failure: challenges and outcome
80. Systematic Review of the Effectiveness, Safety, and Acceptability of Mifepristone and Misoprostol for Medical Abortion in Low- and Middle-Income Countries
81. Chemical Fingerprint Analysis and Ultra-Performance Liquid Chromatography Quadrupole Time-of-Flight Mass Spectrometry-Based Metabolomics Study of the Protective Effect of Buxue Yimu Granule in Medical-Induced Incomplete Abortion Rats
82. Simultaneous compared to interval administration of mifepristone and misoprostol for medical abortion up to 10 +0 weeks' gestation: a systematic review with meta-analyses
83. Risk of surgical evacuation and risk of major surgery following second-trimester medical abortion in Denmark: A nationwide cohort study
84. A repeat dose of misoprostol 800 mcg following mifepristone for outpatient medical abortion at 64–70 and 71–77 days of gestation: A retrospective chart review
85. Medical termination for pregnancy in early first trimester (≤ 63 days) using combination of mifepristone and misoprostol or misoprostol alone: A systematic review
86. Management of pain associated with up-to-9-weeks medical termination of pregnancy (MToP) using mifepristone–misoprostol regimens: expert consensus based on a systematic literature review
87. 2017–19 governmental decisions to allow home use of misoprostol for early medical abortion in the UK
88. Women's voices and medical abortions: A review of the literature
89. Follow-up strategies to confirm the success of medical abortion of pregnancies up to 10 weeks’ gestation: a systematic review with meta-analyses
90. Medical abortion
91. Women's self-reported experiences using misoprostol obtained from drug sellers: A prospective cohort study in Lagos State, Nigeria
92. A non-inferiority study of outpatient mifepristone-misoprostol medical abortion at 64–70 days and 71–77 days of gestation
93. Self-administered versus provider-administered medical abortion
94. Current and potential methods for second trimester abortion
95. First trimester termination of pregnancy
96. Modern methods to induce abortion: Safety, efficacy and choice
97. Prevalence of self induced abortion by self-administration of abortive pills among abortion-related admissions in a tertiary care centre
98. Conflicting recommendations between the French national authority for health (HAS) and clinical practice guidelines (CNGOF); focus on 200 late medical abortions, conducted outside marketing authorizations
99. Cesarean scar ectopic pregnancy: The lurking danger in post cesarean failed medical abortion
100. General practitioner knowledge and practice in relation to unintended pregnancy in the Grampians region of Victoria, Australia
101. Association Between The Route of Administration and Efficacy of The Misoprostol in The Management of Missed Abortion: A Randomized Clinical Trial
102. Pregnancy in antiphospholipid syndrome: Outcomes and risk factors-data from a portuguese multidisclinary unit
103. Medical abortion with mifepristone and vaginal misoprostol between 64 and 70days' gestation
104. Follow-up Rates and Contraceptive Choices after Medical Abortion in Adolescents at Cook County Hospital
105. Comparison between the effectiveness of combination of letrozole with misoprostol and tamoxifen with misoprostol in medical termination of first trimester missed miscarriage
106. Telemedicine for medical abortion: a systematic review
107. A prospective study of mifepristone and unlimited dosing of sublingual misoprostol for termination of second-trimester pregnancy in Uzbekistan and Ukraine
108. Expanding a woman's options to include home use of misoprostol for medical abortion up until 76 days: an observational study of efficacy and safety
109. A comparison of the safety and efficacy of two medical abortion protocols
110. Initiating intramuscular depot medroxyprogesterone acetate 24-48 hours after mifepristone administration does not affect success of early medical abortion
111. Pregnancy outcomes in antiphospholipid syndrome: 8 year-experience from a multidisciplinary unit
112. Outcomes During Early Implementation of Mifepristone-Buccal Misoprostol Abortions up to 63 Days of Gestation in a Canadian Clinical Setting
113. Early abortion with buccal versus sublingual misoprostol alone: a multicenter, randomized trial
114. Efficacy of Misoprostol Alone for First-Trimester Medical Abortion: A Systematic Review
115. Determinants and Outcome of Safe Second Trimester Medical Abortion at Jimma University Medical Center, Southwest Ethiopia
116. Clinical approach and surgical strategy for spinal disorders in pregnant women
117. Outcome of first trimester medical termination of pregnancy: definitions and management
118. Scar Ectopic Pregnancy Type II / Endogenic with Impending Scar Rupture
119. Laparoscopic Management of Caesarean Scar Ectopic Pregnancy A Report of 2 Cases
120. Acceptability and feasibility of outpatient medical abortion with mifepristone and misoprostol up to 70 days gestation in Singapore
121. Comparative study of efficacy and acceptability of two regimens for 1st trimester MTP
122. Lessons learned from introducing dilation and evacuation in tigray region of Ethiopia
123. Cost effective & scalable community models for safe abortion in low resource countries in South Asia
124. Efficacy of mifepristone-misoprostol combination in cases of missed abortion in first trimester
125. Outcome of managing second trimester post abortion cases using a standardized combination of regimen
126. Laparoscopic management of caesarean scar ectopic pregnancy-a report of 2 cases
127. Review of the feasibility of mifepristone & misoprostol for medical abortion in developing Countries
128. New frontiers in abortion care
129. Challenging cases in care for termination of pregnancy
130. Complications related to induced abortion: A combined retrospective and longitudinal follow-up study
131. Successful surgical treatment of invasive pancreatic ductal adenocarcinoma during pregnancy
132. Early medical abortion with self-administered low-dose mifepristone in combination with misoprostol
133. Deciphering the voices in abortion care: The woman's voice matters
134. A retrospective cohort analysis of retained products of conception with vascularity after second-trimester abortion;incidence, outcomes in expectant management, and characteristic ultrasonographic features leading to future severe hemorrhage
135. Is self-medication with over the counter abortion pills really safe? An experience at a tertiary care centre
136. Simultaneous Compared With Interval Medical Abortion Regimens Where Home Use Is Restricted: Correction
137. Providing accessible medical abortion services in a Victorian rural community: A description and audit of service delivery and contraception follow up
138. A direct-to-patient telemedicine abortion service in Australia: Retrospective analysis of the first 18 months
139. Changes in abortion service provision in Bihar and Jharkhand states, India between 2004 and 2013
140. Abortion education in Canadian family medicine residency programs
141. The telabortion project: Delivering the abortion pill to your doorstep by telemedicine and mail
142. A direct-to-patient telemedicine abortion service in Australia: Retrospective analysis of the first 18 months
143. Simultaneous administration of mifepristone and misoprostol increases access to early medical abortion where home-use of misoprostol is not permitted
144. Adult high-risk Burkitt’s acute lymphocytic leukemia was successfully rescued by rituximab combined with hyper-CVAD/MA regimens: Two case reports and a literature review
145. Simplified medical abortion screening: a demonstration project
146. Self-assessment of medical abortion outcome using symptoms and home pregnancy testing
147. Factors associated with follow-up rates after medical abortion in adolescents at cook county hospital
148. Risk factors and the choice of long-acting reversible contraception following medical abortion: effect on subsequent induced abortion and unwanted pregnancy
149. Evaluation of effect of letrozole prior to misoprostol in comparison with misoprostol alone in success rate of induced abortion
150. Provision of medical abortion by midlevel healthcare providers in Kyrgyzstan: testing an intervention to expand safe abortion services to underserved rural and periurban areas
151. Experience of clandestine use of medical abortion among university students in Chile: a qualitative study
152. Efficacy of medical abortion prior to 6 gestational weeks: a systematic review
153. Peri-abortion care: When is it too much and when too little?
154. Safe medical abortion in the second trimester of pregnancy
155. Comparison of two low-sensitivity urine pregnancy tests for confirming the success of early medical abortion
156. Protective Effect of Taohong Siwu Decoction on Abnormal Uterine Bleeding Induced by Incomplete Medical Abortion in Rats during Early Pregnancy
157. Rudimentary uterine horn pregnancy in 15 cases: Case series with a two-year follow-up
158. Simultaneous compared with interval medical abortion regimens where home use is restricted
159. Changes in abortion service provision in Bihar and Jharkhand states, India between 2004 and 2013
160. Corrections: Simultaneous Compared With Interval Medical Abortion Regimens Where Home Use Is Restricted (Obstetrics and Gynecology (2018) 131 (635-41) DOI: 10.1097/AOG.0000000000002536)
161. Clinical approach and surgical strategy for spinal disorders in pregnant women
162. Medical abortion in the community setting
163. A classic case of toxic shock syndrome due to a not so classic organism, clostridium sordellii
164. A retrospective cohort analysis of retained products of conception with vascularity after second-trimester abortion: Incidence, outcomes, and ultrasonographic features leading to severe hemorrhage
165. A maternal death from self-induced medical abortion: a call for action
166. Experiences of women living in Hungary seeking a medical abortion online
167. Follow-up after early medical abortion: Comparing clinical assessment with self-assessment in a rural hospital in northern Norway
168. Efficacy and safety of mifepristone–buccal misoprostol for early medical abortion in an Australian clinical setting
169. Abortion pill: A boon or a curse?
170. A comparison of medical and surgical management of first trimester incomplete miscarriages- a randomised control trial in PMGH
171. Results of first trimester medical abortion care
172. Is self-assessment of medical abortion using a low-sensitivity pregnancy test combined with a checklist and phone text messages feasible in South African primary healthcare settings? A randomized trial
173. Second-trimester surgical and medical abortion practice in Canada in 2012: A national survey
174. Use of an at-home multilevel pregnancy test and an automated telephone system to simplify medical abortion follow-up
175. 2017 NAF AM Abstracts
176. Intrauterine contraception after medical abortion: factors affecting success of early insertion
177. Factors related to completeness of medical abortion with mifepristone and misoprostol
178. Intra-abdominal hemorrhage induced by medical abortion in mid-trimester pregnancy: An unusual presentation of placenta percreta
179. Self reported outcomes and adverse events after medical abortion through online telemedicine: Population based study in the Republic of Ireland and Northern Ireland
180. Retrospective assessment of the influence of gestational age on the first trimester medical abortion efficiency
181. Prostaglandins and mifepriston in stimulation of medical abortion
182. Comparative Study of Mifepristone with Vaginal Misoprostol for First Trimester Termination of Pregnancy at Different Gestational Ages
183. ‘…a one stop shop in their own community’: Medical abortion and the role of general practice
184. Abortion law in France
185. Elevated mRNA expression of PGF2α receptor splice variant 2(FP-V2) in human decidua is associated with incomplete mifepristone–misoprostol-induced early medical abortion by regulation of interleukin-8
186. Immediate versus delayed insertion of an etonogestrel releasing implant at medical abortion - A randomized controlled equivalence trial
187. Outpatient mifepristone-misoprostolmedical abortion through 77 days of gestation
188. Prospective study of home use of mifepristone and misoprostol for medical abortion up to 10 weeks of pregnancy in Kazakhstan
189. Pregnancy termination in a female with paraplegia: A case report
190. Uncomplicated abortion with mifepristone and misoprostol in a hemophilia A carrier
191. The role of medical abortion in the implementation of the law on voluntary termination of pregnancy in Uruguay
192. The role of medical abortion in the implementation of the law on voluntary termination of pregnancy in Uruguay
193. Self-administered multi-level pregnancy tests in simplified follow-up of medical abortion in Tunisia
194. Scenario of self medication for medical abortion in a tertiary care centre
195. Medical abortion provided by nurse-midwives or physicians in a high resource setting: A cost-effectiveness analysis
196. Dealing with the barriers to access to abortion
197. Decentralising medical abortion services in Victoria, Australia
198. Instruction-only versus demonstration of a low sensitivity pregnancy test for self-assessment of medical abortion in South Africa; a multicentre non-inferiority randomised controlled trial
199. Efficacy and safety of the medical method in the voluntary interruption pregnancy over 8 years-experience of a Portuguese medical centre
200. Safety and efficacy of outpatient mifepristone-misoprostol medical abortion through 76 days of gestational age-Portuguese experience in a tertiary hospital
201. Medical abortion in second trimester missed abortion pregnancies
202. Termination of a pregnancy located in one horn of a bicornuate uterus: Challenges and solution
203. Outpatient mifepristone-misoprostol medical abortion through 77 days of gestation
204. The outcome of home medical abortions provided through telemedicine
205. Effect of Immediate Compared With Delayed Insertion of Etonogestrel Implants on Medical Abortion Efficacy and Repeat Pregnancy
206. Safety and feasibility of office hysteroscopy in removing retained products of conception
207. Exhaustion in myeloid lineage and very early defect in HSPC pool: An embryonic origin of fanconi haematological disorders
208. Assessment of the efficacy of different methods of second trimester abortion: An initial experience in eastern Nepal
209. Midwives, nurses and doctors performing MVA in Vietnam and South Africa and early medical abortion in Nepal: Evidence from randomized, controlled trials
210. Medical abortion outcomes and repeat pregnancy following quickstart of contraceptive implants and depot-medroxyprogesterone acetate
211. Early versus delayed insertion of nexplanon at medical abortion-a randomized controlled equivalence trial
212. The effectiveness of home medical abortions provided through telemedicine
213. Standardized second trimester medical abortion training and service provision in Ethiopia, october 2010-september 2014
214. Does starting progestin-only contraception on the same day as a medical abortion with mifepristone affect completion of the abortion? A review of clinic records from Mexico City
215. Randomized trial of mifepristone and buccal misoprostol vs misoprostol alone for medical abortion
216. Progestin-based contraceptive on the same day as medical abortion
217. Medical abortion-let us give quality care! making medical abortion safer and more effective in low resource population. A comparative study from private hospital, Nagpur India
218. Medical abortion outcomes following quickstart of contraceptive implants and depot-medroxyprogesterone acetate
219. Office versus telephone follow-up after medical abortion
220. Safety and efficacy of medical abortion in a pediatric population
221. Polidocanol foam for nonsurgical permanent female contraception: Initial trial in baboons
222. Efficacy of mifepristone and misoprostol in late first trimester medical abortion, missed abortion and blighted ovum
223. Early versus late misoprostol administration after mifepristone for medical abortion
224. Safety of induced abortions at less than 12 weeks of pregnancy in Japan
225. Nurse versus physician-provision of early medical abortion in Mexico: A randomized controlled non-inferiority trial
226. Clinical follow-up compared with self-assessment of outcome after medical abortion: A multicentre, non-inferiority, randomised, controlled trial
227. Clinical follow-up compared with self-assessment of outcome after medical abortion: a multicentre, non-inferiority, randomised, controlled trial
228. Administration of depot medroxyprogesterone acetate on the day of mifepristone for medical abortion: A pilot study
229. The introduction of first trimester medical abortion in Armenia
230. Assessment of completion of early medical abortion using a text questionnaire on mobile phones compared to a self-administered paper questionnaire among women attending four clinics, Cape Town, South Africa
231. The role of auxiliary nurse-midwives and community health volunteers in expanding access to medical abortion in rural Nepal
232. Medical Abortion: Use of Mifepristone and Misoprostol in First and Second Trimesters of Pregnancy
233. Effect of Wujia Shenghua capsule on uterine bleeding following medically-induced incomplete abortion in rats during early pregnancy
234. Mifepristone With Buccal Misoprostol for Medical Abortion
235. Is it safe to provide abortion pills over the counter? A study on outcome following self-medication with abortion pills
236. Self-assessment of the outcome of early medical abortion versus clinic follow-up in India: A randomised, controlled, non-inferiority trial
237. Medical Students' Intentions to Seek Abortion Training and to Provide Abortion Services in Future Practice
238. Change in abortion services after implementation of a restrictive lawin Texas
239. Efficacy and acceptability of a mifepristone-misoprostol combined regimen for early induced abortion among women in Mexico City
240. The feasibility of offering medical abortions by tele medicine: 20 Months of experience
241. Abortion providers' resilience to antichoice tactics in the United States and Canada
242. Online availability of mifepristone and misoprostol
243. Postabortion contraception method mix and uptake in IPAS-supported public health facilities in ethiopia
244. Hysteroscopic resection or conventional blind curettage in the management of retained products of conception, a systematic review
245. Pregnancy course in patients with interstitial lung diseases
246. Attributes and perspectives of public providers related to provision of medical abortion at public health facilities in vietnam: A cross-sectional study in three provinces
247. A randomized trial of hospital vs home self administration of vaginal misoprostol for medical abortion
248. Achievements of the FIGO Initiative for the Prevention of Unsafe Abortion and its Consequences in South-Southeast Asia
249. Replacement of dilation and curettage/evacuation by manual vacuum aspiration and medical abortion, and the introduction of postabortion contraception in Pakistan
250. Evidence-based practice-fact or fiction?
251. Routine follow-up versus self-assessment of complete abortion following medical abortion, effect on its success and acceptability: A randomized controlled trial
252. Discussing the best way of follow-up
253. Feasibility of a self-performed urinary test for the follow-up on medical abortion: The Betina study
254. Outcomes of legal induced abortion at 9-10 weeks ' gestation in adult women
255. First pregnancy abortion as an infectious complications risk factor
256. Medical abortion after 9 weeks
257. A two-pill sublingual misoprostol outpatient regimen following mifepristone for medical abortion through 70 days' LMP: A prospective comparative open-label trial
258. Medical management of first-Trimester abortion
259. Provision of medical abortion using telemedicine in Brazil
260. Minimal effective dose of mifepristone for medical abortion
261. Uterine preservation surgery for placental polyp
262. Practice bulletin no. 143: medical management of first-trimester abortion.
263. Medical abortion with mifepristone and home administration of misoprostol up to 63 days' gestation
264. [Clinical study on 39 cases with caesarean scar pregnancy with sonographic mass].
265. Achievements of the FIGO Initiative for the Prevention of Unsafe Abortion and its Consequences in South-Southeast Asia
266. Replacement of dilation and curettage/evacuation by manual vacuum aspiration and medical abortion, and the introduction of postabortion contraception in Pakistan
267. [Medical abortion for first-trimester termination of the pregnancy].
268. A prospective trial using mifepristone and vaginal misoprostol in termination of pregnancies up to 63 days of gestation
269. Ectopic pregnancy in the era of medical abortion: Are we ready for it? Spectrum of sonographic findings and our experience in a tertiary care service hospital of India
270. Triangular assessment of the etiology of induced abortion in Iran: A qtualitative study
271. Comprehensive abortion care: Evidence of improvements in hospital-level indicators in Tigray, Ethiopia
272. Prophylactic compared with therapeutic ibuprofen analgesia in first-trimester medical abortion: A randomized controlled trial
273. Depot medroxyprogesterone acetate (DMPA) administration at the time of mifepristone for medical abortion: A pilot study
274. [Update in current care guidelines: induced abortion].
275. Misoprostol-induced termination of secondtrimester pregnancy in women with a history of cesarean section: A retrospective analysis of 56 cases
276. Sonographic patterns of the endometrium in assessment of medical abortion outcomes
277. Effectiveness of early medical abortion using low-dose mifepristone and buccal misoprostol in women with no defined intrauterine gestational sac
278. Perinatal outcomes after organ preserving surgery in patient with ovarian cancer
279. Why is access to medical abortion limited in the Russian Federation?
280. Introducing medical abortion in Turkey: Perspectives of nurses/midwives
281. Survey of the attitude to, the knowledge and the practice of contraception and medical abortion in women who attended a family planning clinic
282. Medical abortion follow-up with serum human chorionic gonadotropin compared with ultrasonography: A randomized controlled trial
283. Unsuccessful prior attempts to terminate pregnancy among women seeking first trimester abortion at registered facilities in Bihar and Jharkhand, India.
284. Unsuccessful prior attempts to terminate pregnancy among women seeking first trimester abortion at registered facilities in Bihar and Jharkhand, India
285. Does congenital heart disease severely jeopardise family life and pregnancies? Obstetrical history of women with congenital heart disease in a single tertiary centre
286. Situation analysis of patients attending TU teaching hospital after medical abortion with problems and complications
287. First-trimester medical abortion with mifepristone 200 mg and misoprostol: A systematic review
288. A pilot study on the use of a 7-day course of letrozole followed by misoprostol for the termination of early pregnancy up to 63 days
289. Extending outpatient medical abortion services through 70 days of gestational age
290. Cervical pregnancy: A rare case of reimplantation after abortion. A case report
291. Sonohysterography: A simple tool for the diagnosis of pregnancy in the rudimentary horn: A case report
292. Effect of letrozole on uterine artery Doppler flow indices prior to first-trimester termination of pregnancy: a randomized controlled trial.
293. Menstrual regulation using medication is acceptable and feasible in Bangladesh
294. Errors in after medical abortion recovery period and their overcoming through the training of OB&Gyn
295. Evaluation of abortion services and patient characteristics in a mexico city public hospital after legalization
296. Induced abortion in the first trimester of pregnancy and repeat pregnancy in adolescents
297. Early medical abortion using low-dose mifepristone followed by buccal misoprostol: A large Australian observational study
298. Etonogestrel implant at the time of mifepristone for medical abortion
299. Clinical diagnosis of completeness of medical abortion by nurses: A reliability study in Mozambique
300. Failed early medical abortion: Beware of the uterine scar! Case report
301. Introducing medical abortion in Turkey: Perceptions of physicians
302. Some errors after the medical abortion recovery period and contraception choice
303. The preemptive effect of ibuprofen versus placebo on pain relief and success rates of medical abortion: A double-blind randomised controlled study
304. Restrictions in access to abortion: The Italian situation
305. Medical abortion with home administration of misoprostol up to 63 days gestation
306. Medical abortion at 9-12 weeks
307. Abortion in minors
308. Clinical use and safety of medical method of first trimester abortion
309. Efficacy and safety of mifepristone and buccal misoprostol versus buccal misoprostol alone for medical abortion
310. Comparison of Sublingual, oral and vaginal misopristol for cervical ripening 12 hours before Hysteroscopy
311. Adolescent girls undergoing medical abortion have lower risk of haemorrhage, incomplete evacuation or surgical evacuation than women above 18 years old
312. A randomized comparative study on vaginal administration of acetic acid-moistened versus dry misoprostol for mid-trimester pregnancy termination
313. Can women accurately assess the outcome of medical abortion based on symptoms alone?
314. Introducing medical abortion in Turkey: Perspectives of physicians
315. Women's decision making regarding choice of second trimester termination method for pregnancy complications
316. Pre-emptive effect of ibuprofen versus placebo on pain relief and success rates of medical abortion: A double-blind, randomized, controlled study
317. Clinical, surgical, and histopathologic outcomes following failed medical abortion
318. Comparison of misoprostol-only and combined mifepristone-misoprostol regimens for home-based early medical abortion in Tunisia and Vietnam
319. Early pregnancy termination with oral mifepristone and vaginal misoprostol
320. Effects of taking estrogen and progestogen after medical abortion on reducing vaginal hemorrhage time: a randomized-controlled trial
321. Using oral and intravaginal misoprostol for medical abortion
322. Efficacy of misoprostol administration 24 hours after mifepristone for termination of early pregnancy
323. Prospective study of medical abortion in Nepal Medical College Teaching Hospital (NMCTH). A one year experience.
324. Does follow-up with serum hcg simplify medical abortion? A randomized controlled trial
325. Has mifepristone medical abortion expanded abortion access in New Mexico? A survey of OB-GYN and Family Medicine physicians
326. Randomized trial of oral versus sublingual misoprostol 24 h after mifepristone for medical abortion
327. Methotrexate and misoprostol teratogenicity: Further expansion of the clinical manifestations
328. Simultaneous administration of mifepristone and misoprostol for early termination of pregnancy: A randomized controlled trial
329. Surgical intervention after medical abortion
330. Comparative effectiveness, safety and acceptability of medical abortion at home and in a clinic: A systematic review
331. Comparison of rates of adverse events in adolescent and adult women undergoing medical abortion: Population register based study
332. Evaluation of a network of medical abortion providers in two districts of Maharashtra, India
333. Mifepristone followed by home administration of buccal misoprostol for medical abortion up to 70 days of amenorrhoea in a general practice in Curaçao
334. Placenta accreta: A cause of failed medical abortion
335. Medical methods for mid-trimester termination of pregnancy.
336. The hemostatic system with medical abortion
337. Medical methods for mid-trimester termination of pregnancy
338. Hypoplastic left heart syndrome: 19 years of prenatal diagnosis
339. Can midlevel health-care providers administer early medical abortion as safely and effectively as doctors? A randomised controlled equivalence trial in Nepal
340. Effects of leonurine hydrochloride on medically induced incomplete abortion in early pregnancy rats
341. Successful term-delivery following medically managed 9 weeks live caesarean scar pregnancy
342. Issues in second trimester induced abortion (medical/surgical methods)
343. Preliminary results of the role of semiquantitative pregnancy tests in medical abortion provision
344. The extended gestational age medical abortion study: The effectiveness of medical abortion with mifepristone and misoprostol at 57-63 days versus 64-70 days gestation
345. Medical abortion at 63 to 90 days of gestation
346. Critical appraisal of contraceptive research
347. Reducing the buccal dose of misoprostol in mifepristone medical abortion up to 63 days LMP
348. Home self-administration of vaginal misoprostol for medical abortion at 50 to 63 days compared with gestation of below 50 days
349. Endometrial thickness as predictor of the effectiveness of intracervical misoprostol in early pregnancy failure
350. Accessibility, safety and outcomes in a United Kingdom National Health Service NHS abortion clinic (Walsall)
351. Medical abortion: Clinical aspects
352. Female rehabilitation after medical abortion
353. Efficacy of Single Dose of Mifepristone Combined with Two Doses of Misoprostol in Early Medical Abortions
354. Association of the G2014G genotype in estrogen receptor 1 gene with failure of the mifepristone-induced termination of early pregnancy
355. Home self-administration of vaginal misoprostol for medical abortion at 50-63 days compared with gestation of below 50 days
356. Misoprostol dose and route after mifepristone for early medical abortion: A randomised controlled noninferiority trial
357. A comparative study on sublingual versus oral and vaginal administration of misoprostol for late first and early second trimester abortion
358. Is mifepristone 100mg an effective alternative to standard dose for medical abortion
359. Timing and indication for curettage after medical abortion in early pregnant women with prior uterine incision
360. Increasing women's choices in medical abortion: A study of misoprostol 400 μg swallowed immediately or held sublingually following 200 mg mifepristone
361. Delivering on the promise - IPPF's global action to safe abortion services
362. Medical abortion - A life saving technology
363. Misoprostol for second trimester medical abortion - A comparison of three routes of administration
364. Utility of manual vaccum aspiration (MVA) in a Tunisian tertiary referral center
365. A randomized controlled study of sublingual vs. oral misoprostol (400 mg) following mifepristone (200 mg) for medical abortion up to 63 days L.M.P. in Tunisia
366. Two-pill regimens of misoprostol after mifepristone medical abortion through 63 days' LMP: A randomized controlled trial of buccal and sublingual misoprostol
367. Evaluation of ultrasound needs in first trimester medical abortion with misoprostol
368. Cost-effectiveness of alternative strategies for first-trimester abortion in Mexico and Nigeria
369. The experiences of women accessing legal abortion in Mexico City
370. Endometrial thickness following medical abortion is not predictive of subsequent surgical intervention
371. Medication abortion
372. Effects of mifepristone on fibronectin in human villus and deciduas
373. Exploring the costs and economic consequences of unsafe abortion in Mexico City before legalisation
374. Non-physician clinicians can safely provide first trimester medical abortion
375. Importance of a second dose of misoprostol in the management of medical abortion after failure of the initial treatment
376. Ibuprofen and paracetamol for pain relief during medical abortion: a double-blind randomized controlled study
377. Medical treatment with misoprostol for early failure of pregnancies after assisted reproductive technology: a promising treatment option
378. Setting up a medical abortion service for failure
379. Reply of the Authors: Setting up a medical abortion service for failure
380. Frequency and risk factors for repeat abortions after surgical compared with medical termination of pregnancy
381. High failure rates of medical termination of pregnancy after introduction to a large teaching hospital
382. Effect of previous live birth and prior route of delivery on the outcome of early medical abortion
383. Two mifepristone doses and two intervals of misoprostol administration for termination of early pregnancy: A randomised factorial controlled equivalence trial
384. Mifepristone plus vaginal misoprostol vs vaginal misoprostol alone for medical abortion in gestation 63 days or less in Nepalese women: A quasi-randomized controlled trial
385. Comparison of vaginal and sublingual misoprostol for second trimester abortion: Randomized controlled equivalence trial
386. Efficacy of combination therapy with methotrexate and misoprostol in termination of pregnancy in the first trimester
387. Early pregnancy termination with a simplified mifepristone: Medical abortion outpatient regimen
388. Medical abortion at home and at hospital: A trial of efficacy and acceptability
389. High-dose misoprostol as an alternative therapy after failed medical abortion
390. The role of parity in medical abortion up to 49 days of amenorrhoea
391. Age, parity, history of abortion and contraceptive choices affect the risk of repeat abortion
392. Misoprostol use as a method of medical abortion
393. Integrating medical abortion into safe abortion services: Experience from three pilot sites in South Africa
394. Complications after Second Trimester Surgical and Medical Abortion
395. Second Trimester Abortions in India
396. Second Trimester Medical Abortion with Mifepristone-Misoprostol and Misoprostol Alone: A Review of Methods and Management
397. A randomised comparative study on sublingual versus vaginal administration of misoprostol for termination of pregnancy between 13 to 20 weeks
398. Misoprostol and pregnancy: Risk of malformations
399. First trimester medical termination of pregnancy: The Nottingham experience
400. Survey of the attitude to, knowledge and practice of contraception and medical abortion in women attending a family planning clinic
401. A clinical evaluation of the safety and efficacy of mefepristone and misoprostol in medical abortions
402. Farmacologico abortion by means of mifepristone and misoprostol
403. Endometrial Thickness and Serum β-hCG as Predictors of the Effectiveness of Oral Misoprostol in Early Pregnancy Failure
404. Termination of early pregnancy using flexible, low-dose mifepristone-misoprostol regimens
405. Pathopharmacology of excessive hemorrhage in mifepristone abortions
406. RU 486 (Mifepristone) - "The abortive pill". Our experience
407. Management of pheochromocytoma during pregnancy: About three cases
408. Toxic shock associated with Clostridium sordellii and Clostridium perfringens after medical and spontaneous abortion
409. Clinical study of terminating biochemical pregnancy and early clinical pregnancy with mifepristone and misoprostol
410. Risk factors for unsuccessful medical abortion with mifepristone and misoprostol
411. Reproductive Choice for Women and Men Living with HIV: Contraception, Abortion and Fertility
412. Mifepristone and misoprostol administered simultaneously versus 24 hours apart for abortion: A randomized controlled trial
413. Randomised controlled trial comparing the efficacy of same-day administration of mifepristone and misoprostol for termination of pregnancy with the standard 36 to 48 hour protocol
414. Vaginal misoprostol for salvage therapy after failed medical abortion
415. Alternatives to mifepristone for early medical abortion
416. Does methotrexate confer a significant advantage over misoprostol alone for early medical abortion? A retrospective analysis of 8678 abortions
417. Clinical applications of mifepristone
418. Use of mifepristone and sublingual misoprostol for early medical abortion
419. Evidence for shortening the time interval of prostaglandin after mifepristone for medical abortion
420. Antiprogesterone for medical abortion
421. Monitoring medical abortion using mifepristone/misoprostol combination with ultrasonogram and serum human chorionic gonadotropin
422. Analysis of severe adverse events related to the use of mifepristone as an abortifacient
423. Ambulatory medical abortion performed in a family planning center
424. Consistency of medical abortion efficacy from 5 through 14 weeks' gestation
425. Development and evaluation of the abortion attributes questionnaire
426. Randomized trial of mifepristone and buccal or vaginal misoprostol for abortion through 56 days of last menstrual period
427. Medical abortion in rural Tamil Nadu, South India: A quiet transformation
428. Randomised controlled trial comparing efficacy of same day administration of mifepristone and misoprostol for termination of pregnancy with the standard 36- to 48-hour protocol.
429. Pathophysiology of mifepristone-induced septic shock due to Clostridium sordellii
430. Medical abortion using methotrexate and misoprostol. Efficacy and tolerability
431. Introduction of early medical abortion in New Zealand: An audit of the first 67 cases
432. A randomised controlled trial of mifepristone in combination with misoprostol administered sublingually or vaginally for medical abortion up to 13 weeks of gestation
433. Home self-administration of misoprostol for medical abortion up to 56 days' gestation
434. Mifepristone 100mg for early medical abortion
435. Medical abortion at 9-13 weeks' gestation: A review of 1076 consecutive cases
436. A pilot study of mifepristone and misoprostol administered at the same time for abortion up to 49 days gestation
437. Mifepristone 100mg for early medical abortion
438. Failed medical termination of twin pregnancy with mifepristone: A case report
439. Multicenter trial of a simplified mifepristone medical abortion regimen
440. Side effects of induced abortion: Results from a population-based survey
441. The efficacy and acceptability of mifepristone medical abortion with home administration misoprostol provided by private providers linked with the hospital: A prospective study of 433 patients
442. A randomized trial of mifepristone in combination with misoprostol administered sublingually or vaginally for medical abortion at 13-20 weeks gestation
443. The effect of non-steroidal anti-inflammatory drugs on medical abortion with mifepristone and misoprostol at 13-22 weeks gestation
444. Accounting for time: Insights from a life-table analysis of the efficacy of medical abortion
445. Acceptability of home-use of misoprostol in medical abortion
446. Results and lessons learned from a small medical abortion clinical study in Turkey
447. A fatal case of Clostridium sordellii septic shock syndrome associated with medical abortion.
448. Randomized, double-blind, controlled trial of mifepristone in capsule versus tablet form followed by misoprostol for early medical abortion
449. Misdiagnosis of ectopic pregnancy: Importance of ultrasonography prior to early termination of pregnancy
450. Misoprostol as the primary agent for medical abortion in a low-income urban setting
451. Methods for induced abortion
452. Role of routine ultrasonography in monitoring the outcome of medical abortion in a clinical setting
453. Prediction of late failure after medical abortion from serial β-hCG measurements and ultrasonography
454. Midtrimester medical termination of pregnancy: A review of 1002 consecutive cases
455. Increased risk for medical abortion failure for multiparous women
456. Current medical abortion care
457. Methotrexate/Misoprostol Embryopathy: Report of Four Cases Resulting From Failed Medical Abortion
458. Medical termination of pregnancy with mifepristone - Initial experience at the Sheba Medical Center
459. Medical abortion: Defining success and categorizing failures
460. WHO multinational study of three misoprostol regimens after mifepristone for early medical abortion. I: Efficacy
461. Successful medical abortion of a pregnancy within a noncommunicating rudimentary uterine horn
462. Medical termination of pregnancy in the second trimester
463. Large variation in clinical regimens used to induce medical abortion in Denmark
464. Medical abortion at 64 to 91 days of gestation: A review of 483 consecutive cases
465. A viable alternative to surgical vacuum aspiration: Repeated doses of intravaginal misoprostol over 9 hours for medical termination of pregnancies up to eight weeks
466. Medical abortion in family practice: A case series
467. Options for early therapeutic abortion: A comparative review
468. Factors affecting the outcome of early medical abortion: A review of 4132 consecutive cases
469. Randomized trial of oral versus vaginal misoprostol 2 days after mifepristone 200 mg for abortion up to 63 days of pregnancy
470. Mifepristone-misoprostol abortion: A trial in rural and urban Maharashtra, India
471. Medical abortion with mifepristone and misoprostol: A clinical trial in Taiwanese women
472. Could American women use mifepristone-misoprostol pills safely with less medical supervision?
473. Medical abortion in the first trimester
474. A prospective randomized, double-blinded, placebo-controlled trial comparing mifepristone and vaginal misoprostol to vaginal misoprostol alone for elective termination of early pregnancy
475. Use of misoprostol in gynecology and obstetrics
476. Medical abortion as an alternative to vacuum aspiration: First experiences with the 'abortion pill' in The Netherlands
477. Mifepristone abortion in minors
478. Early pregnancy termination with mifepristone and misoprostol in Norway
479. Randomized trial of oral versus vaginal misoprostol at one day after mifepristone for early medical abortion
480. Second trimester medical abortion with mifepristone and gemeprost: A review of 956 cases
481. Lowering the doses of mifepristone and gemeprost for early abortion: A randomised controlled trial
482. First trimester abortion with mifepristone and vaginal misoprostol
483. Medical abortion at 57 to 63 days' gestation with a lower dose of mifepristone and gemeprost. A randomized controlled trial
484. Double-blind randomized trial of mifepristone in combination with vaginal gemeprost or misoprostol for induction of abortion up to 63 days gestation
485. Parity is a major determinant of success rate in medical abortion: A retrospective analysis of 3161 consecutive cases of early medical abortion treated with reduced doses of mifepristone and vaginal gemeprost
486. Clinical study of four cases with malignant gestation trophoblastic tumor after mifepristone abortion
487. Vaginal misoprostol administered 1, 2, or 3 days after mifepristone for early medical abortion a randomized trial
488. Are US health professionals likely to prescribe mifepristone or methotrexate?
489. The counseling component of medical abortion.
490. Advanced practice clinicians and medical abortion: increasing access to care.
491. Misoprostol alone--a new method of medical abortion?
492. Medical abortion in China.
493. Abortion reporting in the era of medical procedures: why is it important?
494. Comparison of two doses of mifepristone in combination with misoprostol for early medical abortion: A randomised trial
495. The efficacy of medical abortion: A meta-analysis
496. Low-dose mifepristone followed by vaginal misoprostol at 48 hours for abortion up to 63 days
497. Vaginal misoprostol alone for medical abortion up to 9 weeks of gestation: Efficacy and acceptability
498. Effective, low-cost regimens for medical termination of pregnancy at all gestations
499. Methotrexate and misoprostol used alone or in combination for early abortion
500. Early medical abortion - First experiences
501. Methotrexate and misoprostol for early abortion in adolescent women
502. Tamoxifen compared to methotrexate when used with misoprostol for abortion
503. Providing mifepristone-misoprostol medical abortion: the view from the clinic.
504. The influence of abortion legislation on maternal mortality
505. Blood loss with mifepristone-misoprostol abortion: Measures from a trial in China, Cuba and India
506. Cutting unsafe abortions.
507. Acceptability and feasibility of early pregnancy termination by mifepristone-misoprostol. Results of a large multicenter trial in the United States. Mifepristone Clinical Trials Group.
508. Mifepristone (RU 486): current knowledge and future prospects.
509. Oral methotrexate and vaginal misoprostol for early abortion
510. Assessing strength of preference for abortion method using 'willingness to pay': a useful research technique for measuring values.
511. An effective regimen for early medical abortion: A report of 2000 consecutive cases
512. Abortion incidence and services in the united states, 1995-1996
513. Medical abortion with methotrexate 75 mg intramuscularly and vaginal misoprostol
514. Implications for gynecological services of new medical methods of therapeutic abortion
515. Abortion induced with methotrexate and misoprostol: A comparison of various protocols
516. Impact of the introduction of new medical methods on therapeutic abortions at the Royal Infirmary of Edinburgh
517. Oral misoprostol administration in the first and second trimester for termination
518. A phase III multicentre study on medical termination of early pregnancy with two regimens of mifepristone followed by PG05
519. Change in serum β-human chorionic gonadotropin after abortion with methotrexate and misoprostol
520. Medical abortion: what does the research tell us?
521. Medical abortion: What does the research tell us?
522. Acceptability of medical abortion in early pregnancy
523. Termination of early pregnancy by two regimens of mifepristone with misoprostol: a multicentre clinical trial
524. Termination of early pregnancy by two regimens of mifepristone with misoprostol and mifepristone with PG05 - A multicentre randomized clinical trial in China
525. Psychological responses following medical abortion (using mifepristone and gemeprost and surgical vacuum aspiration: A patient-centered, partially randomised prospective study
526. A prospective economic evaluation comparing medical abortion (using mifepristone and gemeprost) and surgical vacuum aspiration
527. Methotrexate and misoprostol vs misoprostol alone for early abortion: A randomized controlled trial
528. A comparison of surgical vacuum aspiration abortion with medical abortion using mifepristone (RU 486) and gemeprost: Implications for nursing staff
529. A comparison of medical abortion (using mifepristone and gemeprost) with surgical vacuum aspiration: Efficacy and early medical sequelae
530. The effect of dose of mifepristone and gestation on the efficacy of medical abortion with mifepristone and misoprostol
531. Comparison of medical abortion with surgical vacuum aspiration: Women's preferences and acceptability of treatment
532. Women's evaluation of three early abortion methods
533. Medical abortion in women of ≤56 days amenorrhoea: A comparison between gemeprost (a PGE(1) analogue) alone and mifepristone and gemeprost
534. Psychiatric morbidity and acceptibility following medical and surgical methods of induced abortion
535. Future direction of abortion technology
536. Experiences with the progesterone antagonist Mifepristone (RU 486) for termination of early pregnancy
537. The use of prostaglandins and antiprogestins for pregnancy termination
538. Anti-progesterones for the interruption of pregnancy
539. Medical responsibility in the practice of voluntary first trimester terminations of pregnancy. Preventive measures drawn from jurisprudence and from the study of 50 files of legal action or threatened legal action
540. Medical abortion complications. An epidemiologic study at a mid-Missouri clinic
541. Septic abortion. Personal experience and management

**1.3 Records identified through Scopus (n=887)**

1. Evaluating women’s acceptability of treatment of incomplete second trimester abortion using misoprostol provided by midwives compared with physicians: a mixed methods study
2. Telemedicine medical abortion at home under 12 weeks' gestation: A prospective observational cohort study during the COVID-19 pandemic
3. Integrative transcriptomics and proteomics analyses to reveal the therapeutic effect and mechanism of Buxue Yimu Pills in medical-induced incomplete abortion rats
4. Pattern of congenital heart disease among Egyptian children: a 3-year retrospective study
5. Comparison of obstetric emergency clinical readiness: A cross-sectional analysis of hospitals in Amhara, Ethiopia
6. Disruptive congenital anomalies associated with misoprostol. Case report; [Anomalías congénitas disruptivas asociadas con misoprostol. Reporte de un caso]
7. The value of posterior cervical angle as a predictor of vaginal delivery: A preliminary study
8. Factors Affecting the Success of Repeated Misoprostol Course for the Treatment of Missed Abortion
9. Impacts of Delivery Mode and Maternal Factors on Neonatal Oral Microbiota
10. Parsing the Q-Markers of Baoyin Jian to Treat Abnormal Uterine Bleeding by High-Throughput Chinmedomics Strategy
11. Abortion in International Human Rights Law: Missed Opportunities in Manuela v El Salvador
12. Analysis of pharmacodynamic components, targets and synergistic action mechanism of Fuyuan Shenghua granule for the treatment of medical-induced incomplete abortion based on network pharmacology
13. A Stalled Revolution? Misoprostol and the Pharmaceuticalization of Reproductive Health in Francophone Africa
14. Myometritis with pelvic septic vein thrombophlebitis secondary to Fusobacterium necrophorum sepsis
15. Misoprostol-Induced Fever and Unnecessary Antibiotic Prescribing: A Retrospective Study
16. Comparison of mifepristone plus misoprostol with misoprostol alone for first trimester medical abortion: A systematic review and meta-analysis
17. Buxue Yimu Pills improve angiogenesis and blood flow in experimental zebrafish and rat models
18. Telephone follow-up after early medical abortion using Australia's first low sensitivity urine pregnancy test
19. Efficacy, Safety, and Acceptability of Misoprostol in the Treatment of Incomplete Miscarriage: A Systematic Review and Meta-analysis; [Eficacia, seguranca e aceitabilidade do misoprostol no tratamento do aborto incompleto: Uma revisao sistem tica e metanalise]
20. The Development of Medical Law in the Field of Gynecology and the Relationship with Criminal Law: The Applicability of the Legal Framework of the Republic of Kosovo; [Rozwój prawa medycznego w dziedzinie ginekologii a jego związek z prawem karnym. Obowiązywanie ram prawnych Republiki Kosowa]
21. ABLE-BODIED WOMEN KILLING DISABLED BABIESHow Modern Narratives on Disability and Abortion Erase Disabled People From the Reproductive Justice Movement
22. Cervical vasovagal shock: A rare complication of incomplete abortion case report
23. GnRH‐a combined fertility‐sparing re‐treatment in women with endometrial carcinoma or atypical endomertial hyperplasia who failed to oral progestin therapy; [GnRH-a 联合治疗用于口服孕激素治疗失败的子宫内膜非典型增生及子宫内膜癌患者的探讨]
24. The portrayal and perceptions of cesarean section in Mexican media Facebook pages: a mixed-methods study
25. Management of pregnancy and delivery in women with transposition of the great arteries after atrial switch operation: A 16-year single-center experience
26. Epidemiology of spontaneous pregnancy loss in Kazakhstan: A national population-based cohort analysis during 2014–2019 using the national electronic healthcare system
27. Effects of laser-assisted hatching on the clinical outcome of freeze-thawedcleavage embryo transfer in patients with repeated implantation failure; [激光辅助孵化对反复种植失败患者行冻融卵裂期胚胎移植临床结局的影]
28. PXR mediates mifepristone-induced hepatomegaly in mice
29. Complications of second trimester induction for abortion or fetal demise for patients with and without prior cesarean delivery
30. Effect of folic acid supplementation on diminished ovarian reserve: study protocol of a single-centre, open-label, randomised, placebo-controlled clinical trial
31. "I will never wish this pain to even my worst enemy": Lived experiences of pain associated with manual vacuum aspiration during postabortion care in Kenya
32. A novel method for office aspiration curettage in cases of retained products of conception: A randomized controlled trial
33. Experiences of midwives and nurses when implementing abortion policies: A systematic integrative review
34. “Nothing More or Less than a Discharged Convict”: The Career of Dr Thomas Millerchip of Coventry, 1874–1912.
35. Abortion policy implementation in Ireland: Lessons from the community model of care
36. Factors Associated with Cesarean Operations of Gestational Diabetic Mellitus and Diabetes Complications
37. The relationship between insecurity and the quality of hospital care provided to women with abortion-related complications in the Democratic Republic of Congo: A cross-sectional analysis
38. Role of isosorbide mononitrate as an agent for cervical ripening in second-trimester abortions
39. Influence of Two Endometrial Preparation Programs Independent of Endogenous Ovarian Cycle on the Pregnancy Outcome of Thin Endometrial Patients with Freeze-thaw Embryo Transfer; [两种非依赖内源性卵巢周期的内膜准备方案对薄型子宫内膜冻融胚胎移植患者妊娠结局的影响研究]
40. The association between history of retained placenta and success rate of misoprostol treatment for early pregnancy failure
41. Status epilepticus in pregnancy: a literature review and a protocol proposal
42. The effect of hysteroscopic metroplasty on fertility in T-shaped and Y-shaped dysmorphic uterus
43. Medical Management of Induced and Incomplete First-Trimester Abortion by Non-physicians in Low- And Middle-Income Countries: A Systematic Review and Meta-analysis of Randomized Controlled Trials
44. Impact of prolonged use of adjuvant tocolytics after cervical cerclage on late abortion and premature delivery
45. Effectiveness of Jian-Pi-An-Tai formula for the pregnancy outcome of in vitro fertilization and embryo transfer in infertile women: Protocol of a randomized controlled trial
46. Conservative management of uterine necrosis following bilateral uterine vessels ligation and B-Lynch suture: Long term follow up
47. The efficacy of Shenghua Decoction supplementation after early medical abortion: A meta-analysis of randomized controlled trials
48. Contraception Utilization in Black Women via a Reproductive Justice Lens
49. Predictive value of peripheral blood α1-acid glycoprotein in medical abortion outcomes with mifepristone and relativity of concentration
50. Analysis of clinical characteristics and prognosis of 21 pregnant women complicated with tuberculosis after in vitro fertilization-embryo transfer; [21例体外受精-胚胎移植后妊娠并发结核病患者的 临床特征及预后分析]
51. Significance of clinical and anamnestic risk factors for the prediction of negative outcomes in assisted reproductive technology programs
52. Prescription for Profit: How Doctors Defraud Medicaid
53. Structure of reproductive losses of adolescent girls and women of reproductive age in the Tyumen
54. Comparing pregnancy outcomes between symptomatic and asymptomatic COVID-19 positive unvaccinated women: Multicenter study in Saudi Arabia
55. Occurrence and Determinants of Psychological Distress among Women Undergoing Abortion/Medical Termination of Pregnancy
56. Design of a syringe extension device (Chloe SED®) for low-resource settings in sub-Saharan Africa: a circular economy approach
57. The legal implications of prenatal diagnosis in Malaysia
58. Factors Associated with the Outcomes of Preimplantation Genetic Testing in Assisted Reproduction; [胚胎种植前遗传学检测助孕妊娠结局的影响因素研究]
59. An examination of loss to follow-up and potential bias in outcome ascertainment in a study of direct-to-patient telemedicine abortion in the United States
60. Assessing survival time of outpatients with cervical cancer: at the university of Gondar referral hospital using the Bayesian approach
61. Progesterone-mediated reversal of mifepristone-induced pregnancy termination in a rat model: an exploratory investigation
62. Advances of Medical termination of pregnancy Amendments act in India.
63. Postpartum Contraception in China: Status, Problems and Coping Strategies; [我国产后避孕现状和面临的问题及应对策略]
64. Assessing an ovarian reserve and risk factors for premature ovarian failure as part of pre-abortion counseling for women under 40 planning to terminate own first pregnancy; [Исследование овариального резерва и факторов риска преждевременной недостаточности яичников в рамках доабортного консультирования женщин в возрасте до 40 лет, планирующих прервать первую беременность]
65. Interpretation to Medication Abortion Up to 70 Days of Gestation •. ACOG Practice Bulletin , Number 225; [2020 AGOG实践简报：孕龄<70 d药物 流产(No. 225)》解读]
66. Safe Abortions: Are They a Reality Yet? A Case Series
67. Trend of serum beta-human chorionic gonadotropin levels after medical abortion in the early first trimester of pregnancy
68. Economic cost of pregnancy care attributable to the failure of Mexico's teenage pregnancy prevention policy; [Costo económico de atención de embarazos atribuibles a la falla de la política de prevención del embarazo adolescente en México]
69. Evaluation of different doses of Femoston therapy for incomplete abortion: A prospective observational trial
70. Self-administration of Abortion Pills and its Maternal Outcome in Tertiary Care Center
71. Return of Fertility after Discontinuation of Contraception According Type of Contraception, Duration of Use, Age and Body Mass Index; [Kembalinya Kesuburan setelah Penghentian Alat Kontrasepsi Berdasarkan Jenis Kontrasepsi, Lama Pemakaian, Usia dan Indeks Massa Tubuh]
72. Reproductive health crisis during waves one and two of the COVID-19 pandemic in India: Incidence and deaths from severe maternal complications in more than 202,000 hospital births
73. Risk factors for intrauterine device embedment in postmenopausal women: An analysis of 731 participants undergoing hysteroscopy
74. Pharmacy dispensing of abortion pills in Ghana: Experiences of pharmacy workers and users
75. Acupuncture benefits to women with recurrent implantation failure: A propensity score-matched cohort study
76. Primary health care, access to legal abortion and the notion of ideal victim among medical practitioners: The case of Chile
77. Fatal necrotizing fasciitis in illegal abortion and the negligence tort
78. Characteristics of maternal cardiac disease and pregnancy outcomes: results from a 4-year observational cohort survey in Central Vietnam
79. Factors associated with the failure of medical treatment for ectopic pregnancy: case study conducted at the Yaoundé Gynaecology, Obstetrics and Pediatrics Hospital; [Facteurs associés à l’échec du traitement médical de la grossesse extra-utérine: cas de l’Hôpital Gyneco-Obstétrique et Pédiatrique de Yaoundé]
80. Pharmacological voluntary interruption of pregnancy in a rural area; [Seguridad en la interrupción voluntaria del embarazo farmacológica en un entorno rural]
81. Subsequent placenta accreta after previous mifepristone-induced abortion: A case report
82. Construction and validation of a spatial database of providers of transgender hormone therapy in the US
83. Outcome of First Trimester Induced Abortions Using Misoprostol by Buccal and Vaginal Routes
84. Accuracy of anemia screening by point-of-care hemoglobin testing in patients seeking abortion
85. Thrombophilia, Anticoagulant Therapy, and Pregnancy Outcome in Women with Poor Obstetric History
86. Misoprostol versus Suction Evacuation in the Management of Patients with Incomplete Abortion: A Scoping Review
87. Accessing abortion in a highly restrictive legal regime: Characteristics of women and pregnant people in Malta self-managing their abortion through online telemedicine
88. Management of incomplete and missed spontaneous abortions: a cohort study of trends in Calgary emergency departments
89. Cervical twin heterotopic pregnancy: Overview of ectopic pregnancies and scanning detection algorithm
90. Pregnancy in Pateints With Exstrophy-Epispadias Complex: Are Higher Rates of Complications and Spontaneous Abortion Inevitable?
91. Reproductive Justice Discourse vis-à-vis Abortion Law in India: A Critical Review
92. Vaginal bleeding and pelvic pain after medical abortion: A case of incomplete abortion diagnosed on POCUS transvaginal ultrasound
93. Perinatal prophylaxis with immunoglobulin anti-D and the impact on RhD sensitizations among pregnant women in Slovenia; [Perinatalna zaščita z imunoglobulinom anti-D in vpliv senzibilizacije na antigen RhD med nosečnicami v Sloveniji]
94. Effects of growth hormone in down-regulating hormone replacement cycle on the outcome of frozen-thawed embryo transfer; [降调节后激素替代方案中应用生长激素 对冻融胚胎移植周期结局的影响]
95. Management of the uterine abnormalities on the reproductive outcomes in women with repeated implantation failure; [宫腔异常治疗对反复种植失败患者助孕结局的影响]
96. Practice of unsafe abortion in Pakistan: Characteristics and outcome
97. Direct Effects of Mifepristone on Mice Embryogenesis: An In Vitro Evaluation by Single-Embryo RNA Sequencing Analysis
98. Evaluation of the success of medical abortion by a plasma hCG control threshold; [Évaluation du succès de l'interruption volontaire de grossesse médicamenteuse par un seuil d'hCG sérique]
99. Effectiveness of Self-Managed Medication Abortion between 9 and 16 Weeks of Gestation
100. Clinical indication of medical ultrasound as a diagnostic tool among the outdoor patients of a selected hospital in Bangladesh
101. Cannulation pitfalls in ECMO
102. Self-assessment of medical abortion outcome using symptoms and home pregnancy testing
103. Strategies for nursing uterine rupture in primiparas during pregnancy
104. Effectiveness and safety of early medication abortion provided in pharmacies by auxiliary nurse-midwives: A non-inferiority study in Nepal
105. Cardiovascular disease and associated comorbid conditions as determinants of adverse perinatal outcomes in pregnancy – An analysis of the results of the register of pregnant BEREG
106. Low-sensitivity urine pregnancy testing to assess medical abortion outcome: A systematic review
107. Understanding of changes in abortion rate following liberalization of abortion law among Brazilian residents in obstetrics and gynecology
108. Evaluation of effect of letrozole prior to misoprostol in comparison with misoprostol alone in success rate of induced abortion
109. ‘A Hope Raised and then Defeated’? the Continuing Harms of Irish Abortion Law
110. Endometrial polyp filled with gestational tissues remained undiscovered in an infertile woman for years: A case report
111. Factors Associated with Management Outcome of Incomplete Abortion in Yirgalem General Hospital, Sidama Zone, Southern Ethiopia
112. Postnatal cardiac outcomes associated with the prenatal diagnosis of absence of ductus venosus; [Evolución cardiológica postnatal y factores asociados a la agenesia de ductus venoso de diagnóstico prenatal]
113. Simultaneous Compared With Interval Medical Abortion Regimens Where Home Use Is Restricted: Correction
114. Late-Term Abortion and Medical Necessity: A Failure of Science
115. Maternal Fontan procedure is a predictor of a small-for-gestational-age neonate: a 10-year retrospective study
116. A maternal death from self-induced medical abortion: a call for action
117. The Language of First-Trimester Nonviable Pregnancy: Patient-Reported Preferences and Clarity
118. Uterine rupture during pregnancy and delivery: risk factors, symptoms and maternal and neonatal outcomes - restrospective cohort; [Ruptura d&#283;lohy v t&#283;hotenství a p&#345;i porodu: rizikové faktory, p&#345;íznaky a perinatální výsledky - retrospektivní analýza]
119. Misoprostol-induced acute coronary syndrome in a premenopausal woman: A case report with literature review
120. A comparison of pregnancy outcomes after second-trimester amniocentesis between cases with penetration of the placenta and nonpenetration
121. Effect of Pregnancy in Arrhythmogenic Right Ventricular Cardiomyopathy
122. Efficacy of medical abortion prior to 6 gestational weeks: a systematic review
123. Retraction notice (Journal of Vascular Surgery (2020) 72(2) (667–671), (S074152141932587X), (10.1016/j.jvs.2019.10.069))
124. Cordarone in obstetrics: may or not?
125. Rheumatoid arthritis and pregnancy. A case report and literature review; [Artritis reumatoide y embarazo: Reporte de un caso y revisión de la bibliografía]
126. Maternal and Perinatal Outcome of Maternal Obesity at RSCM in 2014-2019
127. Conflicting recommendations between the French national authority for health (HAS) and clinical practice guidelines (CNGOF); focus on 200 late medical abortions, conducted outside marketing authorizations; [Recommandations discordantes entre HAS et CNGOF; le point avec 200 IVG médicamenteuses tardives, réalisées hors AMM]
128. Post-abortion care: Ethical and legal duties
129. Targeted client communication via mobile devices for improving sexual and reproductive health
130. Leptospirosis seropositivity and its serovars among cattle in Northeastern Malaysia
131. Determination of Pregnant women Knowledge toward Risk Factors of Vitamin D Deficiency and Measuring Level during Pregnancy in Al- Nasiriyah City
132. Management of pain associated with up-to-9-weeks medical termination of pregnancy (MToP) using mifepristone–misoprostol regimens: expert consensus based on a systematic literature review
133. Clinical research of sequential embryo transfer in frozen thawed cycles of patients with recurrent implantation failure; [解冻周期序贯移植治疗反复种植失败的临床研究]
134. Filum terminale needle placement during caudal epidural steroid injection
135. New evidence on induced abortion in Tehran, Iran: Rates, causes, and changes
136. The use of manual vacuum aspiration in the treatment of incomplete abortions: A descriptive study from three public hospitals in Malawi
137. Diagnosis and clinical criteria for chronic endometritis; [Диагностика и клинические критерии хронического эндометрита]
138. Hysteroscopic Diagnosis of Omentum Incarceration Subsequent to an Iatrogenic Uterine Perforation
139. 2017–19 governmental decisions to allow home use of misoprostol for early medical abortion in the UK
140. Socio-cultural and obstetric risk factors associated with incomplete abortion in peruvian women; [Factores de riesgo socioculturales y obstétricos asociados al aborto incompleto en mujeres peruanas]
141. Experience of clandestine use of medical abortion among university students in Chile: a qualitative study
142. Corrections: Simultaneous Compared With Interval Medical Abortion Regimens Where Home Use Is Restricted (Obstetrics and Gynecology (2018) 131 (635-41) DOI: 10.1097/AOG.0000000000002536)
143. Effectiveness of the FIGO protocol for medical management of first-trimester abortion
144. Early surface ablation on aborted LASIK flaps
145. Medical management of induced and incomplete first-trimester abortion by non-physicians in low- and middle-income countries: A systematic review and meta-analysis of randomized controlled trials
146. Determinants and Outcome of Safe Second Trimester Medical Abortion at Jimma University Medical Center, Southwest Ethiopia
147. Perampanel and pregnancy
148. Avoiding Abortion Stigma: How the “Invisibilization Labour” Reinforces the Procreative Labour; [Esquiver le stigmate lié à l’avortement: le « travail d’invisibilisation» comme renforcement du travail procréatif]
149. The legal implications of prenatal diagnosis in Malaysia
150. Integrating gender perspectives in gynecology and obstetrics: Engaging medical colleges in Maharashtra, India
151. Success rate of methotrexate treatment for recurrent vs. primary ectopic pregnancy: a case-control study
152. Clinical and morphological differences in non-developing pregnancy and spontaneous abortion in early pregnancy
153. Two prophylactic pain management regimens for medical abortion ≤63 days' gestation with mifepristone and misoprostol: A multicenter, randomized, placebo-controlled trial
154. Does sublingual misoprostol reduce pain and facilitate IUD insertion in women with no previous vaginal delivery? A randomized controlled trial
155. Pregnancy rates and outcomes of HIV-infected women in Korea
156. Coronary artery vasospasm after misoprostol treatment for incomplete abortion: a case report
157. “I did not plan to have a baby. This is the outcome of our work”: a qualitative study exploring unintended pregnancy among female sex workers
158. Pregnancy in Wilson's disease: Management and outcome
159. Identifying national availability of abortion care and distance from major US cities: Systematic online search
160. Provision of medical abortion by midlevel healthcare providers in Kyrgyzstan: testing an intervention to expand safe abortion services to underserved rural and periurban areas
161. Causes of maternal death in Ethiopia between 1990 and 2016: Systematic review with meta-analysis
162. Follow-up Rates and Contraceptive Choices after Medical Abortion in Adolescents at Cook County Hospital
163. A STATISTICAL ANALYSIS OF WOMEN'S REPRODUCTIVE HEALTH CHARACTERISTICS AFTER INEFFECTIVE REATTEMPTS OF USING ART
164. Protective Effect of Taohong Siwu Decoction on Abnormal Uterine Bleeding Induced by Incomplete Medical Abortion in Rats during Early Pregnancy
165. Early abortion with buccal versus sublingual misoprostol alone: a multicenter, randomized trial
166. Protective effect of breastfeeding against childhood leukemia in Zhejiang Province, P. R. China: a retrospective case-control study
167. Acute renal failure during the pregnancy: A review on pathophysiology, risk factors and management
168. Maternal and fetal prognosis of subsequent pregnancy in black African women with peripartum cardiomyopathy
169. Retrospective analysis of prognostic value of the neutrophil-to-lymphocyte ratio in early miscarriages: A 8-year survey
170. Pregnancy toxemia and lipid mobilization syndrome in two alpaca (Vicugna pacos) at 6 and 10 months of gestation
171. Crisis Pregnancy Centers in the United States: Lack of Adherence to Medical and Ethical Practice Standards; A Joint Position Statement of the Society for Adolescent Health and Medicine and the North American Society for Pediatric and Adolescent Gynecology
172. Association between uterine position and transvaginal misoprostol treatment for early pregnancy failure
173. Comparison of two low-sensitivity urine pregnancy tests for confirming the success of early medical abortion
174. Chemical Fingerprint Analysis and Ultra-Performance Liquid Chromatography Quadrupole Time-of-Flight Mass Spectrometry-Based Metabolomics Study of the Protective Effect of Buxue Yimu Granule in Medical-Induced Incomplete Abortion Rats
175. Acquired uterine arteriovenous malformation: Management and treatment
176. Risk factors and the choice of long-acting reversible contraception following medical abortion: effect on subsequent induced abortion and unwanted pregnancy
177. Structural equation modelling analysis determining causal role among methyltransferases, methylation, and apoptosis during human pregnancy and abortion
178. Practices and knowledge of female gynecologists regarding contraceptive use: A real-world Chinese survey
179. Estimation of the adolescent pregnancy rate in Thailand 2008-2013: An application of capture-recapture method
180. Medical referral for abortion and freedom of conscience in Australian law
181. Sublingual v/s vaginal misoprostol for second trimester termination of pregnancy: A comparative study
182. Cost-effectiveness of emergency contraception options over 1 year
183. The legal status of the fetus as a patient in Europe
184. Matsubara–Yano suture: a simple uterine compression suture for postpartum hemorrhage during cesarean section
185. Delayed hemolysis, elevated liver enzymes, low platelet count syndrome in succession of switches of preventive anticoagulant treatment in a 41-year-old patient with a history of recurrent assisted implantation failures: A case report
186. Application effect of different ovarian stimulation protocols in elderly patients with diminished ovarian reserve; [不同促排卵方案在高龄卵巢储备功能减退患者中的应用效果比较]
187. Outcomes During Early Implementation of Mifepristone-Buccal Misoprostol Abortions up to 63 Days of Gestation in a Canadian Clinical Setting
188. Protecting safe abortion in humanitarian settings: overcoming legal and policy barriers
189. Clinical outcomes of luteal phase stimulation for in vitro fertilization/intracytoplasmic sperm injection treatment in poor ovarian responders; [卵巢低反应患者黄体期促排卵行体外受精/卵胞质内单精子显微注射助孕临床结局分析]
190. Recurrent pelvic abscess: An unusual presentation of an undetected chronic ectopic pregnancy: A case report
191. Crimes related to illegally carrying out an artificial termination of pregnancy (abortion) in the legislation of foreign countries; [Преступления, связанные с незаконным проведением искусственного прерывания беременности (аборта), в законодательстве зарубежных стран]
192. Report of two cases of cervical pregnancy treated in a divergent way.; [Embarazo cervical tratado de forma divergente. Reporte de dos casos]
193. Abortion in Brazil: What do the official data say?; [Aborto no Brasil: O que dizem os dados oficiais?]; [Aborto en Brasil: ¿qué dicen los datos oficiales?]
194. Second-trimester medication abortion outside the clinic setting: An analysis of electronic client records from a safe abortion hotline in Indonesia
195. Opinion and use of contraceptives among medical students of the University of Nigeria, Enugu campus
196. Abortion education in Canadian family medicine residency programs
197. The Ethical Legacy of Hippocrates
198. Systematic Review of the Effectiveness, Safety, and Acceptability of Mifepristone and Misoprostol for Medical Abortion in Low- and Middle-Income Countries
199. Predicting poor compliance with follow-up and intrauterine contraception services after medical termination of pregnancy
200. Crisis Pregnancy Centers in the U.S.: Lack of Adherence to Medical and Ethical Practice Standards: A Joint Position Statement of the Society for Adolescent Health and Medicine and the North American Society for Pediatric and Adolescent Gynecology
201. Implication of sperm chromosomal abnormalities in recurrent abortion and multiple implantation failure
202. Male experiences of unintended pregnancy: Characteristics and prevalence
203. Validation of ICD-9 codes for stable miscarriage in the emergency department
204. Termination of pregnancy at very early gestation without visible yolk sac on ultrasound
205. Epidemiology
206. Comprehensive abortion care: Evidence of improvements in hospital-level indicators in Tigray, Ethiopia
207. Effect of Immediate Compared With Delayed Insertion of Etonogestrel Implants on Medical Abortion Efficacy and Repeat Pregnancy
208. The morphology, typical combinations of polymorphic genes of hemostasis, and specific features of the pathogenesis of retrochorial hematoma in missed abortion
209. Clinical analysis of ectopic pregnancies in a tertiary care centre in Southern India: A six-year retrospective study
210. Efficacy and safety of uterine tamponade to control bleeding and reduce obstetric hysterectomy. Historic cohort in Nuevo León, México, 2013; [Eficacia y seguridad del taponamiento uterino para control de hemorragia y disminución de histerectomía obstétrica. cohorte histórica en Nuevo León, México, 2013]
211. Bleeding after dilatation and curettage: The efficacy of transcatheter uterine artery embolisation
212. Experiences and prophylactic of panic attacks. The case of magnetic resonance imaging; [Doświadczanie i proflaktyka ataku paniki. Casus obrazowania rezonansem magnetycznym]
213. Curettage and asherman's syndrome-lessons to (Re-) learn?
214. Maternal and fetal outcomes with aortic dissection in pregnant patients with Marfan syndrome
215. Intrauterine administration of hCG immediately after oocyte retrieval and the outcome of ICSI: A randomized controlled trial
216. Constraints and optimization of the laser microwelding process of thin metal foils
217. Should organs from patients in permanent vegetative state be used for transplantation?
218. The case studies
219. Comparison of clinical outcomes of vitrified-thawed embryo transfer and fresh embryos transfer
220. The Outcome of Septic Abortion: A Tertiary Care Hospital Experience
221. Complications during pregnancy and delivery in women with untreated rectovaginal deep infiltrating endometriosis
222. Placenta previa; prevalence, risk factor and outcome
223. Pre-implantation genetic screening among women experiencing recurrent failure of in vitro fertilization
224. Infectious agents identified in aborted swine fetuses in a high-density breeding area: a three-year study
225. Integrating mobile phones into medical abortion provision: Intervention development, use, and lessons learned from a randomized controlled trial
226. Systematic review: The maternal mortality myth in the context of legalized abortion
227. Fertility, gestational and systemic problems in turner's syndrome: Two case reports; [Turner Sendromunda Fertilite, Gestasyonel ve Sistemik Sorunlar: Iki Olgu Sunumu]
228. Comparison of Outcomes before and after Ohio's Law Mandating Use of the FDA-Approved Protocol for Medication Abortion: A Retrospective Cohort Study
229. Life-threatening complications of MTP/abortion
230. Social questions, medical answers: Contesting British abortion law
231. Infertility today: The management of female medical causes
232. A successful twin pregnancy in a patient with HbE-β-thalassemia in western India
233. Effect of Wujia Shenghua capsule on uterine bleeding following medically-induced incomplete abortion in rats during early pregnancy
234. Decision making on unsafe abortions in Sri Lanka: A case-control study
235. Control of tropical theileriosis (Theileria annulata infection in cattle) in North Africa
236. ACR appropriateness criteria® first trimester bleeding
237. Is sex-selective abortion against the law?
238. Is a cleft lip and palate a serious "handicap"? Jepson v Chief Constable of West Mercia--a legal and ethical critique
239. Abortion practices in high school students in Yamoussoukro, Côte d’Ivoire; [Pratiques d’avortement chez des lycéennes à Yamoussoukro, Côte d’Ivoire]
240. Comparative Study of Mifepristone with Vaginal Misoprostol for First Trimester Termination of Pregnancy at Different Gestational Ages
241. The Culture of Illegal Abortion in South Africa
242. India's missing daughters: An ominous sign for democracy
243. Replacement of dilation and curettage/evacuation by manual vacuum aspiration and medical abortion, and the introduction of postabortion contraception in Pakistan
244. The Feasibility of a Modified Method of Laparoscopic Transabdominal Cervicoisthmic Cerclage during Pregnancy
245. Effectiveness of early medical abortion using low-dose mifepristone and buccal misoprostol in women with no defined intrauterine gestational sac
246. Ten years on: A review of medical terminations of pregnancy performed in a sexual health clinic
247. Kisspeptin: A potential factor for unexplained infertility and impaired embryo implantation
248. Complicated illegal induced abortions at a tertiary health institution in Nigeria
249. Ovarian function in systemic lupus erythematosus patients undergoing the use of cyclophosphamide in two major rheumatologic care centers in Curitiba, Paraná State; [Função ovariana em mulheres lúpicas submetidas ao uso de ciclofosfamida em dois grandes centros de atendimento reumatológico de Curitiba, Estado do Paraná]
250. Serum biomarkers may help predict successful misoprostol management of early pregnancy failure
251. Achievements of the FIGO Initiative for the Prevention of Unsafe Abortion and its Consequences in South-Southeast Asia
252. Seroprevalence of babesia caballi and theileria equi in horses in central Germany; [Vorkommen von Antikörpern gegen Babesia caballi und Theileria equi bei Pferden in Mitteldeutschland]
253. Hydatidiform moles among patients with incomplete abortion in Mwanza city, north western Tanzania
254. Diagnostic value of prenatal MR imaging in the detection of brain malformations in fetuses before the 26th week of gestational age
255. Exposure to radiation therapy is associated with female reproductive health among childhood cancer survivors: a meta-analysis study
256. Assessing post-abortion care in health facilities in Afghanistan: A cross-sectional study
257. A randomized trial of hospital vs home self administration of vaginal misoprostol for medical abortion
258. Factors related to completeness of medical abortion with mifepristone and misoprostol
259. Commentary: Access to medical-assisted reproduction and PGD in Italian law: A deadly blow to an illiberal statute? Commentary to the European court on human rights's decision costa and pavan v Italy
260. Comparison of the effects of voluntary termination of pregnancy and uterine evacuation for medical reasons on female sexual function
261. Elevated mRNA expression of PGF2α receptor splice variant 2(FP-V2) in human decidua is associated with incomplete mifepristone–misoprostol-induced early medical abortion by regulation of interleukin-8
262. Assessment of completion of early medical abortion using a text questionnaire on mobile phones compared to a self-administered paper questionnaire among women attending four clinics, Cape Town, South Africa
263. A Study of Incomplete Abortion Following Medical Method of Abortion (MMA)
264. Investigation of hepatitis B virus seroprevalence in hepatitis C infected patients; [Hepatit C virüsü ile enfekte hastalarda Hepatit B seroprevalansinin araştirilmasi]
265. Secondary infertility and the aging male, overview
266. Clinical analyses of 66 cases of mid-trimester pregnancy termination in women with prior cesarean
267. Advanced Abdominal Pregnancy As a Cause of Symptomatic Hemoperitoneum
268. Use of platelet rich plasma in human infertility
269. Dengue fever during pregnancy: Maternal and fetal complications
270. Enduring politics: the culture of obstacles in legislating for assisted reproduction technologies in Ireland
271. Triangular assessment of the etiology of induced abortion in Iran: A qtualitative study
272. A few concerns about bioethics; [Quelques préoccupations au sujet de la bioéthique]
273. Social and medical implications of teenage motherhood
274. Uncommon toxicity of low-dose methotrexate: Case report
275. Cervical ectopic pregnancy
276. Safety of induced abortions at less than 12 weeks of pregnancy in Japan
277. Healthcare students' knowledge and opinions about the Argentinean abortion law
278. Medical treatment of spontaneous abortion in the first trimester; [Tratamiento médico del aborto espontáneo del primer trimestre]
279. Administration of depot medroxyprogesterone acetate on the day of mifepristone for medical abortion: A pilot study
280. Minimal effective dose of mifepristone for medical abortion
281. Causes of acute renal failure in Nishtar Hospital Multan
282. Investigating the reasons of preterm labor among visitors of Shariati hospital in Bandar Abbas during 2012 and 2013
283. CAUSATIVE FACTORS OF PREMATURE BIRTH (A NEW LOOK AT THE PROBLEM); [ПРИЧИННЫЕ ФАКТОРЫ ПРЕЖДЕВРЕМЕННЫХ РОДОВ (НОВЫЙ ВЗГЛЯД НА ПРОБЛЕМУ)]; [ПРИЧИННІ ФАКТОРИ ПЕРЕДЧАСНИХ ПОЛОГІВ (НОВИЙ ПОГЛЯД НА ПРОБЛЕМУ)]
284. The role of auxiliary nurse-midwives and community health volunteers in expanding access to medical abortion in rural Nepal
285. Factors influencing the abortion interval of second trimester pregnancy termination using misoprostol
286. Invasive management of proximal ureteral calculi during pregnancy
287. Doppler velocimetry of the uterine arteries: an early screening test for miscarriage
288. Early versus late misoprostol administration after mifepristone for medical abortion
289. Successful treatment of placenta percreta through a combinatorial treatment involving a bakri balloon and methotrexate - a case report
290. Ectopic pregnancy: A life-threatening gynecological emergency
291. Estimating the costs for the treatment of abortion complications in two public referral hospitals: a cross-sectional study in Ouagadougou, Burkina Faso
292. Medical students and controversial ethical issues: Results from the multicenter study SBRAME
293. Is self-assessment of medical abortion using a low-sensitivity pregnancy test combined with a checklist and phone text messages feasible in South African primary healthcare settings? A randomized trial
294. Abortion, informed consent, and regulatory spillover
295. Ectopic pregnancy in the era of medical abortion: Are we ready for it? Spectrum of sonographic findings and our experience in a tertiary care service hospital of India
296. Placental abnormalities in equine pregnancies generated by SCNT from one donor horse
297. Pericentric inversion of human chromosome 9 epidemiology study in Czech males and females
298. Prophylactic compared with therapeutic ibuprofen analgesia in first-trimester medical abortion: A randomized controlled trial
299. "Right tool," wrong "job": Manual vacuum aspiration, post-abortion care and transnational population politics in Senegal
300. Pregnancy outcomes in multiple sclerosis patients previously treated with cyclophosphamide
301. Provision of abortion by mid-level providers: International policy, practice and perspectives
302. Compare of misoprostol and dinoprost effectivity by induced second-trimester abortion; [Srovnání efektivity misoprostolu a dinoprostu při indukci druhotrimestrálního abortu]
303. Sonohysterography: A simple tool for the diagnosis of pregnancy in the rudimentary horn: A case report
304. Human assisted reproduction
305. First-trimester medical abortion with mifepristone 200 mg and misoprostol: A systematic review
306. Second- and third-trimester termination of pregnancy in women with uterine scar - A retrospective analysis of 111 gemeprost-induced terminations of pregnancy after previous cesarean delivery
307. Your conscience, your right: A history of efforts to violate pro-life medical conscience, and the laws that stand in the way
308. The role of parity in medical abortion up to 49 days of amenorrhoea
309. Reasons due to which, women resort to illegally induced abortions
310. Methotrexate and misoprostol teratogenicity: Further expansion of the clinical manifestations
311. First-trimester pregnancy failure
312. "Reproductive health care," the "demographic imperative," and the real health needs of women in the developing world (part one)
313. Effects of mifepristone on fibronectin in human villus and deciduas
314. The effect of smoking on reproductive failures in couples examined in the genetic outpatient clinic at dr. A. Jurasz university hospital in bydgoszcz; [Rozpowszechnienie palenia tytoniu wśród par z niepowodzeniami cia̧żowymi z poradnigenetycznej szpitala uniwersyteckiego im. Dr. A. Jurasza w bydgoszczy]
315. Septic abortion: A 5-year experience at Siriraj hospital
316. Exploring the costs and economic consequences of unsafe abortion in Mexico City before legalisation
317. Potential use of single measurement of serum progesterone in detecting early pregnancy failure
318. [Study on leuprorelin acetate in treatment of uterine adenomyosis with infertility].
319. A method of abortion and a danger to the woman's health - How young Poles perceive hormonal post-coital contraception; ["Metoda aborcji i zagrożenie zdrowia kobiety" - Czyli jak młodzi Polacy postrzegaja̧ hormonalna̧ antykoncepcjȩ postkoitalna̧]
320. Survey women educational needs about routine O.C.P.s
321. Lown-ganong-levine syndrome in a 3-month-old infant with isolated left ventricular noncompaction
322. Women's Perspectives on Ultrasound Viewing in the Abortion Care Context
323. Association of the G2014G genotype in estrogen receptor 1 gene with failure of the mifepristone-induced termination of early pregnancy
324. Normal and abnormal early pregnancy
325. Imaging and imagining the fetus
326. A randomized comparative study on vaginal administration of acetic acid-moistened versus dry misoprostol for mid-trimester pregnancy termination
327. Mifepristone: ten years later
328. History of Medicine: Volume 4
329. Level of realization of reproductive potential in women of different age
330. Effect of endometrial stimulation on pregnancy outcome of in vitro fertilization patients
331. Office management of early pregnancy loss
332. Importance of a second dose of misoprostol in the management of medical abortion after failure of the initial treatment; [Intérêt de l'administration d'une seconde dose de misoprostol dans la prise en charge de l'interruption volontaire de grossesse après échec du traitement médical initial]
333. Efficacy of misoprostol in relation to uterine position in the treatment of early pregnancy failure
334. Assessment of efficacy and safety of medical treatment of non-viable first trimester pregnancy; [Ocena skuteczności i bezpieczeństwa leczenia zachowawczego w przypadkach ciąży obumarłej w pierwszym trymestrze]
335. Is mifepristone 100mg an effective alternative to standard dose for medical abortion; [Tıbbi abortus için 100 mg mifepriston standart doza etkili bir alternatif midir?]
336. Presenting features of women with uterine arteriovenous malformations
337. Placenta accreta: A cause of failed medical abortion
338. Vaccination against influenza in pregnant women - safety and effectiveness; [Szczepienia przeciwko grypie u kobiet ciężarnych - bezpieczeństwo i efektywność]
339. High failure rates of medical termination of pregnancy after introduction to a large teaching hospital
340. The modern abortion jurisprudence under Article 8 of the European Convention on Human Rights
341. The profile of women who seek emergency contraception from the family planning service
342. A study of psychiatric morbidity during second trimester of pregnancy subsequent to abortion in the previous pregnancy
343. A Profile of abortion cases in a tertiary care hospital
344. Clinical course of hepatitis B virus infection during pregnancy
345. Unsuccessful prior attempts to terminate pregnancy among women seeking first trimester abortion at registered facilities in Bihar and Jharkhand, India
346. Preterm infant born to a mother with severe pandemic H1N1 influenza; [Aǧır pandemik H1N1 i̇nfluenzalı anneden erken doǧan bebek]
347. Bacterial sacroiliitis and gluteal abscess after dilation and curettage for incomplete abortion
348. Contraceptive compliance - Why is contraceptive failure still so frequent?; [Kontrazeptive compliance - Warum kommt es immer wieder zum versagen der kontrazeptiven therapie?]
349. Failure to achieve the association of professors in gynecology and obstetrics objectives for abortion in third-year medical student curriculum
350. Sonographic quantification of endometrial changes after abortion with computer-assisted image analysis
351. Maternal level of pregnancy-associated plasma protein A as a predictor of pregnancy failure in threatened abortion.
352. Body failure: Medical views of women, 1900-1950
353. Contraceptive use among women seeking repeat abortion in Addis Ababa, Ethiopia.
354. Efficacy of Single Dose of Mifepristone Combined with Two Doses of Misoprostol in Early Medical Abortions
355. Mifepristone followed by home administration of buccal misoprostol for medical abortion up to 70 days of amenorrhoea in a general practice in Curaçao
356. Emergency contraception: Presently available formulations and controversies surrounding their use
357. Medical termination of pregnancy and concurrent contraceptive adoption in a tertiary referral hospital in Delhi.
358. Tanzanian lessons in using non-physician clinicians to scale up comprehensive emergency obstetric care in remote and rural areas
359. A comparitive study of use of intravaginal misoprostol after 48 hours and 4 hours of oral mifepristone for medical termination of pregnancy
360. Efficacy and safety of mifepristone and buccal misoprostol versus buccal misoprostol alone for medical abortion
361. Cervical pregnancy: A rare case of reimplantation after abortion. A case report; [La réimplantation cervicale de la grossesse, une complication méconnue des interruptions volontaires de grossesse. à propos d'un cas]
362. Setting up a medical abortion service for failure
363. Fulminant postpartum cerebral vasoconstriction syndrome
364. Abortion services and military medical facilities
365. Ultrasonographic evaluation of endometrial changes using computer assisted image analysis
366. Abortion training at multiple sites: An unexpected curriculum for teaching systems-based practice
367. Effects of leonurine hydrochloride on medically induced incomplete abortion in early pregnancy rats
368. Reproductive health information and abortion services: Standards developed by the European Court of Human Rights
369. Parvovirus B19 infection frequency in placenta of fetal loss cases in children medical center, Tehran, Iran
370. Emergency Contraception: Plan B
371. Effects of taking estrogen and progestogen after medical abortion on reducing vaginal hemorrhage time: a randomized-controlled trial
372. Can midlevel health-care providers administer early medical abortion as safely and effectively as doctors? A randomised controlled equivalence trial in Nepal
373. Cleft lip and palate: Series of unusual clinical cases
374. Survey of the attitude to, the knowledge and the practice of contraception and medical abortion in women who attended a family planning clinic
375. Does congenital heart disease severely jeopardise family life and pregnancies? Obstetrical history of women with congenital heart disease in a single tertiary centre
376. Illegal abortion with misoprostol in Guadeloupe; [Avortements illégaux par le misoprostol en Guadeloupe]
377. Short and long term mortality rates associated with first pregnancy outcome: Population register based study for Denmark 1980-2004
378. Can women accurately assess the outcome of medical abortion based on symptoms alone?
379. Factors associated with severe complications in unsafe abortion
380. Reply of the Authors: Setting up a medical abortion service for failure
381. Minors' behavioral responses to parental involvement laws: Delaying abortion until age 18
382. First trimester bleeding
383. Representations and uses of emergency contraception in West Africa. A social anthropological reading of a northern medicinal product
384. Clinical manifestations of pregnancy in patients with Takayasu arteritis: Experience from a single tertiary center
385. A pilot study on the use of a 7-day course of letrozole followed by misoprostol for the termination of early pregnancy up to 63 days
386. Can the outcome of the next pregnancy be predicted at the time of induced abortion?
387. Conscientious objection to sexual and reproductive health services: International human rights standards and european law and practice
388. Evaluation of a network of medical abortion providers in two districts of Maharashtra, India
389. Clinical pattern of gynecological/early pregnancy complaints and the outcome of pelvic sonography in a private diagnostic center in Ilorin
390. The invisible pregnant athlete and the promise of title IX
391. Comparison of vaginal and sublingual misoprostol for second trimester abortion: Randomized controlled equivalence trial
392. Situation analysis of patients attending TU teaching hospital after medical abortion with problems and complications
393. Evaluation of the teratogenic risks in gestations exposed to misoprostol; [Avaliação de riscos teratogênicos em gestações expostas ao misoprostol]
394. Effect of abortion, in vitro fertilization, and other causes of prenatal death on life expectancy in the United States from 1925 to 2005
395. Obstetric and perinatal outcome of teenage pregnancy.
396. Comparative effectiveness, safety and acceptability of medical abortion at home and in a clinic: A systematic review; [Comparación de la efectividad, la seguridad y la aceptación de los abortos médicos practicados en el domicilio con aquellos realizados en la clínica: Una revisión sistemática]
397. Malaria in pregnancy
398. Developmental toxicity of misoprostol. An update; [Toxicidad del misoprostol sobre la gestación. Revisión de la literatura]
399. Clinical diagnosis of completeness of medical abortion by nurses: A reliability study in Mozambique
400. About the bioethics of abortion at request in Romania. A case presentation
401. Increased risk for medical abortion failure for multiparous women
402. Religion, conscience, and controversial clinical practices
403. Clandestine abortion in Port Harcourt: users' profile and motivation.
404. Pathophysiology of mifepristone-induced septic shock due to Clostridium sordellii
405. Medical abortion in rural Tamil Nadu, South India: A quiet transformation
406. Catastrophic antiphospholipid syndrome
407. Monitoring medical abortion using mifepristone/misoprostol combination with ultrasonogram and serum human chorionic gonadotropin
408. Pregnancy outcome after suicide attempt by drug use: A Danish population-based study
409. Survey of the attitude to, knowledge and practice of contraception and medical abortion in women attending a family planning clinic
410. Homocysteine, folic acid and vitamin B12 concentration in patients with recurrent miscarriages
411. From concept to practice: The recent history of preterm delivery prevention. Part II: Subclinical infection and hormonal effects
412. A comparison of the efficacy of sublingual and oral misoprostol 400 microgram in the management of early pregnancy failure: A randomized controlled trial
413. Congenital heart disease and acquired valvular lesions in pregnancy; [Angeborene herzfehler und erworbene herzklappenfehler in der schwangerschaft]
414. Vaginal misoprostol for salvage therapy after failed medical abortion
415. Moebius syndrome due to the use of misoprostol. Case report; [Sindrome de moebius: Fetopatía por misoprostol. Reporte de un paciente]
416. Complications of unsafe abortion: A case study and the need for abortion law reform in Nigeria
417. The frequency of chromosomal abnormalities in patients with reproductive failure
418. Medical termination of pregnancy in the second trimester
419. Acceptability of home-use of misoprostol in medical abortion
420. Malpractice Issues in Radiology: Wrongful Life
421. Current diagnostics and treatments for immune mediated recurrent spontaneous miscarriages and IVF implantation failures - A review; [Az immunpatológiai hátteru visszatéro spontán vetélések és sikertelen IVF beültetések korszeru diagnosztikája és kezelési lehetoségei]
422. Use of mifepristone and sublingual misoprostol for early medical abortion
423. Umbilical cord lesions in early intrauterine fetal demise
424. Termination of early pregnancy using flexible, low-dose mifepristone-misoprostol regimens
425. Elective abortion as a primary health service in rural India: Experience with manual vacuum aspiration
426. Doctors speak of their experience in announcing a diagnosis. Announcing to parents serious-chronic disease in their child; [Des médecins parlent de leur expérience de l'annonce. L'annonce d'une maladie grave de l'enfant à ses parents]
427. Pathophysiological and clinical aspects of combat anticholinesterase poisoning
428. The clinical use of karyotyping spontaneous abortions.
429. Immunological aspects of pregnancy failures - Novel therapeutic approaches; [Sikertelen terhességek immunológiai háttere: Újabb terápiás lehetoségek]
430. North Florida Women's Health Services v. State.
431. Homocysteine serum concentration and uterine artery color Doppler examination in cases of recurrent miscarriages with unexplained etiology
432. Mifepristone-misoprostol abortion: A trial in rural and urban Maharashtra, India
433. Medically-induced abortion and risk of reproductive failures in subsequent pregnancy; [Medicinsk induceret abort og risiko for bivirkninger ved en efterfølgende graviditet - Sekundærpublikation]
434. Catastrophic antiphospholipid syndrome during pregnancy and puerperium: Maternal and fetal characteristics of 15 cases
435. Antiphospholipid syndrome.
436. Value of hysteroscopy and laparoscopy in differential diagnosis of gestational trophoblastic neoplasia
437. Morbidity that is associated with curettage for the management of spontaneous and induced abortion in women who are infected with HIV
438. Post legalisation challenge: minimizing complications of abortion.
439. Pregnancy and cardiac surgery with cardiopulmonary bypass; [Terhesség és nyitott szívmutét.]
440. Does methotrexate confer a significant advantage over misoprostol alone for early medical abortion? A retrospective analysis of 8678 abortions
441. Failed medical termination of twin pregnancy with mifepristone: A case report
442. Management of gestational trophoblastic tumours: A five-year clinical experience
443. Medical management of missed abortion: A randomized clinical trial
444. Alteration of TH1 and TH2 cells by intracellular cytokine detection in patients with unexplained recurrent abortion before and after immunotherapy with the husband's mononuclear cells
445. Choosing abortion: Teens who make the decision without parental involvement
446. A pilot study of mifepristone and misoprostol administered at the same time for abortion up to 49 days gestation
447. Misoprostol and pregnancy: Risk of malformations
448. Antiprogesterone for medical abortion
449. Application of multiplex quantitative fluorescent PCR with non-polymorphic loci in prenatal diagnosis.
450. The effects of electromagnetic field on pregnancy; [Elektromanyetik alanin gebelik üzerine etkileri]
451. Emergency contraception for adolescents; [La contraccezione d'emergenza in adolescenza. Considerazioni generali e proposte di un gruppo di lavoro]
452. Non-developing pregnancy: Histological and immunohistochemical markers of endocrine disorders in endometrial scrapes
453. WHO multinational study of three misoprostol regimens after mifepristone for early medical abortion. I: Efficacy
454. Alternatives to mifepristone for early medical abortion
455. Sequelae of reentry of tachycardia during intrauterine development and in the newborn period; [Dôsledky reentry tachykardie prítomnej počas vnútromaternicového vývoja a v novorodeneckom období]
456. [The fetal adrenal gland in risk pregnancy].; [Nadbubrezne zlezde fetusa u rizicnim trudnoćama.]
457. Ultrasonographic characteristics in patients clinically diagnosed with threatened abortion
458. Success rate of second-trimester termination of pregnancy using misoprostol
459. Planned Parenthood of Rocky Mountains v. Owens.
460. Age, parity, history of abortion and contraceptive choices affect the risk of repeat abortion
461. Improving the quality of care after spontaneous abortions in rural Senegal; [Amélioration de la qualité des soins après avortement (SAA) en zone rurale au Sénégal]
462. Legal duties to respect abortion choices
463. Uterine artery embolization in the treatment and prevention of postpartum hemorrhage
464. Pregnancy following uterine artery embolization with polyvinyl alcohol particles for patients with uterine fibroid or adenomyosis
465. Effects of making emergency contraception available without a physician's prescription: A population-based study
466. Pregnancy, fertility, and recurrence risk in corrected tetralogy of Fallot
467. Secular trends in uncertain-sex births and proportion of male births in Norway, 1967-1998
468. Acute renal failure in pregnancy in a developing country: Twenty years of experience
469. Randomized study on the effect of adding oxytocin to ethacridine lactate or misoprostol for second-trimester termination of pregnancy
470. Risk of complications during pregnancy in women with congenital aortic stenosis
471. Mifepristone and misoprostol administered simultaneously versus 24 hours apart for abortion: A randomized controlled trial
472. Medical abortion in family practice: A case series
473. Personal accounts of 'near-miss' maternal mortalities in Kampala, Uganda
474. Abortion Policies and Practices in Chile: Ambiguities and Dilemmas
475. Soluble MHC Class I chain-related molecule serum levels are predictive markers of implantation failure and successful term pregnancies following IVF
476. Induced abortions in Khamano Block of Fatehgarh Sahib district, Punjab
477. Failure of sterilization after clip placement
478. Endometrial Thickness and Serum β-hCG as Predictors of the Effectiveness of Oral Misoprostol in Early Pregnancy Failure
479. Therapeutic termination of second trimester pregnancies with low dose misoprostol
480. A short-scheme protocol of gemeprost for midtrimester termination of pregnancy with uterine scar
481. A fatal case of clostridium sordellii septic shock syndrome associated with medical abortion
482. Misoprostol as the primary agent for medical abortion in a low-income urban setting
483. Results and lessons learned from a small medical abortion clinical study in Turkey
484. A randomised comparative study on sublingual versus vaginal administration of misoprostol for termination of pregnancy between 13 to 20 weeks
485. Bleeding patterns after vaginal misoprostol for treatment of early pregnancy failure
486. Cytomegalovirus and the expression of immunological markers in reproductive failure
487. Informed consent or institutionalized eugenics? How the medical profession encourages abortion of fetuses with down syndrome
488. Medical student awareness of sexual health is poor
489. Antiphospholipid Syndrome
490. Misoprostol for termination of second trimester pregnancy in a scarred uterus
491. Misoprostol for women's health: A review
492. Methotrexate/Misoprostol Embryopathy: Report of Four Cases Resulting From Failed Medical Abortion
493. Contraception: From accessibility to efficiency
494. Estimates of pregnancies averted through California's family planning waiver program in 2002
495. Menstrual induction with mifepristone and misoprostol
496. Cushing's syndrome during pregnancy secondary to adrenal adenoma: Metyrapone treatment and laparoscopic adrenalectomy
497. Abortion procedures in a tertiary care institution in India
498. Tucson Woman's Clinic v. Eden.
499. Fertility and pregnancy outcomes following uterine devascularization for severe postpartum haemorrhage
500. Medical-social aspects pregnancy, labor and puerperium in patients the solitary mother home in Karwowo near Szczecin; [Medyczno-społeczne aspekty przebiegu ciazy, porodu i połogu u pacjentek--pensjonariuszek domu samotnej matki w Karwowie koło Szczecina.]
501. Primary cause of death in extremely low birth weight infants
502. Cervical ectopic pregnancy: Diagnosis with endovaginal ultrasound examination and successful treatment with methotrexate
503. District court finds proposed Iowa women's health facility does not need state approval.
504. Outcome of pregnancy in Takayasu arteritis
505. Ethics and reproductive health: A principled approach
506. A medical method of early pregnancy termination using tamoxifen and misoprostol
507. Abortion related acute renal failure - A study in Dhaka Medical College Hospital
508. Management of maternal Amanita phalloïdes poisoning during the first trimester of pregnancy: A case report and review of the literature
509. How safe motherhood in India is.
510. T-Helper 1-Type Immunity to Trophoblast in Women With Recurrent Spontaneous Abortion
511. Severe acute maternal morbidity: A pilot study of a definition for a near-miss
512. Medical claims and women's experience. Physician-performed abortions in the Weimar Republic; [Heilanspruch und medizinische Kunstfehler. Abtreibungen durch Arzte in der Weimarer Republik: offizielle Beurteilung und weibliche Erfahrung.]
513. 200 mg mifepristone was as effective as 600 mg when used with oral misoprostol for early medical abortion
514. A phase III multicentre study on medical termination of early pregnancy with two regimens of mifepristone followed by PG05
515. Contours of development
516. Bio-artificial liver from cultured human foetal hepatocytes: feasibility and prospects.
517. An analysis of the cost of incomplete abortion to the public health sector in South Africa - 1994
518. Study of pregnancy outcome over a period of five years in a postgraduate institute of west Bengal.
519. Decreasing the need for abortion: Challenges and constraints
520. Factors associated with the persistence of retained products following D and C. Is curettage still the treatment of choice?; [Factores que se asocian a la persistencia de restos abortivos tras legrado evacuador. Sigue siendo el curetaje el tratamiento de eleccion?]
521. Psychopathology of recurrent spontaneous abortions; [Fausses couches a repetition: Origine psychogene et/ou psychopathologie induite]
522. Reproductive health and blurred professional boundaries
523. Contraception; [La contraccezione.]
524. A Medical Record Linkage Analysis of Abortion Underreporting
525. The perimenopause and contraception
526. Unsafe abortion: An avoidable tragedy
527. Factors influencing the delivery of abortion services in Ontario: A descriptive study
528. California may become first state to mandate insurance coverage for contraception.
529. Are serum progesterone levels predictive of recurrent miscarriage in future pregnancies?
530. Maternal and fetal outcomes of subsequent pregnancies in women with peripartum cardiomyopathy
531. Putting the ICPD Plan of Action to work. Turkey. Women's health tops the agenda.
532. Obstetric determinants of neonatal survival: Antenatal predictors of neonatal survival and morbidity in extremely low birth weight infants
533. Perinatal infection with the human immunodeficiency virus; [Perinatalna infekcija virusom humane imunodeficijencije.]
534. Abortion trends in Singapore: A 25-year review
535. An overview of unsafe abortion in Africa. Foreword.
536. First-trimester spontaneous abortions and the incidence of human immunodeficiency virus seropositivity
537. Women's Medical Center of Northwestern Houston v. Bell.
538. The effects of the antihormones RU486 and tamoxifen on fetoplacental development and placental bed vascularisation in the rat: A model for intrauterine fetal growth retardation
539. Management of incomplete abortion with manual vacuum aspiration in comparison to sharp metallic curette in an Ethiopian setting
540. Manual vacuum aspiration technique draws interest.
541. Misoprostol alone--a new method of medical abortion?
542. Pregnancy related acute renal failure - Still a major problem
543. Reproductive failure due to spontaneous abortion and recurrent miscarriage
544. Change in serum β-human chorionic gonadotropin after abortion with methotrexate and misoprostol
545. An experience with misoprostol for the induction of first trimester abortions in a secondary hospital in South Africa
546. Media reviews
547. WomanCare of Southfield, P.C. v. Granholm.
548. Angiographic transcatheter embolization in gynaecological practice
549. Management of incomplete spontaneous abortion with suction curettage in the pediatric emergency department
550. Clinical study of four cases with malignant gestation trophoblastic tumor after mifepristone abortion
551. Abortion, breast cancer, and informed consent
552. Medical abortion at 57 to 63 days' gestation with a lower dose of mifepristone and gemeprost. A randomized controlled trial
553. Pediatrics
554. Methotrexate compared with mercaptopurine for early induced abortion
555. Methotrexate and misoprostol used alone or in combination for early abortion
556. The outcome of in vitro fertilization in unexplained habitual aborters concurrent with secondary infertility
557. A comparison of misoprostol with and without laminaria tents for induction of second-trimester abortion
558. Post-abortion counseling; [L'entretien post-IVG.]
559. A pilot study of the effect of methotrexate or combined oral contraceptive on bleeding patterns after induction of abortion with mifepristone and a prostaglandin pessary
560. Clinical observation on termination of early pregnancy of 213 cases after caesarian section with repeated use of mifepristone and misoprostol.
561. Fetal complications associated with unsuccessful attempts at termination of pregnancy; [Fetale komplikationen nach misslungenem schwangerschaftsabbruch im ersten trimester]
562. Determinants of abortion among women admitted to hospitals in Fortaleza, North Eastern Brazil
563. Systemic inflammatory response syndrome, organ failure, and outcome in critically ill obstetric patients treated in an ICU
564. The influence of abortion legislation on maternal mortality
565. Contraceptive failure rates: New estimates from the 1995 National Survey of Family Growth
566. WomanCare of Southfield, P.C. v. Granholm.
567. Study of side-effects of Cu-T as intra-uterine contraceptive device in post medical termination of pregnancy and interval cases
568. The fight for reproductive rights in Central and Eastern Europe. Poland: Catholic backlash.
569. Abortion related acute renal failure - A study in Dhaka Medical College Hospital
570. Study results translate to better services.
571. MISH publishes new framework for fear-based, abstinence-only education.
572. Induced abortion: Physician training and practice patterns
573. Outbreaks of porcine reproductive failure: Report on a collaborative field investigation
574. New York appeals court finds state not liable for death after physician under review performed abortion.
575. Pregnancies, growth and development of children conceived by subzonal injection of spermatozoa
576. Psychosocial aspects of induced abortion
577. The political challenges and educational opportunities around very early abortion.
578. Lowering the doses of mifepristone and gemeprost for early abortion: A randomised controlled trial
579. When is "emergency" contraception the right name for postcoital treatment?
580. APF. Open letter to the political parties, on the abortion situation in Portugal; [APF. Carta aberta aos partidos politicos, sobre a situacao do aborto em Portugal.]
581. Partial-birth abortion, congress, and the constitution
582. Latin American hospitals improve postabortion care. Maternal health.
583. Early pregnancy failure - Current management concepts
584. Misoprostol use in obstetrics and gynecology in Brazil, Jamaica, and the United States
585. Implantable cardioverter-defibrillators and pregnancy: A safe combination?
586. Oriéntame: Preventing and solving problems related to unwanted pregnancy for 25 years in Colombia
587. Meeting women's need for a flexible abortion service: Retrospective study of a specialist day-care unit
588. Misoprostol (cytotec8)exposure pregnancy : A french coijaborative study
589. Use of misoprostol in gynecology and obstetrics; [Utilisation du misoprostol en gynécologie-obstétrique]
590. Abortion needs of women in India: A case study of rural Maharashtra
591. Diagnosis of aneuploidy in archival, paraffin-embedded pregnancy-loss tissues by comparative genomic hybridization
592. Comparison of two doses of mifepristone in combination with misoprostol for early medical abortion: A randomised trial
593. Eisenmenger syndrome. Factors relating to deterioration and death
594. Abortion in Belgium, 1880-1940; [Abortus in België 1880-1940]
595. The efficacy of medical abortion: A meta-analysis
596. Quality focuses on clients' needs.
597. Abortion at Gondar College Hospital, Ethiopia
598. Medical genetics in reproductive medicine; [Vyuzití lékarské genetiky v reprodukcní medicíne.]
599. Planned Parenthood of Southern Arizona v. Lawall.
600. Prevalence of and attitudes to abortion among migrant women in Sydney
601. Clinical study of termination of early pregnancy by combination of dl-15-methyl-prostaglandin F2 alpha and RU 486
602. Cervical Internal OS Cerclage: Description of a New Technique and Comparison with Shirodkar Operation
603. Early Termination of Pregnancy with Mifepristone (RU 486) and the Orally Active Prostaglandin Misoprostol
604. Mifepristone (RU 486) — An Abortifacient to Prevent Abortion?
605. Increased Transient Tachypnea of the Newborn in Infants of Asthmatic Mothers
606. STD prevention and unplanned pregnancy: A U.K. perspective; [PREVENTION DES MST ET GROSSESSES INDESIREES EN GRANDE-BRETAGNE]
607. Amniocentesis before 15 weeks' gestation: technical aspects and obstetric risks
608. Birth control and social group: contraception, accidental pregnancies and abortion; [Maitrise de la fecondite et appartenance sociale: contraception, grossesses accidentelles et avortements]
609. Clinical trial on termination of early pregnancy with RU486 in combination with prostaglandin
610. Aspects of the significance of operatively corrected heart valve failure on fertility; [ASPEKTE ZUR BEDEUTUNG OPERATIV KORRIGIERTER HERZKLAPPENVITIEN AUF DIE FERTILITAT]
611. Characteristics of women having abortion in China
612. Demand for abortion. Special aspects of drug-induced abortion; [La demande d'avortement. Aspects particuliers de l'IVG médicamenteuse.]
613. Must a Catholic hospital inform a rape victim of the availability of the 'morning-after pill'?
614. Pregnancy: The effect of dose of mifepristone and gestation on the efficacy of medical abortion with mifepristone and misoprostol
615. Analysis of motivation of sterilised women wishing to be refertilised
616. Adolescent pregnancy: occurrence and consequences.
617. Maternal mortality and morbidity associated with clandestine abortions.
618. Compliance with new ban means no mention of abortion.
619. Manual vacuum aspiration saves lives.
620. Is ward evacuation for uncomplicated incomplete abortion under systemic analgesia safe and effective? A randomised clinical trial
621. What is needed to ensure the health and survival of mother and baby?
622. Effectiveness of the diaphragm, used continuously, without spermicide
623. Termination of early pregnancy by two regimens of mifepristone with misoprostol: a multicentre clinical trial
624. Fertility following ectopic pregnancy
625. Abortions in a hospital setting: hidden realities in Dar es Salaam, Tanzania
626. A thoroughly regressive law.
627. Medical Evacuation of First Trimester (Twelve Weeks Gestation) Incomplete Abortion and Missed Abortion
628. Youth often risk unsafe abortions.
629. Microbiology relevant to recurrent miscarriage
630. Resurgence of Congenital Rubella Syndrome in the 1990s: Report on Missed Opportunities and Failed Prevention Policies Among Women of Childbearing Age
631. The abortion pill: A solution for unsafe abortions in developing countries?
632. 5 Embryo manipulation and experimentation
633. Radiology rounds. Intrauterine contraceptive device.
634. Septic abortion
635. Contraceptive efficacy; [A eficacia contraceptiva.]
636. A pilot study of acceptability of RU486 and ONO 802 in a Chinese population
637. A comparison of the costs of manual vacuum aspiration (MVA) and evacuation and curettage (E and C) in the treatment of early incomplete abortions in Kenya.
638. Four perspectives of women's health. Workshop participants talk about women's health issues in four countries. [Hong Kong].
639. Mifepristone in mid trimester termination of pregnancy-value for money?
640. Cuban health system -- call it superior? [letter]
641. Counseling women about childbearing and childrearing risks
642. Human rights, maternal mortality and reproductive health; [Derechos humanos, mortalidad materna y salud reproductiva.]
643. Current management of complete and partial molar pregnancy
644. The midwife's role in abortion care
645. Cabergoline: A Review of its Pharmacological Properties and Therapeutic Potential in the Treatment of Hyperprolactinaemia and Inhibition of Lactation
646. Of human rights and women's health.
647. Family planning in Europe.
648. On inhuman practices in gynaecology and their victims in Germany during national socialist rule: A study of concrete events; [UBER INHUMANE PRAKTIKEN DER FRAUENHEILKUNDE IM NATIONALSOZIALISMUS UND IHRE OPFER: UNTERSUCHUNG ZU KONKRETEN EREIGNISSEN]
649. Hummel v. Reiss.
650. The contribution of environmental teratogens to embryonic and fetal loss
651. Cost-effectiveness of levonorgestrel subdermal implants: Comparison with other contraceptive methods available in the United States
652. The clinical management of abortion.
653. Mifepristone (RU 486) Compared with High-Dose Estrogen and Progestogen for Emergency Postcoital Contraception
654. Options in making use of pregnancy history in planning and analysing studies of reproductive failure
655. Termination of early pregnancy using RU 486 alone or in combination with prostaglandin. The RU 486 Collaboration Group
656. Medical abortion in women of ≤56 days amenorrhoea; a comparison between gemeprost (a PGE1 analogue) alone and mifepristone and gemeprost
657. Incidence of induced abortion determined by the randomised response technique.
658. Future uncertain as Congressional drive to block Title X gag rule fails.
659. "Criminality".
660. Mandatory parental involvement/judicial bypass laws: Do they promote adolescents' health?
661. Estimates of demand for abortion among Soviet immigrants in Israel.
662. Adverse pregnancy outcome and childhood malignancy with reference to paternal welding exposure
663. Ultrasound in Republic of Yemen; [ECHOGRAPHIE EN REPUBLIQUE DU YEMEN]
664. "RU486: Misconceptions, myths and morals." A critique of the criticisms.
665. Current management of molar pregnancy
666. Late midtrimester medical pregnancy terminations: Three different procedurew with prostaglandin F2α and laminaria tents
667. Contraceptive behavior of Jerusalem women seeking pregnancy counseling, 1980-1989
668. Trauma in Pregnancy: Predicting Pregnancy Outcome
669. Design options and methodological fallacies in the studies of reproductive failures
670. A study of 500 cases of acute renal failure (1978-1991)
671. Oral contraception noncompliance: The extent of the problem
672. Emergency contraceptive pills: A simple proposal to reduce unintended pregnancies
673. Obstetric and gynecologic dysfunction in the Ehlers-Danlos syndrome
674. Reduced Fertility among Women Employed as Dental Assistants Exposed to High Levels of Nitrous Oxide
675. Outcome of first-trimester exposure to low-dose methotrexate in eight patients with rheumatic disease
676. Contraceptive practices of women attending for termination of pregnancy - A study from South Australia
677. Abortion policy and women's health in developing countries
678. Restrictive lung disease in pregnancy
679. Clinical study of early pregnancy termination by administration of DL-15-methyl prostaglandin F2 alpha combined with tamoxifen or norethisterone.
680. The Feasibility of Early Second Trimester Pregnancy (12 to 14 weeks) Termination with Single Intra‐Amniotic Injection of 15‐Methyl PGF2α
681. Clinton reform proposal faces Congressional test on covered services, cost.
682. Prenatal Diagnosis and Late Termination: A Legal Perspective
683. Termination of early pregnancy by two regimens of mifepristone with misoprostol and mifepristone with PG05-a multicentre randomized clinical trial in China
684. Comparison of adolescent and adult experiences with norplant levonorgestrel contraceptive implants
685. Autoantibodies in Normal and Abnormal Pregnancy
686. The problem of teenage pregnancy
687. Syphilitic endometritis causing first trimester abortion: A potential infectious cause of fetal morbidity in early gestation
688. Induced abortion: a vulnerable public health problem; [El aborto inducido: un problema de salud publica vulnerable.]
689. Sequelae of premature sexual life
690. Acute renal failure in blacks and indians in South Africa- Comparison after 10 years
691. Problems loom ahead.
692. Contraceptive discontinuation among white, black, and Hispanic adolescents
693. Report of survey: pregnancy outcomes in medical laboratory technologists.
694. Induction of abortion with mifepristone and misoprostol in early pregnancy
695. A family planning center: role of the nurse; [Un centre de planification familiale: place et rôle de l'infirmière.]
696. The abortion battle: The Canadian scene
697. Cost-effectiveness of managing abortions: manual vacuum aspiration (MVA) compared to evacuation by curettage in Tanzania.
698. USA: Victory over gag rule for family planning groups
699. How family planning can save lives in Africa.
700. Hysteroscopic findings after missed abortion
701. The pattern of maternal mortality at maternity hospital Kuala Lumpur.
702. Unusual intrauterine objects: Potential pitfalls in ultrasonographic identification
703. Health benefits of legal abortion: an analysis.
704. Ghana.
705. Decision making by single women seeking abortion.
706. Prostacyclin deficiency in a young woman with recurrent thrombosis
707. Maternal mortality at the University of Benin Teaching Hospital Benin City, Nigeria.
708. Post abortion syndrome: myth or reality?
709. Sexual activity in girls under 16 years of age
710. Assessment of rapid radioimmunoassay for urinary β-human chorionic gonadotropin (βhcg-ria) in early pregnancy
711. False positive results of tests for syphilis and outcome of pregnancy: A retrospective case-control study
712. Abortion due to infection with chlamydia psittaci in a sheep farmer's wife
713. The usefulness of the chorionic villi technic in prenatal diagnosis; [Korisnost korion frondozum tehnike u prenatalnoj dijagnozi.]
714. Fatal hemorrhage from legal abortion in the United States
715. Acute Renal Failure in Pregnancy
716. Acute Renal Failure in Pregnancy: 1987
717. Mid-trimester pregnancy termination in teenage women
718. Termination of pregnancy in Papua New Guinea: the traditional and contemporary position.
719. Postcoital contraception: a report from the London Brook Advisory Centres, United Kingdom.
720. Injectable contraception.
721. Abortion at the first trimester of pregnancy. The risks of responsibility from jurisprudence and the study of 81 complaints; [L'I.V.G. DU PREMIER TRIMESTRE ET SES RISQUES D'ENGAGEMENT DE LA RESPONSABILITE]
722. Wish of pregnancy - refusal of pregnancy - ambivalence in family planning; [DESIR DE GROSSESSE - REFUS DE GROSSESSE OU LES EMBUCHES DU DESIR DANS LE PLANNING FAMILIAL]
723. Planned Parenthood continues prescribing birth control for teens.
724. RU 486 (mifepristone): From clinical trials to the perspectives for clinical use; [RU 486 (MIFEPRISTONE): DES ESSAIS CLINIQUES AUX PERSPECTIVES D'UTILISATION]
725. Voluntary interruption of pregnancy: not an insignificant act; [L'I.V.G.: un acte non anodin.]
726. Die neuere Entwicklung des Arztrechts
727. Maternal mortality at the University of Nigeria Teaching Hospital, Enugu: a 10-year survey.
728. Concurrent copper T insertion with medical termination of pregnancy in women with previous caesarean section delivery.
729. Choice at any cost.
730. Maternal mortality in a semi-urban Nigerian community
731. Risk management in pregnancy termination
732. Induction of abortion during the second trimenon of pregnancy - endocervical PG-E2 gel application, intramuscular application of sulproston and combined treatment (endocervical PG-E2 gel/intramuscular sulproston); [ABORTINDUKTION IM ZWEITEN SCHWANGERSCHAFTSTRIMENON. ENDOZERVIKALE PGE2-GEL-APPLIKATION, INTRAMUSKULARE SULPROSTONAPPLIKATION UND KOMBINIERTE (ENDOZERVIKALE PGE2-GEL/INTRAMUSKULARE SULPROSTON) BEHANDLUNG]
733. Public Law No. 262, 25 March 1988.
734. Sex education: prerequisite for family planning; [Education sexuelle: prealable necessaire a la planification familiale.]
735. Medical responsibility in the practice of voluntary first trimester terminations of pregnancy. Preventive measures drawn from jurisprudence and from the study of 50 files of legal action or threatened legal action; [RESPONSABILITE MEDICALE DANS LA PRATIQUE DES INTERRUPTIONS VOLONTAIRES DE GROSSESSE DU PREMIER TRIMESTRE. MESURES PREVENTIVES TIREES DE LA JURISPRUDENCE ET DE L'ETUDE DE 50 DOSSIERS DE PLAINTES OU DE MENACES DE PLAINTES]
736. Contraception: Failure in practice
737. Complications of induced abortions; [Complications des interruptions volontaires de grossesse.]
738. Teen pregnancy: effect on family well-being.
739. The experience of carrying out the technical responsibility system in the birth control of Peng County, Sichuan Province
740. Abortion in today's world.
741. How antiabortion legislation would put doctors in a bind.
742. Family planning clinics share blame for men's failure to use services.
743. The abortion paradox
744. The Safety and Efficacy of Chorionic Villus Sampling for Early Prenatal Diagnosis of Cytogenetic Abnormalities
745. Acute renal failure in pregnancy - A decade of change
746. The applied of computer for analysis on contraceptive efficacy of IUD of rural women in Guangdong province
747. Formulation and noncontraceptive uses of the new, low-dose oral contraceptive
748. Assessment of results and notes on the organisational aspects of Law No. 194 of 1978 as applied in a provincial hospital. Interruption of pregnancy; [ANALISI DEI RISULTATI, CONSIDERAZIONI DI ORDINE ORGANIZZATIVO CIRCA LA LEGGE 194/78 APPLICATA IN UN ENTE OSPEDALIERO DI PROVINCIA]
749. Amyloidosis associated with igg (λ) m-proteinemia: A case of autopsy
750. Diaphragm Method Contraceptors: Implications for Service Organization and Delivery
751. The clinical aspects of acute renal failure, with particular reference to the post-partum period; [ASPETTI CLINICI DELL'INSUFFICIENZA RENALE ACUTA CON PARTICOLARE RIGUARDO ALL'IRA POST-PARTUM]
752. Termination of Early Pregnancy by the 3β-Hydroxysteroid Dehydrogenase Inhibitor Epostane
753. An account of 2180 cases of percoelioscopic tubal ligation using Yoon's mechanical method; [A PROPOS DE 2180 STERILISATIONS TUBAIRES PER-COELIOSCOPIQUES PAR PROCEDE MECANIQUE DE YOON]
754. Controversy surrounds use of test for open spina bifida.
755. Contraceptive coverage after medical termination of pregnancy.
756. Azzolino v. Dingfelder.
757. Contraceptive problems unique to the United States
758. Fetal Cystic Hygroma: Cause and Natural History
759. Medical termination of pregnancy: a study of acceptor characteristics.
760. Yoruba traditional healers' knowledge of contraception, abortion and infertility
761. Ethical issues in prenatal diagnosis
762. Women's Medical Center of Providence v. Roberts.
763. Garrison v. Medical Center of Delaware, Inc., 12 December 1989.
764. Pregnancy after heart valve replacement
765. Coping with fertility in Israel: A case study of culture clash
766. Fetal death ratios in a prospective study compared to state fetal death certificate reporting
767. The characteristics of and the contraceptive practice among women seeking therapeutic termination of pregnancy in the Scottish Highlands.
768. Risks and costs of illegally induced abortion in Bangladesh
769. Contraception in adolescence: knowing is not enough; [La contraception à l'adolescence: savoir ne suffit pas.]
770. Abortion techniques in Australia: a history.
771. Chorionic villi sampling: Cytogenetic and clinical findings in 500 pregnancies
772. Induction of abortion in the second trimester of pregnancy by intramuscular 15-methyl prostaglandin F(2α) 15M)
773. Abortion: an attitude study of professional staff at Ramathibodi Hospital.
774. The Spectrum of Relapsing Fever in the Rocky Mountains
775. Evaluating well-woman clinics
776. Gregory v. Pembrokeshire Health Authority [31 January 1989].
777. Attitudes to contraception in women seeking termination of pregnancy
778. Abortion and legal policy
779. Judgment, 28 January 1987.
780. The effect of cardiac surgery on a success rate of postoperative pregnancy. The results of 108 cases of pregnancy after cardiac surgery for congenital and acquired heart diseases
781. Reproductive Performance of Patients Treated with Clomiphene Citrate
782. Postcoital contraception: a delicate political issue.
783. Chronic Bone Marrow Failure Due to Persistent B19 Parvovirus Infection
784. Two cases of auto-immune hemolytic anemia during pregnancy
785. Wilson v. Kuenzi, 17 May 1988.
786. Gestational trophoblastic disease: Treatment results at the brewer trophoblastic disease center
787. Rendering follicle tubes permeable again after post-partum sterilization. A report on 25 cases, should post-partum sterilization be performed or not?; [REPERMEABILISATION TUBAIRE APRES STERILISATION DU POST-PARTUM. A PROPOS DE 25 CAS, FAUT-IL REMETTRE EN CAUSE LA STERILISATION DU POST-PARTUM?]
788. The Montana Fetal Genetic Pathology Program and a review of prenatal death in humans.
789. Comment: "Analysis of and reflections on some abortion request statistics" (letter); [A propos de: "Analyse d'une statistique de demandes d'I.V.G. et les reflexions qu'elle suscite".]
790. Psychological decision making with regard to motherhood by women with high-risk pregnancy and normal pregnancy; [Psychologiczne uwarunkowania decyzji o macierzyństwie kobiet w ciazy wyosokiego ryzyka i w ciazy prawidłowej.]
791. Use of Quinine for Self-Induced Abortion
792. The induction of abortion in the second trimester by combined administration of Minprostin and Sulproston, compared with the use of Sulproston alone; [KOMBINIERTER EINSATZ VON MINPROSTIN UND SULPROSTON BEI DER ABORTEINLEITUNG IM II. TRIMENON UND BEI DER GEBURTSEINLEITUNG BEIM TOTEN KIND IM VERGLEICH ZUM ALLEINIGEN SULPROSTONEINSATZ]
793. Characteristics of medical termination of pregnancy acceptors in Pondicherry State, 1972-1976.
794. Treatment of epilepsy in women of childbearing age.
795. Tissue distribution of cocaine in a pregnant woman
796. Fatal septic abortion in the United States, 1975-1977
797. Lamicel is More Effective in Preparing the Cervix for Midtrimester Termination of Pregnancy than Laminaria Japonicum
798. Current state and future of IVF
799. Prescribing of ergometrine for abortion without physical examination; [Voorschrijven van ergometrine wegens abortus zonder onderzoek.]
800. Contraception and diabetes mellitus
801. Change in attitudes about illegal abortion; [Panoramawandel illegaler Schwangerschaftsabbrüche.]
802. Prevention of Rh haemolytic disease
803. Antifertility Effect of Continuous Low-dosage Oral Progestogen Therapy
804. Use of prostaglandin for induction of second trimester abortions in high risk pregnancy
805. FACTORS AFFECTING GESTATIONAL AGE AT TERMINATION OF PREGNANCY
806. Evaluation of irreversible contraception; [ZUM STELLENWERT DER IRREVERSIBLEN KONTRAZEPTION]
807. Comparison of intra-amniotic prostaglandin F2αand hypertonic saline for induction of second-trimester abortion
808. Abortion in four Asian countries. Patient characteristics, morbidity, and contraceptive acceptance
809. The gynecologist and the problem of therapeutic abortion; [Le medecin gynecologue face au problème de l'avortement thérapeutique.]
810. Socio-psychological aspects of voluntary abortion at the Obstetrical and Gynecological Clinic of Catania; [Aspetti socio-psicologici dell'aborto volontario nella Clinica Ostetrica e Ginecologica di Catania.]
811. A study of the effects of folklore about the body on IUD use by black American adolescents.
812. A depth study of a married woman; impact of induced abortions on fertility.
813. Do the risks of a diagnostic X-ray during early pregnancy justify therapeutic abortion?; [LES RISQUES D'UN EXAMEN DE RADIO-DIAGNOSTIC AU DEBUT DE LA GESTATION JUSTIFIENT-ILS L'INTERRUPTION DE CELLE-CI?]
814. Male contraception; [LA CONTRACEPTION MASCULINE]
815. Acute Renal Failure Following Hexol-Induced Abortion
816. Researches in aetiology of non-specific urethritis
817. Acute renal failure in obstetrics
818. Clinical Effects of Myotonic Dystrophy on Pregnancy and the Neonate
819. Etiologies and subsequent reproductive performance of 100 couples with recurrent abortion
820. Investigation and treatment of amenorrhoea resulting in normal fertility
821. The use of prostaglandins in obstetrics.
822. Maternal and Neonatal Listeriosis: Report of Case and Brief Review of Literature of Listeriosis in Man
823. Spontaneous foetal losses in women using different contraceptives around the time of conception
824. Abortion attitudes in Trinidad
825. Abortion in Four Asian Countries Patient Characteristics, Morbidity and Contraceptive Acceptance
826. Data for reform of French legislation on abortion; [Eléments pour une réforme de la législation française de l'avortement.]
827. Intrauterine devices in the immediate, early and late postabortion period; [Dispositivos intrauterinos en el postaborto inmediato, precoz y tardío.]
828. The risk of birth defects: Jacobs v. Theimer and parents' right to know
829. Rubella Vaccine in Postpubertal Women: Experience in Western Washington State
830. Massachusetts Medical Society
831. THE DIAGNOSIS OF EARLY PREGNANCY FAILURE BY SONAR
832. Y to X Translocation in a Woman With Reproductive Failure: A New Rearrangement
833. The influence of contraceptive practice upon maternal and child health
834. Medical abortion complications. An epidemiologic study at a mid-Missouri clinic
835. Management of Septic Abortion
836. Correction: Management of Septic Chemical Abortion with Renal Failure (N Engl J Med (1975) 292 (722–725) (10.1056/NEJM197504032921404))
837. The hospital's role in contraception
838. A KAP study on MTP acceptors and their contraceptive practice.
839. Hodgkin’s disease in pregnancy
840. The medical management of incomplete abortion.
841. Hysteroscopic removal of intrauterine contraceptive devices with missing threads
842. Progesterone implantation in habitual abortion
843. Artificial Insemination With Fresh or Frozen Semen: A Comparative Study
844. Abortion law reform in India.
845. Employee benefits: no abortion benefits, no contracts.
846. Ten-minute abortions
847. Medical and Social Aspects of Adolescent Pregnancies: I. Adolescents Applying for Termination of an Illegitimate Pregnancy
848. Hyperosmolar urea for elective midtrimester abortion. Experience in 1,913 cases
849. Therapeutic abortion by Intra-amniotic Injection of Prostaglandins
850. Bacteroidaceae Bacteremia: Effect of Age and Focus of Infection Upon Clinical Course
851. Dermatoglyphics Associated with Fetal Wastage
852. Effects of neonatal intensive care on the mortality rate of idiopathic respiratory distress syndrome; [INFLUENCIA DE LOS CUIDADOS INTENSIVOS NEONATALES EN LA MORTALIDAD DEL SINDROME DE LA DIFICULTAD RESPIRATORIA IDIOPATICA]
853. Fetal Erythrocytes in Maternal Circulation After Spontaneous Abortion
854. Abortion in relation to fetal and maternal welfare
855. Evaluation of a lower dose schedule of intramuscular 15(S)-15-methyl prostaglandin F2 alpha for induction of early midtrimester abortion.
856. The contraceptive practice of abortion patients
857. 74 Requests for interruption of pregnancy after failure of oral contraception; [74 DEMANDES D'INTERRUPTION DE GROSSESSE SUR ECHEC DE LA CONTRACEPTION ORALE; QUELQUES REFLEXIONS]
858. Lupus erythematodes associated with pregnancy (a death case after delivery)
859. Interregional project concerning abortion; [PROJET INTERREGIONAL SUR L'I.V.G.]
860. Intra-amniotic injection of hypertonic solution as a method for termination of pregnancy.
861. Third time unlucky: A study of women who have three or more legal abortions
862. Some psychiatric aspects of abortion
863. Contraception for the teenager
864. The induction of abortion with prostaglandins.
865. The medical management of incomplete abortion
866. Mortality associated with hypertonic saline abortion
867. Uterine Aspiration for Evacuation of the Pregnant Uterus
868. Management of cases of abortion
869. Sexual knowledge and attitudes of adolescents: Relationship to contraceptive use
870. Impact of the supreme court decisions on the performance of abortions in the United States
871. Hypertonic saline-induced abortion complicated by consumptive coagulopathy: A case report
872. Menstrual regulation in family planning services
873. British abortion act.
874. The Immunobiology of Abortion
875. Modern techniques of medical pregnancy termination; [LES TECHNIQUES MODERNES D'INTERRUPTION MEDICALE DE LA GROSSESSE]
876. Pitfalls in the diagnosis of infection following medical termination of pregnancy.
877. Continued clinical experience with an increasing dosage regimen of clomiphene citrate administration
878. Mid-Trimester Abortion by Dilatation and Evacuation: A Safe and Practical Alternative
879. C-FILM: a new local contraceptive.
880. Immediate postabortion intrauterine contraception in nulliparous adolescents
881. Morbidity of therapeutic abortion in Auckland
882. Teenage pregnancy and motherhood: A review of the literature
883. A survey on serious accidents associated with abortion before and after the law of 1975; [Enquete sur les accidents graves de l'avortement avant et apres la loi de 1975.]
884. Transplacental Passage of Fetal Red Cells in Abortion; Increased Incidence after Curettage and Effect of Oxytocic Drugs
885. A Prospective Study of Spontaneous Fetal Losses after Induced Abortions
886. Study of 'spontaneous' abortion in Thailand
887. Choice of analgesia or anesthesia for pain relief in suction curettage

**1.4 Records identified through Web of Science (n=744)**

1. A Study of Incomplete Abortion Following Medical Method of Abortion (MMA).
2. Efficacy of medical abortion prior to 6 gestational weeks: a systematic review
3. Results and lessons learned from a small medical abortion clinical study in Turkey
4. Late-Term Abortion and Medical Necessity: A Failure of Science.
5. Factors related to completeness of medical abortion with mifepristone and misoprostol
6. Elevated mRNA expression of PGF2 receptor splice variant 2(FP-V2) in human decidua is associated with incomplete mifepristone-misoprostol-induced early medical abortion by regulation of interleukin-8
7. Medical management of induced and incomplete first-trimester abortion by non-physicians in low- and middle-income countries: A systematic review and meta-analysis of randomized controlled trials
8. The efficacy of medical abortion: A meta-analysis
9. Integrative transcriptomics and proteomics analyses to reveal the therapeutic effect and mechanism of Buxue Yimu Pills in medical-induced incomplete abortion rats
10. Analysis of pharmacodynamic components, targets and synergistic action mechanism of Fuyuan Shenghua granule for the treatment of medical-induced incomplete abortion based on network pharmacology
11. Misoprostol alone--a new method of medical abortion?
12. Use of mifepristone and sublingual misoprostol for early medical abortion.
13. A Comparative Study of Manual Vacuum Aspiration with Medical Method of Management for Incomplete and Missed Abortion
14. Alternatives to mifepristone for early medical abortion
15. 2017-19 governmental decisions to allow home use of misoprostol for early medical abortion in the UK
16. Medical abortion in family practice: A case series
17. A randomized trial of hospital vs home self administration of vaginal misoprostol for medical abortion.
18. [Antiprogesterone for medical abortion].
19. Determinants and Outcome of Safe Second Trimester Medical Abortion at Jimma University Medical Center, Southwest Ethiopia.
20. Social Questions, Medical Answers: Contesting British Abortion Law
21. Medical abortion in rural Tamil Nadu, South India: A quiet transformation
22. Clinical diagnosis of completeness of medical abortion by nurses: a reliability study in Mozambique
23. Can women accurately assess the outcome of medical abortion based on symptoms alone?
24. Minimal effective dose of mifepristone for medical abortion.
25. Endometrial thickness and serum beta-hCG as predictors of the effectiveness of oral misoprostol in early pregnancy failure.
26. Medical management of missed abortion: A randomized clinical trial
27. Acceptability of home-use of misoprostol in medical abortion
28. First-trimester medical abortion with mifepristone 200 mg and misoprostol: a systematic review
29. The role of parity in medical abortion up to 49 days of amenorrhoea
30. Chemical Fingerprint Analysis and Ultra-Performance Liquid Chromatography Quadrupole Time-of-Flight Mass Spectrometry-Based Metabolomics Study of the Protective Effect of Buxue Yimu Granule in Medical-Induced Incomplete Abortion Rats
31. Does methotrexate confer a significant advantage over misoprostol alone for early medical abortion? A retrospective analysis of 8678 abortions
32. MEDICAL EVACUATION OF 1ST TRIMESTER (12 WEEKS GESTATION) INCOMPLETE ABORTION AND MISSED ABORTION
33. The trophoblastic blood flow during the course of medical abortion
34. The role of auxiliary nurse-midwives and community health volunteers in expanding access to medical abortion in rural Nepal
35. Situation Analysis of Patients Attending TU Teaching Hospital after Medical Abortion with Problems and Complications
36. Provision of medical abortion by midlevel healthcare providers in Kyrgyzstan: testing an intervention to expand safe abortion services to underserved rural and periurban areas
37. Misoprostol as the primary agent for medical abortion in a low-income urban setting
38. A medical record linkage analysis of abortion underreporting
39. Efficacy of misoprostol in relation to uterine position in the treatment of early pregnancy failure
40. Monitoring medical abortion using mifepristone/misoprostol combination with ultrasonogram and serum human chorionic gonadotropin.
41. Can midlevel health-care providers administer early medical abortion as safely and effectively as doctors? A randomised controlled equivalence trial in Nepal
42. Experience of clandestine use of medical abortion among university students in Chile: a qualitative study
43. Effect of Immediate Compared With Delayed Insertion of Etonogestrel Implants on Medical Abortion Efficacy and Repeat Pregnancy
44. [The first 100 early medical abortions].
45. A fatal case of Clostridium sordellii septic shock syndrome associated with medical abortion
46. [Effects of taking estrogen and progestogen after medical abortion on reducing vaginal hemorrhage time: a randomized-controlled trial].
47. An analysis of the cost of incomplete abortion to the public health sector in South Africa - 1994
48. Administration of depot medroxyprogesterone acetate on the day of mifepristone for medical abortion: a pilot study
49. Bacterial Sacroiliitis and Gluteal Abscess After Dilation and Curettage for Incomplete Abortion
50. Factors Associated with Management Outcome of Incomplete Abortion in Yirgalem General Hospital, Sidama Zone, Southern Ethiopia.
51. Intravaginal misoprostol for medical evacuation of first trimester missed abortion.
52. Hydatidiform moles among patients with incomplete abortion in Mwanza City, North western Tanzania
53. A Comparison of the Safety and Efficacy of Three Medical Abortion Protocols
54. Informed consent or institutionalized eugenics? How the medical profession encourages abortion of fetuses with Down syndrome
55. The efficacy of Shenghua Decoction supplementation after early medical abortion: A meta-analysis of randomized controlled trials
56. Mifepristone followed by home administration of buccal misoprostol for medical abortion up to 70 days of amenorrhoea in a general practice in Curacao
57. Efficacy and safety of mifepristone and buccal misoprostol versus buccal misoprostol alone for medical abortion
58. Assessment of completion of early medical abortion using a text questionnaire on mobile phones compared to a self-administered paper questionnaire among women attending four clinics, Cape Town, South Africa
59. Coronary artery vasospasm after misoprostol treatment for incomplete abortion: a case report
60. Telemedicine medical abortion at home under 12 weeks' gestation: a prospective observational cohort study during the COVID-19 pandemic
61. Evaluation of different doses of Femoston therapy for incomplete abortion: A prospective observational trial
62. Replacement of dilation and curettage/evacuation by manual vacuum aspiration and medical abortion, and the introduction of postabortion contraception in Pakistan
63. Management of incomplete spontaneous abortion with suction curettage in the pediatric emergency department
64. Surgical management of early pregnancy failure: history, politics, and safe, cost-effective care
65. Failure to Achieve the Association of Professors in Gynecology and Obstetrics Objectives for Abortion in Third-Year Medical Student Curriculum
66. Comparison of mifepristone plus misoprostol with misoprostol alone for first trimester medical abortion: A systematic review and meta-analysis.
67. Is mifepristone 100mg an effective alternative to standard dose for medical abortion.
68. Evaluation of the success of medical abortion by a plasma hCG control threshold
69. Trend of serum beta-human chorionic gonadotropin levels after medical abortion in the early first trimester of pregnancy
70. Low-sensitivity urine pregnancy testing to assess medical abortion outcome: A systematic review
71. Cervical vasovagal shock: A rare complication of incomplete abortion case report.
72. Abortion policies and practices in Chile: Ambiguities and dilemmas
73. Conflicting recommendations between the French national authority for health (HAS) and clinical practice guidelines (CNGOF); focus on 200 late medical abortions, conducted outside marketing authorizations
74. [Medical claims and women's experience. Physician-performed abortions in the Weimar Republic].
75. The Use of Manual Vacuum Aspiration in the Treatment of Incomplete Abortions: A Descriptive Study from Three Public Hospitals in Malawi
76. Integrating Mobile Phones into Medical Abortion Provision: Intervention Development, Use, and Lessons Learned From a Randomized Controlled Trial
77. Management of incomplete abortion with manual vacuum aspiration in comparison to sharp metallic curette in an ethiopian setting
78. CONTINUATION OF PREGNANCY AFTER MEDICAL ADVICE IN PATIENTS WITH DESIRED ABORTION
79. Telephone follow-up after early medical abortion using Australia's first low sensitivity urine pregnancy test
80. Prophylactic Compared With Therapeutic Ibuprofen Analgesia in First-Trimester Medical Abortion A Randomized Controlled Trial
81. Comparison of two doses of mifepristone in combination with misoprostol for early medical abortion:: a randomised trial
82. [Clinical study of termination of early pregnancy by combination of dl-15-methyl-prostaglandin F2 alpha and RU 486].
83. Induction of abortion in the second trimester of pregnancy by intramuscular 15-methyl prostaglandin F 2 alpha (Prostin 15M).
84. ABORTION AT THE 1ST TRIMESTER OF PREGNANCY - THE RISKS OF RESPONSIBILITY FROM JURISPRUDENCE AND THE STUDY OF 81 COMPLAINTS
85. Effectiveness of the FIGO protocol for medical management of first-trimester abortion
86. Effects of leonurine hydrochloride on medically induced incomplete abortion in early pregnancy rats
87. Is self-assessment of medical abortion using a low-sensitivity pregnancy test combined with a checklist and phone text messages feasible in South African primary healthcare settings? A randomized trial
88. Medical and Social Problems and Attitudes to Optional Abortions in Bulgaria
89. WHO multinational study of three misoprostol regimens after mifepristone for early medical abortion. I: Efficacy
90. UNSUCCESSFUL PRIOR ATTEMPTS TO TERMINATE PREGNANCY AMONG WOMEN SEEKING FIRST TRIMESTER ABORTION AT REGISTERED FACILITIES IN BIHAR AND JHARKHAND, INDIA
91. Medical abortion at 57 to 63 days' gestation with a lower dose of mifepristone and gemeprost -: A randomized controlled trial
92. IS WARD EVACUATION FOR UNCOMPLICATED INCOMPLETE ABORTION UNDER SYSTEMIC ANALGESIA SAFE AND EFFECTIVE - A RANDOMIZED CLINICAL-TRIAL
93. Evaluating women's acceptability of treatment of incomplete second trimester abortion using misoprostol provided by midwives compared with physicians: a mixed methods study
94. Controversias en el tratamiento del aborto incompleto: AMEU versus tratamiento médico con misoprostol
95. [Change in attitudes about illegal abortion].
96. Effectiveness of early medical abortion using low-dose mifepristone and buccal misoprostol in women with no defined intrauterine gestational sac
97. [Clinical study of early pregnancy termination by administration of DL-15-methyl prostaglandin F2 alpha combined with tamoxifen or norethisterone].
98. Reproductive failure due to spontaneous abortion and recurrent miscarriage
99. Survey of the attitude to, the knowledge and the practice of contraception and medical abortion in women who attended a family planning clinic.
100. Failure of sterilization after clip placement
101. A comparison of the costs of manual vacuum aspiration (MVA) and evacuation and curettage (E and C) in the treatment of early incomplete abortions in Kenya.
102. Outcomes During Early Implementation of Mifepristone-Buccal Misoprostol Abortions up to 63 Days of Gestation in a Canadian Clinical Setting.
103. Primary health care, access to legal abortion and the notion of ideal victim among medical practitioners: The case of Chile
104. Clinical observation on termination of early pregnancy of 213 cases after caesarian section with repeated use of mifepristone and misoprostol.
105. Comparative effectiveness, safety and acceptability of medical abortion at home and in a clinic: a systematic review
106. Management of incomplete and missed spontaneous abortions: a cohort study of trends in Calgary emergency departments
107. Effect of Wujia Shenghua capsule on uterine bleeding following medically-induced incomplete abortion in rats during early pregnancy
108. MEDICAL ABORTION IN WOMEN OF LESS-THAN-OR-EQUAL-TO-56 DAYS AMENORRHEA - A COMPARISON BETWEEN GEMEPROST (A PGE(1) ANALOG) ALONE AND MIFEPRISTONE AND GEMEPROST
109. INDUCTION OF ABORTION WITH MIFEPRISTONE AND MISOPROSTOL IN EARLY-PREGNANCY
110. [Clinical study of four cases with malignant gestation trophoblastic tumor after mifepristone abortion].
111. Assessment of efficacy and safety of medical treatment of non-viable first trimester pregnancy
112. Fetal complications associated with unsuccessful attempts at termination of pregnancy
113. [Factors associated with the failure of medical treatment for ectopic pregnancy: case study conducted at the Yaounde Gynaecology, Obstetrics and Pediatrics Hospital].
114. ASPECTS OF THE SIGNIFICANCE OF OPERATIVELY CORRECTED HEART-VALVE FAILURE ON FERTILITY
115. Choice of analgesia or anesthesia for pain relief in suction curettage.
116. MIDGESTATIONAL ABORTION FOR MEDICAL OR GENETIC INDICATIONS
117. Analysis of Complications and Management After Self-Administration of Medical Termination of Pregnancy Pills.
118. Termination of early pregnancy using flexible, low-dose mifepristone-misoprostol regimens
119. [Medically-induced abortion and risk of reproductive failures in subsequent pregnancy].
120. High failure rates of medical termination of pregnancy after introduction to a large teaching hospital
121. ACUTE-RENAL-FAILURE IN PREGNANCY - 1987
122. A foreign body in the cervix after spontaneous abortion: a rare case of a traumatic fetal decapitation.
123. Sonographic quantification of endometrial changes after abortion with computer-assisted image analysis
124. Experience with midtrimester abortion.
125. ACUTE-RENAL-FAILURE IN BLACKS AND INDIANS IN SOUTH-AFRICA - COMPARISON AFTER 10 YEARS
126. Successful treatment of Placenta Percreta through a combinatorial treatment involving a Bakri Balloon and Methotrexate - a case report
127. Understanding of changes in abortion rate following liberalization of abortion law among Brazilian residents in obstetrics and gynecology
128. [Acute renal insufficiency in pregnancy and the postpartum period].
129. First Trimester Bleeding
130. FATAL SEPTIC ABORTION IN THE UNITED-STATES, 1975-1977
131. Association of the G2014G Genotype in Estrogen Receptor 1 Gene with Failure of the Mifepristone-Induced Termination of Early Pregnancy
132. First-trimester spontaneous abortions and the incidence of human immunodeficiency virus seropositivity
133. RISKS AND COSTS OF ILLEGALLY INDUCED-ABORTION IN BANGLADESH
134. The medical management of incomplete abortion.
135. The midwife's role in abortion care.
136. The clinical management of abortion.
137. Contraceptive failure rates: New estimates from the 1995 National Survey of Family Growth
138. Safety of induced abortions at less than 12 weeks of pregnancy in Japan
139. Prevalence of morbidity associated with abortion before and after legalisation in South Africa
140. Exploring the costs and economic consequences of unsafe abortion in Mexico City before legalisation
141. Achievements of the FIGO Initiative for the Prevention of Unsafe Abortion and its Consequences in South-Southeast Asia
142. Early pregnancy failure - Current management concepts
143. Ectopic pregnancy in the era of medical abortion: are we ready for it? Spectrum of sonographic findings and our experience in a tertiary care service hospital of India.
144. Catastrophic antiphospholipid syndrome during pregnancy and puerperium:: maternal and fetal characteristics of 15 cases
145. RU486: Misconceptions, myths and morals. A critique of the criticisms.
146. A pilot study of mifepristone and misoprostol administered at the same time for abortion up to 49 days gestation
147. Protective Effect of Taohong Siwu Decoction on Abnormal Uterine Bleeding Induced by Incomplete Medical Abortion in Rats during Early Pregnancy
148. HYSTEROSCOPIC FINDINGS AFTER MISSED ABORTION
149. [Termination of early pregnancy using RU 486 alone or in combination with prostaglandin. The RU 486 Collaboration Group].
150. Misoprostol use in obstetrics and gynecology in Brazil, Jamaica, and the United States
151. Success rate of second-trimester termination of pregnancy using misoprostol
152. Study of spontaneous abortion in Thailand.
153. Alteration of TH1 and TH2 cells by intracellular cytokine detection in patients with unexplained recurrent abortion before and after immunotherapy with the husband's mononuclear cells
154. ABORTION - AN ATTITUDE STUDY OF PROFESSIONAL STAFF AT RAMATHIBODI-HOSPITAL
155. Induced abortion -: Physician training and practice patterns
156. DESIGN OPTIONS AND METHODOLOGICAL FALLACIES IN THE STUDIES OF REPRODUCTIVE FAILURES
157. Accessing abortion in a highly restrictive legal regime: characteristics of women and pregnant people in Malta self-managing their abortion through online telemedicine
158. Presenting features of women with uterine arteriovenous malformations
159. Outbreaks of porcine reproductive failure: Report on a collaborative field investigation
160. An overview of unsafe abortion in Africa. Foreword.
161. Maternal level of pregnancy-associated plasma protein A as a predictor of pregnancy failure in threatened abortion.
162. COST-EFFECTIVENESS OF MANAGING ABORTIONS - MANUAL VACUUM ASPIRATION (MVA) COMPARED TO EVACUATION BY CURETTAGE IN TANZANIA
163. A PILOT-STUDY OF ACCEPTABILITY OF RU486 AND ONO-802 IN A CHINESE POPULATION
164. The gag rule revisited: physicians as abortion gatekeepers.
165. The outcome of in vitro fertilization in unexplained habitual aborters concurrent with secondary infertility
166. Manual vacuum aspiration saves lives.
167. Abortion policy implementation in Ireland: Lessons from the community model of care
168. Change in serum beta-human chorionic gonadotropin after abortion with methotrexate and misoprostol
169. A medical method of early pregnancy termination using tamoxifen and misoprostol
170. Septic Abortion: A 5-Year Experience at Siriraj Hospital
171. Mifepristone-misoprostol abortion: a trial in rural and urban Maharashtra, India
172. Comparative Study of Mifepristone with Vaginal Misoprostol for First Trimester Termination of Pregnancy at Different Gestational Ages.
173. Complications of misoprostol and other abortion induction methods in the developing world: A systematic review
174. Ultrasonographic characteristics in patients clinically diagnosed with threatened abortion
175. Study results translate to better services.
176. Legal duties to respect abortion choices.
177. Manual vacuum aspiration technique draws interest.
178. Misoprostol-Induced Fever and Unnecessary Antibiotic Prescribing: A Retrospective Study
179. Buxue Yimu Pills improve angiogenesis and blood flow in experimental zebrafish and rat models
180. Methotrexate and misoprostol used alone or in combination for early abortion
181. Triangular Assessment of the Etiology of Induced Abortion in Iran: A Qtualitative Study
182. OPTIONS IN MAKING USE OF PREGNANCY HISTORY IN PLANNING AND ANALYZING STUDIES OF REPRODUCTIVE FAILURE
183. Clinical pattern of gynecological/early pregnancy complaints and the outcome of pelvic sonography in a private diagnostic center in Ilorin
184. TERMINATION OF EARLY-PREGNANCY BY 2 REGIMENS OF MIFEPRISTONE WITH MISOPROSTOL AND MIFEPRISTONE WITH PG05 - A MULTICENTER RANDOMIZED CLINICAL-TRIAL IN CHINA
185. A study of psychiatric morbidity during second trimester of pregnancy subsequent to abortion in the previous pregnancy.
186. Abortion procedures in a tertiary care institution in India
187. Maternal mortality and morbidity associated with clandestine abortions.
188. THE FEASIBILITY OF EARLY 2ND TRIMESTER PREGNANCY (12 TO 14 WEEKS) TERMINATION WITH SINGLE INTRAAMNIOTIC INJECTION OF 15-METHYL PGF2-ALPHA
189. Ten years on: a review of medical terminations of pregnancy performed in a sexual health clinic
190. [Interregional project concerning abortion].
191. Potential use of single measurement of serum progesterone in detecting early pregnancy failure.
192. [Value of hysteroscopy and laparoscopy in differential diagnosis of gestational trophoblastic neoplasia].
193. Systemic inflammatory response syndrome, organ failure, and outcome in critically ill obstetric patients treated in an ICU
194. Reproductive health information and abortion services: Standards developed by the European Court of Human Rights
195. Secular trends in uncertain-sex births and proportion of male births in Norway, 1967-1998
196. Implication of sperm chromosomal abnormalities in recurrent abortion and multiple implantation failure
197. The influence of abortion legislation on maternal mortality
198. Medical termination of pregnancy in the second trimester
199. Retrospective analysis of prognostic value of the neutrophil-to-lymphocyte ratio in early miscarriages A 8-year survey
200. Complicated illegal induced abortions at a tertiary health institution in Nigeria
201. Bleeding patterns after vaginal misoprostol for treatment of early pregnancy failure
202. Abortion in today's world.
203. Comprehensive abortion care: evidence of improvements in hospital-level indicators in Tigray, Ethiopia
204. Pre-implantation genetic screening among women experiencing recurrent failure of in vitro fertilization
205. TERMINATION OF PREGNANCY IN PAPUA-NEW-GUINEA - THE TRADITIONAL AND CONTEMPORARY POSITION
206. Women's Perspectives on Ultrasound Viewing in the Abortion Care Context
207. The Feasibility of a Modified Method of Laparoscopic Transabdominal Cervicoisthmic Cerclage During Pregnancy
208. CRANIORACHISCHISIS WITH INCOMPLETE TWINS DIPROSOPUS - REPORT OF A CASE AND REVIEW OF THE LITERATURE
209. Is Sex-Selective Abortion against the Law?
210. Orientame:: Preventing and solving problems related to unwanted pregnancy for 25 years in Colombia
211. Unsafe abortion: an avoidable tragedy
212. Post-abortion care: Ethical and legal duties
213. A STUDY OF 500 CASES OF ACUTE-RENAL-FAILURE (1978-1891)
214. I will never wish this pain to even my worst enemy: Lived experiences of pain associated with manual vacuum aspiration during post-abortion care in Kenya
215. Abortion education in Canadian family medicine residency programs
216. An examination of loss to follow-up and potential bias in outcome ascertainment in a study of direct-to-patient telemedicine abortion in the United States
217. A pilot study on the use of a 7-day course of letrozole followed by misoprostol for the termination of early pregnancy up to 63 days
218. Is therapeutic abortion preventable?
219. Latin American hospitals improve postabortion care. Maternal health.
220. Lowering the doses of mifepristone and gemeprost for early abortion:: a randomised controlled trial
221. Lowering the doses of mifepristone and gameprost for early abortion: a randomised controlled trial. World Health Organization Task Force on Post-ovulatory Methods for Fertility Regulation.
222. Medical abortion complications. An epidemiologic study at a mid-Missouri clinic.
223. [74 requests for interruption of pregnancy after failure of oral contraception (author's transl)].
224. Morbidity of therapeutic abortion in Auckland.
225. Abortion at Gondar College Hospital, Ethiopia.
226. A randomised comparative study on sublingual versus vaginal administration of misoprostol for termination of pregnancy between 13 to 20 weeks
227. Women's Medical Center of Providence v. Roberts.
228. Motives for self-induced abortion among women in Alexandria: An exploratory study.
229. Early abortion with buccal versus sublingual misoprostol alone: a multicenter, randomized trial
230. Abortion in four Asian countries: patient characteristics, morbidity, and contraceptive acceptance.
231. Mifepristone: ten years later
232. FETAL DEATH RATIOS IN A PROSPECTIVE-STUDY COMPARED TO STATE FETAL DEATH CERTIFICATE REPORTING
233. Fatal necrotizing fasciitis in illegal abortion and the negligence tort
234. SEPTIC ABORTION
235. Youth often risk unsafe abortions.
236. Association between uterine position and transvaginal misoprostol treatment for early pregnancy failure
237. Contraceptive use among women seeking repeat abortion in Addis Ababa, Ethiopia.
238. PROSTAGLANDIN ANALOGS AND THEIR USES
239. A thoroughly regressive law.
240. [Physicians confront abortion].
241. ABORTION POLICY AND WOMENS HEALTH IN DEVELOPING-COUNTRIES
242. Cervical Twin Heterotopic Pregnancy: Overview of Ectopic Pregnancies and Scanning Detection Algorithm
243. The outcome of septic abortion: a tertiary care hospital experience.
244. THE EFFECT OF SMOKING ON REPRODUCTIVE FAILURES IN COUPLES EXAMINED IN THE GENETIC OUTPATIENT CLINIC AT DR. A. JURASZ UNIVERSITY HOSPITAL IN BYDGOSZCZ
245. Abortion, Informed Consent, and Regulatory Spillover
246. The psychosocial factors of the abortion experience: a critical review.
247. Cytogenetic studies of couples with reproductive failure in alexandria, egypt.
248. Suggestions on Revision Regarding Abortion Laws in Korea: an Empirical Study Using Qualitative Research Method
249. A short-scheme protocol of gemeprost for midtrimester termination of pregnancy with uterine scar
250. The determinants and health consequences of unsafe abortion in rio de janeiro, brazil
251. Effectiveness and safety of early medication abortion provided in pharmacies by auxiliary nurse-midwives: A non-inferiority study in Nepal
252. A MUTATION IN THE MECHANISMS OF SOCIAL-CONTROL - THE CASE OF ABORTION
253. Willing and unable: Doctors' constraints in abortion care
254. Complications of unsafe abortion: A case study and the need for abortion law reform in Nigeria
255. [Post-abortion counseling].
256. [Improving the quality of care after spontaneous abortions in rural Senegal].
257. The association between history of retained placenta and success rate of misoprostol treatment for early pregnancy failure
258. Abortion Training at Multiple Sites: An Unexpected Curriculum for Teaching Systems-Based Practice
259. INDUCTION OF ABORTION DURING THE 2ND TRIMENON OF PREGNANCY - ENDOCERVICAL PG-E2 GEL APPLICATION, INTRAMUSCULAR APPLICATION OF SULPROSTON AND COMBINED TREATMENT (ENDOCERVICAL PG-E2 GEL INTRAMUSCULAR SULPROSTON)
260. Illegal abortion with misoprostol in Guadeloupe
261. Future uncertain as Congressional drive to block Title X gag rule fails.
262. EXPERIENCES WITH GEMEPROST IN 1ST-TRIMESTER TERMINATION OF PREGNANCY
263. [Induced abortion. Legislation, epidemiology, complications].
264. Contraceptive risk-taking and contraceptive failure among users of female barrier methods
265. MALARIA IN PREGNANCY - CLINICAL-FEATURES AND OUTCOME OF TREATMENT
266. Post legalisation challenge: minimizing complications of abortion.
267. Estimating the costs for the treatment of abortion complications in two public referral hospitals: a cross-sectional study in Ouagadougou, Burkina Faso
268. Protecting safe abortion in humanitarian settings: overcoming legal and policy barriers
269. Angiographic transcatheter embolization in gynaecological practice
270. Contraception: from accessibility to efficiency
271. Soluble MHC Class I chain-related molecule serum levels are predictive markers of implantation failure and successful term pregnancies following IVF
272. Criminality.
273. LATE MIDTRIMESTER MEDICAL PREGNANCY TERMINATIONS - 3 DIFFERENT PROCEDURES WITH PROSTAGLANDIN-F2-ALPHA AND LAMINARIA TENTS
274. Cervical pregnancy: A rare case of reimplantation after abortion. A case report
275. Abortion, breast cancer, and informed consent
276. Age, parity, history of abortion and contraceptive choices affect the risk of repeat abortion
277. [The gynecologist and the problem of therapeutic abortion].
278. A depth study of a married woman; impact of induced abortions on fertility.
279. Role of Ultrasonography in the Evaluation of Causes of Bleeding Per Vaginum in the First Trimester of Pregnancy
280. Pregnancy in Wilson's disease: Management and outcome
281. Effectiveness of Self-Managed Medication Abortion Between 9 and 16 Weeks of Gestation
282. USE OF QUININE FOR SELF-INDUCED ABORTION
283. Right tool, wrong job: Manual vacuum aspiration, post-abortion care and transnational population politics in Senegal
284. The Culture of Illegal Abortion in South Africa
285. Factors influencing the delivery of abortion services in Ontario: A descriptive study
286. Infertility today: The management of female medical causes
287. Efficacy, Safety, and Acceptability of Misoprostol in the Treatment of Incomplete Miscarriage: A Systematic Review and Meta-analysis.
288. Acupuncture benefits to women with recurrent implantation failure: A propensity score-matched cohort study
289. Ultrasonographic evaluation of endometrial changes using computer assisted image analysis
290. [A survey on serious accidents associated with abortion before and after the law of 1975].
291. [Voluntary interruption of pregnancy: not an insignificant act].
292. Cervical ectopic pregnancy - Diagnosis with endovaginal ultrasound examination and successful treatment with methotrexate
293. Identifying National Availability of Abortion Care and Distance From Major US Cities: Systematic Online Search
294. Misoprostol for termination of second trimester pregnancy in a scarred uterus
295. Factors Associated with Severe Complications in Unsafe Abortion
296. ABORTIONS IN A HOSPITAL SETTING - HIDDEN REALITIES IN DAR-ES-SALAAM, TANZANIA
297. BIRTH-CONTROL AND SOCIAL GROUP - CONTRACEPTION, ACCIDENTAL PREGNANCIES AND ABORTION
298. Setting up a medical abortion service for failure
299. Evaluation of a lower dose schedule of intramuscular 15(S)-15-methyl prostaglandin F2 alpha for induction of early midtrimester abortion.
300. Comparison of the effects of voluntary termination of pregnancy and uterine evacuation for medical reasons on female sexual function
301. A comparison of the efficacy of sublingual and oral misoprostol 400 microgram in the management of early pregnancy failure: a randomized controlled trial.
302. CLINICAL-TRIAL ON TERMINATION OF EARLY-PREGNANCY WITH RU486 IN COMBINATION WITH PROSTAGLANDIN
303. Hyperosmolar urea for elective midtrimester abortion. Experience in 1,913 cases.
304. Abortion practices in high school students in Yamoussoukro, Cote d'Ivoire
305. ABOUT - ANALYSIS OF AND REFLECTIONS ON SOME ABORTION REQUEST STATISTIC
306. TISSUE DISTRIBUTION OF COCAINE IN A PREGNANT WOMAN
307. OBSERVATIONS ON ABORTION IN ZAMBIA
308. Predictive value of peripheral blood α1-acid glycoprotein in medical abortion outcomes with mifepristone and relativity of concentration
309. Doppler velocimetry of the uterine arteries: an early screening test for miscarriage.
310. Opinion and use of contraceptives among medical students of the University of Nigeria, Enugu campus
311. THE CONTRIBUTION OF ENVIRONMENTAL TERATOGENS TO EMBRYONIC AND FETAL LOSS
312. Management of maternal Amanita phalloides poisoning during the first trimester of pregnancy:: A case report and review of the literature
313. Delayed hemolysis, elevated liver enzymes, low platelet count syndrome in succession of switches of preventive anticoagulant treatment in a 41-year-old patient with a history of recurrent assisted implantation failures: a case report.
314. Misoprostol-induced Acute Coronary Syndrome in a Premenopausal Woman: A Case Report with Literature Review.
315. Who will do the abortions?
316. CONCURRENT COPPER-T INSERTION WITH MEDICAL TERMINATION OF PREGNANCY IN WOMEN WITH PREVIOUS CESAREAN-SECTION DELIVERY
317. Acute renal failure in obstetric patients: treatment by hemodialysis.
318. SEXUAL-ACTIVITY IN GIRLS UNDER 16 YEARS OF AGE
319. Recours à l'avortement provoqué à Lomé (Togo): évolution, facteurs associés et perceptions
320. Lown-Ganong-Levine Syndrome in a 3-Month-Old Infant with Isolated Left Ventricular Noncompaction
321. Cardiac surgical experience in northern Nigeria
322. ESTIMATES OF DEMAND FOR ABORTION AMONG SOVIET IMMIGRANTS IN ISRAEL
323. MEDICAL METHODS TO TERMINATE EARLY-PREGNANCY
324. CHARACTERISTICS OF WOMEN HAVING ABORTION IN CHINA
325. Medical termination of pregnancy and concurrent contraceptive adoption in a tertiary referral hospital in Delhi.
326. [Trends in the indications for termination of pregnancy in the last 10 years in Switzerland].
327. Midtrimester abortion with intramuscular injection of 15-methyl-prostaglandin F2alpha.
328. The impact of the COVID-19 pandemic on the course of miscarriages.
329. Comparison of vaginal and sublingual misoprostol for second trimester abortion: randomized controlled equivalence trial
330. Pregnancy and lupus nephritis.
331. Setting up a medical abortion service for failure Reply
332. Study on Clinical Presentation and Outcome of Septic Abortion and Its Relationship with Person Inducing Abortion.
333. Increased risk for medical abortion failure for multiparous women
334. THE INDUCTION OF ABORTION IN THE 2ND TRIMESTER BY COMBINED ADMINISTRATION OF MINPROSTIN AND SULPROSTON, COMPARED WITH THE USE OF SULPROSTON ALONE
335. A comparison of misoprostol with and without laminaria tents for induction of second-trimester abortion
336. Placental polyp:: An unusual hysteroscopic finding
337. Role of Ultrasonography in Evaluation of Bleeding Per Vagina in First Trimester of Pregnancy
338. MODERN INDICATIONS FOR ABORTION
339. Health benefits of legal abortion: an analysis.
340. Medical student awareness of sexual health is poor
341. A randomized comparative study on vaginal administration of acetic acid-moistened versus dry misoprostol for mid-trimester pregnancy termination
342. Characteristics of medical termination of pregnancy acceptors in Pondicherry State, 1972-1976.
343. Vaginal PGE2 and intraamniotic PGF2 alpha for the termination of second trimester pregnancy.
344. [Data for reform of French legislation on abortion].
345. [Termination of early pregnancy by two regimens of mifepristone with misoprostol: a multicentre clinical trial].
346. AMNIOCENTESIS BEFORE 15 WEEKS GESTATION - TECHNICAL ASPECTS AND OBSTETRIC RISKS
347. Outcome of pregnancy in Takayasu arteritis
348. Methotrexate compared with mercaptopurine for early induced abortion
349. The induction of abortion with prostaglandins.
350. Morbidity that is associated with curettage for the management of spontaneous and induced abortion in women who are infected with HIV
351. Psychosocial aspects of induced abortion
352. WOMENS REPRODUCTIVE HEALTH
353. Serum biomarkers may help predict successful misoprostol management of early pregnancy failure
354. Hysteroscopic Diagnosis of Omentum Incarceration Subsequent to an Iatrogenic Uterine Perforation
355. Clandestine abortion in Port Harcourt: users' profile and motivation.
356. Experiences of midwives and nurses when implementing abortion policies: A systematic integrative review
357. [Comparison of clinical outcomes of vitrified-thawed embryo transfer and fresh embryos transfer].
358. Pathophysiology of mifepristone-induced septic shock due to Clostridium sordellii
359. Acute renal failure in pregnancy in a developing country: Twenty years of experience
360. The characteristics of and the contraceptive practice among women seeking therapeutic termination of pregnancy in the Scottish Highlands.
361. The frequency of chromosomal, abnormalities in patients with reproductive failure
362. BRAZIL INVESTIGATES DRUGS POSSIBLE LINK WITH BIRTH-DEFECTS
363. Umbilical cord lesions in early intrauterine fetal demise
364. [A phase III multicentre study on medical termination of early pregnancy with two regimens of mifepristone followed by PG05].
365. Silent uterine rupture with the use of misoprostol for second trimester termination of pregnancy : a case report.
366. [Moebius syndrome due to the use of misoprostol. Case report].
367. Induced Abortion: Incidence and Trends Worldwide From 1995 to 2008
368. Continued clinical experience with an increasing dosage regimen of clomiphene citrate administration.
369. NON-DEVELOPING PREGNANCY: HISTOLOGICAL AND IMMUNOHISTOCHEMICAL MARKERS OF ENDOCRINE DISORDERS IN ENDOMETRIAL SCRAPES
370. INDUCED-ABORTION IN KENYA - CASE-HISTORIES
371. INDUCED-ABORTION AMONG UNMARRIED WOMEN IN SICHUAN PROVINCE, CHINA
372. Determinants of abortion among women admitted to hospitals in Fortaleza, North Eastern Brazil
373. Are serum progesterone levels predictive of recurrent miscarriage in future pregnancies?
374. Contraception for the teenager.
375. Manual vacuum aspiration: a safe and cost-effective substitute of Electric vacuum aspiration for the surgical management of early pregnancy loss
376. Protective effect of breastfeeding against childhood leukemia in Zhejiang Province, P. R. China: a retrospective case-control study
377. [Socio-psychological aspects of voluntary abortion at the Obstetrical and Gynecological Clinic of Catania].
378. Restrictive State Abortion Bans-A Reproductive Injustice
379. The pattern of maternal mortality at maternity hospital Kuala Lumpur.
380. Crisis Pregnancy Centers in the US: Lack of Adherence to Medical and Ethical Practice Standards A Joint Position Statement of the Society for Adolescent Health and Medicine and the North American Society for Pediatric and Adolescent Gynecology Society for Adolescent Health and Medicine and the North American Society for Pediatric and Adolescent Gynecology
381. Mifepristone and misoprostol administered simultaneously versus 24 hours apart for abortion - A Randomized controlled trial
382. How family planning can save lives in Africa.
383. Crisis Pregnancy Centers in the U.S.: Lack of Adherence to Medical and Ethical Practice Standards: A Joint Position Statement of the Society for Adolescent Health and Medicine and the North American Society for Pediatric and Adolescent Gynecology.
384. Crisis Pregnancy Centers in the United States: Lack of Adherence to Medical and Ethical Practice Standards; A Joint Position Statement of the Society for Adolescent Health and Medicine and the North American Society for Pediatric and Adolescent Gynecology
385. Pitfalls in the diagnosis of infection following medical termination of pregnancy.
386. PREGNANCY AFTER HEART-VALVE REPLACEMENT
387. [APF. Open letter to the political parties, on the abortion situation in Portugal].
388. MIFEPRISTONE IN MID-TRIMESTER TERMINATION OF PREGNANCY - VALUE FOR MONEY
389. Cleft lip and palate: series of unusual clinical cases
390. Launch, Drift, and Withdrawal of Public Policy: A Case Study of the Korean Medical School System
391. Efficacy of Single Dose Vaginal Misoprostol 800μg in i Trimester Abortion
392. Abortion.
393. [The experience of carrying out the technical responsibility system in the birth control of Peng County, Sichuan Province].
394. Successful medical abortion of a pregnancy within a noncommunicating rudimentary uterine horn
395. Cesarean Scar Ectopic Pregnancy: The Lurking Danger in Post Cesarean Failed Medical Abortion.
396. [The applied of computer for analysis on contraceptive efficacy of IUD of rural women in Guangdong province].
397. Contraceptive coverage after medical termination of pregnancy.
398. SPANISH ABORTION REFORM FACES OPPOSITION
399. Public Law No. 262, 25 March 1988.
400. Pregnancy, fertility, and recurrence risk in corrected tetralogy of Fallot
401. Progesterone-mediated reversal of mifepristone-induced pregnancy termination in a rat model: an exploratory investigation
402. MUST A CATHOLIC HOSPITAL INFORM A RAPE VICTIM OF THE AVAILABILITY OF THE MORNING-AFTER PILL
403. Post abortion syndrome: myth or reality?
404. ABORTION - EPIDEMIOLOGY, SAFETY, AND TECHNIQUE
405. A Stalled Revolution? Misoprostol and the Pharmaceuticalization of Reproductive Health in Francophone Africa.
406. Controversy surrounds use of test for open spina bifida.
407. The abortion battle: the Canadian scene.
408. Choice at any cost.
409. [Intrauterine devices in the immediate, early and late postabortion period].
410. [Demand for abortion. Special aspects of drug-induced abortion].
411. The legal implications of prenatal diagnosis in Malaysia.
412. Introduction: menstrual regulation - the method and the issues.
413. PSYCHOLOGICAL SEQUELAE OF INDUCED-ABORTION
414. A successful twin pregnancy in a patient with HbE-β-thalassemia in western India
415. INDUCTION OF ABORTION IN EARLY-PREGNANCY WITH MIFEPRISTONE IN CONJUNCTION WITH GEMEPROST
416. Diagnosis of aneuploidy in arrival, paraffin-embedded pregnancy-loss tissues by comparative genomic hybridization
417. [Contraception].
418. Decision making on unsafe abortions in Sri Lanka: a case-control study
419. [Analysis of the results and considerations on the organizational aspects of the implementation of the Law 194/78 in a provincial hospital].
420. [Use of misoprostol in gynecology and obstetrics].
421. OBSTETRIC AND GYNECOLOGIC DYSFUNCTION IN THE EHLERS-DANLOS SYNDROME
422. UNUSUAL INTRAUTERINE OBJECTS - POTENTIAL PITFALLS IN ULTRASONOGRAPHIC IDENTIFICATION
423. Infectious agents identified in aborted swine fetuses in a high-density breeding area: a three-year study
424. Randomized study on the effect of adding oxytocin to ethacridine lactate or misoprostol for second-trimester termination of pregnancy
425. Pregnancy outcome after suicide attempt by drug use: a Danish population-based study
426. COPING WITH FERTILITY IN ISRAEL - A CASE-STUDY OF CULTURE CLASH
427. A STATISTICAL ANALYSIS OF WOMEN'S REPRODUCTIVE HEALTH CHARACTERISTICS AFTER INEFFECTIVE REATTEMPTS OF USING ART.
428. Family planning in Europe.
429. Comparison of Outcomes before and after Ohio's Law Mandating Use of the FDA-Approved Protocol for Medication Abortion: A Retrospective Cohort Study
430. Menstrual induction with mifepristone and misoprostol
431. SEQUELAE OF PREMATURE SEXUAL LIFE
432. [Induced abortion: a vulnerable public health problem].
433. GESTATIONAL TROPHOBLASTIC DISEASE - TREATMENT RESULTS AT THE BREWER TROPHOBLASTIC DISEASE CENTER
434. CONTRACEPTIVE PRACTICES OF WOMEN ATTENDING FOR TERMINATION OF PREGNANCY - A STUDY FROM SOUTH AUSTRALIA
435. [Induced abortion. Epidemiological study after eight years of enforcement of Law 194].
436. Integrating gender perspectives in gynecology and obstetrics: Engaging medical colleges in Maharashtra, India
437. Compliance with new ban means no mention of abortion.
438. Estimation of the adolescent pregnancy rate in Thailand 2008-2013: an application of capture-recapture method
439. THE CLINICAL MANAGEMENT OF REPEATED EARLY-PREGNANCY WASTAGE
440. Uterine artery embolization in the treatment and prevention of postpartum hemorrhage
441. CURRENT MANAGEMENT OF MOLAR PREGNANCY
442. Role of Ultrasound in First Trimester Bleeding
443. Gastrojejunostomy as successful surgical treatment of acute duodenitis/proximal jejunitis in a mare
444. Provision of abortion by mid-level providers: international policy, practice and perspectives
445. Incidence of diandric triploidy (partial mole) in misscarriages
446. Comparitive Study of Early (24 Hours) Versus Late (48 Hours) Misoprostol Administration After Mifepristone for Termination of Early Pregnancy
447. Legal Limits Relaxed: Time to Look at Other Barriers Faced by Women Seeking Termination of Pregnancy for Fetal Anomalies.
448. What is needed to ensure the health and survival of mother and baby?
449. FATAL HEMORRHAGE FROM LEGAL-ABORTION IN THE UNITED-STATES
450. A safe and effective method of termination of mid-trimester pregnancy.
451. Validation of ICD-9 Codes for Stable Miscarriage in the Emergency Department.
452. Assessing post-abortion care in health facilities in Afghanistan: a cross-sectional study
453. PROSTAGLANDIN-E2 INDUCTION OF ABORTION AND FETAL DEMISE
454. A retrospective comparison between lamicel and gemeprost for cervical ripening before surgical interruption of first-trimester pregnancy
455. ADVERSE PREGNANCY OUTCOME AND CHILDHOOD MALIGNANCY WITH REFERENCE TO PATERNAL WELDING EXPOSURE
456. The Language of First-Trimester Nonviable Pregnancy Patient-Reported Preferences and Clarity
457. Office Management of Early Pregnancy Loss
458. RECENT DEVELOPMENTS OF MEDICAL LAW
459. INDUCED-ABORTION IN MATERNAL AND CHILD HEALTH CENTERS IN A GENERAL FAMILY-PLANNING PROGRAM IN CAP BON, TUNISIA
460. The profile of women who seek emergency contraception from the family planning service
461. Interrupção da gestação por opção na Póvoa de Varzim: análise e perspectivas
462. Design of a syringe extension device (Chloe SED) for low-resource settings in sub-Saharan Africa: a circular economy approach.
463. Constraints and optimization of the laser microwelding process of thin metal foils
464. Maternal mortality at the University of Benin Teaching Hospital Benin City, Nigeria.
465. RISK MANAGEMENT IN PREGNANCY TERMINATION
466. Pregnancy in Pateints With Exstrophy-Epispadias Complex: Are Higher Rates of Complications and Spontaneous Abortion Inevitable?
467. The risk of birth defects: Jacobs v. Theimer and parents' right to know.
468. Comparison of obstetric emergency clinical readiness: A cross-sectional analysis of hospitals in Amhara, Ethiopia
469. [Complications of induced abortions].
470. New York appeals court finds state not liable for death after physician under review performed abortion.
471. Early versus late misoprostol administration after mifepristone for medical abortion
472. Estudios citogenéticos en pacientes con fallas reproductivas. Pinar del Río, 2015-2020
473. Problems loom ahead.
474. EFFECTIVENESS OF THE DIAPHRAGM, USED CONTINUOUSLY, WITHOUT SPERMICIDE
475. [Medical and socio-demographic implications of abortion at Felix Bulnes Hospital in Santiago (author's transl)].
476. Does congenital heart disease severely jeopardise family life and pregnancies? Obstetrical history of women with congenital heart disease in a single tertiary centre
477. Implantable cardioverter-defibrillators and pregnancy - A safe combination?
478. Immediate postabortion intrauterine contraception in nulliparous adolescents.
479. A comparison of pregnancy outcomes after second-trimester amniocentesis between cases with penetration of the placenta and nonpenetration
480. Assessing survival time of outpatients with cervical cancer: at the university of Gondar referral hospital using the Bayesian approach
481. Not every subseptate uterus requires surgical correction to reduce poor reproductive outcome
482. Leptospirosis seropositivity and its serovars among cattle in Northeastern Malaysia.
483. The morphology, typical combinations of polymorphic genes of hemostasis, and specific features of the pathogenesis of retrochorial hematoma in missed abortion
484. Experience and acceptability of emergency hormonal contraception.
485. [Contraceptive compliance - why is contraceptive failure still so frequent?].
486. Experts, non -experts, and policy discourse: A case study of the Royal Commission on New Reproductive Technologies
487. REPRODUCTIVE PATTERNS AS ENVIRONMENTAL MARKERS IN RURAL MOROCCO
488. [A family planning center: role of the nurse].
489. Second- and third-trimester termination of pregnancy in women with uterine scar - a retrospective analysis of 111 gemeprost-induced terminations of pregnancy after previous cesarean delivery
490. The relationship between insecurity and the quality of hospital care provided to women with abortion-related complications in the Democratic Republic of Congo: A cross-sectional analysis
491. PREGNANCY AND SYSTEMIC LUPUS-ERYTHEMATOSUS
492. THE PREGNANT PLASTIC SURGICAL RESIDENT - RESULTS OF A SURVEY OF WOMEN PLASTIC SURGEONS AND PLASTIC-SURGERY RESIDENCY DIRECTORS
493. Evaluation of the Incidence and Outcome of Gestational Diabetes Mellitus Using the Current International Consensus Guidelines for Diagnosing Hyperglycaemia in Pregnancy
494. Impact of prolonged use of adjuvant tocolytics after cervical cerclage on late abortion and premature delivery
495. Evaluation of the Incidence and Outcome of Gestational Diabetes Mellitus Using the Current International Consensus Guidelines for Diagnosing Hyperglycaemia in Pregnancy
496. The impact of the prenatal ultrasonography on birth of babies with Korean pediatric surgical index diseases
497. MEDICAL-MANAGEMENT OF MISCARRIAGE - NONSURGICAL UTERINE EVACUATION OF INCOMPLETE AND INEVITABLE SPONTANEOUS-ABORTION (VOL 306, PG 894, 1993)
498. Construction and validation of a spatial database of providers of transgender hormone therapy in the US
499. [Prescribing of ergometrine for abortion without physical examination].
500. Abortion in Brazil: what do the official data say?
501. When is emergency contraception the right name for postcoital treatment?
502. Bleeding after dilatation and curettage: the efficacy of transcatheter uterine artery embolisation
503. Complications during pregnancy and delivery in women with untreated rectovaginal deep infiltrating endometriosis
504. A case of Morbius syndrome in a newborn baby after medical elective abortion failure
505. Aborto no Brasil: o que dizem os dados oficiais?
506. SEXUAL KNOWLEDGE AND ATTITUDES OF ADOLESCENTS - RELATIONSHIP TO CONTRACEPTIVE USE
507. DIAPHRAGM METHOD CONTRACEPTORS - IMPLICATIONS FOR SERVICE ORGANIZATION AND DELIVERY
508. Azzolino v. Dingfelder.
509. A Study of the Prevalence of Reproductive Tract Infections and its Associated Factors among Married Women of Age 15 to 49 Years in an Adopted PHC of Adichunchanagiri Institute of Medical Sciences, B.G. Nagara, Mandya
510. How antiabortion legislation would put doctors in a bind.
511. Honour and interests: Medical ethics in Britain, and the work of the British Medical Association's Central Ethical Committee, 1902-1939
512. The Cost of Child Rearing for Wrongful Conception
513. Pericentric Inversion of Human Chromosome 9 Epidemiology Study in Czech Males and Females
514. Honour and interests medical ethics in Britain and the work of the British Medical Association's Central Ethical Committee, 1902-1939. (BL: DXN050009)
515. Structural equation modelling analysis determining causal role among methyltransferases, methylation, and apoptosis during human pregnancy and abortion
516. Hysteroscopic removal of intrauterine contraceptive devices with missing threads.
517. Risk of complications during pregnancy in women with congenital aortic stenosis
518. Pregnancy outcomes in multiple sclerosis patients previously treated with cyclophosphamide
519. [STD prevention and unplanned pregnancies in Great Britain].
520. Pregnancy Rates and Outcomes of HIV-Infected Women in Korea
521. Estimates of pregnancies averted through California's family planning waiver program in 2002
522. Homocysteine serum concentration and uterine artery color Doppler examination in cases of recurrent miscarriages with unexplained etiology
523. Status epilepticus in pregnancy: a literature review and a protocol proposal
524. Management of pregnancy and delivery in women with transposition of the great arteries after atrial switch operation: A 16-year single-center experience
525. Fertility and pregnancy outcomes following uterine devascularization for severe postpartum haemorrhage
526. Risk factors for intrauterine device embedment in postmenopausal women: an analysis of 731 participants undergoing hysteroscopy
527. Menstrual regulation in family planning services.
528. EFFECTIVENESS OF GENETIC CONSULTATION AND GENETIC PREVENTION
529. California may become first state to mandate insurance coverage for contraception.
530. [Sex education: prerequisite for family planning].
531. C-FILM: a new local contraceptive.
532. Obstetric and perinatal outcome of teenage pregnancy.
533. [Contraceptive efficacy].
534. Clinical course of hepatitis B virus infection during pregnancy
535. COST-EFFECTIVENESS OF LEVONORGESTREL SUBDERMAL IMPLANTS - COMPARISON WITH OTHER CONTRACEPTIVE METHODS AVAILABLE IN THE UNITED-STATES
536. CURRENT MANAGEMENT OF COMPLETE AND PARTIAL MOLAR PREGNANCY
537. Of human rights and women's health.
538. Study of pregnancy outcome over a period of five years in a postgraduate institute of west Bengal.
539. THE EFFECT OF DOSE OF MIFEPRISTONE AND GESTATION ON THE EFFICACY OF MEDICAL ABORTION WITH MIFEPRISTONE AND MISOPROSTOL
540. Pérdida recurrente del embarazo e hipertensión pulmonar idiopática
541. STRATEGIES FOR NURSING UTERINE RUPTURE IN PRIMIPARAS DURING PREGNANCY
542. Maternal and fetal outcomes of subsequent pregnancies in women with peripartum cardiomyopathy.
543. Placental abnormalities in equine pregnancies generated by SCNT from one donor horse
544. The unwanted child.
545. Practices and knowledge of female gynecologists regarding contraceptive use: a real-world Chinese survey
546. Is preimplantation genetic diagnosis the ideal embryo selection method in aneuploidy screening?
547. Effectiveness of Jian-Pi-An-Tai formula for the pregnancy outcome of in vitro fertilization and embryo transfer in infertile women: Protocol of a randomized controlled trial
548. Matsubara-Yano suture: a simple uterine compression suture for postpartum hemorrhage during cesarean section
549. Secondary infertility and the aging male, overview.
550. Kisspeptin: A Potential Factor for Unexplained Infertility and Impaired Embryo Implantation.
551. TERMINATION OF 2ND TRIMESTER PREGNANCY WITH SULPROSTONE AND MIFEPRISTONE - A RANDOMIZED DOUBLE-BLIND PLACEBO-CONTROLLED TRIAL
552. Clinton reform proposal faces Congressional test on covered services, cost.
553. Survey women educational needs about routine OCPs
554. PREGNANCY OUTCOME IN WOMEN EXPOSED TO DIETHYLSTILBESTROL INUTERO
555. Clinical manifestations of pregnancy in patients with Takayasu arteritis: experience from a single tertiary center
556. Cuban health system -- call it superior?
557. Putting the ICPD Plan of Action to work. Turkey. Women's health tops the agenda.
558. Family planning clinics share blame for men's failure to use services.
559. [Termination of pregnancy in the 2nd trimester using intra-amniotic administration of prostaglandins].
560. Epidemiology of spontaneous pregnancy loss in Kazakhstan: A national population-based cohort analysis during 2014-2019 using the national electronic healthcare system
561. Ectopic pregnancy: a life-threatening gynecological emergency.
562. Uterine rupture during pregnancy and delivery: risk factors, symptoms and maternal and neonatal outcomes - restrospective cohort.
563. Success rate of methotrexate treatment for recurrent vs. primary ectopic pregnancy: a case-control study
564. Maternal mortality in hospitals in Zululand, July 1993 June 1994
565. Parsing the Q-Markers of Baoyin Jian to Treat Abnormal Uterine Bleeding by High-Throughput Chinmedomics Strategy
566. Exposure to radiation therapy is associated with female reproductive health among childhood cancer survivors: a meta-analysis study
567. Ethics and reproductive health: A principled approach
568. Decision making by single women seeking abortion.
569. Five years experience of haemodialysis at the Lagos University Teaching Hospital--November 1981 to November 1986.
570. Different types of serologic reactivity to Bedsonia (psittacosis group) antigen in various hosts. Discussion of some related problems.
571. Pregnancies, growth and development of children conceived by subzonal injection of spermatozoa
572. Representations and uses of emergency contraception in West Africa. A social anthropological reading of a northern medicinal product
573. Pattern of congenital heart disease among Egyptian children: a 3-year retrospective study.
574. Cognitive Bias in the Management of a Critically Ill 29-Year-Old Patient.
575. [Desire for pregnancy, refusal of pregnancy, ambivalence in family planning].
576. Misconceptions: Monstrosity and the politics of interpretation in American culture from the Antinomian Controversy to biotechnology
577. Personal accounts of 'near-miss' maternal mortalities in Kampala, Uganda
578. Clinical Significance of Sub Chorionic and Retroplacental Hematomas Detected in the First Trimester of Pregnancy
579. Adult high-risk Burkitt's acute lymphocytic leukemia was successfully rescued by rituximab combined with hyper-CVAD/MA regimens: two case reports and a literature review
580. Enduring politics: the culture of obstacles in legislating for assisted reproduction technologies in Ireland.
581. Contraception Utilization in Black Women via a Reproductive Justice Lens
582. Septic pelvic thrombophlebitis of unknown origin: an ever threatening entity.
583. Quality focuses on clients' needs.
584. COMPARISON OF ADOLESCENT AND ADULT EXPERIENCES WITH NORPLANT LEVONORGESTREL CONTRACEPTIVE IMPLANTS
585. The perimenopause and contraception
586. I did not plan to have a baby. This is the outcome of our work: a qualitative study exploring unintended pregnancy among female sex workers
587. Use of platelet rich plasma in human infertility.
588. POSTCOITAL CONTRACEPTION - SOME CHARACTERISTICS OF WOMEN WHO USE THIS METHOD
589. Early Surface Ablation on Aborted LASIK Flaps
590. CANDIDA SEPSIS IN PREGNANCY AND THE POSTPARTUM PERIOD
591. Maternal mortality at the University of Nigeria Teaching Hospital, Enugu: a 10-year survey.
592. Maternal mortality in a semi-urban Nigerian community.
593. Tanzanian lessons in using non-physician clinicians to scale up comprehensive emergency obstetric care in remote and rural areas
594. Four perspectives of women's health. Workshop participants talk about women's health issues in four countries. [Hong Kong].
595. Pregnancy Rates and Outcomes of HIV-Infected Women in Korea
596. MIFEPRISTONE (RU-486) COMPARED WITH HIGH-DOSE ESTROGEN AND PROGESTOGEN FOR EMERGENCY POSTCOITAL CONTRACEPTION
597. [Pregnancy and cardiac surgery with cardiopulmonary bypass].
598. CONTRACEPTIVE PRACTICE OF ABORTION PATIENTS
599. (Un)Knowing Women: Sexuality, Pregnancy, and Knowledge Production in Modernist Literature
600. Replacing the dead: The politics of reproduction in the postwar Soviet Union, 1944–1955
601. Abortamento Espontâneo – Um Estudo Epidemiológico
602. Laparoscopic tubal sterilization coincident with therapeutic abortion by suction curettage.
603. Postcoital contraception: a report from the London Brook Advisory Centres, United Kingdom.
604. Medical Developments and Religious Belief with Special Reference to Europe in the 18th and 19th Centuries
605. Postcoital contraception: a delicate political issue.
606. [Sterilization as a method of family planning].
607. Service Delivery Correlates of Choosing Short-Acting Contraceptives at the Time of Uterine Evacuation in Bangladesh
608. Prenatal Risk Factors for Optic Nerve Hypoplasia
609. [Food contaminated by metals. A study of health consequences in a limited region].
610. Effect of Pregnancy in Arrhythmogenic Right Ventricular Cardiomyopathy
611. A novel method for office aspiration curettage in cases of retained products of conception: A randomized controlled trial
612. Maternal and fetal prognosis of subsequent pregnancy in black African women with peripartum cardiomyopathy
613. Perampanel and pregnancy
614. [Male contraception].
615. Antiphospholipid syndrome
616. Use of misoprostol during pregnancy and Mobius syndrome in infants
617. Malaria in pregnancy.
618. AN ACCOUNT OF 2180 CASES OF PERCOELIOSCOPIC TUBAL-LIGATION USING YOON MECHANICAL METHOD
619. Treatment of epilepsy in women of childbearing age.
620. Direct Effects of Mifepristone on Mice Embryogenesis: An In Vitro Evaluation by Single-Embryo RNA Sequencing Analysis
621. EVALUATING WELL-WOMAN CLINICS
622. ETHICAL ISSUES IN PRENATAL-DIAGNOSIS
623. College women's experience with emergency contraception
624. Maternal activity in relation to birth size in rural India. The Pune Maternal Nutrition Study
625. How safe motherhood in India is.
626. The Value of Posterior Cervical Angle as a Predictor of Vaginal Delivery: A Preliminary Study
627. Long-term Outcome of Lupus Nephritis: A Single Center Study.
628. The effect of hysteroscopic metroplasty on fertility in T-shaped and Y-shaped dysmorphic uterus
629. The theory and practice of self -ownership
630. Maternal Fontan procedure is a predictor of a small-for-gestational-age neonate: a 10-year retrospective study
631. Providing accessible medical abortion services in a Victorian rural community: A description and audit of service delivery and contraception follow up
632. KNOWLEDGE AND USE OF SECONDARY CONTRACEPTION AMONG PATIENTS REQUESTING TERMINATION OF PREGNANCY
633. EMERGENCY CONTRACEPTIVE PILLS - A SIMPLE PROPOSAL TO REDUCE UNINTENDED PREGNANCIES
634. Deformidad del principio de autonomía para sustentar actos éticamente ilícitos
635. VASECTOMY ON REQUEST - STILL A MOOT POINT FOR DISCUSSION
636. Life and death issues in bioethics: Abortion, persistent vegetative state, and the definition of death
637. Cardiovascular disease and associated comorbid conditions as determinants of adverse perinatal outcomes in pregnancy - an analysis of the results of the register of pregnant BEREG
638. Effect of folic acid supplementation on diminished ovarian reserve: study protocol of a single-centre, open-label, randomised, placebo-controlled clinical trial
639. Economic cost of pregnancy care attributable to the failure of Mexico's teenage pregnancy prevention policy
640. A Comprehensive Study of Jaundice in Pregnancy with Emphasis on Fetomaternal Outcome
641. SEXUALITY, WORDS AND FAMILY-PLANNING
642. Clinical experience with intramuscular norethisterone oenanthate as a contraceptive.
643. Ghana.
644. Ulipristal acetate and pregnancy outcome-an observational study
645. [Psychological decision making with regard to motherhood by women with high-risk pregnancy and normal pregnancy].
646. Characteristics of maternal cardiac disease and pregnancy outcomes: results from a 4-year observational cohort survey in Central Vietnam
647. A STUDY OF THE EFFECTS OF FOLKLORE ABOUT THE BODY ON IUD USE BY BLACK-AMERICAN ADOLESCENTS
648. THE PROBLEM OF TEENAGE PREGNANCY
649. Pregnancy Outcome in Women with a History of Congenital Heart Disease: a Case Series Study
650. Congenital heart disease and acquired valvular lesions in pregnancy
651. Garrison v. Medical Center of Delaware, Inc., 12 December 1989.
652. Reproductive health crisis during waves one and two of the COVID-19 pandemic in India: Incidence and deaths from severe maternal complications in more than 202,000 hospital births
653. RU 486 (MIFEPRISTONE) - FROM CLINICAL-TRIALS TO THE PERSPECTIVES FOR CLINICAL USE
654. T-HELPER 1-TYPE IMMUNITY TO TROPHOBLAST IN WOMEN WITH RECURRENT SPONTANEOUS-ABORTION
655. [Maternal and fetal outcomes with aortic dissection in pregnant patients with Marfan syndrome].
656. Pregnancy toxemia and lipid mobilization syndrome in two alpaca (Vicugna pacos) at 6 and 10 months of gestation
657. Medical treatment for missed abortion- difference in gestational age based on menstrual dating and on ultrasound criteria, and the correlation to failure
658. Medical abortion outcomes after a second-dose of misoprostol: Is the presence of cardiac motion on follow-up ultrasonography predictive of failure?
659. Misoprostol for women's health: A review
660. Síndrome de HELLP en el Hospital Nacional Guillermo Almenara Irigoyen: Presentación clínica y complicaciones de una emergencia obstétrica
661. Encefalocele occipital gigante sin complicaciones neonatales inmediatas
662. Drug interactions between oral contraceptives and antibiotics
663. Genetic findings in early miscarriage analysis by Chromosomal Microarray and Whole Exome Sequencing
664. Medical Management of Induced and Incomplete First-Trimester Abortion by Non-physicians in Low- and Middle-Income Countries: A Systematic Review and Meta-analysis of Randomized Controlled Trials
665. Obstetric determinants of neonatal survival: Antenatal predictors of neonatal survival and morbidity in extremely low birth weight infants
666. Bio-artificial liver from cultured human foetal hepatocytes: feasibility and prospects.
667. Eisenmenger syndrome - Factors relating to deterioration and death
668. THE DIFFICULTIES OF CONTRACEPTION - CONFLICTS AND PARADOXES
669. ETHICS AND MEDICAL DECISION-MAKING
670. ROCKING THE CRADLE: THE UNCERTAIN POLICY OF WITHHOLDING TREATMENT FROM ANOMALOUS NEWBORNS (INFANTICIDE, MERCY-KILLING, DEFECTIVE INFANTS, SELECTIVE NON-TREATMENT, EUTHANASIA)
671. Homocysteine, folic acid and vitamin B12 concentration in patientswith recurrent miscarriages
672. [Chorionic villus needle sampling by the transabdominal route or by placental centesis. A series of 930 cases].
673. MISH publishes new framework for fear-based, abstinence-only education.
674. Teen pregnancy: effect on family well-being.
675. Characterisation of the molecular basis underlying chlamydial subversion of focal adhesion signaling
676. PREGNANCY TERMINATION - TECHNIQUES, RISKS, AND COMPLICATIONS AND THEIR MANAGEMENT
677. Decision-making and choice about child-bearing: a case study of deferred motherhood
678. Curettage and Asherman's syndrome-lessons to (re-) learn?
679. ON INHUMAN PRACTICES IN GYNECOLOGY AND THEIR VICTIMS IN GERMANY DURING NATIONAL-SOCIALIST RULE - A STUDY OF CONCRETE EVENTS
680. Maternal heart disease and pregnancy outcome: A single
681. Eficacia y seguridad del taponamiento uterino para control de hemorragia y disminución de histerectomía obstétrica: Cohorte histórica en Nuevo León, México, 2013
682. Cost-effectiveness of emergency contraception options over 1 year
683. AUTOANTIBODIES IN NORMAL AND ABNORMAL PREGNANCY
684. [Ovarian function in systemic lupus erythematosus patients undergoing the use of cyclophosphamide in two major rheumatologic care centers in Curitiba, Parana State].
685. Comparing pregnancy outcomes between symptomatic and asymptomatic COVID-19 positive unvaccinated women: Multicenter study in Saudi Arabia
686. [The value of irreversible contraception].
687. Evaluation of Anticoagulant Use during Pregnancy in Mothers with Antiphospholipid Syndrome: An Observational Cross-Sectional Study
688. Targeted client communication via mobile devices for improving sexual and reproductive health
689. Case Study on the Wrongful Birth Action
690. FORENSIC PROBLEMS IN BOVINE OBSTETRICS AND GYNECOLOGY
691. ATTITUDES TO CONTRACEPTION IN WOMEN SEEKING TERMINATION OF PREGNANCY
692. Study of side-effects of Cu-T as intra-uterine contraceptive device in post medical termination of pregnancy and interval cases.
693. [Medical genetics in reproductive medicine].
694. Maternal and Perinatal Outcome of Maternal Obesity at RSCM in 2014-2019.
695. Study of the Relation Between Preterm Delivery and Periodontitis the Role of Prophylactic Dental Therapy in the Prevention of Preterm Delivery
696. Evaluation of Competitive ELISA For Detection of Antibodies to Rift Valley Fever Virus in Cattle and Sheep Sera
697. RESTRICTIVE LUNG-DISEASE IN PREGNANCY
698. The effects of religious beliefs on the health care practices of the Amish.
699. Impacto del estrés laboral en el anestesiólogo
700. Observations on female sterilization in Chile.
701. Acute Kidney Injury in Asia
702. Planned Parenthood continues prescribing birth control for teens.
703. Comprehensive Genetic Analysis of Pregnancy Loss by Chromosomal Microarrays: Outcomes, Benefits, and Challenges
704. Sífilis congénita: auditorías en tiempo real
705. The Montana Fetal Genetic Pathology Program and a review of prenatal death in humans.
706. Male experiences of unintended pregnancy: characteristics and prevalence
707. Laparoscopic sterilisation: Opinion and practice among gynaecologists in Scotland
708. Causes of perinatal mortality. Report of a study of 4,429 cases.
709. Uptake and Outcome of Artificial Reproductive Techniques Following Allogeneic Stem Cell Tranplantation: A Single Centre Experience
710. LAPAROSCOPIC TUBAL-STERILIZATION USING YOONS RINGS - THE TECHNIQUE AND THE PSYCHOLOGICAL EFFECTS
711. [Human rights, maternal mortality and reproductive health].
712. FERTILITY TRENDS IN SINGAPORE
713. Status of male contraception.
714. CONTOURS OF DEVELOPMENT
715. Fertility and Pregnancy Outcomes in Females with Dyskeratosis Congenita
716. Vaccination against influenza in pregnant women - safety and effectiveness
717. Developmental and reproductive risks of radiological procedures utilizing ionizing radiation during pregnancy
718. The Position of General Practitioner Midwifery.
719. Evolución cardiológica postnatal y factores asociados a la agenesia de ductus venoso de diagnóstico prenatal
720. Formulation and noncontraceptive uses of the new, low-dose oral contraceptive.
721. Pregnancy in patients with prosthetic and homograft heart valves - maternal and fetal outcome
722. [Contraception in adolescence: knowing is not enough].
723. Outcome of Noac Exposure during Pregnancy (... and the problem of event reporting ...)
724. Changing trends in maternity care in Sri Lanka.
725. Predictors of Pregnancy Outcome in Essential Thrombocythemia: A Single Institution Study of 63 Pregnancies
726. Afro-Caribbean Children in Local Authority Day Care 0 - 3
727. Pregnancy Complications In Sickle Cell Disease Are More Prevalent In Women with Vaso-Occlusion Related Organ Damage Than Hemolysis Related Organ Damage
728. Phase III clinical trial with Norplant II (two covered rods): report on five years of use.
729. Chronic Myeloid Leukemia: Comparison of Survival between Pregnant and Non-Pregnant Women
730. Comparative Study of Transdermal Nitroglycerine Patch and Oral Nifedipine in Preterm Labour
731. Maternal and infant follow-up after total cervical occlusion
732. Seroprevalence of Babesia caballi and Theileria equi in horses in Central Germany
733. The portrayal and perceptions of cesarean section in Mexican media Facebook pages: a mixed-methods study
734. RENDERING FOLLICLE TUBES PERMEABLE AGAIN AFTER POSTPARTUM STERILIZATION - A REPORT ON 25 CASES, SHOULD POSTPARTUM STERILIZATION BE PERFORMED OR NOT
735. Intrauterine administration of hCG immediately after oocyte retrieval and the outcome of ICSI: a randomized controlled trial
736. Exhaustion in Myeloid Lineage and Very Early Defect in HSPC Pool: An Embryonic Origin of Fanconi Haematological Disorders
737. Disfunção Tiroideia em Grávidas de Alto e Baixo Risco Numa Região com Défice de Iodo (Beira Interior)
738. Sperm Proteins As Targets for Contraception and Fertility Biomarkers
739. [The fetal adrenal gland in risk pregnancy].
740. Prevalence of unprofessional social media content among young vascular surgeons (Retracted article. See vol. 72, pg. 1514, 2020)
741. DETERMINANTS OF LOW BIRTH-WEIGHT - METHODOLOGICAL ASSESSMENT AND META-ANALYSIS
742. Canadian Contraception Consensus (Part 1 of 4).
743. Canadian Contraception Consensus (Part 2 of 4).
744. Observations on trypanosomiasis in the Belgian Congo.

**1.5 Records identified through Cochrane Library (n=9)**

1. Cleavage-stage versus blastocyst-stage embryo transfer in assisted reproductive technology
2. Intracytoplasmic sperm injection versus conventional in vitro fertilisation in couples with males presenting with normal total sperm count and motility
3. Culture media for human pre-implantation embryos in assisted reproductive technology cycles
4. Antioxidants for male subfertility
5. Growth hormone for in vitro fertilisation (IVF)
6. Targeted client communication via mobile devices for improving sexual and reproductive health
7. Gonadotrophins for ovulation induction in women with polycystic ovary syndrome
8. Endometrial preparation for women undergoing embryo transfer with frozen embryos or embryos derived from donor oocytes
9. Anticonvulsant therapy for status epilepticus

**2. Records after duplicates removed by EndNote (n=1690)**

1. "criminality"
2. "i will never wish this pain to even my worst enemy" lived experiences of pain associated with manual vacuum aspiration during postabortion care in kenya
3. "reproductive health care," the "demographic imperative," and the real health needs of women in the developing world (part one)
4. "right tool," wrong "job" manual vacuum aspiration, postabortion care and transnational population politics in senegal
5. "ru486 misconceptions, myths and morals" a critique of the criticisms
6. (un)knowing women sexuality, pregnancy, and knowledge production in modernist literature
7. [74 requests for interruption of pregnancy after failure of oral contraception (author's transl)]
8. [a family planning center role of the nurse]
9. [a phase iii multicentre study on medical termination of early pregnancy with two regimens of mifepristone followed by pg05]
10. [a survey on serious accidents associated with abortion before and after the law of 1975]
11. [abortion law in france]
12. [acute renal insufficiency in pregnancy and the postpartum period]
13. [ambulatory medical abortion performed in a family planning center]
14. [analysis of the results and considerations on the organizational aspects of the implementation of the law 194/78 in a provincial hospital]
15. [antiprogesterone for medical abortion]
16. [apf open letter to the political parties, on the abortion situation in portugal]
17. [cervical pregnancy a rare case of reimplantation after abortion a case report]
18. [change in attitudes about illegal abortion]
19. [chorionic villus needle sampling by the transabdominal route or by placental centesis a series of 930 cases]
20. [clinical study of early pregnancy termination by administration of dl15methyl prostaglandin f2 alpha combined with tamoxifen or norethisterone]
21. [clinical study of four cases with malignant gestation trophoblastic tumor after mifepristone abortion]
22. [clinical study of terminating biochemical pregnancy and early clinical pregnancy with mifepristone and misoprostol]
23. [clinical study of termination of early pregnancy by combination of dl15methylprostaglandin f2 alpha and ru 486]
24. [clinical study on 39 cases with caesarean scar pregnancy with sonographic mass]
25. [comparison of clinical outcomes of vitrifiedthawed embryo transfer and fresh embryos transfer]
26. [complications of induced abortions]
27. [conflicting recommendations between the french national authority for health (has) and clinical practice guidelines (cngof); focus on 200 late medical abortions, conducted outside marketing authorizations]
28. [contraception in adolescence knowing is not enough]
29. [contraception]
30. [contraceptive compliance why is contraceptive failure still so frequent?]
31. [contraceptive efficacy]
32. [craniorachischisis in conjoined "diprosopus" twins case report and review of the literature]
33. [data for reform of french legislation on abortion]
34. [demand for abortion special aspects of druginduced abortion]
35. [desire for pregnancy, refusal of pregnancy, ambivalence in family planning]
36. [effects of taking estrogen and progestogen after medical abortion on reducing vaginal hemorrhage time a randomizedcontrolled trial]
37. [evaluation of the success of medical abortion by a plasma hcg control threshold]
38. [experiences with the antigestagen mifepristone (ru 486) in the interruption of early pregnancy]
39. [factors associated with the failure of medical treatment for ectopic pregnancy case study conducted at the yaounde gynaecology, obstetrics and pediatrics hospital]
40. [food contaminated by metals a study of health consequences in a limited region]
41. [human rights, maternal mortality and reproductive health]
42. [if you were to have another abortion, would you choose the same method? a study on 1032 patients' level of satisfaction]
43. [improving the quality of care after spontaneous abortions in rural senegal]
44. [induced abortion epidemiological study after eight years of enforcement of law 194]
45. [induced abortion legislation, epidemiology, complications]
46. [induced abortion a vulnerable public health problem]
47. [interregional project concerning abortion]
48. [intrauterine devices in the immediate, early and late postabortion period]
49. [large variation in clinical regimens use to induce medical abortion in denmark]
50. [male contraception]
51. [management of pheochromocytoma during pregnancy about three cases]
52. [maternal and fetal outcomes with aortic dissection in pregnant patients with marfan syndrome]
53. [medical abortion at home and at hospital a trial of efficacy and acceptability]
54. [medical abortion for firsttrimester termination of the pregnancy]
55. [medical abortion provided by telemedicine to women in latin america complications and their treatment]
56. [medical abortion using methotrexate and misoprostol efficacy and tolerability]
57. [medical and sociodemographic implications of abortion at felix bulnes hospital in santiago (author's transl)]
58. [medical claims and women's experience physicianperformed abortions in the weimar republic]
59. [medical genetics in reproductive medicine]
60. [medical termination of pregnancy with mifepristoneinitial experience at the sheba medical center]
61. [medicallyinduced abortion and risk of reproductive failures in subsequent pregnancy]
62. [mifepristone 100 mg for early medical abortion]
63. [misoprostol offlabel use in the first trimester of pregnancy (spontaneous abortion, and voluntary medical termination of pregnancy)]
64. [moebius syndrome due to the use of misoprostol case report]
65. [nonsurgical female sterilization using quinacrine efficacy of two insertions of quinacrine pellets]
66. [ovarian function in systemic lupus erythematosus patients undergoing the use of cyclophosphamide in two major rheumatologic care centers in curitiba, parana state]
67. [physicians confront abortion]
68. [postabortion counseling]
69. [pregnancy and cardiac surgery with cardiopulmonary bypass]
70. [prescribing of ergometrine for abortion without physical examination]
71. [psychological decision making with regard to motherhood by women with highrisk pregnancy and normal pregnancy]
72. [retrospective assessment of the influence of gestational age on the first trimester medical abortion efficiency]
73. [sex education prerequisite for family planning]
74. [should antiprogesterone be used in pregnancy termination?]
75. [side effects of induced abortion results from a populationbased survey]
76. [sociopsychological aspects of voluntary abortion at the obstetrical and gynecological clinic of catania]
77. [std prevention and unplanned pregnancies in great britain]
78. [sterilization as a method of family planning]
79. [study on leuprorelin acetate in treatment of uterine adenomyosis with infertility]
80. [termination of early pregnancy by two regimens of mifepristone with misoprostol a multicentre clinical trial]
81. [termination of early pregnancy using ru 486 alone or in combination with prostaglandin the ru 486 collaboration group]
82. [termination of pregnancy in the 2nd trimester using intraamniotic administration of prostaglandins]
83. [the applied of computer for analysis on contraceptive efficacy of iud of rural women in guangdong province]
84. [the efficacy and acceptability of mifepristone medical abortion with home administration misoprostol provided by private providers linked with the hospital a prospective study of 433 patients]
85. [the experience of carrying out the technical responsibility system in the birth control of peng county, sichuan province]
86. [the fetal adrenal gland in risk pregnancy]
87. [the fetal adrenal gland in risk pregnancy]; [nadbubrezne zlezde fetusa u rizicnim trudnoćama]
88. [the first 100 early medical abortions]
89. [the gynecologist and the problem of therapeutic abortion]
90. [the value of irreversible contraception]
91. [trends in the indications for termination of pregnancy in the last 10 years in switzerland]
92. [update in current care guidelines induced abortion]
93. [use of misoprostol in gynecology and obstetrics]
94. [value of hysteroscopy and laparoscopy in differential diagnosis of gestational trophoblastic neoplasia]
95. [voluntary interruption of pregnancy not an insignificant act]
96. ‘…a one stop shop in their own community’ medical abortion and the role of general practice
97. ‘a hope raised and then defeated’? the continuing harms of irish abortion law
98. “i did not plan to have a baby this is the outcome of our work” a qualitative study exploring unintended pregnancy among female sex workers
99. “nothing more or less than a discharged convict” the career of dr thomas millerchip of coventry, 18741912
100. …a one stop shop in their own community' medical abortion and the role of general practice
101. 10year evaluation of the use of medical abortion through telemedicine a retrospective cohort study
102. 200 mg mifepristone was as effective as 600 mg when used with oral misoprostol for early medical abortion
103. 2017 naf am abstracts
104. 201719 governmental decisions to allow home use of misoprostol for early medical abortion in the uk
105. 346 efficacy and safety of mifepristone and sublingual misoprostol for second trimester medical abortion a prospective study
106. 5 embryo manipulation and experimentation
107. 74 requests for interruption of pregnancy after failure of oral contraception; [74 demandes d'interruption de grossesse sur echec de la contraception orale; quelques reflexions]
108. a case of fetal methotrexate syndromea rare cause of multiple congenital anomalies
109. a case of morbius syndrome in a newborn baby after medical elective abortion failure
110. a classic case of toxic shock syndrome due to a not so classic organism, clostridium sordellii
111. a clinical evaluation of the safety and efficacy of mefepristone and misoprostol in medical abortions
112. a comparative study of manual vacuum aspiration with medical method of management for incomplete and missed abortion
113. a comparative study on sublingual versus oral and vaginal administration of misoprostol for late first and early second trimester abortion
114. a comparison of medical abortion (using mifepristone and gemeprost) with surgical vacuum aspiration efficacy and early medical sequelae
115. a comparison of medical and surgical management of first trimester incomplete miscarriages a randomised control trial in pmgh
116. a comparison of misoprostol with and without laminaria tents for induction of secondtrimester abortion
117. a comparison of misoprostol with and without methylergometrine and oxytocin in outpatient medical abortion a phase iii randomized controlled trial
118. a comparison of pregnancy outcomes after secondtrimester amniocentesis between cases with penetration of the placenta and nonpenetration
119. a comparison of surgical vacuum aspiration abortion with medical abortion using mifepristone (ru 486) and gemeprost implications for nursing staff
120. a comparison of the costs of manual vacuum aspiration (mva) and evacuation and curettage (e and c) in the treatment of early incomplete abortions in kenya
121. a comparison of the efficacy of sublingual and oral misoprostol 400 microgram in the management of early pregnancy failure a randomized controlled trial
122. a comparison of the safety and efficacy of three medical abortion protocols
123. a comparison of the safety and efficacy of two medical abortion protocols
124. a comparitive study of use of intravaginal misoprostol after 48 hours and 4 hours of oral mifepristone for medical termination of pregnancy
125. a comprehensive study of jaundice in pregnancy with emphasis on fetomaternal outcome
126. a depth study of a married woman; impact of induced abortions on fertility
127. a directtopatient telemedicine abortion service in australia retrospective analysis of the first 18 months
128. a family planning center role of the nurse; [un centre de planification familiale place et rôle de l'infirmière]
129. a fatal case of clostridium sordellii septic shock syndrome associated with medical abortion
130. a few concerns about bioethics; [quelques préoccupations au sujet de la bioéthique]
131. a foreign body in the cervix after spontaneous abortion a rare case of a traumatic fetal decapitation
132. a kap study on mtp acceptors and their contraceptive practice
133. a maternal death from selfinduced medical abortion a call for action
134. a medical method of early pregnancy termination using tamoxifen and misoprostol
135. a medical record linkage analysis of abortion underreporting
136. a method of abortion and a danger to the woman's health how young poles perceive hormonal postcoital contraception; ["metoda aborcji i zagrożenie zdrowia kobiety" czyli jak młodzi polacy postrzegaja̧ hormonalna̧ antykoncepcjȩ postkoitalna̧]
137. a mutation in the mechanisms of socialcontrol the case of abortion
138. a noninferiority study of outpatient mifepristonemisoprostol medical abortion at 6470 days and 7177 days of gestation
139. a novel method for office aspiration curettage in cases of retained products of conception a randomized controlled trial
140. a phase iii multicentre study on medical termination of early pregnancy with two regimens of mifepristone followed by pg05
141. a pilot study of acceptability of ru486 and ono 802 in a chinese population
142. a pilot study of mifepristone and misoprostol administered at the same time for abortion up to 49 days gestation
143. a pilot study of the effect of methotrexate or combined oral contraceptive on bleeding patterns after induction of abortion with mifepristone and a prostaglandin pessary
144. a pilot study on the use of a 7day course of letrozole followed by misoprostol for the termination of early pregnancy up to 63 days
145. a pilotstudy of acceptability of ru486 and ono802 in a chinese population
146. a profile of abortion cases in a tertiary care hospital
147. a prospective economic evaluation comparing medical abortion (using mifepristone and gemeprost) and surgical vacuum aspiration
148. a prospective randomized, doubleblinded, placebocontrolled trial comparing mifepristone and vaginal misoprostol to vaginal misoprostol alone for elective termination of early pregnancy
149. a prospective study of mifepristone and unlimited dosing of sublingual misoprostol for termination of secondtrimester pregnancy in uzbekistan and ukraine
150. a prospective study of spontaneous fetal losses after induced abortions
151. a prospective trial using mifepristone and vaginal misoprostol in termination of pregnancies up to 63 days of gestation
152. a prospective, comparative study of clinical outcomes following clinicbased versus selfuse of medical abortion
153. a randomised comparative study on sublingual versus vaginal administration of misoprostol for termination of pregnancy between 13 to 20 weeks
154. a randomised controlled trial of mifepristone in combination with misoprostol administered sublingually or vaginally for medical abortion up to 13 weeks of gestation
155. a randomised study of two doses of gemeprost in combination with mifepristone for induction of abortion in the second trimester of pregnancy
156. a randomized comparative study on vaginal administration of acetic acidmoistened versus dry misoprostol for midtrimester pregnancy termination
157. a randomized controlled study of sublingual vs oral misoprostol (400 mg) following mifepristone (200 mg) for medical abortion up to 63 days lmp in tunisia
158. a randomized trial of hospital vs home self administration of vaginal misoprostol for medical abortion
159. a randomized trial of mifepristone in combination with misoprostol administered sublingually or vaginally for medical abortion at 1320 weeks gestation
160. a repeat dose of misoprostol 800 mcg following mifepristone for outpatient medical abortion at 6470 and 7177 days of gestation a retrospective chart review
161. a retrospective cohort analysis of retained products of conception with vascularity after secondtrimester abortion incidence, outcomes, and ultrasonographic features leading to severe hemorrhage
162. a retrospective cohort analysis of retained products of conception with vascularity after secondtrimester abortion;incidence, outcomes in expectant management, and characteristic ultrasonographic features leading to future severe hemorrhage
163. a retrospective comparison between lamicel and gemeprost for cervical ripening before surgical interruption of firsttrimester pregnancy
164. a retrospective service evaluation of early medical abortion at home by a district general hospital during the covid19 pandemic 2020 to 2022
165. a safe and effective method of termination of midtrimester pregnancy
166. a shortscheme protocol of gemeprost for midtrimester termination of pregnancy with uterine scar
167. a stalled revolution? misoprostol and the pharmaceuticalization of reproductive health in francophone africa
168. a statistical analysis of women's reproductive health characteristics after ineffective reattempts of using art
169. a study of 500 cases of acute renal failure (19781991)
170. a study of fertility and pregnancy outcomes in women after surgery for valvular heart disease
171. a study of incomplete abortion following medical method of abortion (mma)
172. a study of psychiatric morbidity during second trimester of pregnancy subsequent to abortion in the previous pregnancy
173. a study of the effects of folklore about the body on iud use by black american adolescents
174. a study of the prevalence of reproductive tract infections and its associated factors among married women of age 15 to 49 years in an adopted phc of adichunchanagiri institute of medical sciences, bg nagara, mandya
175. a successful twin pregnancy in a patient with hbeβthalassemia in western india
176. a survey on serious accidents associated with abortion before and after the law of 1975; [enquete sur les accidents graves de l'avortement avant et apres la loi de 1975]
177. a thoroughly regressive law
178. a twopill sublingual misoprostol outpatient regimen following mifepristone for medical abortion through 70 days' lmp a prospective comparative openlabel trial
179. a viable alternative to surgical vacuum aspiration repeated doses of intravaginal misoprostol over 9 hours for medical termination of pregnancies up to eight weeks
180. ablebodied women killing disabled babieshow modern narratives on disability and abortion erase disabled people from the reproductive justice movement
181. abortamento espontâneo um estudo epidemiológico
182. abortion
183. abortion an attitude study of professional staff at ramathibodihospital
184. abortion epidemiology, safety, and technique
185. abortion and legal policy
186. abortion at gondar college hospital, ethiopia
187. abortion at the 1st trimester of pregnancy the risks of responsibility from jurisprudence and the study of 81 complaints
188. abortion at the first trimester of pregnancy the risks of responsibility from jurisprudence and the study of 81 complaints; [l'ivg du premier trimestre et ses risques d'engagement de la responsabilite]
189. abortion attitudes in trinidad
190. abortion due to infection with chlamydia psittaci in a sheep farmer's wife
191. abortion education in canadian family medicine residency programs
192. abortion in belgium, 18801940; [abortus in belgië 18801940]
193. abortion in brazil what do the official data say?
194. abortion in brazil what do the official data say?; [aborto no brasil o que dizem os dados oficiais?]; [aborto en brasil ¿qué dicen los datos oficiales?]
195. abortion in four asian countries patient characteristics, morbidity and contraceptive acceptance
196. abortion in four asian countries patient characteristics, morbidity, and contraceptive acceptance
197. abortion in international human rights law missed opportunities in manuela v el salvador
198. abortion in minors
199. abortion in relation to fetal and maternal welfare
200. abortion in the first trimester guideline of the dggg (s2klevel, awmf registry no 015094, december 2022) part 1 with recommendations on care structures, information and advice on decisionmaking, measures before abortion and medical abortion
201. abortion in today's world
202. abortion incidence and services in the united states, 19951996
203. abortion induced with methotrexate and misoprostol a comparison of various protocols
204. abortion law in france
205. abortion law reform in india
206. abortion medical management between 14−16 weeks’ amenorrhea after french legislation deadline extension
207. abortion needs of women in india a case study of rural maharashtra
208. abortion pill a boon or a curse?
209. abortion policies and practices in chile ambiguities and dilemmas
210. abortion policy and women's health in developing countries
211. abortion policy and womens health in developingcountries
212. abortion policy implementation in ireland lessons from the community model of care
213. abortion practices in high school students in yamoussoukro, côte d’ivoire; [pratiques d’avortement chez des lycéennes à yamoussoukro, côte d’ivoire]
214. abortion practices in high school students in yamoussoukro, cote d'ivoire
215. abortion procedures in a tertiary care institution in india
216. abortion providers' experiences and views on selfmanaged medication abortion an exploratory study
217. abortion providers' resilience to antichoice tactics in the united states and canada
218. abortion related acute renal failure a study in dhaka medical college hospital
219. abortion reporting in the era of medical procedures why is it important?
220. abortion services and military medical facilities
221. abortion services during the covid19 pandemic a systematic review
222. abortion techniques in australia a history
223. abortion training at multiple sites an unexpected curriculum for teaching systemsbased practice
224. abortion trends in singapore a 25year review
225. abortion, breast cancer, and informed consent
226. abortion, informed consent, and regulatory spillover
227. abortion an attitude study of professional staff at ramathibodi hospital
228. abortions in a hospital setting hidden realities in daressalaam, tanzania
229. abortions in a hospital setting hidden realities in dar es salaam, tanzania
230. aborto no brasil o que dizem os dados oficiais?
231. about analysis of and reflections on some abortion request statistic
232. about the bioethics of abortion at request in romania a case presentation
233. abstract #1002287 a case of diabetic ketoacidosis in medical management of miscarriage
234. acceptability and feasibility of early pregnancy termination by mifepristonemisoprostol results of a large multicenter trial in the united states mifepristone clinical trials group
235. acceptability and feasibility of outpatient medical abortion with mifepristone and misoprostol up to 70 days gestation in singapore
236. acceptability of homeuse of misoprostol in medical abortion
237. acceptability of medical abortion in early pregnancy
238. accessibility, safety and outcomes in a united kingdom national health service nhs abortion clinic (walsall)
239. accessing abortion in a highly restrictive legal regime characteristics of women and pregnant people in malta selfmanaging their abortion through online telemedicine
240. accounting for time insights from a lifetable analysis of the efficacy of medical abortion
241. accuracy of anemia screening by pointofcare hemoglobin testing in patients seeking abortion
242. achievements of the figo initiative for the prevention of unsafe abortion and its consequences in southsoutheast asia
243. acquired uterine arteriovenous malformation management and treatment
244. acr appropriateness criteria® first trimester bleeding
245. acupuncture benefits to women with recurrent implantation failure a propensity scorematched cohort study
246. acute kidney injury in asia
247. acute renal failure during the pregnancy a review on pathophysiology, risk factors and management
248. acute renal failure following hexolinduced abortion
249. acute renal failure in blacks and indians in south africa comparison after 10 years
250. acute renal failure in obstetric patients treatment by hemodialysis
251. acute renal failure in obstetrics
252. acute renal failure in pregnancy
253. acute renal failure in pregnancy a decade of change
254. acute renal failure in pregnancy in a developing country twenty years of experience
255. acute renal failure in pregnancy 1987
256. acute uterine inversion following an induced abortion
257. acuterenalfailure in blacks and indians in southafrica comparison after 10 years
258. acuterenalfailure in pregnancy 1987
259. administration of depot medroxyprogesterone acetate on the day of mifepristone for medical abortion a pilot study
260. adolescent girls undergoing medical abortion have lower risk of haemorrhage, incomplete evacuation or surgical evacuation than women above 18 years old
261. adolescent pregnancy occurrence and consequences
262. adult highrisk burkitt’s acute lymphocytic leukemia was successfully rescued by rituximab combined with hypercvad/ma regimens two case reports and a literature review
263. advanced abdominal pregnancy as a cause of symptomatic hemoperitoneum
264. advanced practice clinicians and medical abortion increasing access to care
265. advances of medical termination of pregnancy amendments act in india
266. advantages and disadvantages of medical abortion, according to brazilian residents in obstetrics and gynaecology
267. adverse pregnancy outcome and childhood malignancy with reference to paternal welding exposure
268. afrocaribbean children in local authority day care 0 3
269. age, parity, history of abortion and contraceptive choices affect the risk of repeat abortion
270. alteration of th1 and th2 cells by intracellular cytokine detection in patients with unexplained recurrent abortion before and after immunotherapy with the husband's mononuclear cells
271. alternatives to mifepristone for early medical abortion
272. ambulatory medical abortion performed in a family planning center
273. amniocentesis before 15 weeks gestation technical aspects and obstetric risks
274. amniocentesis before 15 weeks' gestation technical aspects and obstetric risks
275. amyloidosis associated with igg (λ) mproteinemia a case of autopsy
276. an account of 2180 cases of percoelioscopic tubal ligation using yoon's mechanical method; [a propos de 2180 sterilisations tubaires percoelioscopiques par procede mecanique de yoon]
277. an account of 2180 cases of percoelioscopic tuballigation using yoon mechanical method
278. an analysis of the cost of incomplete abortion to the public health sector in south africa 1994
279. an effective regimen for early medical abortion a report of 2000 consecutive cases
280. an examination of loss to followup and potential bias in outcome ascertainment in a study of directtopatient telemedicine abortion in the united states
281. an experience with misoprostol for the induction of first trimester abortions in a secondary hospital in south africa
282. an overview of unsafe abortion in africa foreword
283. analysis of clinical characteristics and prognosis of 21 pregnant women complicated with tuberculosis after in vitro fertilizationembryo transfer; [21例体外受精胚胎移植后妊娠并发结核病患者的 临床特征及预后分析]
284. analysis of complications and management after selfadministration of medical termination of pregnancy pills
285. analysis of motivation of sterilised women wishing to be refertilised
286. analysis of pharmacodynamic components, targets and synergistic action mechanism of fuyuan shenghua granule for the treatment of medicalinduced incomplete abortion based on network pharmacology
287. analysis of severe adverse events related to the use of mifepristone as an abortifacient
288. angiographic transcatheter embolization in gynaecological practice
289. anticonvulsant therapy for status epilepticus
290. antifertility effect of continuous lowdosage oral progestogen therapy
291. antioxidants for male subfertility
292. antiphospholipid syndrome
293. antiprogesterone for medical abortion
294. antiprogesterones for the interruption of pregnancy
295. apf open letter to the political parties, on the abortion situation in portugal; [apf carta aberta aos partidos politicos, sobre a situacao do aborto em portugal]
296. application effect of different ovarian stimulation protocols in elderly patients with diminished ovarian reserve; [不同促排卵方案在高龄卵巢储备功能减退患者中的应用效果比较]
297. application of multiplex quantitative fluorescent pcr with nonpolymorphic loci in prenatal diagnosis
298. are serum progesterone levels predictive of recurrent miscarriage in future pregnancies?
299. are us health professionals likely to prescribe mifepristone or methotrexate?
300. artificial insemination with fresh or frozen semen a comparative study
301. aspects of the significance of operatively corrected heart valve failure on fertility; [aspekte zur bedeutung operativ korrigierter herzklappenvitien auf die fertilitat]
302. aspects of the significance of operatively corrected heartvalve failure on fertility
303. assessing an ovarian reserve and risk factors for premature ovarian failure as part of preabortion counseling for women under 40 planning to terminate own first pregnancy; [исследование овариального резерва и факторов риска преждевременной недостаточности яичников в рамках доабортного консультирования женщин в возрасте до 40 лет, планирующих прервать первую беременность]
304. assessing postabortion care in health facilities in afghanistan a crosssectional study
305. assessing strength of preference for abortion method using 'willingness to pay' a useful research technique for measuring values
306. assessing survival time of outpatients with cervical cancer at the university of gondar referral hospital using the bayesian approach
307. assessment of completion of early medical abortion using a text questionnaire on mobile phones compared to a selfadministered paper questionnaire among women attending four clinics, cape town, south africa
308. assessment of efficacy and safety of medical treatment of nonviable first trimester pregnancy
309. assessment of efficacy and safety of medical treatment of nonviable first trimester pregnancy; [ocena skuteczności i bezpieczeństwa leczenia zachowawczego w przypadkach ciąży obumarłej w pierwszym trymestrze]
310. assessment of rapid radioimmunoassay for urinary βhuman chorionic gonadotropin (βhcgria) in early pregnancy
311. assessment of results and notes on the organisational aspects of law no 194 of 1978 as applied in a provincial hospital interruption of pregnancy; [analisi dei risultati, considerazioni di ordine organizzativo circa la legge 194/78 applicata in un ente ospedaliero di provincia]
312. assessment of the efficacy of different methods of second trimester abortion an initial experience in eastern nepal
313. association between the route of administration and efficacy of the misoprostol in the management of missed abortion a randomized clinical trial
314. association between ultrasonographic parameters of cesarean scar defect and outcome of early termination of pregnancy
315. association between uterine position and transvaginal misoprostol treatment for early pregnancy failure
316. association of the g2014g genotype in estrogen receptor 1 gene with failure of the mifepristoneinduced termination of early pregnancy
317. attitudes to contraception in women seeking termination of pregnancy
318. attributes and perspectives of public providers related to provision of medical abortion at public health facilities in vietnam a crosssectional study in three provinces
319. autoantibodies in normal and abnormal pregnancy
320. avoiding abortion stigma how the “invisibilization labour” reinforces the procreative labour; [esquiver le stigmate lié à l’avortement le « travail d’invisibilisation» comme renforcement du travail procréatif]
321. azzolino v dingfelder
322. bacterial sacroiliitis and gluteal abscess after dilation and curettage for incomplete abortion
323. bacteroidaceae bacteremia effect of age and focus of infection upon clinical course
324. barriers and enablers to nurse practitioner provision of medication abortion in canada results from a national survey
325. bioartificial liver from cultured human foetal hepatocytes feasibility and prospects
326. birth control and social group contraception, accidental pregnancies and abortion; [maitrise de la fecondite et appartenance sociale contraception, grossesses accidentelles et avortements]
327. birthcontrol and social group contraception, accidental pregnancies and abortion
328. bleeding after dilatation and curettage the efficacy of transcatheter uterine artery embolisation
329. bleeding patterns after vaginal misoprostol for treatment of early pregnancy failure
330. blood loss with mifepristonemisoprostol abortion measures from a trial in china, cuba and india
331. body failure medical views of women, 19001950
332. brazil investigates drugs possible link with birthdefects
333. british abortion act
334. buxue yimu pills improve angiogenesis and blood flow in experimental zebrafish and rat models
335. cabergoline a review of its pharmacological properties and therapeutic potential in the treatment of hyperprolactinaemia and inhibition of lactation
336. california may become first state to mandate insurance coverage for contraception
337. can midlevel healthcare providers administer early medical abortion as safely and effectively as doctors? a randomised controlled equivalence trial in nepal
338. can the outcome of the next pregnancy be predicted at the time of induced abortion?
339. can women accurately assess the outcome of medical abortion based on symptoms alone?
340. can women determine the success of early medical termination of pregnancy themselves?
341. canadian contraception consensus (part 1 of 4)
342. canadian contraception consensus (part 2 of 4)
343. candida sepsis in pregnancy and the postpartum period
344. cannulation pitfalls in ecmo
345. cardiac surgical experience in northern nigeria
346. cardiovascular disease and associated comorbid conditions as determinants of adverse perinatal outcomes in pregnancy an analysis of the results of the register of pregnant bereg
347. case study on the wrongful birth action
348. catastrophic antiphospholipid syndrome
349. catastrophic antiphospholipid syndrome during pregnancy and puerperium maternal and fetal characteristics of 15 cases
350. causative factors of premature birth (a new look at the problem); [причинные факторы преждевременных родов (новый взгляд на проблему)]; [причинні фактори передчасних пологів (новий погляд на проблему)]
351. causes of acute renal failure in nishtar hospital multan
352. causes of maternal death in ethiopia between 1990 and 2016 systematic review with metaanalysis
353. causes of perinatal mortality report of a study of 4,429 cases
354. cervical ectopic pregnancy
355. cervical ectopic pregnancy diagnosis with endovaginal ultrasound examination and successful treatment with methotrexate
356. cervical internal os cerclage description of a new technique and comparison with shirodkar operation
357. cervical pregnancy a rare case of reimplantation after abortion a case report
358. cervical pregnancy a rare case of reimplantation after abortion a case report; [la réimplantation cervicale de la grossesse, une complication méconnue des interruptions volontaires de grossesse à propos d'un cas]
359. cervical twin heterotopic pregnancy overview of ectopic pregnancies and scanning detection algorithm
360. cervical vasovagal shock a rare complication of incomplete abortion case report
361. cesarean scar ectopic pregnancy the lurking danger in post cesarean failed medical abortion
362. cfilm a new local contraceptive
363. challenging cases in care for termination of pregnancy
364. change in abortion services after implementation of a restrictive law in texas
365. change in abortion services after implementation of a restrictive lawin texas
366. change in attitudes about illegal abortion; [panoramawandel illegaler schwangerschaftsabbrüche]
367. change in serum betahuman chorionic gonadotropin after abortion with methotrexate and misoprostol
368. change in serum βhuman chorionic gonadotropin after abortion with methotrexate and misoprostol
369. changes in abortion service provision in bihar and jharkhand states, india between 2004 and 2013
370. changing trends in maternity care in sri lanka
371. characterisation of the molecular basis underlying chlamydial subversion of focal adhesion signaling
372. characteristics of maternal cardiac disease and pregnancy outcomes results from a 4year observational cohort survey in central vietnam
373. characteristics of medical termination of pregnancy acceptors in pondicherry state, 19721976
374. characteristics of women having abortion in china
375. chemical fingerprint analysis and ultraperformance liquid chromatography quadrupole timeofflight mass spectrometrybased metabolomics study of the protective effect of buxue yimu granule in medicalinduced incomplete abortion rats
376. choice at any cost
377. choice of analgesia or anesthesia for pain relief in suction curettage
378. choosing abortion teens who make the decision without parental involvement
379. chorionic villi sampling cytogenetic and clinical findings in 500 pregnancies
380. chronic bone marrow failure due to persistent b19 parvovirus infection
381. chronic myeloid leukemia comparison of survival between pregnant and nonpregnant women
382. clandestine abortion in port harcourt users' profile and motivation
383. cleavage鈥恠tage versus blastocyst鈥恠tage embryo transfer in assisted reproductive technology
384. cleft lip and palate series of unusual clinical cases
385. client perspectives on choice of abortion method in england and wales
386. clinical analyses of 66 cases of midtrimester pregnancy termination in women with prior cesarean
387. clinical analysis of ectopic pregnancies in a tertiary care centre in southern india a sixyear retrospective study
388. clinical analysis of secondtrimester pregnancy termination after previous caesarean delivery in 51 patients with placenta previa and placenta accreta spectrum a retrospective study
389. clinical and morphological differences in nondeveloping pregnancy and spontaneous abortion in early pregnancy
390. clinical applications of mifepristone
391. clinical approach and surgical strategy for spinal disorders in pregnant women
392. clinical course of hepatitis b virus infection during pregnancy
393. clinical diagnosis of completeness of medical abortion by nurses a reliability study in mozambique
394. clinical effects of myotonic dystrophy on pregnancy and the neonate
395. clinical experience with intramuscular norethisterone oenanthate as a contraceptive
396. clinical followup compared with selfassessment of outcome after medical abortion a multicentre, noninferiority, randomised, controlled trial
397. clinical indication of medical ultrasound as a diagnostic tool among the outdoor patients of a selected hospital in bangladesh
398. clinical manifestations of pregnancy in patients with takayasu arteritis experience from a single tertiary center
399. clinical observation on termination of early pregnancy of 213 cases after caesarian section with repeated use of mifepristone and misoprostol
400. clinical outcomes of luteal phase stimulation for in vitro fertilization/intracytoplasmic sperm injection treatment in poor ovarian responders; [卵巢低反应患者黄体期促排卵行体外受精/卵胞质内单精子显微注射助孕临床结局分析]
401. clinical pattern of gynecological/early pregnancy complaints and the outcome of pelvic sonography in a private diagnostic center in ilorin
402. clinical research of sequential embryo transfer in frozen thawed cycles of patients with recurrent implantation failure; [解冻周期序贯移植治疗反复种植失败的临床研究]
403. clinical significance of sub chorionic and retroplacental hematomas detected in the first trimester of pregnancy
404. clinical study of early pregnancy termination by administration of dl15methyl prostaglandin f2 alpha combined with tamoxifen or norethisterone
405. clinical study of four cases with malignant gestation trophoblastic tumor after mifepristone abortion
406. clinical study of terminating biochemical pregnancy and early clinical pregnancy with mifepristone and misoprostol
407. clinical study of termination of early pregnancy by combination of dl15methylprostaglandin f2 alpha and ru 486
408. clinical trial on termination of early pregnancy with ru486 in combination with prostaglandin
409. clinical use and safety of medical method of first trimester abortion
410. clinical, surgical, and histopathologic outcomes following failed medical abortion
411. clinicaltrial on termination of earlypregnancy with ru486 in combination with prostaglandin
412. clinicianled transformation of sexual and reproductive healthcare in ni postdecriminalisation of abortion
413. clinton reform proposal faces congressional test on covered services, cost
414. cognitive bias in the management of a critically ill 29yearold patient
415. college women's experience with emergency contraception
416. combined vesicouterine rupture during secondtrimester medical abortion for fetal abnormality after prior cesarean delivery a case report
417. comment "analysis of and reflections on some abortion request statistics" (letter); [a propos de "analyse d'une statistique de demandes d'ivg et les reflexions qu'elle suscite"]
418. commentary access to medicalassisted reproduction and pgd in italian law a deadly blow to an illiberal statute? commentary to the european court on human rights's decision costa and pavan v italy
419. comparative effectiveness, safety and acceptability of medical abortion at home and in a clinic a systematic review
420. comparative effectiveness, safety and acceptability of medical abortion at home and in a clinic a systematic review; [comparación de la efectividad, la seguridad y la aceptación de los abortos médicos practicados en el domicilio con aquellos realizados en la clínica una revisión sistemática]
421. comparative study of efficacy and acceptability of two regimens for 1st trimester mtp
422. comparative study of mifepristone with vaginal misoprostol for first trimester termination of pregnancy at different gestational ages
423. comparative study of regimen mifepristone and misoprostol with misoprostol alone at first trimester termination of pregnancy
424. comparative study of transdermal nitroglycerine patch and oral nifedipine in preterm labour
425. compare of misoprostol and dinoprost effectivity by induced secondtrimester abortion; [srovnání efektivity misoprostolu a dinoprostu při indukci druhotrimestrálního abortu]
426. comparing pregnancy outcomes between symptomatic and asymptomatic covid19 positive unvaccinated women multicenter study in saudi arabia
427. comparing the effects of misoprostol/letrozole and misoprostol/placebo on medical abortion success rate a randomized clinical trial
428. comparison between the effectiveness of combination of letrozole with misoprostol and tamoxifen with misoprostol in medical termination of first trimester missed miscarriage
429. comparison of adolescent and adult experiences with norplant levonorgestrel contraceptive implants
430. comparison of clinical outcomes of vitrifiedthawed embryo transfer and fresh embryos transfer
431. comparison of intraamniotic prostaglandin f2αand hypertonic saline for induction of secondtrimester abortion
432. comparison of medical abortion with surgical vacuum aspiration women's preferences and acceptability of treatment
433. comparison of mifepristone plus misoprostol with misoprostol alone for first trimester medical abortion a systematic review and metaanalysis
434. comparison of misoprostolonly and combined mifepristonemisoprostol regimens for homebased early medical abortion in tunisia and vietnam
435. comparison of obstetric emergency clinical readiness a crosssectional analysis of hospitals in amhara, ethiopia
436. comparison of outcomes before and after ohio's law mandating use of the fdaapproved protocol for medication abortion a retrospective cohort study
437. comparison of rates of adverse events in adolescent and adult women undergoing medical abortion population register based study
438. comparison of sublingual, oral and vaginal misopristol for cervical ripening 12 hours before hysteroscopy
439. comparison of the effects of voluntary termination of pregnancy and uterine evacuation for medical reasons on female sexual function
440. comparison of the efficacy and safety of two combined misoprostol regimens for second trimester medical abortion
441. comparison of two doses of mifepristone in combination with misoprostol for early medical abortion a randomised trial
442. comparison of two lowsensitivity urine pregnancy tests for confirming the success of early medical abortion
443. comparison of vaginal and sublingual misoprostol for second trimester abortion randomized controlled equivalence trial
444. comparitive study of early (24 hours) versus late (48 hours) misoprostol administration after mifepristone for termination of early pregnancy
445. compliance with new ban means no mention of abortion
446. complicated illegal induced abortions at a tertiary health institution in nigeria
447. complications after second trimester surgical and medical abortion
448. complications during pregnancy and delivery in women with untreated rectovaginal deep infiltrating endometriosis
449. complications of induced abortions; [complications des interruptions volontaires de grossesse]
450. complications of misoprostol and other abortion induction methods in the developing world a systematic review
451. complications of second trimester induction for abortion or fetal demise for patients with and without prior cesarean delivery
452. complications of unsafe abortion a case study and the need for abortion law reform in nigeria
453. complications related to induced abortion a combined retrospective and longitudinal followup study
454. comprehensive abortion care evidence of improvements in hospitallevel indicators in tigray, ethiopia
455. comprehensive genetic analysis of pregnancy loss by chromosomal microarrays outcomes, benefits, and challenges
456. concurrent copper t insertion with medical termination of pregnancy in women with previous caesarean section delivery
457. concurrent coppert insertion with medical termination of pregnancy in women with previous cesareansection delivery
458. conflicting recommendations between the french national authority for health (has) and clinical practice guidelines (cngof); focus on 200 late medical abortions, conducted outside marketing authorizations
459. conflicting recommendations between the french national authority for health (has) and clinical practice guidelines (cngof); focus on 200 late medical abortions, conducted outside marketing authorizations; [recommandations discordantes entre has et cngof; le point avec 200 ivg médicamenteuses tardives, réalisées hors amm]
460. congenital heart disease and acquired valvular lesions in pregnancy
461. congenital heart disease and acquired valvular lesions in pregnancy; [angeborene herzfehler und erworbene herzklappenfehler in der schwangerschaft]
462. conscientious objection to sexual and reproductive health services international human rights standards and european law and practice
463. conservative management of uterine necrosis following bilateral uterine vessels ligation and blynch suture long term follow up
464. consistency of medical abortion efficacy from 5 through 14 weeks' gestation
465. constraints and optimization of the laser microwelding process of thin metal foils
466. construction and validation of a spatial database of providers of transgender hormone therapy in the us
467. continuation of pregnancy after medical advice in patients with desired abortion
468. continued clinical experience with an increasing dosage regimen of clomiphene citrate administration
469. contours of development
470. contraception and diabetes mellitus
471. contraception for the teenager
472. contraception in adolescence knowing is not enough; [la contraception à l'adolescence savoir ne suffit pas]
473. contraception utilization in black women via a reproductive justice lens
474. contraception failure in practice
475. contraception from accessibility to efficiency
476. contraception; [la contraccezione]
477. contraceptive behavior of jerusalem women seeking pregnancy counseling, 19801989
478. contraceptive compliance why is contraceptive failure still so frequent?; [kontrazeptive compliance warum kommt es immer wieder zum versagen der kontrazeptiven therapie?]
479. contraceptive coverage after medical termination of pregnancy
480. contraceptive discontinuation among white, black, and hispanic adolescents
481. contraceptive efficacy; [a eficacia contraceptiva]
482. contraceptive failure rates new estimates from the 1995 national survey of family growth
483. contraceptive practice of abortion patients
484. contraceptive practices of women attending for termination of pregnancy a study from south australia
485. contraceptive problems unique to the united states
486. contraceptive risktaking and contraceptive failure among users of female barrier methods
487. contraceptive use among women seeking repeat abortion in addis ababa, ethiopia
488. control of tropical theileriosis (theileria annulata infection in cattle) in north africa
489. controversias en el tratamiento del aborto incompleto ameu versus tratamiento médico con misoprostol
490. controversy surrounds use of test for open spina bifida
491. coping with fertility in israel a case study of culture clash
492. cordarone in obstetrics may or not?
493. coronary artery vasospasm after misoprostol treatment for incomplete abortion a case report
494. correction management of septic chemical abortion with renal failure (n engl j med (1975) 292 (722725) (101056/nejm197504032921404))
495. corrections simultaneous compared with interval medical abortion regimens where home use is restricted (obstetrics and gynecology (2018) 131 (63541) doi 101097/aog0000000000002536)
496. cost effective & scalable community models for safe abortion in low resource countries in south asia
497. costeffectiveness of alternative strategies for firsttrimester abortion in mexico and nigeria
498. costeffectiveness of emergency contraception options over 1 year
499. costeffectiveness of levonorgestrel subdermal implants comparison with other contraceptive methods available in the unitedstates
500. costeffectiveness of levonorgestrel subdermal implants comparison with other contraceptive methods available in the united states
501. costeffectiveness of managing abortions manual vacuum aspiration (mva) compared to evacuation by curettage in tanzania
502. costeffectiveness of managing abortions manual vacuum aspiration (mva) compared to evacuation by curettage in tanzania
503. could american women use mifepristonemisoprostol pills safely with less medical supervision?
504. counseling women about childbearing and childrearing risks
505. craniorachischisis with incomplete twins diprosopus report of a case and review of the literature
506. crimes related to illegally carrying out an artificial termination of pregnancy (abortion) in the legislation of foreign countries; [преступления, связанные с незаконным проведением искусственного прерывания беременности (аборта), в законодательстве зарубежных стран]
507. criminality
508. crisis pregnancy centers in the us lack of adherence to medical and ethical practice standards a joint position statement of the society for adolescent health and medicine and the north american society for pediatric and adolescent gynecology
509. crisis pregnancy centers in the united states lack of adherence to medical and ethical practice standards; a joint position statement of the society for adolescent health and medicine and the north american society for pediatric and adolescent gynecology
510. crisis pregnancy centers in the us lack of adherence to medical and ethical practice standards a joint position statement of the society for adolescent health and medicine and the north american society for pediatric and adolescent gynecology society for adolescent health and medicine and the north american society for pediatric and adolescent gynecology
511. critical appraisal of contraceptive research
512. cuban health system call it superior?
513. cuban health system call it superior? [letter]
514. culture media for human pre鈥恑mplantation embryos in assisted reproductive technology cycles
515. curettage and asherman's syndromelessons to (re) learn?
516. current and potential methods for second trimester abortion
517. current diagnostics and treatments for immune mediated recurrent spontaneous miscarriages and ivf implantation failures a review; [az immunpatológiai hátteru visszatéro spontán vetélések és sikertelen ivf beültetések korszeru diagnosztikája és kezelési lehetoségei]
518. current management of complete and partial molar pregnancy
519. current management of molar pregnancy
520. current medical abortion care
521. current state and future of ivf
522. cushing's syndrome during pregnancy secondary to adrenal adenoma metyrapone treatment and laparoscopic adrenalectomy
523. cutting unsafe abortions
524. cytogenetic studies of couples with reproductive failure in alexandria, egypt
525. cytomegalovirus and the expression of immunological markers in reproductive failure
526. data for reform of french legislation on abortion; [eléments pour une réforme de la législation française de l'avortement]
527. dealing with the barriers to access to abortion
528. decentralising medical abortion services in victoria, australia
529. deciphering the voices in abortion care the woman's voice matters
530. decision making by single women seeking abortion
531. decision making on unsafe abortions in sri lanka a casecontrol study
532. decisionmaking and choice about childbearing a case study of deferred motherhood
533. declining risk of surgical intervention following early medical abortion a time trend analysis
534. decreasing the need for abortion challenges and constraints
535. deformidad del principio de autonomía para sustentar actos éticamente ilícitos
536. delayed hemolysis, elevated liver enzymes, low platelet count syndrome in succession of switches of preventive anticoagulant treatment in a 41yearold patient with a history of recurrent assisted implantation failures a case report
537. delivering on the promise ippf's global action to safe abortion services
538. demand for abortion special aspects of druginduced abortion; [la demande d'avortement aspects particuliers de l'ivg médicamenteuse]
539. dengue fever during pregnancy maternal and fetal complications
540. depot medroxyprogesterone acetate (dmpa) administration at the time of mifepristone for medical abortion a pilot study
541. dermatoglyphics associated with fetal wastage
542. design of a syringe extension device (chloe sed) for lowresource settings in subsaharan africa a circular economy approach
543. design of a syringe extension device (chloe sed®) for lowresource settings in subsaharan africa a circular economy approach
544. design options and methodological fallacies in the studies of reproductive failures
545. determinants and outcome of safe second trimester medical abortion at jimma university medical center, southwest ethiopia
546. determinants of abortion among women admitted to hospitals in fortaleza, north eastern brazil
547. determinants of low birthweight methodological assessment and metaanalysis
548. determination of pregnant women knowledge toward risk factors of vitamin d deficiency and measuring level during pregnancy in al nasiriyah city
549. development and evaluation of the abortion attributes questionnaire
550. development and pilot testing of the 2019 canadian abortion provider survey
551. developmental and reproductive risks of radiological procedures utilizing ionizing radiation during pregnancy
552. developmental toxicity of misoprostol an update; [toxicidad del misoprostol sobre la gestación revisión de la literatura]
553. diagnosis and clinical criteria for chronic endometritis; [диагностика и клинические критерии хронического эндометрита]
554. diagnosis of aneuploidy in archival, paraffinembedded pregnancyloss tissues by comparative genomic hybridization
555. diagnosis of aneuploidy in arrival, paraffinembedded pregnancyloss tissues by comparative genomic hybridization
556. diagnosis of medulloblastoma in pregnant women to continue with the pregnancy or not? a case report and literature review
557. diagnostic value of prenatal mr imaging in the detection of brain malformations in fetuses before the 26th week of gestational age
558. diaphragm method contraceptors implications for service organization and delivery
559. diaphragm method contraceptors implications for service organization and delivery
560. die neuere entwicklung des arztrechts
561. different types of serologic reactivity to bedsonia (psittacosis group) antigen in various hosts discussion of some related problems
562. direct effects of mifepristone on mice embryogenesis an in vitro evaluation by singleembryo rna sequencing analysis
563. discussing the best way of followup
564. disfunção tiroideia em grávidas de alto e baixo risco numa região com défice de iodo (beira interior)
565. disruptive congenital anomalies associated with misoprostol case report; [anomalías congénitas disruptivas asociadas con misoprostol reporte de un caso]
566. district court finds proposed iowa women's health facility does not need state approval
567. do the risks of a diagnostic xray during early pregnancy justify therapeutic abortion?; [les risques d'un examen de radiodiagnostic au debut de la gestation justifientils l'interruption de celleci?]
568. doctors speak of their experience in announcing a diagnosis announcing to parents seriouschronic disease in their child; [des médecins parlent de leur expérience de l'annonce l'annonce d'une maladie grave de l'enfant à ses parents]
569. does congenital heart disease severely jeopardise family life and pregnancies? obstetrical history of women with congenital heart disease in a single tertiary centre
570. does followup with serum hcg simplify medical abortion? a randomized controlled trial
571. does methotrexate confer a significant advantage over misoprostol alone for early medical abortion? a retrospective analysis of 8678 abortions
572. does starting progestinonly contraception on the same day as a medical abortion with mifepristone affect completion of the abortion? a review of clinic records from mexico city
573. does sublingual misoprostol reduce pain and facilitate iud insertion in women with no previous vaginal delivery? a randomized controlled trial
574. does supportive legislation guarantee access to pregnancy termination and postabortion care services? findings from a facility census in central province, zambia
575. doppler velocimetry of the uterine arteries an early screening test for miscarriage
576. doubleblind randomized trial of mifepristone in combination with vaginal gemeprost or misoprostol for induction of abortion up to 63 days gestation
577. drug interactions between oral contraceptives and antibiotics
578. early abortion with buccal versus sublingual misoprostol alone a multicenter, randomized trial
579. early medical abortion first experiences
580. early medical abortion using lowdose mifepristone followed by buccal misoprostol a large australian observational study
581. early medical abortion with selfadministered lowdose mifepristone in combination with misoprostol
582. early pregnancy failure current management concepts
583. early pregnancy termination with a simplified mifepristone medical abortion outpatient regimen
584. early pregnancy termination with mifepristone and misoprostol in norway
585. early pregnancy termination with mifepristone and misoprostol in the united states
586. early pregnancy termination with oral mifepristone and vaginal misoprostol
587. early surface ablation on aborted lasik flaps
588. early termination of pregnancy with mifepristone (ru 486) and the orally active prostaglandin misoprostol
589. early versus delayed insertion of nexplanon at medical abortiona randomized controlled equivalence trial
590. early versus late misoprostol administration after mifepristone for medical abortion
591. economic cost of pregnancy care attributable to the failure of mexico's teenage pregnancy prevention policy
592. economic cost of pregnancy care attributable to the failure of mexico's teenage pregnancy prevention policy; [costo económico de atención de embarazos atribuibles a la falla de la política de prevención del embarazo adolescente en méxico]
593. ectopic pregnancy in the era of medical abortion are we ready for it? spectrum of sonographic findings and our experience in a tertiary care service hospital of india
594. ectopic pregnancy a lifethreatening gynecological emergency
595. effect of abortion, in vitro fertilization, and other causes of prenatal death on life expectancy in the united states from 1925 to 2005
596. effect of endometrial stimulation on pregnancy outcome of in vitro fertilization patients
597. effect of folic acid supplementation on diminished ovarian reserve study protocol of a singlecentre, openlabel, randomised, placebocontrolled clinical trial
598. effect of immediate compared with delayed insertion of etonogestrel implants on medical abortion efficacy and repeat pregnancy
599. effect of immediate compared with delayed insertion of etonogestrel implants on medical abortion efficacy and repeat pregnancy a randomized controlled trial
600. effect of letrozole on uterine artery doppler flow indices prior to firsttrimester termination of pregnancy a randomized controlled trial
601. effect of pregnancy in arrhythmogenic right ventricular cardiomyopathy
602. effect of previous live birth and prior route of delivery on the outcome of early medical abortion
603. effect of wujia shenghua capsule on uterine bleeding following medicallyinduced incomplete abortion in rats during early pregnancy
604. effective, lowcost regimens for medical termination of pregnancy at all gestations
605. effectiveness and acceptability of “at home” versus “at hospital” early medical abortion a lesson from the covid19 pandemic a retrospective cohort study
606. effectiveness and acceptability of home use of misoprostol for medical abortion up to 10 weeks of pregnancy
607. effectiveness and acceptability of home use of misoprostol for medical abortion up to 10 weeks of pregnancy
608. effectiveness and safety of early medication abortion provided in pharmacies by auxiliary nursemidwives a noninferiority study in nepal
609. effectiveness and safety of sublingual misoprostol in medical treatment of the 1st trimester miscarriage experience of offlabel use in korea
610. effectiveness of early medical abortion using lowdose mifepristone and buccal misoprostol in women with no defined intrauterine gestational sac
611. effectiveness of genetic consultation and genetic prevention
612. effectiveness of jianpiantai formula for the pregnancy outcome of in vitro fertilization and embryo transfer in infertile women protocol of a randomized controlled trial
613. effectiveness of selfmanaged medication abortion between 9 and 16 weeks of gestation
614. effectiveness of the diaphragm, used continuously, without spermicide
615. effectiveness of the figo protocol for medical management of firsttrimester abortion
616. effects of growth hormone in downregulating hormone replacement cycle on the outcome of frozenthawed embryo transfer; [降调节后激素替代方案中应用生长激素 对冻融胚胎移植周期结局的影响]
617. effects of laserassisted hatching on the clinical outcome of freezethawedcleavage embryo transfer in patients with repeated implantation failure; [激光辅助孵化对反复种植失败患者行冻融卵裂期胚胎移植临床结局的影]
618. effects of leonurine hydrochloride on medically induced incomplete abortion in early pregnancy rats
619. effects of making emergency contraception available without a physician's prescription a populationbased study
620. effects of mifepristone on fibronectin in human villus and deciduas
621. effects of neonatal intensive care on the mortality rate of idiopathic respiratory distress syndrome; [influencia de los cuidados intensivos neonatales en la mortalidad del sindrome de la dificultad respiratoria idiopatica]
622. effects of taking estrogen and progestogen after medical abortion on reducing vaginal hemorrhage time a randomizedcontrolled trial
623. efficacy and acceptability of a mifepristonemisoprostol combined regimen for early induced abortion among women in mexico city
624. efficacy and safety of intravaginal misoprostol for midtrimester medical termination of pregnancy
625. efficacy and safety of mifepristone and buccal misoprostol versus buccal misoprostol alone for medical abortion
626. efficacy and safety of mifepristonebuccal misoprostol for early medical abortion in an australian clinical setting
627. efficacy and safety of the medical method in the voluntary interruption pregnancy over 8 yearsexperience of a portuguese medical centre
628. efficacy and safety of uterine tamponade to control bleeding and reduce obstetric hysterectomy historic cohort in nuevo león, méxico, 2013; [eficacia y seguridad del taponamiento uterino para control de hemorragia y disminución de histerectomía obstétrica cohorte histórica en nuevo león, méxico, 2013]
629. efficacy of combination therapy with methotrexate and misoprostol in termination of pregnancy in the first trimester
630. efficacy of concurrent administration of mifepristone and misoprostol for termination of pregnancy
631. efficacy of medical abortion prior to 6 gestational weeks a systematic review
632. efficacy of mifepristone and misoprostol in late first trimester medical abortion, missed abortion and blighted ovum
633. efficacy of mifepristonemisoprostol combination in cases of missed abortion in first trimester
634. efficacy of misoprostol administration 24 hours after mifepristone for termination of early pregnancy
635. efficacy of misoprostol alone for firsttrimester medical abortion a systematic review
636. efficacy of misoprostol in relation to uterine position in the treatment of early pregnancy failure
637. efficacy of single dose of mifepristone combined with two doses of misoprostol in early medical abortions
638. efficacy of single dose vaginal misoprostol 800μg in i trimester abortion
639. efficacy, safety, and acceptability of misoprostol in the treatment of incomplete miscarriage a systematic review and metaanalysis
640. efficacy, safety, and acceptability of misoprostol in the treatment of incomplete miscarriage a systematic review and metaanalysis; [eficacia, seguranca e aceitabilidade do misoprostol no tratamento do aborto incompleto uma revisao sistem tica e metanalise]
641. eficacia y seguridad del taponamiento uterino para control de hemorragia y disminución de histerectomía obstétrica cohorte histórica en nuevo león, méxico, 2013
642. eisenmenger syndrome factors relating to deterioration and death
643. eisenmenger syndrome factors relating to deterioration and death
644. elective abortion as a primary health service in rural india experience with manual vacuum aspiration
645. elevated mrna expression of pgf2 receptor splice variant 2(fpv2) in human decidua is associated with incomplete mifepristonemisoprostolinduced early medical abortion by regulation of interleukin8
646. emergency contraception for adolescents; [la contraccezione d'emergenza in adolescenza considerazioni generali e proposte di un gruppo di lavoro]
647. emergency contraception plan b
648. emergency contraception presently available formulations and controversies surrounding their use
649. emergency contraceptive pills a simple proposal to reduce unintended pregnancies
650. emergency contraceptive pills a simple proposal to reduce unintended pregnancies
651. employee benefits no abortion benefits, no contracts
652. encefalocele occipital gigante sin complicaciones neonatales inmediatas
653. endometrial polyp filled with gestational tissues remained undiscovered in an infertile woman for years a case report
654. endometrial preparation for women undergoing embryo transfer with frozen embryos or embryos derived from donor oocytes
655. endometrial thickness and serum betahcg as predictors of the effectiveness of oral misoprostol in early pregnancy failure
656. endometrial thickness and serum βhcg as predictors of the effectiveness of oral misoprostol in early pregnancy failure
657. endometrial thickness as predictor of the effectiveness of intracervical misoprostol in early pregnancy failure
658. endometrial thickness following medical abortion is not predictive of subsequent surgical intervention
659. enduring politics the culture of obstacles in legislating for assisted reproduction technologies in ireland
660. epidemiology
661. epidemiology of spontaneous pregnancy loss in kazakhstan a national populationbased cohort analysis during 20142019 using the national electronic healthcare system
662. errors in after medical abortion recovery period and their overcoming through the training of ob&gyn
663. estimates of demand for abortion among soviet immigrants in israel
664. estimates of pregnancies averted through california's family planning waiver program in 2002
665. estimating the costs for the treatment of abortion complications in two public referral hospitals a crosssectional study in ouagadougou, burkina faso
666. estimation of the adolescent pregnancy rate in thailand 20082013 an application of capturerecapture method
667. estudios citogenéticos en pacientes con fallas reproductivas pinar del río, 20152020
668. ethical issues in prenatal diagnosis
669. ethical issues in prenataldiagnosis
670. ethics and medical decisionmaking
671. ethics and reproductive health a principled approach
672. etiologies and subsequent reproductive performance of 100 couples with recurrent abortion
673. etonogestrel implant at the time of mifepristone for medical abortion
674. evaluating wellwoman clinics
675. evaluating women’s acceptability of treatment of incomplete second trimester abortion using misoprostol provided by midwives compared with physicians a mixed methods study
676. evaluating women's acceptability of treatment of incomplete second trimester abortion using misoprostol provided by midwives compared with physicians a mixed methods study
677. evaluation of a lower dose schedule of intramuscular 15(s)15methyl prostaglandin f2 alpha for induction of early midtrimester abortion
678. evaluation of a network of medical abortion providers in two districts of maharashtra, india
679. evaluation of abortion services and patient characteristics in a mexico city public hospital after legalization
680. evaluation of anticoagulant use during pregnancy in mothers with antiphospholipid syndrome an observational crosssectional study
681. evaluation of competitive elisa for detection of antibodies to rift valley fever virus in cattle and sheep sera
682. evaluation of different doses of femoston therapy for incomplete abortion a prospective observational trial
683. evaluation of effect of letrozole prior to misoprostol in comparison with misoprostol alone in success rate of induced abortion
684. evaluation of irreversible contraception; [zum stellenwert der irreversiblen kontrazeption]
685. evaluation of the incidence and outcome of gestational diabetes mellitus using the current international consensus guidelines for diagnosing hyperglycaemia in pregnancy
686. evaluation of the success of medical abortion by a plasma hcg control threshold
687. evaluation of the success of medical abortion by a plasma hcg control threshold; [évaluation du succès de l'interruption volontaire de grossesse médicamenteuse par un seuil d'hcg sérique]
688. evaluation of the teratogenic risks in gestations exposed to misoprostol; [avaliação de riscos teratogênicos em gestações expostas ao misoprostol]
689. evaluation of ultrasound needs in first trimester medical abortion with misoprostol
690. evidence for shortening the time interval of prostaglandin after mifepristone for medical abortion
691. evidencebased practicefact or fiction?
692. evolución cardiológica postnatal y factores asociados a la agenesia de ductus venoso de diagnóstico prenatal
693. exhaustion in myeloid lineage and very early defect in hspc pool an embryonic origin of fanconi haematological disorders
694. expanding a woman's options to include home use of misoprostol for medical abortion up until 76 days an observational study of efficacy and safety
695. experience and acceptability of emergency hormonal contraception
696. experience of clandestine use of medical abortion among university students in chile a qualitative study
697. experience with midtrimester abortion
698. experiences and prophylactic of panic attacks the case of magnetic resonance imaging; [doświadczanie i proflaktyka ataku paniki casus obrazowania rezonansem magnetycznym]
699. experiences of midwives and nurses when implementing abortion policies a systematic integrative review
700. experiences of women living in hungary seeking a medical abortion online
701. experiences with gemeprost in 1sttrimester termination of pregnancy
702. experiences with the progesterone antagonist mifepristone (ru 486) for termination of early pregnancy
703. experts, non experts, and policy discourse a case study of the royal commission on new reproductive technologies
704. exploring the costs and economic consequences of unsafe abortion in mexico city before legalisation
705. exposure to radiation therapy is associated with female reproductive health among childhood cancer survivors a metaanalysis study
706. expulsion and continuation rates after postabortion insertion of framed iuds versus frameless iuds review of the literature
707. expulsion at home for early medical abortion a systematic review with metaanalyses
708. extending outpatient medical abortion services through 70 days of gestational age
709. factors affecting gestational age at termination of pregnancy
710. factors affecting the outcome of early medical abortion a review of 4132 consecutive cases
711. factors affecting the success of repeated misoprostol course for the treatment of missed abortion
712. factors associated with cesarean operations of gestational diabetic mellitus and diabetes complications
713. factors associated with followup rates after medical abortion in adolescents at cook county hospital
714. factors associated with management outcome of incomplete abortion in yirgalem general hospital, sidama zone, southern ethiopia
715. factors associated with severe complications in unsafe abortion
716. factors associated with the failure of medical treatment for ectopic pregnancy case study conducted at the yaoundé gynaecology, obstetrics and pediatrics hospital; [facteurs associés à l’échec du traitement médical de la grossesse extrautérine cas de l’hôpital gynecoobstétrique et pédiatrique de yaoundé]
717. factors associated with the outcomes of preimplantation genetic testing in assisted reproduction; [胚胎种植前遗传学检测助孕妊娠结局的影响因素研究]
718. factors associated with the persistence of retained products following d and c is curettage still the treatment of choice?; [factores que se asocian a la persistencia de restos abortivos tras legrado evacuador sigue siendo el curetaje el tratamiento de eleccion?]
719. factors hindering access to abortion services
720. factors influencing the abortion interval of second trimester pregnancy termination using misoprostol
721. factors influencing the delivery of abortion services in ontario a descriptive study
722. factors related to completeness of medical abortion with mifepristone and misoprostol
723. failed early medical abortion beware of the uterine scar! case report
724. failed early medical abortion beware of the uterine scar!case report
725. failed medical termination of twin pregnancy with mifepristone a case report
726. failure of sterilization after clip placement
727. failure rate of medical treatment for miscarriage correlated with the difference between gestational age according to last menstrual period and gestational size calculated via ultrasound
728. failure to achieve the association of professors in gynecology and obstetrics objectives for abortion in thirdyear medical student curriculum
729. false positive results of tests for syphilis and outcome of pregnancy a retrospective casecontrol study
730. family planning clinics share blame for men's failure to use services
731. family planning in europe
732. farmacologico abortion by means of mifepristone and misoprostol
733. fatal hemorrhage from legal abortion in the united states
734. fatal hemorrhage from legalabortion in the unitedstates
735. fatal necrotizing fasciitis in illegal abortion and the negligence tort
736. fatal septic abortion in the united states, 19751977
737. fatal septic abortion in the unitedstates, 19751977
738. feasibility of a hospital outpatient day procedure for medication abortion at 1318 weeks gestation findings from nepal()
739. feasibility of a selfperformed urinary test for the followup on medical abortion the betina study
740. female rehabilitation after medical abortion
741. fertility and pregnancy outcomes following uterine devascularization for severe postpartum haemorrhage
742. fertility and pregnancy outcomes in females with dyskeratosis congenita
743. fertility following ectopic pregnancy
744. fertility trends in singapore
745. fertility, gestational and systemic problems in turner's syndrome two case reports; [turner sendromunda fertilite, gestasyonel ve sistemik sorunlar iki olgu sunumu]
746. fetal complications associated with unsuccessful attempts at termination of pregnancy
747. fetal complications associated with unsuccessful attempts at termination of pregnancy; [fetale komplikationen nach misslungenem schwangerschaftsabbruch im ersten trimester]
748. fetal cystic hygroma cause and natural history
749. fetal death ratios in a prospective study compared to state fetal death certificate reporting
750. fetal death ratios in a prospectivestudy compared to state fetal death certificate reporting
751. fetal erythrocytes in maternal circulation after spontaneous abortion
752. filum terminale needle placement during caudal epidural steroid injection
753. first pregnancy abortion as an infectious complications risk factor
754. first trimester abortion with mifepristone and vaginal misoprostol
755. first trimester bleeding
756. first trimester medical abortion practice in north east india
757. first trimester medical termination of pregnancy the nottingham experience
758. first trimester termination of pregnancy
759. firsttrimester medical abortion with mifepristone 200 mg and misoprostol a systematic review
760. firsttrimester pregnancy failure
761. firsttrimester spontaneous abortions and the incidence of human immunodeficiency virus seropositivity
762. five years experience of haemodialysis at the lagos university teaching hospitalnovember 1981 to november 1986
763. followup after early medical abortion comparing clinical assessment with selfassessment in a rural hospital in northern norway
764. followup rates and contraceptive choices after medical abortion in adolescents at cook county hospital
765. followup strategies to confirm the success of medical abortion of pregnancies up to 10 weeks’ gestation a systematic review with metaanalyses
766. followup strategies to confirm the success of medical abortion of pregnancies up to 10 weeks' gestation a systematic review with metaanalyses
767. forensic problems in bovine obstetrics and gynecology
768. formulation and noncontraceptive uses of the new, lowdose oral contraceptive
769. four perspectives of women's health workshop participants talk about women's health issues in four countries [hong kong]
770. frequency and risk factors for repeat abortions after surgical compared with medical termination of pregnancy
771. from concept to practice the recent history of preterm delivery prevention part ii subclinical infection and hormonal effects
772. fulminant postpartum cerebral vasoconstriction syndrome
773. further acceptability evaluation of ru486 and ono 802 as abortifacient agents in a chinese population
774. future direction of abortion technology
775. future uncertain as congressional drive to block title x gag rule fails
776. garrison v medical center of delaware, inc, 12 december 1989
777. gastrojejunostomy as successful surgical treatment of acute duodenitis/proximal jejunitis in a mare
778. general practitioner knowledge and practice in relation to unintended pregnancy in the grampians region of victoria, australia
779. genetic findings in early miscarriage analysis by chromosomal microarray and whole exome sequencing
780. gestational trophoblastic disease treatment results at the brewer trophoblastic disease center
781. ghana
782. gnrh‐a combined fertility‐sparing re‐treatment in women with endometrial carcinoma or atypical endomertial hyperplasia who failed to oral progestin therapy; [gnrha 联合治疗用于口服孕激素治疗失败的子宫内膜非典型增生及子宫内膜癌患者的探讨]
783. gonadotrophins for ovulation induction in women with polycystic ovary syndrome
784. gregory v pembrokeshire health authority [31 january 1989]
785. growth hormone for in vitro fertilisation (ivf)
786. has mifepristone medical abortion expanded abortion access in new mexico? a survey of obgyn and family medicine physicians
787. heads up! potential legal obstacles to medical abortion
788. health benefits of legal abortion an analysis
789. healthcare students' knowledge and opinions about the argentinean abortion law
790. high failure rates of medical termination of pregnancy after introduction to a large teaching hospital
791. highdose misoprostol as an alternative therapy after failed medical abortion
792. history of medicine volume 4
793. hodgkin’s disease in pregnancy
794. home selfadministration of misoprostol for medical abortion up to 56 days' gestation
795. home selfadministration of vaginal misoprostol for medical abortion at 50 to 63 days compared with gestation of below 50 days
796. home selfadministration of vaginal misoprostol for medical abortion at 5063 days compared with gestation of below 50 days
797. homocysteine serum concentration and uterine artery color doppler examination in cases of recurrent miscarriages with unexplained etiology
798. homocysteine, folic acid and vitamin b12 concentration in patients with recurrent miscarriages
799. homocysteine, folic acid and vitamin b12 concentration in patientswith recurrent miscarriages
800. honour and interests medical ethics in britain and the work of the british medical association's central ethical committee, 19021939 (bl dxn050009)
801. honour and interests medical ethics in britain, and the work of the british medical association's central ethical committee, 19021939
802. how antiabortion legislation would put doctors in a bind
803. how family planning can save lives in africa
804. how safe motherhood in india is
805. how the coronavirus disease 2019 pandemic is impacting sexual and reproductive health and rights and response results from a global survey of providers, researchers, and policymakers
806. human assisted reproduction
807. human rights, maternal mortality and reproductive health; [derechos humanos, mortalidad materna y salud reproductiva]
808. hummel v reiss
809. hydatidiform moles among patients with incomplete abortion in mwanza city, north western tanzania
810. hyperosmolar urea for elective midtrimester abortion experience in 1,913 cases
811. hypertonic salineinduced abortion complicated by consumptive coagulopathy a case report
812. hypoplastic left heart syndrome 19 years of prenatal diagnosis
813. hysteroscopic diagnosis of omentum incarceration subsequent to an iatrogenic uterine perforation
814. hysteroscopic findings after missed abortion
815. hysteroscopic removal of intrauterine contraceptive devices with missing threads
816. hysteroscopic resection or conventional blind curettage in the management of retained products of conception, a systematic review
817. i did not plan to have a baby this is the outcome of our work a qualitative study exploring unintended pregnancy among female sex workers
818. i will never wish this pain to even my worst enemy lived experiences of pain associated with manual vacuum aspiration during postabortion care in kenya
819. ibuprofen and paracetamol for pain relief during medical abortion a doubleblind randomized controlled study
820. identifying national availability of abortion care and distance from major us cities systematic online search
821. if you were to have another abortion, would you choose the same method? a study on 1032 patients’ level of satisfaction
822. illegal abortion with misoprostol in guadeloupe
823. illegal abortion with misoprostol in guadeloupe; [avortements illégaux par le misoprostol en guadeloupe]
824. imaging and imagining the fetus
825. immediate postabortion intrauterine contraception in nulliparous adolescents
826. immediate versus delayed insertion of an etonogestrel releasing implant at medical abortion a randomized controlled equivalence trial
827. immediate versus delayed insertion of an etonogestrel releasing implant at medical abortiona randomized controlled equivalence trial
828. immunological aspects of pregnancy failures novel therapeutic approaches; [sikertelen terhességek immunológiai háttere újabb terápiás lehetoségek]
829. impact of prolonged use of adjuvant tocolytics after cervical cerclage on late abortion and premature delivery
830. impact of the covid19 pandemic on the prevalence of mtp cases and their clinicodemographic profile in india a retrospective multicentric study
831. impact of the introduction of new medical methods on therapeutic abortions at the royal infirmary of edinburgh
832. impact of the supreme court decisions on the performance of abortions in the united states
833. impacto del estrés laboral en el anestesiólogo
834. impacts of delivery mode and maternal factors on neonatal oral microbiota
835. implantable cardioverterdefibrillators and pregnancy a safe combination?
836. implantable cardioverterdefibrillators and pregnancy a safe combination?
837. implication of sperm chromosomal abnormalities in recurrent abortion and multiple implantation failure
838. implications for gynecological services of new medical methods of therapeutic abortion
839. importance of a second dose of misoprostol in the management of medical abortion after failure of the initial treatment
840. importance of a second dose of misoprostol in the management of medical abortion after failure of the initial treatment; [intérêt de l'administration d'une seconde dose de misoprostol dans la prise en charge de l'interruption volontaire de grossesse après échec du traitement médical initial]
841. improving safe postabortion care practices a study on interventions implemented by ipas pakistan
842. improving the quality of care after spontaneous abortions in rural senegal; [amélioration de la qualité des soins après avortement (saa) en zone rurale au sénégal]
843. incidence of diandric triploidy (partial mole) in misscarriages
844. incidence of induced abortion determined by the randomised response technique
845. increased risk for medical abortion failure for multiparous women
846. increased transient tachypnea of the newborn in infants of asthmatic mothers
847. increasing women's choices in medical abortion a study of misoprostol 400 microg swallowed immediately or held sublingually following 200 mg mifepristone
848. increasing women's choices in medical abortion a study of misoprostol 400 μg swallowed immediately or held sublingually following 200 mg mifepristone
849. india's missing daughters an ominous sign for democracy
850. induced abortion physician training and practice patterns
851. induced abortion in the first trimester of pregnancy and repeat pregnancy in adolescents
852. induced abortion a vulnerable public health problem; [el aborto inducido un problema de salud publica vulnerable]
853. induced abortion incidence and trends worldwide from 1995 to 2008
854. induced abortion physician training and practice patterns
855. induced abortions in khamano block of fatehgarh sahib district, punjab
856. inducedabortion among unmarried women in sichuan province, china
857. inducedabortion in kenya casehistories
858. inducedabortion in maternal and child health centers in a general familyplanning program in cap bon, tunisia
859. induction of abortion during the 2nd trimenon of pregnancy endocervical pge2 gel application, intramuscular application of sulproston and combined treatment (endocervical pge2 gel intramuscular sulproston)
860. induction of abortion during the second trimenon of pregnancy endocervical pge2 gel application, intramuscular application of sulproston and combined treatment (endocervical pge2 gel/intramuscular sulproston); [abortinduktion im zweiten schwangerschaftstrimenon endozervikale pge2gelapplikation, intramuskulare sulprostonapplikation und kombinierte (endozervikale pge2gel/intramuskulare sulproston) behandlung]
861. induction of abortion in earlypregnancy with mifepristone in conjunction with gemeprost
862. induction of abortion in the second trimester of pregnancy by intramuscular 15methyl prostaglandin f 2 alpha (prostin 15m)
863. induction of abortion in the second trimester of pregnancy by intramuscular 15methyl prostaglandin f(2α) 15m)
864. induction of abortion with mifepristone and misoprostol in early pregnancy
865. induction of abortion with mifepristone and misoprostol in earlypregnancy
866. inequalities in access to and quality of abortion services in mexico can tasksharing be an opportunity to increase legal and safe abortion care?
867. infectious agents identified in aborted swine fetuses in a highdensity breeding area a threeyear study
868. infertility today the management of female medical causes
869. influence of two endometrial preparation programs independent of endogenous ovarian cycle on the pregnancy outcome of thin endometrial patients with freezethaw embryo transfer; [两种非依赖内源性卵巢周期的内膜准备方案对薄型子宫内膜冻融胚胎移植患者妊娠结局的影响研究]
870. informed consent or institutionalized eugenics? how the medical profession encourages abortion of fetuses with down syndrome
871. initiating intramuscular depot medroxyprogesterone acetate 2448 hours after mifepristone administration does not affect success of early medical abortion
872. injectable contraception
873. instructiononly versus demonstration of a low sensitivity pregnancy test for selfassessment of medical abortion in south africa; a multicentre noninferiority randomised controlled trial
874. integrating gender perspectives in gynecology and obstetrics engaging medical colleges in maharashtra, india
875. integrating medical abortion into safe abortion services experience from three pilot sites in south africa
876. integrating mobile phones into medical abortion provision intervention development, use, and lessons learned from a randomized controlled trial
877. integrative transcriptomics and proteomics analyses to reveal the therapeutic effect and mechanism of buxue yimu pills in medicalinduced incomplete abortion rats
878. interpretation to medication abortion up to 70 days of gestation • acog practice bulletin , number 225; [2020 agog实践简报孕龄<70 d药物 流产(no 225)》解读]
879. interregional project concerning abortion; [projet interregional sur l'ivg]
880. interrupção da gestação por opção na póvoa de varzim análise e perspectivas
881. interruption of nonviable pregnancies of 2428 weeks' gestation using medical methods release date june 2013 sfp guideline #20133
882. intraabdominal hemorrhage induced by medical abortion in midtrimester pregnancy an unusual presentation of placenta percreta
883. intraamniotic injection of hypertonic solution as a method for termination of pregnancy
884. intracytoplasmic sperm injection versus conventional in vitro fertilisation in couples with males presenting with normal total sperm count and motility
885. intrauterine administration of hcg immediately after oocyte retrieval and the outcome of icsi a randomized controlled trial
886. intrauterine contraception after medical abortion factors affecting success of early insertion
887. intrauterine devices in the immediate, early and late postabortion period; [dispositivos intrauterinos en el postaborto inmediato, precoz y tardío]
888. intravaginal misoprostol for medical evacuation of first trimester missed abortion
889. introducing medical abortion in turkey perceptions of physicians
890. introducing medical abortion in turkey perspectives of nurses/midwives
891. introducing medical abortion in turkey perspectives of physicians
892. introduction of early medical abortion in new zealand an audit of the first 67 cases
893. introduction menstrual regulation the method and the issues
894. invasive management of proximal ureteral calculi during pregnancy
895. investigating the reasons of preterm labor among visitors of shariati hospital in bandar abbas during 2012 and 2013
896. investigation and treatment of amenorrhoea resulting in normal fertility
897. investigation of hepatitis b virus seroprevalence in hepatitis c infected patients; [hepatit c virüsü ile enfekte hastalarda hepatit b seroprevalansinin araştirilmasi]
898. is a cleft lip and palate a serious "handicap"? jepson v chief constable of west merciaa legal and ethical critique
899. is it safe to provide abortion pills over the counter? a study on outcome following selfmedication with abortion pills
900. is mifepristone 100mg an effective alternative to standard dose for medical abortion
901. is mifepristone 100mg an effective alternative to standard dose for medical abortion; [tıbbi abortus için 100 mg mifepriston standart doza etkili bir alternatif midir?]
902. is preimplantation genetic diagnosis the ideal embryo selection method in aneuploidy screening?
903. is selfassessment of medical abortion using a lowsensitivity pregnancy test combined with a checklist and phone text messages feasible in south african primary healthcare settings? a randomized trial
904. is selfmedication with over the counter abortion pills really safe? an experience at a tertiary care centre
905. is sexselective abortion against the law?
906. is therapeutic abortion preventable?
907. is ward evacuation for uncomplicated incomplete abortion under systemic analgesia safe and effective a randomized clinicaltrial
908. is ward evacuation for uncomplicated incomplete abortion under systemic analgesia safe and effective? a randomised clinical trial
909. issues in second trimester induced abortion (medical/surgical methods)
910. it’s a small bit of advice, but actually on the day, made such a difference… perceptions of quality in abortion care in england and wales
911. it's a small bit of advice, but actually on the day, made such a difference… perceptions of quality in abortion care in england and wales
912. judgment, 28 january 1987
913. kisspeptin a potential factor for unexplained infertility and impaired embryo implantation
914. knowledge and use of secondary contraception among patients requesting termination of pregnancy
915. lamicel is more effective in preparing the cervix for midtrimester termination of pregnancy than laminaria japonicum
916. laparoscopic management of caesarean scar ectopic pregnancy a report of 2 cases
917. laparoscopic management of caesarean scar ectopic pregnancya report of 2 cases
918. laparoscopic sterilisation opinion and practice among gynaecologists in scotland
919. laparoscopic tubal sterilization coincident with therapeutic abortion by suction curettage
920. laparoscopic tubalsterilization using yoons rings the technique and the psychological effects
921. large variation in clinical regimens used to induce medical abortion in denmark
922. late midtrimester medical pregnancy terminations 3 different procedures with prostaglandinf2alpha and laminaria tents
923. late midtrimester medical pregnancy terminations three different procedurew with prostaglandin f2α and laminaria tents
924. lateterm abortion and medical necessity a failure of science
925. latin american hospitals improve postabortion care maternal health
926. launch, drift, and withdrawal of public policy a case study of the korean medical school system
927. legal duties to respect abortion choices
928. legal issues in the provision of medical abortion
929. legal limits relaxed time to look at other barriers faced by women seeking termination of pregnancy for fetal anomalies
930. leptospirosis seropositivity and its serovars among cattle in northeastern malaysia
931. lessons learned from introducing dilation and evacuation in tigray region of ethiopia
932. level of realization of reproductive potential in women of different age
933. life and death issues in bioethics abortion, persistent vegetative state, and the definition of death
934. lifethreatening complications of mtp/abortion
935. longterm outcome of lupus nephritis a single center study
936. lowdose mifepristone followed by vaginal misoprostol at 48 hours for abortion up to 63 days
937. lowering the doses of mifepristone and gameprost for early abortion a randomised controlled trial world health organization task force on postovulatory methods for fertility regulation
938. lowering the doses of mifepristone and gemeprost for early abortion a randomised controlled trial
939. lownganonglevine syndrome in a 3monthold infant with isolated left ventricular noncompaction
940. lowsensitivity urine pregnancy testing to assess medical abortion outcome a systematic review
941. lupus erythematodes associated with pregnancy (a death case after delivery)
942. malaria in pregnancy
943. malaria in pregnancy clinicalfeatures and outcome of treatment
944. male contraception; [la contraception masculine]
945. male experiences of unintended pregnancy characteristics and prevalence
946. malpractice issues in radiology wrongful life
947. management and treatment of brain tumors during pregnancy an italian survey
948. management of cases of abortion
949. management of gestational trophoblastic tumours a fiveyear clinical experience
950. management of incomplete abortion with manual vacuum aspiration in comparison to sharp metallic curette in an ethiopian setting
951. management of incomplete and missed spontaneous abortions a cohort study of trends in calgary emergency departments
952. management of incomplete spontaneous abortion with suction curettage in the pediatric emergency department
953. management of maternal amanita phalloïdes poisoning during the first trimester of pregnancy a case report and review of the literature
954. management of maternal amanita phalloides poisoning during the first trimester of pregnancy a case report and review of the literature
955. management of pain associated with upto9weeks medical termination of pregnancy (mtop) using mifepristonemisoprostol regimens expert consensus based on a systematic literature review
956. management of pheochromocytoma during pregnancy about three cases
957. management of pregnancy and delivery in women with transposition of the great arteries after atrial switch operation a 16year singlecenter experience
958. management of septic abortion
959. management of the uterine abnormalities on the reproductive outcomes in women with repeated implantation failure; [宫腔异常治疗对反复种植失败患者助孕结局的影响]
960. mandatory parental involvement/judicial bypass laws do they promote adolescents' health?
961. manual vacuum aspiration saves lives
962. manual vacuum aspiration technique draws interest
963. manual vacuum aspiration a safe and costeffective substitute of electric vacuum aspiration for the surgical management of early pregnancy loss
964. massachusetts medical society
965. maternal activity in relation to birth size in rural india the pune maternal nutrition study
966. maternal and fetal outcomes of subsequent pregnancies in women with peripartum cardiomyopathy
967. maternal and fetal outcomes with aortic dissection in pregnant patients with marfan syndrome
968. maternal and fetal prognosis of subsequent pregnancy in black african women with peripartum cardiomyopathy
969. maternal and infant followup after total cervical occlusion
970. maternal and neonatal listeriosis report of case and brief review of literature of listeriosis in man
971. maternal and perinatal outcome of maternal obesity at rscm in 20142019
972. maternal fontan procedure is a predictor of a smallforgestationalage neonate a 10year retrospective study
973. maternal heart disease and pregnancy outcome a single
974. maternal level of pregnancyassociated plasma protein a as a predictor of pregnancy failure in threatened abortion
975. maternal mortality and morbidity associated with clandestine abortions
976. maternal mortality at the university of benin teaching hospital benin city, nigeria
977. maternal mortality at the university of nigeria teaching hospital, enugu a 10year survey
978. maternal mortality in a semiurban nigerian community
979. maternal mortality in hospitals in zululand, july 1993 june 1994
980. matsubarayano suture a simple uterine compression suture for postpartum hemorrhage during cesarean section
981. media reviews
982. medical abortion
983. medical abortion a life saving technology
984. medical abortion after 9 weeks
985. medical abortion as an alternative to vacuum aspiration first experiences with the 'abortion pill' in the netherlands
986. medical abortion at 57 to 63 days' gestation with a lower dose of mifepristone and gemeprost a randomized controlled trial
987. medical abortion at 57 to 63 days' gestation with a lower dose of mifepristone and gemeprost a randomized controlled trial
988. medical abortion at 63 to 90 days of gestation
989. medical abortion at 64 to 91 days of gestation a review of 483 consecutive cases
990. medical abortion at 912 weeks
991. medical abortion at 913 weeks' gestation a review of 1076 consecutive cases
992. medical abortion at home and at hospital a trial of efficacy and acceptability
993. medical abortion complications an epidemiologic study at a midmissouri clinic
994. medical abortion followup with serum human chorionic gonadotropin compared with ultrasonography a randomized controlled trial
995. medical abortion in china
996. medical abortion in family practice a case series
997. medical abortion in rural tamil nadu, south india a quiet transformation
998. medical abortion in second trimester missed abortion pregnancies
999. medical abortion in the community setting
1000. medical abortion in the first trimester
1001. medical abortion in the second trimester an update
1002. medical abortion in women of ≤56 days amenorrhoea a comparison between gemeprost (a pge(1) analogue) alone and mifepristone and gemeprost
1003. medical abortion in women of ≤56 days amenorrhoea; a comparison between gemeprost (a pge1 analogue) alone and mifepristone and gemeprost
1004. medical abortion in women of less than or equal to 56 days amenorrhoea a comparison between gemeprost (a pge1 analogue) alone and mifepristone and gemeprost
1005. medical abortion in women of lessthanorequalto56 days amenorrhea a comparison between gemeprost (a pge(1) analog) alone and mifepristone and gemeprost
1006. medical abortion offered in pharmacy versus clinicbased settings
1007. medical abortion outcomes after a seconddose of misoprostol is the presence of cardiac motion on followup ultrasonography predictive of failure?
1008. medical abortion outcomes and repeat pregnancy following quickstart of contraceptive implants and depotmedroxyprogesterone acetate
1009. medical abortion outcomes following quickstart of contraceptive implants and depotmedroxyprogesterone acetate
1010. medical abortion provided by nursemidwives or physicians in a high resource setting a costeffectiveness analysis
1011. medical abortion self use in kenya results from a process evaluation of women's experiences
1012. medical abortion using methotrexate and misoprostol efficacy and tolerability
1013. medical abortion with home administration of misoprostol up to 63 days gestation
1014. medical abortion with methotrexate 75 mg intramuscularly and vaginal misoprostol
1015. medical abortion with mifepristone and home administration of misoprostol up to 63 days' gestation
1016. medical abortion with mifepristone and home administration of misoprostol up to 63 days' gestation
1017. medical abortion with mifepristone and misoprostol a clinical trial in taiwanese women
1018. medical abortion with mifepristone and vaginal misoprostol between 64 and 70 days' gestation
1019. medical abortion with mifepristone and vaginal misoprostol between 64 and 70days' gestation
1020. medical abortion defining success and categorizing failures
1021. medical abortion a practical quick reference algorithm to support the gynecologists
1022. medical abortion clinical aspects
1023. medical abortion public health and private lives
1024. medical abortion use of mifepristone and misoprostol in first and second trimesters of pregnancy
1025. medical abortion what does the research tell us?
1026. medical abortionlet us give quality care! making medical abortion safer and more effective in low resource population a comparative study from private hospital, nagpur india
1027. medical and social aspects of adolescent pregnancies i adolescents applying for termination of an illegitimate pregnancy
1028. medical and social problems and attitudes to optional abortions in bulgaria
1029. medical claims and women's experience physicianperformed abortions in the weimar republic; [heilanspruch und medizinische kunstfehler abtreibungen durch arzte in der weimarer republik offizielle beurteilung und weibliche erfahrung]
1030. medical developments and religious belief with special reference to europe in the 18th and 19th centuries
1031. medical evacuation of 1st trimester (12 weeks gestation) incomplete abortion and missed abortion
1032. medical evacuation of first trimester (twelve weeks gestation) incomplete abortion and missed abortion
1033. medical genetics in reproductive medicine; [vyuzití lékarské genetiky v reprodukcní medicíne]
1034. medical management of firsttrimester abortion
1035. medical management of induced and incomplete firsttrimester abortion by nonphysicians in low and middleincome countries a systematic review and metaanalysis of randomized controlled trials
1036. medical management of missed abortion a randomized clinical trial
1037. medical methods for first trimester abortion
1038. medical methods for midtrimester termination of pregnancy
1039. medical methods to terminate early pregnancy
1040. medical methods to terminate earlypregnancy
1041. medical referral for abortion and freedom of conscience in australian law
1042. medical responsibility in the practice of voluntary first trimester terminations of pregnancy preventive measures drawn from jurisprudence and from the study of 50 files of legal action or threatened legal action
1043. medical responsibility in the practice of voluntary first trimester terminations of pregnancy preventive measures drawn from jurisprudence and from the study of 50 files of legal action or threatened legal action; [responsabilite medicale dans la pratique des interruptions volontaires de grossesse du premier trimestre mesures preventives tirees de la jurisprudence et de l'etude de 50 dossiers de plaintes ou de menaces de plaintes]
1044. medical student awareness of sexual health is poor
1045. medical students and controversial ethical issues results from the multicenter study sbrame
1046. medical students' intentions to seek abortion training and to provide abortion services in future practice
1047. medical termination for pregnancy in early first trimester (≤ 63 days) using combination of mifepristone and misoprostol or misoprostol alone a systematic review
1048. medical termination of pregnancy and concurrent contraceptive adoption in a tertiary referral hospital in delhi
1049. medical termination of pregnancy during the second versus the first trimester and its effects on subsequent pregnancy
1050. medical termination of pregnancy in the second trimester
1051. medical termination of pregnancy with mifepristone initial experience at the sheba medical center
1052. medical termination of pregnancy a study of acceptor characteristics
1053. medical treatment for missed abortion difference in gestational age based on menstrual dating and on ultrasound criteria, and the correlation to failure
1054. medical treatment of spontaneous abortion in the first trimester; [tratamiento médico del aborto espontáneo del primer trimestre]
1055. medical treatment with misoprostol for early failure of pregnancies after assisted reproductive technology a promising treatment option
1056. medicallyinduced abortion and risk of reproductive failures in subsequent pregnancy; [medicinsk induceret abort og risiko for bivirkninger ved en efterfølgende graviditet sekundærpublikation]
1057. medicalmanagement of miscarriage nonsurgical uterine evacuation of incomplete and inevitable spontaneousabortion (vol 306, pg 894, 1993)
1058. medicalsocial aspects pregnancy, labor and puerperium in patients the solitary mother home in karwowo near szczecin; [medycznospołeczne aspekty przebiegu ciazy, porodu i połogu u pacjentekpensjonariuszek domu samotnej matki w karwowie koło szczecina]
1059. medication abortion
1060. meeting women's need for a flexible abortion service retrospective study of a specialist daycare unit
1061. menstrual induction with mifepristone and misoprostol
1062. menstrual regulation in family planning services
1063. menstrual regulation using medication is acceptable and feasible in bangladesh
1064. methods for induced abortion
1065. methotrexate and misoprostol for early abortion in adolescent women
1066. methotrexate and misoprostol teratogenicity further expansion of the clinical manifestations
1067. methotrexate and misoprostol used alone or in combination for early abortion
1068. methotrexate and misoprostol vs misoprostol alone for early abortion a randomized controlled trial
1069. methotrexate compared with mercaptopurine for early induced abortion
1070. methotrexate/misoprostol embryopathy report of four cases resulting from failed medical abortion
1071. microbiology relevant to recurrent miscarriage
1072. midgestational abortion for medical or genetic indications
1073. midtrimester abortion by dilatation and evacuation a safe and practical alternative
1074. midtrimester abortion with intramuscular injection of 15methylprostaglandin f2alpha
1075. midtrimester medical termination of pregnancy a review of 1002 consecutive cases
1076. midtrimester pregnancy termination in teenage women
1077. midwives, nurses and doctors performing mva in vietnam and south africa and early medical abortion in nepal evidence from randomized, controlled trials
1078. mifepristone (ru 486) — an abortifacient to prevent abortion?
1079. mifepristone (ru 486) compared with highdose estrogen and progestogen for emergency postcoital contraception
1080. mifepristone (ru 486) current knowledge and future prospects
1081. mifepristone (ru486) compared with highdose estrogen and progestogen for emergency postcoital contraception
1082. mifepristone 100mg for early medical abortion
1083. mifepristone abortion in minors
1084. mifepristone and misoprostol administered simultaneously versus 24 hours apart for abortion a randomized controlled trial
1085. mifepristone and misoprostol administered simultaneously versus 24 hours apart for abortion a randomized controlled trial
1086. mifepristone followed by home administration of buccal misoprostol for medical abortion up to 70 days of amenorrhoea in a general practice in curacao
1087. mifepristone followed by home administration of buccal misoprostol for medical abortion up to 70 days of amenorrhoea in a general practice in curaçao
1088. mifepristone in mid trimester termination of pregnancyvalue for money?
1089. mifepristone in midtrimester termination of pregnancy value for money
1090. mifepristone plus vaginal misoprostol vs vaginal misoprostol alone for medical abortion in gestation 63 days or less in nepalese women a quasirandomized controlled trial
1091. mifepristone with buccal misoprostol for medical abortion
1092. mifepristone with buccal misoprostol for medical abortion a systematic review
1093. mifepristone ten years later
1094. mifepristonemisoprostol abortion a trial in rural and urban maharashtra, india
1095. minimal effective dose of mifepristone for medical abortion
1096. minors' behavioral responses to parental involvement laws delaying abortion until age 18
1097. misconceptions monstrosity and the politics of interpretation in american culture from the antinomian controversy to biotechnology
1098. misdiagnosis of ectopic pregnancy importance of ultrasonography prior to early termination of pregnancy
1099. mish publishes new framework for fearbased, abstinenceonly education
1100. misoprostol (cytotec8)exposure pregnancy a french coijaborative study
1101. misoprostol abortion ultrasonography versus betahcg testing for verification of effectiveness
1102. misoprostol alonea new method of medical abortion?
1103. misoprostol and pregnancy risk of malformations
1104. misoprostol as the primary agent for medical abortion in a lowincome urban setting
1105. misoprostol dose and route after mifepristone for early medical abortion a randomised controlled noninferiority trial
1106. misoprostol for second trimester medical abortion a comparison of three routes of administration
1107. misoprostol for termination of second trimester pregnancy in a scarred uterus
1108. misoprostol for women's health a review
1109. misoprostol use as a method of medical abortion
1110. misoprostol use in obstetrics and gynecology in brazil, jamaica, and the united states
1111. misoprostol versus suction evacuation in the management of patients with incomplete abortion a scoping review
1112. misoprostolinduced acute coronary syndrome in a premenopausal woman a case report with literature review
1113. misoprostolinduced fever and unnecessary antibiotic prescribing a retrospective study
1114. misoprostolinduced termination of secondtrimester pregnancy in women with a history of cesarean section a retrospective analysis of 56 cases
1115. modern indications for abortion
1116. modern methods to induce abortion safety, efficacy and choice
1117. modern techniques of medical pregnancy termination; [les techniques modernes d'interruption medicale de la grossesse]
1118. moebius syndrome due to the use of misoprostol case report; [sindrome de moebius fetopatía por misoprostol reporte de un paciente]
1119. monitoring medical abortion using mifepristone/misoprostol combination with ultrasonogram and serum human chorionic gonadotropin
1120. morbidity of therapeutic abortion in auckland
1121. morbidity that is associated with curettage for the management of spontaneous and induced abortion in women who are infected with hiv
1122. mortality associated with hypertonic saline abortion
1123. motives for selfinduced abortion among women in alexandria an exploratory study
1124. multicenter trial of a simplified mifepristone medical abortion regimen
1125. must a catholic hospital inform a rape victim of the availability of the morningafter pill
1126. must a catholic hospital inform a rape victim of the availability of the 'morningafter pill'?
1127. myometritis with pelvic septic vein thrombophlebitis secondary to fusobacterium necrophorum sepsis
1128. new evidence on induced abortion in tehran, iran rates, causes, and changes
1129. new frontiers in abortion care
1130. new york appeals court finds state not liable for death after physician under review performed abortion
1131. nondeveloping pregnancy histological and immunohistochemical markers of endocrine disorders in endometrial scrapes
1132. nonphysician clinicians can safely provide first trimester medical abortion
1133. normal and abnormal early pregnancy
1134. north florida women's health services v state
1135. not every subseptate uterus requires surgical correction to reduce poor reproductive outcome
1136. nurse versus physicianprovision of early medical abortion in mexico a randomized controlled noninferiority trial
1137. observations on abortion in zambia
1138. observations on female sterilization in chile
1139. observations on trypanosomiasis in the belgian congo
1140. obstetric and gynecologic dysfunction in the ehlersdanlos syndrome
1141. obstetric and perinatal outcome of teenage pregnancy
1142. obstetric determinants of neonatal survival antenatal predictors of neonatal survival and morbidity in extremely low birth weight infants
1143. occurrence and determinants of psychological distress among women undergoing abortion/medical termination of pregnancy
1144. of human rights and women's health
1145. office management of early pregnancy loss
1146. office versus telephone followup after medical abortion
1147. on inhuman practices in gynaecology and their victims in germany during national socialist rule a study of concrete events; [uber inhumane praktiken der frauenheilkunde im nationalsozialismus und ihre opfer untersuchung zu konkreten ereignissen]
1148. on inhuman practices in gynecology and their victims in germany during nationalsocialist rule a study of concrete events
1149. oncologic and fertility outcomes after simple trachelectomy in women with early cervical cancer
1150. online availability of mifepristone and misoprostol
1151. opinion and use of contraceptives among medical students of the university of nigeria, enugu campus
1152. options for early therapeutic abortion a comparative review
1153. options in making use of pregnancy history in planning and analysing studies of reproductive failure
1154. options in making use of pregnancy history in planning and analyzing studies of reproductive failure
1155. oral contraception noncompliance the extent of the problem
1156. oral methotrexate and vaginal misoprostol for early abortion
1157. oral misoprostol administration in the first and second trimester for termination
1158. oriéntame preventing and solving problems related to unwanted pregnancy for 25 years in colombia
1159. orientame preventing and solving problems related to unwanted pregnancy for 25 years in colombia
1160. outbreaks of porcine reproductive failure report on a collaborative field investigation
1161. outcome of first trimester induced abortions using misoprostol by buccal and vaginal routes
1162. outcome of first trimester medical termination of pregnancy definitions and management
1163. outcome of firsttrimester exposure to lowdose methotrexate in eight patients with rheumatic disease
1164. outcome of managing second trimester post abortion cases using a standardized combination of regimen
1165. outcome of noac exposure during pregnancy ( and the problem of event reporting )
1166. outcome of pregnancy in takayasu arteritis
1167. outcomes during early implementation of mifepristonebuccal misoprostol abortions up to 63 days of gestation in a canadian clinical setting
1168. outcomes of legal induced abortion at 910 weeks ' gestation in adult women
1169. outpatient mifepristonemisoprostol medical abortion through 77 days of gestation
1170. outpatient mifepristonemisoprostolmedical abortion through 77 days of gestation
1171. ovarian function in systemic lupus erythematosus patients undergoing the use of cyclophosphamide in two major rheumatologic care centers in curitiba, paraná state; [função ovariana em mulheres lúpicas submetidas ao uso de ciclofosfamida em dois grandes centros de atendimento reumatológico de curitiba, estado do paraná]
1172. pain assessment during medical abortion up to 14 weeks a 1year prospective comparative study
1173. parity is a major determinant of success rate in medical abortion a retrospective analysis of 3161 consecutive cases of early medical abortion treated with reduced doses of mifepristone and vaginal gemeprost
1174. parsing the qmarkers of baoyin jian to treat abnormal uterine bleeding by highthroughput chinmedomics strategy
1175. partialbirth abortion, congress, and the constitution
1176. parvovirus b19 infection frequency in placenta of fetal loss cases in children medical center, tehran, iran
1177. pathopharmacology of excessive hemorrhage in mifepristone abortions
1178. pathophysiological and clinical aspects of combat anticholinesterase poisoning
1179. pathophysiology of mifepristoneinduced septic shock due to clostridium sordellii
1180. pattern of congenital heart disease among egyptian children a 3year retrospective study
1181. pediatrics
1182. perampanel and pregnancy
1183. pérdida recurrente del embarazo e hipertensión pulmonar idiopática
1184. periabortion care when is it too much and when too little?
1185. pericentric inversion of human chromosome 9 epidemiology study in czech males and females
1186. perinatal infection with the human immunodeficiency virus; [perinatalna infekcija virusom humane imunodeficijencije]
1187. perinatal outcomes after organ preserving surgery in patient with ovarian cancer
1188. perinatal prophylaxis with immunoglobulin antid and the impact on rhd sensitizations among pregnant women in slovenia; [perinatalna zaščita z imunoglobulinom antid in vpliv senzibilizacije na antigen rhd med nosečnicami v sloveniji]
1189. personal accounts of 'nearmiss' maternal mortalities in kampala, uganda
1190. pharmacological voluntary interruption of pregnancy in a rural area; [seguridad en la interrupción voluntaria del embarazo farmacológica en un entorno rural]
1191. pharmacy dispensing of abortion pills in ghana experiences of pharmacy workers and users
1192. pharmacy workers in nepal can provide the correct information about using mifepristone and misoprostol to women seeking medication to induce abortion
1193. phase iii clinical trial with norplant ii (two covered rods) report on five years of use
1194. pitfalls in the diagnosis of infection following medical termination of pregnancy
1195. placenta accreta a cause of failed medical abortion
1196. placenta previa; prevalence, risk factor and outcome
1197. placental abnormalities in equine pregnancies generated by scnt from one donor horse
1198. placental polyp an unusual hysteroscopic finding
1199. planned parenthood continues prescribing birth control for teens
1200. planned parenthood of rocky mountains v owens
1201. planned parenthood of southern arizona v lawall
1202. polidocanol foam for nonsurgical permanent female contraception initial trial in baboons
1203. post abortion syndrome myth or reality?
1204. post legalisation challenge minimizing complications of abortion
1205. postabortion care ethical and legal duties
1206. postabortion contraception method mix and uptake in ipassupported public health facilities in ethiopia
1207. postabortion counseling; [l'entretien postivg]
1208. postcoital contraception some characteristics of women who use this method
1209. postcoital contraception a delicate political issue
1210. postcoital contraception a report from the london brook advisory centres, united kingdom
1211. postnatal cardiac outcomes associated with the prenatal diagnosis of absence of ductus venosus; [evolución cardiológica postnatal y factores asociados a la agenesia de ductus venoso de diagnóstico prenatal]
1212. postpartum contraception in china status, problems and coping strategies; [我国产后避孕现状和面临的问题及应对策略]
1213. potential use of single measurement of serum progesterone in detecting early pregnancy failure
1214. practice bulletin no 143 medical management of firsttrimester abortion
1215. practice of unsafe abortion in pakistan characteristics and outcome
1216. practices and knowledge of female gynecologists regarding contraceptive use a realworld chinese survey
1217. predicting poor compliance with followup and intrauterine contraception services after medical termination of pregnancy
1218. prediction of late failure after medical abortion from serial betahcg measurements and ultrasonography
1219. prediction of late failure after medical abortion from serial βhcg measurements and ultrasonography
1220. predictive value of peripheral blood α1acid glycoprotein in medical abortion outcomes with mifepristone and relativity of concentration
1221. predictors of pregnancy outcome in essential thrombocythemia a single institution study of 63 pregnancies
1222. preemptive effect of ibuprofen versus placebo on pain relief and success rates of medical abortion a doubleblind, randomized, controlled study
1223. pregnancies, growth and development of children conceived by subzonal injection of spermatozoa
1224. pregnancy after heart valve replacement
1225. pregnancy after heartvalve replacement
1226. pregnancy and cardiac surgery with cardiopulmonary bypass; [terhesség és nyitott szívmutét]
1227. pregnancy and lupus nephritis
1228. pregnancy and systemic lupuserythematosus
1229. pregnancy complications in sickle cell disease are more prevalent in women with vasoocclusion related organ damage than hemolysis related organ damage
1230. pregnancy course in patients with interstitial lung diseases
1231. pregnancy following uterine artery embolization with polyvinyl alcohol particles for patients with uterine fibroid or adenomyosis
1232. pregnancy in antiphospholipid syndrome outcomes and risk factorsdata from a portuguese multidisclinary unit
1233. pregnancy in pateints with exstrophyepispadias complex are higher rates of complications and spontaneous abortion inevitable?
1234. pregnancy in patients with prosthetic and homograft heart valves maternal and fetal outcome
1235. pregnancy in wilson's disease management and outcome
1236. pregnancy outcome after suicide attempt by drug use a danish populationbased study
1237. pregnancy outcome in women exposed to diethylstilbestrol inutero
1238. pregnancy outcome in women with a history of congenital heart disease a case series study
1239. pregnancy outcomes in antiphospholipid syndrome 8 yearexperience from a multidisciplinary unit
1240. pregnancy outcomes in multiple sclerosis patients previously treated with cyclophosphamide
1241. pregnancy rates and outcomes of hivinfected women in korea
1242. pregnancy related acute renal failure still a major problem
1243. pregnancy termination techniques, risks, and complications and their management
1244. pregnancy termination in a female with paraplegia a case report
1245. pregnancy toxemia and lipid mobilization syndrome in two alpaca (vicugna pacos) at 6 and 10 months of gestation
1246. pregnancy, fertility, and recurrence risk in corrected tetralogy of fallot
1247. pregnancy the effect of dose of mifepristone and gestation on the efficacy of medical abortion with mifepristone and misoprostol
1248. preimplantation genetic screening among women experiencing recurrent failure of in vitro fertilization
1249. preliminary results of the role of semiquantitative pregnancy tests in medical abortion provision
1250. prenatal diagnosis and late termination a legal perspective
1251. prenatal risk factors for optic nerve hypoplasia
1252. prescribing of ergometrine for abortion without physical examination; [voorschrijven van ergometrine wegens abortus zonder onderzoek]
1253. prescription for profit how doctors defraud medicaid
1254. presenting features of women with uterine arteriovenous malformations
1255. preterm infant born to a mother with severe pandemic h1n1 influenza; [aǧır pandemik h1n1 i̇nfluenzalı anneden erken doǧan bebek]
1256. prevalence of anaemia in women with unsupervised medical abortion an observational study
1257. prevalence of and attitudes to abortion among migrant women in sydney
1258. prevalence of morbidity associated with abortion before and after legalisation in south africa
1259. prevalence of self induced abortion by selfadministration of abortive pills among abortionrelated admissions in a tertiary care centre
1260. prevalence of unprofessional social media content among young vascular surgeons (retracted article see vol 72, pg 1514, 2020)
1261. prevention of rh haemolytic disease
1262. primary cause of death in extremely low birth weight infants
1263. primary health care, access to legal abortion and the notion of ideal victim among medical practitioners the case of chile
1264. problems loom ahead
1265. profile of women receiving secondtrimester safe abortion service at paropakar maternity and women’s hospital
1266. profile of women receiving secondtrimester safe abortion service at paropakar maternity and women's hospital
1267. progesterone implantation in habitual abortion
1268. progesteronemediated reversal of mifepristoneinduced pregnancy termination in a rat model an exploratory investigation
1269. progestinbased contraceptive on the same day as medical abortion
1270. prophylactic compared with therapeutic ibuprofen analgesia in firsttrimester medical abortion a randomized controlled trial
1271. prospective study of home use of mifepristone and misoprostol for medical abortion up to 10 weeks of pregnancy in kazakhstan
1272. prospective study of home use of mifepristone and misoprostol for medical abortion up to 10weeks of pregnancy in kazakhstan
1273. prospective study of medical abortion in nepal medical college teaching hospital (nmcth) a one year experience
1274. prostacyclin deficiency in a young woman with recurrent thrombosis
1275. prostaglandin analogs and their uses
1276. prostaglandine2 induction of abortion and fetal demise
1277. prostaglandins and mifepriston in stimulation of medical abortion
1278. protecting safe abortion in humanitarian settings overcoming legal and policy barriers
1279. protective effect of breastfeeding against childhood leukemia in zhejiang province, p r china a retrospective casecontrol study
1280. protective effect of taohong siwu decoction on abnormal uterine bleeding induced by incomplete medical abortion in rats during early pregnancy
1281. providing accessible medical abortion services in a victorian rural community a description and audit of service delivery and contraception follow up
1282. providing mifepristonemisoprostol medical abortion the view from the clinic
1283. provision of abortion by midlevel providers international policy, practice and perspectives
1284. provision of medical abortion by midlevel healthcare providers in kyrgyzstan testing an intervention to expand safe abortion services to underserved rural and periurban areas
1285. provision of medical abortion using telemedicine in brazil
1286. psychiatric morbidity and acceptability following medical and surgical methods of induced abortion
1287. psychiatric morbidity and acceptibility following medical and surgical methods of induced abortion
1288. psychological decision making with regard to motherhood by women with highrisk pregnancy and normal pregnancy; [psychologiczne uwarunkowania decyzji o macierzyństwie kobiet w ciazy wyosokiego ryzyka i w ciazy prawidłowej]
1289. psychological responses following medical abortion (using mifepristone and gemeprost and surgical vacuum aspiration a patientcentered, partially randomised prospective study
1290. psychological responses following medical abortion (using mifepristone and gemeprost) and surgical vacuum aspiration a patientcentered, partially randomised prospective study
1291. psychological sequelae of inducedabortion
1292. psychopathology of recurrent spontaneous abortions; [fausses couches a repetition origine psychogene et/ou psychopathologie induite]
1293. psychosocial aspects of induced abortion
1294. public law no 262, 25 march 1988
1295. putting the icpd plan of action to work turkey women's health tops the agenda
1296. pxr mediates mifepristoneinduced hepatomegaly in mice
1297. quality focuses on clients' needs
1298. radiology rounds intrauterine contraceptive device
1299. randomised controlled trial comparing efficacy of same day administration of mifepristone and misoprostol for termination of pregnancy with the standard 36 to 48hour protocol
1300. randomised controlled trial comparing the efficacy of sameday administration of mifepristone and misoprostol for termination of pregnancy with the standard 36 to 48 hour protocol
1301. randomized study on the effect of adding oxytocin to ethacridine lactate or misoprostol for secondtrimester termination of pregnancy
1302. randomized trial of mifepristone and buccal misoprostol vs misoprostol alone for medical abortion
1303. randomized trial of mifepristone and buccal or vaginal misoprostol for abortion through 56 days of last menstrual period
1304. randomized trial of oral versus sublingual misoprostol 24 h after mifepristone for medical abortion
1305. randomized trial of oral versus vaginal misoprostol 2 days after mifepristone 200 mg for abortion up to 63 days of pregnancy
1306. randomized trial of oral versus vaginal misoprostol at one day after mifepristone for early medical abortion
1307. randomized, doubleblind, controlled trial of mifepristone in capsule versus tablet form followed by misoprostol for early medical abortion
1308. reasons due to which, women resort to illegally induced abortions
1309. recent developments of medical law
1310. recours à l'avortement provoqué à lomé (togo) évolution, facteurs associés et perceptions
1311. recurrent pelvic abscess an unusual presentation of an undetected chronic ectopic pregnancy a case report
1312. reduced fertility among women employed as dental assistants exposed to high levels of nitrous oxide
1313. reducing the buccal dose of misoprostol in mifepristone medical abortion up to 63 days lmp
1314. religion, conscience, and controversial clinical practices
1315. rendering follicle tubes permeable again after postpartum sterilization a report on 25 cases, should postpartum sterilization be performed or not
1316. rendering follicle tubes permeable again after postpartum sterilization a report on 25 cases, should postpartum sterilization be performed or not?; [repermeabilisation tubaire apres sterilisation du postpartum a propos de 25 cas, fautil remettre en cause la sterilisation du postpartum?]
1317. replacement of dilation and curettage/evacuation by manual vacuum aspiration and medical abortion, and the introduction of postabortion contraception in pakistan
1318. replacing the dead the politics of reproduction in the postwar soviet union, 19441955
1319. reply of the authors setting up a medical abortion service for failure
1320. report of survey pregnancy outcomes in medical laboratory technologists
1321. report of two cases of cervical pregnancy treated in a divergent way; [embarazo cervical tratado de forma divergente reporte de dos casos]
1322. representations and uses of emergency contraception in west africa a social anthropological reading of a northern medicinal product
1323. reproductive choice for women and men living with hiv contraception, abortion and fertility
1324. reproductive failure due to spontaneous abortion and recurrent miscarriage
1325. reproductive health and blurred professional boundaries
1326. reproductive health crisis during waves one and two of the covid19 pandemic in india incidence and deaths from severe maternal complications in more than 202,000 hospital births
1327. reproductive health information and abortion services standards developed by the european court of human rights
1328. reproductive justice discourse visàvis abortion law in india a critical review
1329. reproductive patterns as environmental markers in rural morocco
1330. reproductive performance of patients treated with clomiphene citrate
1331. researches in aetiology of nonspecific urethritis
1332. restrictions in access to abortion the italian situation
1333. restrictive lung disease in pregnancy
1334. restrictive lungdisease in pregnancy
1335. restrictive state abortion bansa reproductive injustice
1336. results and lessons learned from a small medical abortion clinical study in turkey
1337. results of first trimester medical abortion care
1338. resurgence of congenital rubella syndrome in the 1990s report on missed opportunities and failed prevention policies among women of childbearing age
1339. retraction notice (journal of vascular surgery (2020) 72(2) (667671), (s074152141932587x), (101016/jjvs201910069))
1340. retrospective analysis of prognostic value of the neutrophiltolymphocyte ratio in early miscarriages a 8year survey
1341. retrospective assessment of the influence of gestational age on the first trimester medical abortion efficiency
1342. retrospective observational assessment of the hazards of otc availability of mtp pills
1343. return of fertility after discontinuation of contraception according type of contraception, duration of use, age and body mass index; [kembalinya kesuburan setelah penghentian alat kontrasepsi berdasarkan jenis kontrasepsi, lama pemakaian, usia dan indeks massa tubuh]
1344. review of the feasibility of mifepristone & misoprostol for medical abortion in developing countries
1345. rheumatoid arthritis and pregnancy a case report and literature review; [artritis reumatoide y embarazo reporte de un caso y revisión de la bibliografía]
1346. right tool, wrong job manual vacuum aspiration, postabortion care and transnational population politics in senegal
1347. risk factors and the choice of longacting reversible contraception following medical abortion effect on subsequent induced abortion and unwanted pregnancy
1348. risk factors for intrauterine device embedment in postmenopausal women an analysis of 731 participants undergoing hysteroscopy
1349. risk factors for unsuccessful medical abortion with mifepristone and misoprostol
1350. risk management in pregnancy termination
1351. risk of complications during pregnancy in women with congenital aortic stenosis
1352. risk of surgical evacuation and risk of major surgery following secondtrimester medical abortion in denmark a nationwide cohort study
1353. risks and costs of illegally induced abortion in bangladesh
1354. risks and costs of illegally inducedabortion in bangladesh
1355. rocking the cradle the uncertain policy of withholding treatment from anomalous newborns (infanticide, mercykilling, defective infants, selective nontreatment, euthanasia)
1356. role of isosorbide mononitrate as an agent for cervical ripening in secondtrimester abortions
1357. role of routine ultrasonography in monitoring the outcome of medical abortion in a clinical setting
1358. role of ultrasonography in evaluation of bleeding per vagina in first trimester of pregnancy
1359. role of ultrasonography in the evaluation of causes of bleeding per vaginum in the first trimester of pregnancy
1360. role of ultrasound in first trimester bleeding
1361. routine followup versus selfassessment of complete abortion following medical abortion, effect on its success and acceptability a randomized controlled trial
1362. ru 486 (mifepristone) "the abortive pill" our experience
1363. ru 486 (mifepristone) from clinicaltrials to the perspectives for clinical use
1364. ru 486 (mifepristone) from clinical trials to the perspectives for clinical use; [ru 486 (mifepristone) des essais cliniques aux perspectives d'utilisation]
1365. ru486 misconceptions, myths and morals a critique of the criticisms
1366. rubella vaccine in postpubertal women experience in western washington state
1367. rudimentary uterine horn pregnancy in 15 cases case series with a twoyear followup
1368. safe abortions are they a reality yet? a case series
1369. safe medical abortion in the second trimester of pregnancy
1370. safety and efficacy of medical abortion in a pediatric population
1371. safety and efficacy of outpatient mifepristonemisoprostol medical abortion through 76 days of gestational ageportuguese experience in a tertiary hospital
1372. safety and feasibility of office hysteroscopy in removing retained products of conception
1373. safety of induced abortions at less than 12 weeks of pregnancy in japan
1374. scar ectopic pregnancy type ii / endogenic with impending scar rupture
1375. scenario of self medication for medical abortion in a tertiary care centre
1376. second and thirdtrimester termination of pregnancy in women with uterine scar a retrospective analysis of 111 gemeprostinduced terminations of pregnancy after previous cesarean delivery
1377. second trimester abortions in india
1378. second trimester medical abortion in a primigravida with lupus nephritis and rapidly progressive renal failure challenges and outcome
1379. second trimester medical abortion with mifepristone and gemeprost a review of 956 cases
1380. second trimester medical abortion with mifepristonemisoprostol and misoprostol alone a review of methods and management
1381. secondary infertility and the aging male, overview
1382. secondtrimester medication abortion outside the clinic setting an analysis of electronic client records from a safe abortion hotline in indonesia
1383. secondtrimester surgical and medical abortion practice in canada in 2012 a national survey
1384. secular trends in uncertainsex births and proportion of male births in norway, 19671998
1385. self administration versus supervised use of medical abortion pills impact on women’s health
1386. self reported outcomes and adverse events after medical abortion through online telemedicine population based study in the republic of ireland and northern ireland
1387. selfadministered multilevel pregnancy tests in simplified followup of medical abortion in tunisia
1388. selfadministered versus provideradministered medical abortion
1389. selfadministration of abortion pills and its maternal outcome in tertiary care center
1390. selfassessment of medical abortion outcome using symptoms and home pregnancy testing
1391. selfassessment of the outcome of early medical abortion versus clinic followup in india a randomised, controlled, noninferiority trial
1392. septic abortion
1393. septic abortion personal experience and management
1394. septic abortion a 5year experience at siriraj hospital
1395. septic pelvic thrombophlebitis of unknown origin an ever threatening entity
1396. sequelae of premature sexual life
1397. sequelae of reentry of tachycardia during intrauterine development and in the newborn period; [dôsledky reentry tachykardie prítomnej počas vnútromaternicového vývoja a v novorodeneckom období]
1398. sequential use of foley catheter with misoprostol for second trimester pregnancy termination in women with and without caesarean scars a prospective cohort study
1399. seroprevalence of babesia caballi and theileria equi in horses in central germany
1400. seroprevalence of babesia caballi and theileria equi in horses in central germany; [vorkommen von antikörpern gegen babesia caballi und theileria equi bei pferden in mitteldeutschland]
1401. serum biomarkers may help predict successful misoprostol management of early pregnancy failure
1402. serum βhcg concentration is a predictive factor for successful early medical abortion with vaginal misoprostol within 24 hours
1403. service delivery correlates of choosing shortacting contraceptives at the time of uterine evacuation in bangladesh
1404. setting up a medical abortion service for failure
1405. setting up a medical abortion service for failure reply
1406. severe acute maternal morbidity a pilot study of a definition for a nearmiss
1407. sex education prerequisite for family planning; [education sexuelle prealable necessaire a la planification familiale]
1408. sexual activity in girls under 16 years of age
1409. sexual knowledge and attitudes of adolescents relationship to contraceptive use
1410. sexual knowledge and attitudes of adolescents relationship to contraceptive use
1411. sexualactivity in girls under 16 years of age
1412. sexuality, words and familyplanning
1413. short and long term mortality rates associated with first pregnancy outcome population register based study for denmark 19802004
1414. should organs from patients in permanent vegetative state be used for transplantation?
1415. side effects of induced abortion results from a populationbased survey
1416. sífilis congénita auditorías en tiempo real
1417. significance of clinical and anamnestic risk factors for the prediction of negative outcomes in assisted reproductive technology programs
1418. silent uterine rupture with the use of misoprostol for second trimester termination of pregnancy a case report
1419. simplified medical abortion screening a demonstration project
1420. simultaneous administration of mifepristone and misoprostol for early termination of pregnancy a randomized controlled trial
1421. simultaneous administration of mifepristone and misoprostol increases access to early medical abortion where homeuse of misoprostol is not permitted
1422. simultaneous compared to interval administration of mifepristone and misoprostol for medical abortion up to 10 +0 weeks' gestation a systematic review with metaanalyses
1423. simultaneous compared to interval administration of mifepristone and misoprostol for medical abortion up to 10(+0) weeks' gestation a systematic review with metaanalyses
1424. simultaneous compared with interval medical abortion regimens where home use is restricted
1425. simultaneous compared with interval medical abortion regimens where home use is restricted correction
1426. síndrome de hellp en el hospital nacional guillermo almenara irigoyen presentación clínica y complicaciones de una emergencia obstétrica
1427. situation analysis of patients attending tu teaching hospital after medical abortion with problems and complications
1428. social and medical implications of teenage motherhood
1429. social questions, medical answers contesting british abortion law
1430. sociocultural and obstetric risk factors associated with incomplete abortion in peruvian women; [factores de riesgo socioculturales y obstétricos asociados al aborto incompleto en mujeres peruanas]
1431. sociopsychological aspects of voluntary abortion at the obstetrical and gynecological clinic of catania; [aspetti sociopsicologici dell'aborto volontario nella clinica ostetrica e ginecologica di catania]
1432. soluble mhc class i chainrelated molecule serum levels are predictive markers of implantation failure and successful term pregnancies following ivf
1433. some errors after the medical abortion recovery period and contraception choice
1434. some psychiatric aspects of abortion
1435. sonographic patterns of the endometrium in assessment of medical abortion outcomes
1436. sonographic quantification of endometrial changes after abortion with computerassisted image analysis
1437. sonohysterography a simple tool for the diagnosis of pregnancy in the rudimentary horn a case report
1438. spanish abortion reform faces opposition
1439. sperm proteins as targets for contraception and fertility biomarkers
1440. spontaneous foetal losses in women using different contraceptives around the time of conception
1441. standardized second trimester medical abortion training and service provision in ethiopia, october 2010september 2014
1442. status epilepticus in pregnancy a literature review and a protocol proposal
1443. status of male contraception
1444. std prevention and unplanned pregnancy a uk perspective; [prevention des mst et grossesses indesirees en grandebretagne]
1445. strategies for nursing uterine rupture in primiparas during pregnancy
1446. structural barriers or patient preference? a mixed methods appraisal of medical abortion use in england and wales
1447. structural equation modelling analysis determining causal role among methyltransferases, methylation, and apoptosis during human pregnancy and abortion
1448. structure of reproductive losses of adolescent girls and women of reproductive age in the tyumen
1449. study of pregnancy outcome over a period of five years in a postgraduate institute of west bengal
1450. study of sideeffects of cut as intrauterine contraceptive device in post medical termination of pregnancy and interval cases
1451. study of 'spontaneous' abortion in thailand
1452. study of spontaneous abortion in thailand
1453. study of the relation between preterm delivery and periodontitis the role of prophylactic dental therapy in the prevention of preterm delivery
1454. study on clinical presentation and outcome of septic abortion and its relationship with person inducing abortion
1455. study results translate to better services
1456. sublingual v/s vaginal misoprostol for second trimester termination of pregnancy a comparative study
1457. subsequent placenta accreta after previous mifepristoneinduced abortion a case report
1458. success rate of methotrexate treatment for recurrent vs primary ectopic pregnancy a casecontrol study
1459. success rate of secondtrimester termination of pregnancy using misoprostol
1460. successful medical abortion of a pregnancy within a noncommunicating rudimentary uterine horn
1461. successful surgical treatment of invasive pancreatic ductal adenocarcinoma during pregnancy
1462. successful termdelivery following medically managed 9 weeks live caesarean scar pregnancy
1463. successful treatment of placenta percreta through a combinatorial treatment involving a bakri balloon and methotrexate a case report
1464. suggested fda restrictions might curtail access to mifepristone
1465. suggestions on revision regarding abortion laws in korea an empirical study using qualitative research method
1466. surgical intervention after medical abortion
1467. surgical management of early pregnancy failure history, politics, and safe, costeffective care
1468. surgical management of incomplete abortion by manual vacuum aspiration (mva)
1469. surgical or medical abortion of pregnancies between 13 +0 and 23 +6 weeks' gestation? a systematic review and new nice national guidelines
1470. surgical or medical abortion of pregnancies between 13(+0) and 23(+6) weeks' gestation? a systematic review and new nice national guidelines
1471. survey of the attitude to, knowledge and practice of contraception and medical abortion in women attending a family planning clinic
1472. survey of the attitude to, the knowledge and the practice of contraception and medical abortion in women who attended a family planning clinic
1473. survey women educational needs about routine ocps
1474. syphilitic endometritis causing first trimester abortion a potential infectious cause of fetal morbidity in early gestation
1475. systematic review of the effectiveness, safety, and acceptability of mifepristone and misoprostol for medical abortion in low and middleincome countries
1476. systematic review the maternal mortality myth in the context of legalized abortion
1477. systemic inflammatory response syndrome, organ failure, and outcome in critically ill obstetric patients treated in an icu
1478. tamoxifen compared to methotrexate when used with misoprostol for abortion
1479. tanzanian lessons in using nonphysician clinicians to scale up comprehensive emergency obstetric care in remote and rural areas
1480. targeted client communication via mobile devices for improving sexual and reproductive health
1481. teen pregnancy effect on family wellbeing
1482. teenage pregnancy and motherhood a review of the literature
1483. telemedicine for medical abortion a systematic review
1484. telemedicine medical abortion at home under 12 weeks' gestation a prospective observational cohort study during the covid19 pandemic
1485. telephone followup after early medical abortion using australia's first low sensitivity urine pregnancy test
1486. ten years on a review of medical terminations of pregnancy performed in a sexual health clinic
1487. tenminute abortions
1488. termination of 2nd trimester pregnancy with sulprostone and mifepristone a randomized doubleblind placebocontrolled trial
1489. termination of a pregnancy located in one horn of a bicornuate uterus challenges and solution
1490. termination of early pregnancy by the 3βhydroxysteroid dehydrogenase inhibitor epostane
1491. termination of early pregnancy by two regimens of mifepristone with misoprostol and mifepristone with pg05 a multicentre randomized clinical trial in china
1492. termination of early pregnancy by two regimens of mifepristone with misoprostol and mifepristone with pg05a multicentre randomized clinical trial in china
1493. termination of early pregnancy by two regimens of mifepristone with misoprostol a multicentre clinical trial
1494. termination of early pregnancy using flexible, lowdose mifepristonemisoprostol regimens
1495. termination of early pregnancy using ru 486 alone or in combination with prostaglandin the ru 486 collaboration group
1496. termination of earlypregnancy by 2 regimens of mifepristone with misoprostol and mifepristone with pg05 a multicenter randomized clinicaltrial in china
1497. termination of pregnancy at very early gestation without visible yolk sac on ultrasound
1498. termination of pregnancy in papua new guinea the traditional and contemporary position
1499. termination of pregnancy in papuanewguinea the traditional and contemporary position
1500. termination of pregnancy services in irish general practice from january 2019 to june 2019
1501. the abortion battle the canadian scene
1502. the abortion paradox
1503. the abortion pill a solution for unsafe abortions in developing countries?
1504. the applied of computer for analysis on contraceptive efficacy of iud of rural women in guangdong province
1505. the association between history of retained placenta and success rate of misoprostol treatment for early pregnancy failure
1506. the case studies
1507. the characteristics of and the contraceptive practice among women seeking therapeutic termination of pregnancy in the scottish highlands
1508. the clinical aspects of acute renal failure, with particular reference to the postpartum period; [aspetti clinici dell'insufficienza renale acuta con particolare riguardo all'ira postpartum]
1509. the clinical management of abortion
1510. the clinical management of repeated earlypregnancy wastage
1511. the clinical use of karyotyping spontaneous abortions
1512. the contraceptive choice at the time of a surgical and pharmacological abortion a possible and effective option thanks to a dedicate counselling
1513. the contraceptive practice of abortion patients
1514. the contribution of environmental teratogens to embryonic and fetal loss
1515. the cost of child rearing for wrongful conception
1516. the counseling component of medical abortion
1517. the culture of illegal abortion in south africa
1518. the determinants and health consequences of unsafe abortion in rio de janeiro, brazil
1519. the development of medical law in the field of gynecology and the relationship with criminal law the applicability of the legal framework of the republic of kosovo; [rozwój prawa medycznego w dziedzinie ginekologii a jego związek z prawem karnym obowiązywanie ram prawnych republiki kosowa]
1520. the diagnosis of early pregnancy failure by sonar
1521. the difficulties of contraception conflicts and paradoxes
1522. the effect of cardiac surgery on a success rate of postoperative pregnancy the results of 108 cases of pregnancy after cardiac surgery for congenital and acquired heart diseases
1523. the effect of dose of mifepristone and gestation on the efficacy of medical abortion with mifepristone and misoprostol
1524. the effect of hysteroscopic metroplasty on fertility in tshaped and yshaped dysmorphic uterus
1525. the effect of misoprostol with and without letrozole in inducing successful medical abortion
1526. the effect of nonsteroidal antiinflammatory drugs on medical abortion with mifepristone and misoprostol at 1322 weeks gestation
1527. the effect of smoking on reproductive failures in couples examined in the genetic outpatient clinic at dr a jurasz university hospital in bydgoszcz
1528. the effect of smoking on reproductive failures in couples examined in the genetic outpatient clinic at dr a jurasz university hospital in bydgoszcz; [rozpowszechnienie palenia tytoniu wśród par z niepowodzeniami cia̧żowymi z poradnigenetycznej szpitala uniwersyteckiego im dr a jurasza w bydgoszczy]
1529. the effectiveness of home medical abortions provided through telemedicine
1530. the effects of electromagnetic field on pregnancy; [elektromanyetik alanin gebelik üzerine etkileri]
1531. the effects of religious beliefs on the health care practices of the amish
1532. the effects of the antihormones ru486 and tamoxifen on fetoplacental development and placental bed vascularisation in the rat a model for intrauterine fetal growth retardation
1533. the efficacy and acceptability of mifepristone medical abortion with home administration misoprostol provided by private providers linked with the hospital a prospective study of 433 patients
1534. the efficacy of medical abortion a metaanalysis
1535. the efficacy of shenghua decoction supplementation after early medical abortion a metaanalysis of randomized controlled trials
1536. the ethical legacy of hippocrates
1537. the experience of carrying out the technical responsibility system in the birth control of peng county, sichuan province
1538. the experiences of women accessing legal abortion in mexico city
1539. the extended gestational age medical abortion study the effectiveness of medical abortion with mifepristone and misoprostol at 5763 days versus 6470 days gestation
1540. the feasibility of a modified method of laparoscopic transabdominal cervicoisthmic cerclage during pregnancy
1541. the feasibility of early 2nd trimester pregnancy (12 to 14 weeks) termination with single intraamniotic injection of 15methyl pgf2alpha
1542. the feasibility of early second trimester pregnancy (12 to 14 weeks) termination with single intra‐amniotic injection of 15‐methyl pgf2α
1543. the feasibility of offering medical abortions by tele medicine 20 months of experience
1544. the fight for reproductive rights in central and eastern europe poland catholic backlash
1545. the frequency of chromosomal abnormalities in patients with reproductive failure
1546. the frequency of chromosomal, abnormalities in patients with reproductive failure
1547. the gag rule revisited physicians as abortion gatekeepers
1548. the gynecologist and the problem of therapeutic abortion; [le medecin gynecologue face au problème de l'avortement thérapeutique]
1549. the hemostatic system with medical abortion
1550. the hospital's role in contraception
1551. the immunobiology of abortion
1552. the impact of provider restrictions on abortionrelated outcomes a synthesis of legal and health evidence
1553. the impact of the covid19 pandemic on the course of miscarriages
1554. the impact of the prenatal ultrasonography on birth of babies with korean pediatric surgical index diseases
1555. the induction of abortion in the 2nd trimester by combined administration of minprostin and sulproston, compared with the use of sulproston alone
1556. the induction of abortion in the second trimester by combined administration of minprostin and sulproston, compared with the use of sulproston alone; [kombinierter einsatz von minprostin und sulproston bei der aborteinleitung im ii trimenon und bei der geburtseinleitung beim toten kind im vergleich zum alleinigen sulprostoneinsatz]
1557. the induction of abortion with prostaglandins
1558. the influence of abortion legislation on maternal mortality
1559. the influence of contraceptive practice upon maternal and child health
1560. the introduction of first trimester medical abortion in armenia
1561. the invisible pregnant athlete and the promise of title ix
1562. the language of firsttrimester nonviable pregnancy patientreported preferences and clarity
1563. the legal implications of prenatal diagnosis in malaysia
1564. the legal status of the fetus as a patient in europe
1565. the medical management of incomplete abortion
1566. the midwife's role in abortion care
1567. the modern abortion jurisprudence under article 8 of the european convention on human rights
1568. the montana fetal genetic pathology program and a review of prenatal death in humans
1569. the morphology, typical combinations of polymorphic genes of hemostasis, and specific features of the pathogenesis of retrochorial hematoma in missed abortion
1570. the outcome of home medical abortions provided through telemedicine
1571. the outcome of in vitro fertilization in unexplained habitual aborters concurrent with secondary infertility
1572. the outcome of septic abortion a tertiary care hospital experience
1573. the pattern of maternal mortality at maternity hospital kuala lumpur
1574. the perimenopause and contraception
1575. the political challenges and educational opportunities around very early abortion
1576. the portrayal and perceptions of cesarean section in mexican media facebook pages a mixedmethods study
1577. the position of general practitioner midwifery
1578. the preemptive effect of ibuprofen versus placebo on pain relief and success rates of medical abortion a doubleblind randomised controlled study
1579. the pregnant plastic surgical resident results of a survey of women plastic surgeons and plasticsurgery residency directors
1580. the problem of teenage pregnancy
1581. the profile of women who seek emergency contraception from the family planning service
1582. the psychosocial factors of the abortion experience a critical review
1583. the relationship between insecurity and the quality of hospital care provided to women with abortionrelated complications in the democratic republic of congo a crosssectional analysis
1584. the risk of birth defects jacobs v theimer and parents' right to know
1585. the role of auxiliary nursemidwives and community health volunteers in expanding access to medical abortion in rural nepal
1586. the role of medical abortion in the implementation of the law on voluntary termination of pregnancy in uruguay
1587. the role of parity in medical abortion up to 49 days of amenorrhoea
1588. the safety and efficacy of chorionic villus sampling for early prenatal diagnosis of cytogenetic abnormalities
1589. the spectrum of relapsing fever in the rocky mountains
1590. the telabortion project delivering the abortion pill to your doorstep by telemedicine and mail
1591. the theory and practice of self ownership
1592. the trophoblastic blood flow during the course of medical abortion
1593. the unwanted child
1594. the use of manual vacuum aspiration in the treatment of incomplete abortions a descriptive study from three public hospitals in malawi
1595. the use of prostaglandins and antiprogestins for pregnancy termination
1596. the use of prostaglandins in obstetrics
1597. the usefulness of the chorionic villi technic in prenatal diagnosis; [korisnost korion frondozum tehnike u prenatalnoj dijagnozi]
1598. the value of posterior cervical angle as a predictor of vaginal delivery a preliminary study
1599. thelper 1type immunity to trophoblast in women with recurrent spontaneous abortion
1600. thelper 1type immunity to trophoblast in women with recurrent spontaneousabortion
1601. therapeutic abortion by intraamniotic injection of prostaglandins
1602. therapeutic termination of second trimester pregnancies with low dose misoprostol
1603. third time unlucky a study of women who have three or more legal abortions
1604. thrombophilia, anticoagulant therapy, and pregnancy outcome in women with poor obstetric history
1605. timing and indication for curettage after medical abortion in early pregnant women with prior uterine incision
1606. tissue distribution of cocaine in a pregnant woman
1607. toward a standard measure of abortion service qualitya stakeholder first approach
1608. toxic shock associated with clostridium sordellii and clostridium perfringens after medical and spontaneous abortion
1609. transplacental passage of fetal red cells in abortion; increased incidence after curettage and effect of oxytocic drugs
1610. trauma in pregnancy predicting pregnancy outcome
1611. treatment of epilepsy in women of childbearing age
1612. trend of serum betahuman chorionic gonadotropin levels after medical abortion in the early first trimester of pregnancy
1613. trends in elective medical and surgical abortions in the united states during the covid19 pandemic
1614. triangular assessment of the etiology of induced abortion in iran a qtualitative study
1615. triangular assessment of the etiology of induced abortion in iran a qualitative study
1616. tucson woman's clinic v eden
1617. two cases of autoimmune hemolytic anemia during pregnancy
1618. two mifepristone doses and two intervals of misoprostol administration for termination of early pregnancy a randomised factorial controlled equivalence trial
1619. two prophylactic pain management regimens for medical abortion ≤63 days' gestation with mifepristone and misoprostol a multicenter, randomized, placebocontrolled trial
1620. twopill regimens of misoprostol after mifepristone medical abortion through 63 days' lmp a randomized controlled trial of buccal and sublingual misoprostol
1621. ulipristal acetate and pregnancy outcomean observational study
1622. ultrasonographic characteristics in patients clinically diagnosed with threatened abortion
1623. ultrasonographic evaluation of endometrial changes using computer assisted image analysis
1624. ultrasonographic features of the endometrium following successful medical termination of early pregnancy
1625. ultrasound evaluation of the endometrium after medical termination of pregnancy
1626. ultrasound in republic of yemen; [echographie en republique du yemen]
1627. umbilical cord lesions in early intrauterine fetal demise
1628. uncommon toxicity of lowdose methotrexate case report
1629. uncomplicated abortion with mifepristone and misoprostol in a hemophilia a carrier
1630. understanding of changes in abortion rate following liberalization of abortion law among brazilian residents in obstetrics and gynecology
1631. unsafe abortion an avoidable tragedy
1632. unsuccessful prior attempts to terminate pregnancy among women seeking first trimester abortion at registered facilities in bihar and jharkhand, india
1633. unusual intrauterine objects potential pitfalls in ultrasonographic identification
1634. unusual intrauterine objects potential pitfalls in ultrasonographic identification
1635. uptake and outcome of artificial reproductive techniques following allogeneic stem cell tranplantation a single centre experience
1636. usa victory over gag rule for family planning groups
1637. use of an athome multilevel pregnancy test and an automated telephone system to simplify medical abortion followup
1638. use of mifepristone and sublingual misoprostol for early medical abortion
1639. use of misoprostol during pregnancy and mobius syndrome in infants
1640. use of misoprostol in gynecology and obstetrics
1641. use of misoprostol in gynecology and obstetrics; [utilisation du misoprostol en gynécologieobstétrique]
1642. use of platelet rich plasma in human infertility
1643. use of prostaglandin for induction of second trimester abortions in high risk pregnancy
1644. use of quinine for selfinduced abortion
1645. usercentred design and development of a patient decision aid for choice of first trimester abortion method
1646. using oral and intravaginal misoprostol for medical abortion
1647. using telemedicine to improve early medical abortion at home (utah) a randomised controlled trial to compare telemedicine with inperson consultation for early medical abortion
1648. utah using telemedicine to improve early medical abortion at home a protocol for a randomised controlled trial comparing facetoface with telephone consultations for women seeking early medical abortion
1649. uterine artery embolization in the treatment and prevention of postpartum hemorrhage
1650. uterine aspiration for evacuation of the pregnant uterus
1651. uterine preservation surgery for placental polyp
1652. uterine rupture during pregnancy and delivery risk factors, symptoms and maternal and neonatal outcomes restrospective cohort
1653. uterine rupture during pregnancy and delivery risk factors, symptoms and maternal and neonatal outcomes restrospective cohort; [ruptura d&#283;lohy v t&#283;hotenství a p&#345;i porodu rizikové faktory, p&#345;íznaky a perinatální výsledky retrospektivní analýza]
1654. utility of manual vaccum aspiration (mva) in a tunisian tertiary referral center
1655. vaccination against influenza in pregnant women safety and effectiveness
1656. vaccination against influenza in pregnant women safety and effectiveness; [szczepienia przeciwko grypie u kobiet ciężarnych bezpieczeństwo i efektywność]
1657. vaginal bleeding and pelvic pain after medical abortion a case of incomplete abortion diagnosed on pocus transvaginal ultrasound
1658. vaginal misoprostol administered 1, 2, or 3 days after mifepristone for early medical abortion a randomized trial
1659. vaginal misoprostol alone for medical abortion up to 9 weeks of gestation efficacy and acceptability
1660. vaginal misoprostol for salvage therapy after failed medical abortion
1661. vaginal pge2 and intraamniotic pgf2 alpha for the termination of second trimester pregnancy
1662. validation of a spanishlanguage scale for evaluating perceived quality of care of medical abortions before 9 weeks gestation
1663. validation of icd9 codes for stable miscarriage in the emergency department
1664. value of hysteroscopy and laparoscopy in differential diagnosis of gestational trophoblastic neoplasia
1665. vasectomy on request still a moot point for discussion
1666. voluntary interruption of pregnancy not an insignificant act; [l'ivg un acte non anodin]
1667. what is needed to ensure the health and survival of mother and baby?
1668. when is "emergency" contraception the right name for postcoital treatment?
1669. when is emergency contraception the right name for postcoital treatment?
1670. which evacuation method for missed abortion is preferred by women with recurrent pregnancy loss?
1671. who multinational study of three misoprostol regimens after mifepristone for early medical abortion i efficacy
1672. who will do the abortions?
1673. why is access to medical abortion limited in the russian federation?
1674. willing and unable doctors' constraints in abortion care
1675. willing but not able a survey of new zealand health practitioners' interests in providing second trimester abortion care and the obstacles they face
1676. wilson v kuenzi, 17 may 1988
1677. wish of pregnancy refusal of pregnancy ambivalence in family planning; [desir de grossesse refus de grossesse ou les embuches du desir dans le planning familial]
1678. womancare of southfield, pc v granholm
1679. women's decision making regarding choice of second trimester termination method for pregnancy complications
1680. women's evaluation of three early abortion methods
1681. women's medical center of northwestern houston v bell
1682. women's medical center of providence v roberts
1683. women's perspectives on ultrasound viewing in the abortion care context
1684. womens reproductive health
1685. women's selfreported experiences using misoprostol obtained from drug sellers a prospective cohort study in lagos state, nigeria
1686. women's voices and medical abortions a review of the literature
1687. y to x translocation in a woman with reproductive failure a new rearrangement
1688. yoruba traditional healers' knowledge of contraception, abortion and infertility
1689. your conscience, your right a history of efforts to violate prolife medical conscience, and the laws that stand in the way
1690. youth often risk unsafe abortions

**3. Records excluded (n=1466): Case reports (n=74), Reviews (n=197), Non-English language records (n=212), Irrelevant records (n=983)**

**3.1 Records excluded: Case reports (n=74)**

1. a case of fetal methotrexate syndromea rare cause of multiple congenital anomalies
2. a case of morbius syndrome in a newborn baby after medical elective abortion failure
3. a classic case of toxic shock syndrome due to a not so classic organism, clostridium sordellii
4. a comparison of pregnancy outcomes after secondtrimester amniocentesis between cases with penetration of the placenta and nonpenetration
5. a fatal case of clostridium sordellii septic shock syndrome associated with medical abortion
6. a foreign body in the cervix after spontaneous abortion a rare case of a traumatic fetal decapitation
7. a mutation in the mechanisms of socialcontrol the case of abortion
8. a novel method for office aspiration curettage in cases of retained products of conception a randomized controlled trial
9. a profile of abortion cases in a tertiary care hospital
10. a study of 500 cases of acute renal failure (19781991)
11. a study of 500 cases of acuterenalfailure (19781891)
12. a maternal death from selfinduced medical abortion a call for action
13. abortion needs of women in india a case study of rural maharashtra
14. about the bioethics of abortion at request in romania a case presentation
15. amyloidosis associated with igg (λ) mproteinemia a case of autopsy
16. an account of 2180 cases of percoelioscopic tuballigation using yoon mechanical method
17. an effective regimen for early medical abortion a report of 2000 consecutive cases
18. case study on the wrongful birth action
19. causes of perinatal mortality report of a study of 4,429 cases
20. cervical vasovagal shock a rare complication of incomplete abortion case report
21. challenging cases in care for termination of pregnancy
22. cleft lip and palate series of unusual clinical cases
23. clinical analyses of 66 cases of midtrimester pregnancy termination in women with prior cesarean
24. clinical observation on termination of early pregnancy of 213 cases after caesarian section with repeated use of mifepristone and misoprostol
25. clinical study of four cases with malignant gestation trophoblastic tumor after mifepristone abortion
26. combined vesicouterine rupture during secondtrimester medical abortion for fetal abnormality after prior cesarean delivery a case report
27. complications of unsafe abortion a case study and the need for abortion law reform in nigeria
28. coping with fertility in israel a case study of culture clash
29. coronary artery vasospasm after misoprostol treatment for incomplete abortion a case report
30. decision making on unsafe abortions in sri lanka a casecontrol study
31. decisionmaking and choice about childbearing a case study of deferred motherhood
32. delayed hemolysis, elevated liver enzymes, low platelet count syndrome in succession of switches of preventive anticoagulant treatment in a 41yearold patient with a history of recurrent assisted implantation failures a case report
33. efficacy of mifepristonemisoprostol combination in cases of missed abortion in first trimester
34. endometrial polyp filled with gestational tissues remained undiscovered in an infertile woman for years a case report
35. experts, non experts, and policy discourse a case study of the royal commission on new reproductive technologies
36. failed early medical abortion beware of the uterine scar! case report
37. failed medical termination of twin pregnancy with mifepristone a case report
38. false positive results of tests for syphilis and outcome of pregnancy a retrospective casecontrol study
39. homocysteine serum concentration and uterine artery color doppler examination in cases of recurrent miscarriages with unexplained etiology
40. hyperosmolar urea for elective midtrimester abortion experience in 1,913 cases
41. hypertonic salineinduced abortion complicated by consumptive coagulopathy a case report
42. impact of the covid19 pandemic on the prevalence of mtp cases and their clinicodemographic profile in india a retrospective multicentric study
43. inducedabortion in kenya casehistories
44. introduction of early medical abortion in new zealand an audit of the first 67 cases
45. laparoscopic management of caesarean scar ectopic pregnancy a report of 2 cases
46. laparoscopic management of caesarean scar ectopic pregnancya report of 2 cases
47. launch, drift, and withdrawal of public policy a case study of the korean medical school system
48. lupus erythematodes associated with pregnancy (a death case after delivery)
49. management of cases of abortion
50. management of pheochromocytoma during pregnancy about three cases
51. medical abortion in family practice a case series
52. misoprostolinduced termination of secondtrimester pregnancy in women with a history of cesarean section a retrospective analysis of 56 cases
53. outcome of managing second trimester post abortion cases using a standardized combination of regimen
54. parity is a major determinant of success rate in medical abortion a retrospective analysis of 3161 consecutive cases of early medical abortion treated with reduced doses of mifepristone and vaginal gemeprost
55. parvovirus b19 infection frequency in placenta of fetal loss cases in children medical center, tehran, iran
56. pregnancy outcome in women with a history of congenital heart disease a case series study
57. pregnancy termination in a female with paraplegia a case report
58. primary health care, access to legal abortion and the notion of ideal victim among medical practitioners the case of chile
59. protective effect of breastfeeding against childhood leukemia in zhejiang province, p r china a retrospective casecontrol study
60. recurrent pelvic abscess an unusual presentation of an undetected chronic ectopic pregnancy a case report
61. rendering follicle tubes permeable again after postpartum sterilization a report on 25 cases, should postpartum sterilization be performed or not
62. rudimentary uterine horn pregnancy in 15 cases case series with a twoyear followup
63. safe abortions are they a reality yet? a case series
64. silent uterine rupture with the use of misoprostol for second trimester termination of pregnancy a case report
65. sonohysterography a simple tool for the diagnosis of pregnancy in the rudimentary horn a case report
66. study of sideeffects of cut as intrauterine contraceptive device in post medical termination of pregnancy and interval cases
67. subsequent placenta accreta after previous mifepristoneinduced abortion a case report
68. success rate of methotrexate treatment for recurrent vs primary ectopic pregnancy a casecontrol study
69. successful treatment of placenta percreta through a combinatorial treatment involving a bakri balloon and methotrexate a case report
70. the case studies
71. the effect of cardiac surgery on a success rate of postoperative pregnancy the results of 108 cases of pregnancy after cardiac surgery for congenital and acquired heart diseases
72. two cases of autoimmune hemolytic anemia during pregnancy
73. uncommon toxicity of lowdose methotrexate case report
74. vaginal bleeding and pelvic pain after medical abortion a case of incomplete abortion diagnosed on pocus transvaginal ultrasound

**3.2 Records excluded: Reviews (n=197)**

1. "criminality"
2. "i will never wish this pain to even my worst enemy" lived experiences of pain associated with manual vacuum aspiration during postabortion care in kenya
3. "reproductive health care," the "demographic imperative," and the real health needs of women in the developing world (part one)
4. "right tool," wrong "job" manual vacuum aspiration, postabortion care and transnational population politics in senegal
5. "ru486 misconceptions, myths and morals" a critique of the criticisms
6. (un)knowing women sexuality, pregnancy, and knowledge production in modernist literature
7. 2017 naf am abstracts
8. a comparison of medical and surgical management of first trimester incomplete miscarriages a randomised control trial in pmgh
9. a noninferiority study of outpatient mifepristonemisoprostol medical abortion at 6470 days and 7177 days of gestation
10. a prospective trial using mifepristone and vaginal misoprostol in termination of pregnancies up to 63 days of gestation
11. a repeat dose of misoprostol 800 mcg following mifepristone for outpatient medical abortion at 6470 and 7177 days of gestation a retrospective chart review
12. a repeat dose of misoprostol 800 mcg following mifepristone for outpatient medical abortion at 6470 and 7177 days of gestation a retrospective chart review
13. a study of the effects of folklore about the body on iud use by blackamerican adolescents
14. abortion epidemiology, safety, and technique
15. abortion in four asian countries patient characteristics, morbidity, and contraceptive acceptance
16. abortion medical management between 14−16 weeks’ amenorrhea after french legislation deadline extension
17. abortion policy and women's health in developing countries
18. abortion services during the covid19 pandemic a systematic review
19. abortion trends in singapore a 25year review
20. abortions in a hospital setting hidden realities in dar es salaam, tanzania
21. abstract #1002287 a case of diabetic ketoacidosis in medical management of miscarriage
22. acute renal failure during the pregnancy a review on pathophysiology, risk factors and management
23. acuterenalfailure in pregnancy 1987
24. adolescent pregnancy occurrence and consequences
25. adult highrisk burkitt’s acute lymphocytic leukemia was successfully rescued by rituximab combined with hypercvad/ma regimens two case reports and a literature review
26. adult highrisk burkitt's acute lymphocytic leukemia was successfully rescued by rituximab combined with hypercvad/ma regimens two case reports and a literature review
27. advances of medical termination of pregnancy amendments act in india
28. alternatives to mifepristone for early medical abortion
29. amniocentesis before 15 weeks' gestation technical aspects and obstetric risks
30. anticonvulsant therapy for status epilepticus
31. antioxidants for male subfertility
32. antiphospholipid syndrome
33. antiprogesterones for the interruption of pregnancy
34. cabergoline a review of its pharmacological properties and therapeutic potential in the treatment of hyperprolactinaemia and inhibition of lactation
35. canadian contraception consensus (part 2 of 4)
36. candida sepsis in pregnancy and the postpartum period
37. catastrophic antiphospholipid syndrome during pregnancy and puerperium maternal and fetal characteristics of 15 cases
38. causes of maternal death in ethiopia between 1990 and 2016 systematic review with metaanalysis
39. cervical ectopic pregnancy diagnosis with endovaginal ultrasound examination and successful treatment with methotrexate
40. cervical pregnancy a rare case of reimplantation after abortion a case report
41. change in abortion services after implementation of a restrictive lawin texas
42. cleavage鈥恠tage versus blastocyst鈥恠tage embryo transfer in assisted reproductive technology
43. clinical applications of mifepristone
44. comparative effectiveness, safety and acceptability of medical abortion at home and in a clinic a systematic review
45. comparison of mifepristone plus misoprostol with misoprostol alone for first trimester medical abortion a systematic review and metaanalysis
46. complications after second trimester surgical and medical abortion
47. complications of misoprostol and other abortion induction methods in the developing world a systematic review
48. contraception and diabetes mellitus
49. control of tropical theileriosis (theileria annulata infection in cattle) in north africa
50. coping with fertility in israel a casestudy of culture clash
51. cordarone in obstetrics may or not?
52. craniorachischisis with incomplete twins diprosopus report of a case and review of the literature
53. crisis pregnancy centers in the us lack of adherence to medical and ethical practice standards a joint position statement of the society for adolescent health and medicine and the north american society for pediatric and adolescent gynecology society for adolescent health and medicine and the north american society for pediatric and adolescent gynecology
54. culture media for human pre鈥恑mplantation embryos in assisted reproductive technology cycles
55. current and potential methods for second trimester abortion
56. current management of complete and partial molar pregnancy
57. current management of molar pregnancy
58. current medical abortion care
59. diagnosis of medulloblastoma in pregnant women to continue with the pregnancy or not? a case report and literature review
60. die neuere entwicklung des arztrechts
61. does starting progestinonly contraception on the same day as a medical abortion with mifepristone affect completion of the abortion? a review of clinic records from mexico city
62. early pregnancy failure current management concepts
63. ectopic pregnancy a lifethreatening gynecological emergency
64. effectiveness and acceptability of "at home" versus "at hospital" early medical abortion a lesson from the covid19 pandemic a retrospective cohort study
65. effectiveness and acceptability of “at home” versus “at hospital” early medical abortion a lesson from the covid19 pandemic a retrospective cohort study
66. effectiveness and acceptability of home use of misoprostol for medical abortion up to 10 weeks of pregnancy
67. efficacy of medical abortion prior to 6 gestational weeks a systematic review
68. efficacy of misoprostol alone for firsttrimester medical abortion a systematic review
69. efficacy, safety, and acceptability of misoprostol in the treatment of incomplete miscarriage a systematic review and metaanalysis
70. elevated mrna expression of pgf2α receptor splice variant 2(fpv2) in human decidua is associated with incomplete mifepristonemisoprostolinduced early medical abortion by regulation of interleukin8
71. emergency contraception presently available formulations and controversies surrounding their use
72. endometrial preparation for women undergoing embryo transfer with frozen embryos or embryos derived from donor oocytes
73. epidemiology
74. ethical issues in prenataldiagnosis
75. evaluating women's acceptability of treatment of incomplete second trimester abortion using misoprostol provided by midwives compared with physicians a mixed methods study
76. evidence for shortening the time interval of prostaglandin after mifepristone for medical abortion
77. experiences of midwives and nurses when implementing abortion policies a systematic integrative review
78. expulsion and continuation rates after postabortion insertion of framed iuds versus frameless iuds review of the literature
79. expulsion at home for early medical abortion a systematic review with metaanalyses
80. factors affecting the outcome of early medical abortion a review of 4132 consecutive cases
81. failed early medical abortion beware of the uterine scar!case report
82. fatal hemorrhage from legalabortion in the unitedstates
83. fatal septic abortion in the united states, 19751977
84. fatal septic abortion in the unitedstates, 19751977
85. fetal complications associated with unsuccessful attempts at termination of pregnancy
86. fetal death ratios in a prospectivestudy compared to state fetal death certificate reporting
87. first trimester bleeding
88. first trimester termination of pregnancy
89. firsttrimester medical abortion with mifepristone 200 mg and misoprostol a systematic review
90. followup strategies to confirm the success of medical abortion of pregnancies up to 10 weeks’ gestation a systematic review with metaanalyses
91. followup strategies to confirm the success of medical abortion of pregnancies up to 10 weeks' gestation a systematic review with metaanalyses
92. from concept to practice the recent history of preterm delivery prevention part ii subclinical infection and hormonal effects
93. future direction of abortion technology
94. ghana
95. gonadotrophins for ovulation induction in women with polycystic ovary syndrome
96. growth hormone for in vitro fertilisation (ivf)
97. homocysteine, folic acid and vitamin b12 concentration in patientswith recurrent miscarriages
98. honour and interests medical ethics in britain and the work of the british medical association's central ethical committee, 19021939 (bl dxn050009)
99. hysteroscopic resection or conventional blind curettage in the management of retained products of conception, a systematic review
100. implication of sperm chromosomal abnormalities in recurrent abortion and multiple implantation failure
101. induction of abortion in the second trimester of pregnancy by intramuscular 15methyl prostaglandin f(2α) 15m)
102. informed consent or institutionalized eugenics? how the medical profession encourages abortion of fetuses with down syndrome
103. integrating gender perspectives in gynecology and obstetrics engaging medical colleges in maharashtra, india
104. interruption of nonviable pregnancies of 2428 weeks' gestation using medical methods release date june 2013 sfp guideline #20133
105. intracytoplasmic sperm injection versus conventional in vitro fertilisation in couples with males presenting with normal total sperm count and motility
106. investigation and treatment of amenorrhoea resulting in normal fertility
107. is a cleft lip and palate a serious "handicap"? jepson v chief constable of west merciaa legal and ethical critique
108. is preimplantation genetic diagnosis the ideal embryo selection method in aneuploidy screening?
109. is ward evacuation for uncomplicated incomplete abortion under systemic analgesia safe and effective? a randomised clinical trial
110. it's a small bit of advice, but actually on the day, made such a difference… perceptions of quality in abortion care in england and wales
111. late midtrimester medical pregnancy terminations three different procedurew with prostaglandin f2α and laminaria tents
112. legal issues in the provision of medical abortion
113. lowsensitivity urine pregnancy testing to assess medical abortion outcome a systematic review
114. malpractice issues in radiology wrongful life
115. management and treatment of brain tumors during pregnancy an italian survey
116. management of maternal amanita phalloïdes poisoning during the first trimester of pregnancy a case report and review of the literature
117. management of maternal amanita phalloides poisoning during the first trimester of pregnancy a case report and review of the literature
118. management of pain associated with upto9weeks medical termination of pregnancy (mtop) using mifepristonemisoprostol regimens expert consensus based on a systematic literature review
119. maternal and neonatal listeriosis report of case and brief review of literature of listeriosis in man
120. media reviews
121. medical abortion at 64 to 91 days of gestation a review of 483 consecutive cases
122. medical abortion at 913 weeks' gestation a review of 1076 consecutive cases
123. medical abortion in the community setting
124. medical abortion in the first trimester
125. medical abortion offered in pharmacy versus clinicbased settings
126. medical abortion with mifepristone and vaginal misoprostol between 64 and 70days' gestation
127. medical management of induced and incomplete firsttrimester abortion by nonphysicians in low and middleincome countries a systematic review and metaanalysis of randomized controlled trials
128. medical methods for first trimester abortion
129. medical methods for midtrimester termination of pregnancy
130. medical methods to terminate early pregnancy
131. medical methods to terminate earlypregnancy
132. medical termination for pregnancy in early first trimester (≤ 63 days) using combination of mifepristone and misoprostol or misoprostol alone a systematic review
133. medication abortion
134. methods for induced abortion
135. methotrexate/misoprostol embryopathy report of four cases resulting from failed medical abortion
136. midtrimester medical termination of pregnancy a review of 1002 consecutive cases
137. mifepristone (ru 486) current knowledge and future prospects
138. mifepristone with buccal misoprostol for medical abortion
139. mifepristone with buccal misoprostol for medical abortion a systematic review
140. mifepristone ten years later
141. misoprostol for women's health a review
142. misoprostol versus suction evacuation in the management of patients with incomplete abortion a scoping review
143. misoprostolinduced acute coronary syndrome in a premenopausal woman a case report with literature review
144. modern methods to induce abortion safety, efficacy and choice
145. new york appeals court finds state not liable for death after physician under review performed abortion
146. normal and abnormal early pregnancy
147. office management of early pregnancy loss
148. options for early therapeutic abortion a comparative review
149. outcome of first trimester medical termination of pregnancy definitions and management
150. pathophysiological and clinical aspects of combat anticholinesterase poisoning
151. pathophysiology of mifepristoneinduced septic shock due to clostridium sordellii
152. practices and knowledge of female gynecologists regarding contraceptive use a realworld chinese survey
153. profile of women receiving secondtrimester safe abortion service at paropakar maternity and women's hospital
154. prostaglandin analogs and their uses
155. provision of abortion by midlevel providers international policy, practice and perspectives
156. psychological sequelae of inducedabortion
157. psychosocial aspects of induced abortion
158. reproductive choice for women and men living with hiv contraception, abortion and fertility
159. reproductive failure due to spontaneous abortion and recurrent miscarriage
160. reproductive justice discourse visàvis abortion law in india a critical review
161. restrictive lung disease in pregnancy
162. restrictive lungdisease in pregnancy
163. review of the feasibility of mifepristone & misoprostol for medical abortion in developing countries
164. ru 486 (mifepristone) "the abortive pill" our experience
165. second trimester medical abortion with mifepristone and gemeprost a review of 956 cases
166. second trimester medical abortion with mifepristonemisoprostol and misoprostol alone a review of methods and management
167. secondary infertility and the aging male, overview
168. selfadministered versus provideradministered medical abortion
169. septic abortion
170. sequelae of premature sexual life
171. simultaneous compared to interval administration of mifepristone and misoprostol for medical abortion up to 10 +0 weeks' gestation a systematic review with metaanalyses
172. simultaneous compared to interval administration of mifepristone and misoprostol for medical abortion up to 10(+0) weeks' gestation a systematic review with metaanalyses
173. simultaneous compared with interval medical abortion regimens where home use is restricted correction
174. status epilepticus in pregnancy a literature review and a protocol proposal
175. success rate of secondtrimester termination of pregnancy using misoprostol
176. surgical or medical abortion of pregnancies between 13 +0 and 23 +6 weeks' gestation? a systematic review and new nice national guidelines
177. surgical or medical abortion of pregnancies between 13(+0) and 23(+6) weeks' gestation? a systematic review and new nice national guidelines
178. systematic review of the effectiveness, safety, and acceptability of mifepristone and misoprostol for medical abortion in low and middleincome countries
179. systematic review the maternal mortality myth in the context of legalized abortion
180. targeted client communication via mobile devices for improving sexual and reproductive health
181. teenage pregnancy and motherhood a review of the literature
182. telemedicine for medical abortion a systematic review
183. ten years on a review of medical terminations of pregnancy performed in a sexual health clinic
184. termination of pregnancy services in irish general practice from january 2019 to june 2019
185. the clinical management of repeated earlypregnancy wastage
186. the contribution of environmental teratogens to embryonic and fetal loss
187. the efficacy of medical abortion a metaanalysis
188. the efficacy of shenghua decoction supplementation after early medical abortion a metaanalysis of randomized controlled trials
189. the impact of provider restrictions on abortionrelated outcomes a synthesis of legal and health evidence
190. the influence of abortion legislation on maternal mortality
191. the montana fetal genetic pathology program and a review of prenatal death in humans
192. the perimenopause and contraception
193. the psychosocial factors of the abortion experience a critical review
194. the use of prostaglandins and antiprogestins for pregnancy termination
195. unsafe abortion an avoidable tragedy
196. when is "emergency" contraception the right name for postcoital treatment?
197. women's voices and medical abortions a review of the literature

**3.3 Records excluded: Non-English language records (n=212)**

1. [74 requests for interruption of pregnancy after failure of oral contraception (author's transl)]
2. [a family planning center role of the nurse]
3. [a phase iii multicentre study on medical termination of early pregnancy with two regimens of mifepristone followed by pg05]
4. [a survey on serious accidents associated with abortion before and after the law of 1975]
5. [abortion law in france]
6. [acute renal insufficiency in pregnancy and the postpartum period]
7. [ambulatory medical abortion performed in a family planning center]
8. [analysis of the results and considerations on the organizational aspects of the implementation of the law 194/78 in a provincial hospital]
9. [antiprogesterone for medical abortion]
10. [apf open letter to the political parties, on the abortion situation in portugal]
11. [cervical pregnancy a rare case of reimplantation after abortion a case report]
12. [change in attitudes about illegal abortion]
13. [chorionic villus needle sampling by the transabdominal route or by placental centesis a series of 930 cases]
14. [clinical study of early pregnancy termination by administration of dl15methyl prostaglandin f2 alpha combined with tamoxifen or norethisterone]
15. [clinical study of four cases with malignant gestation trophoblastic tumor after mifepristone abortion]
16. [clinical study of terminating biochemical pregnancy and early clinical pregnancy with mifepristone and misoprostol]
17. [clinical study of termination of early pregnancy by combination of dl15methylprostaglandin f2 alpha and ru 486]
18. [clinical study on 39 cases with caesarean scar pregnancy with sonographic mass]
19. [comparison of clinical outcomes of vitrifiedthawed embryo transfer and fresh embryos transfer]
20. [complications of induced abortions]
21. [conflicting recommendations between the french national authority for health (has) and clinical practice guidelines (cngof); focus on 200 late medical abortions, conducted outside marketing authorizations]
22. [contraception in adolescence knowing is not enough]
23. [contraception]
24. [contraceptive compliance why is contraceptive failure still so frequent?]
25. [contraceptive efficacy]
26. [craniorachischisis in conjoined "diprosopus" twins case report and review of the literature]
27. [data for reform of french legislation on abortion]
28. [demand for abortion special aspects of druginduced abortion]
29. [desire for pregnancy, refusal of pregnancy, ambivalence in family planning]
30. [effects of taking estrogen and progestogen after medical abortion on reducing vaginal hemorrhage time a randomizedcontrolled trial]
31. [evaluation of the success of medical abortion by a plasma hcg control threshold]
32. [experiences with the antigestagen mifepristone (ru 486) in the interruption of early pregnancy]
33. [factors associated with the failure of medical treatment for ectopic pregnancy case study conducted at the yaounde gynaecology, obstetrics and pediatrics hospital]
34. [food contaminated by metals a study of health consequences in a limited region]
35. [human rights, maternal mortality and reproductive health]
36. [if you were to have another abortion, would you choose the same method? a study on 1032 patients' level of satisfaction]
37. [improving the quality of care after spontaneous abortions in rural senegal]
38. [induced abortion epidemiological study after eight years of enforcement of law 194]
39. [induced abortion legislation, epidemiology, complications]
40. [induced abortion a vulnerable public health problem]
41. [interregional project concerning abortion]
42. [intrauterine devices in the immediate, early and late postabortion period]
43. [large variation in clinical regimens use to induce medical abortion in denmark]
44. [male contraception]
45. [management of pheochromocytoma during pregnancy about three cases]
46. [maternal and fetal outcomes with aortic dissection in pregnant patients with marfan syndrome]
47. [medical abortion at home and at hospital a trial of efficacy and acceptability]
48. [medical abortion for firsttrimester termination of the pregnancy]
49. [medical abortion provided by telemedicine to women in latin america complications and their treatment]
50. [medical abortion using methotrexate and misoprostol efficacy and tolerability]
51. [medical and sociodemographic implications of abortion at felix bulnes hospital in santiago (author's transl)]
52. [medical claims and women's experience physicianperformed abortions in the weimar republic]
53. [medical genetics in reproductive medicine]
54. [medical termination of pregnancy with mifepristoneinitial experience at the sheba medical center]
55. [medicallyinduced abortion and risk of reproductive failures in subsequent pregnancy]
56. [mifepristone 100 mg for early medical abortion]
57. [misoprostol offlabel use in the first trimester of pregnancy (spontaneous abortion, and voluntary medical termination of pregnancy)]
58. [moebius syndrome due to the use of misoprostol case report]
59. [nonsurgical female sterilization using quinacrine efficacy of two insertions of quinacrine pellets]
60. [ovarian function in systemic lupus erythematosus patients undergoing the use of cyclophosphamide in two major rheumatologic care centers in curitiba, parana state]
61. [physicians confront abortion]
62. [postabortion counseling]
63. [pregnancy and cardiac surgery with cardiopulmonary bypass]
64. [prescribing of ergometrine for abortion without physical examination]
65. [psychological decision making with regard to motherhood by women with highrisk pregnancy and normal pregnancy]
66. [retrospective assessment of the influence of gestational age on the first trimester medical abortion efficiency]
67. [sex education prerequisite for family planning]
68. [should antiprogesterone be used in pregnancy termination?]
69. [side effects of induced abortion results from a populationbased survey]
70. [sociopsychological aspects of voluntary abortion at the obstetrical and gynecological clinic of catania]
71. [std prevention and unplanned pregnancies in great britain]
72. [sterilization as a method of family planning]
73. [study on leuprorelin acetate in treatment of uterine adenomyosis with infertility]
74. [termination of early pregnancy by two regimens of mifepristone with misoprostol a multicentre clinical trial]
75. [termination of early pregnancy using ru 486 alone or in combination with prostaglandin the ru 486 collaboration group]
76. [termination of pregnancy in the 2nd trimester using intraamniotic administration of prostaglandins]
77. [the applied of computer for analysis on contraceptive efficacy of iud of rural women in guangdong province]
78. [the efficacy and acceptability of mifepristone medical abortion with home administration misoprostol provided by private providers linked with the hospital a prospective study of 433 patients]
79. [the experience of carrying out the technical responsibility system in the birth control of peng county, sichuan province]
80. [the fetal adrenal gland in risk pregnancy]
81. [the fetal adrenal gland in risk pregnancy]; [nadbubrezne zlezde fetusa u rizicnim trudnoćama]
82. [the first 100 early medical abortions]
83. [the gynecologist and the problem of therapeutic abortion]
84. [the value of irreversible contraception]
85. [trends in the indications for termination of pregnancy in the last 10 years in switzerland]
86. [update in current care guidelines induced abortion]
87. [use of misoprostol in gynecology and obstetrics]
88. [value of hysteroscopy and laparoscopy in differential diagnosis of gestational trophoblastic neoplasia]
89. [voluntary interruption of pregnancy not an insignificant act]
90. 74 requests for interruption of pregnancy after failure of oral contraception; [74 demandes d'interruption de grossesse sur echec de la contraception orale; quelques reflexions]
91. a family planning center role of the nurse; [un centre de planification familiale place et rôle de l'infirmière]
92. a few concerns about bioethics; [quelques préoccupations au sujet de la bioéthique]
93. a method of abortion and a danger to the woman's health how young poles perceive hormonal postcoital contraception; ["metoda aborcji i zagrożenie zdrowia kobiety" czyli jak młodzi polacy postrzegaja̧ hormonalna̧ antykoncepcjȩ postkoitalna̧]
94. a survey on serious accidents associated with abortion before and after the law of 1975; [enquete sur les accidents graves de l'avortement avant et apres la loi de 1975]
95. abortion at the first trimester of pregnancy the risks of responsibility from jurisprudence and the study of 81 complaints; [l'ivg du premier trimestre et ses risques d'engagement de la responsabilite]
96. abortion in belgium, 18801940; [abortus in belgië 18801940]
97. abortion in brazil what do the official data say?; [aborto no brasil o que dizem os dados oficiais?]; [aborto en brasil ¿qué dicen los datos oficiales?]
98. abortion practices in high school students in yamoussoukro, côte d’ivoire; [pratiques d’avortement chez des lycéennes à yamoussoukro, côte d’ivoire]
99. an account of 2180 cases of percoelioscopic tubal ligation using yoon's mechanical method; [a propos de 2180 sterilisations tubaires percoelioscopiques par procede mecanique de yoon]
100. analysis of clinical characteristics and prognosis of 21 pregnant women complicated with tuberculosis after in vitro fertilizationembryo transfer; [21例体外受精胚胎移植后妊娠并发结核病患者的 临床特征及预后分析]
101. apf open letter to the political parties, on the abortion situation in portugal; [apf carta aberta aos partidos politicos, sobre a situacao do aborto em portugal]
102. application effect of different ovarian stimulation protocols in elderly patients with diminished ovarian reserve; [不同促排卵方案在高龄卵巢储备功能减退患者中的应用效果比较]
103. aspects of the significance of operatively corrected heart valve failure on fertility; [aspekte zur bedeutung operativ korrigierter herzklappenvitien auf die fertilitat]
104. assessing an ovarian reserve and risk factors for premature ovarian failure as part of preabortion counseling for women under 40 planning to terminate own first pregnancy; [исследование овариального резерва и факторов риска преждевременной недостаточности яичников в рамках доабортного консультирования женщин в возрасте до 40 лет, планирующих прервать первую беременность]
105. assessment of efficacy and safety of medical treatment of nonviable first trimester pregnancy; [ocena skuteczności i bezpieczeństwa leczenia zachowawczego w przypadkach ciąży obumarłej w pierwszym trymestrze]
106. assessment of results and notes on the organisational aspects of law no 194 of 1978 as applied in a provincial hospital interruption of pregnancy; [analisi dei risultati, considerazioni di ordine organizzativo circa la legge 194/78 applicata in un ente ospedaliero di provincia]
107. avoiding abortion stigma how the “invisibilization labour” reinforces the procreative labour; [esquiver le stigmate lié à l’avortement le « travail d’invisibilisation» comme renforcement du travail procréatif]
108. birth control and social group contraception, accidental pregnancies and abortion; [maitrise de la fecondite et appartenance sociale contraception, grossesses accidentelles et avortements]
109. causative factors of premature birth (a new look at the problem); [причинные факторы преждевременных родов (новый взгляд на проблему)]; [причинні фактори передчасних пологів (новий погляд на проблему)]
110. cervical pregnancy a rare case of reimplantation after abortion a case report; [la réimplantation cervicale de la grossesse, une complication méconnue des interruptions volontaires de grossesse à propos d'un cas]
111. change in attitudes about illegal abortion; [panoramawandel illegaler schwangerschaftsabbrüche]
112. clinical outcomes of luteal phase stimulation for in vitro fertilization/intracytoplasmic sperm injection treatment in poor ovarian responders; [卵巢低反应患者黄体期促排卵行体外受精/卵胞质内单精子显微注射助孕临床结局分析]
113. clinical research of sequential embryo transfer in frozen thawed cycles of patients with recurrent implantation failure; [解冻周期序贯移植治疗反复种植失败的临床研究]
114. comment "analysis of and reflections on some abortion request statistics" (letter); [a propos de "analyse d'une statistique de demandes d'ivg et les reflexions qu'elle suscite"]
115. comparative effectiveness, safety and acceptability of medical abortion at home and in a clinic a systematic review; [comparación de la efectividad, la seguridad y la aceptación de los abortos médicos practicados en el domicilio con aquellos realizados en la clínica una revisión sistemática]
116. compare of misoprostol and dinoprost effectivity by induced secondtrimester abortion; [srovnání efektivity misoprostolu a dinoprostu při indukci druhotrimestrálního abortu]
117. complications of induced abortions; [complications des interruptions volontaires de grossesse]
118. conflicting recommendations between the french national authority for health (has) and clinical practice guidelines (cngof); focus on 200 late medical abortions, conducted outside marketing authorizations; [recommandations discordantes entre has et cngof; le point avec 200 ivg médicamenteuses tardives, réalisées hors amm]
119. congenital heart disease and acquired valvular lesions in pregnancy; [angeborene herzfehler und erworbene herzklappenfehler in der schwangerschaft]
120. contraception in adolescence knowing is not enough; [la contraception à l'adolescence savoir ne suffit pas]
121. contraception; [la contraccezione]
122. contraceptive compliance why is contraceptive failure still so frequent?; [kontrazeptive compliance warum kommt es immer wieder zum versagen der kontrazeptiven therapie?]
123. contraceptive efficacy; [a eficacia contraceptiva]
124. crimes related to illegally carrying out an artificial termination of pregnancy (abortion) in the legislation of foreign countries; [преступления, связанные с незаконным проведением искусственного прерывания беременности (аборта), в законодательстве зарубежных стран]
125. cuban health system call it superior? [letter]
126. current diagnostics and treatments for immune mediated recurrent spontaneous miscarriages and ivf implantation failures a review; [az immunpatológiai hátteru visszatéro spontán vetélések és sikertelen ivf beültetések korszeru diagnosztikája és kezelési lehetoségei]
127. data for reform of french legislation on abortion; [eléments pour une réforme de la législation française de l'avortement]
128. demand for abortion special aspects of druginduced abortion; [la demande d'avortement aspects particuliers de l'ivg médicamenteuse]
129. developmental toxicity of misoprostol an update; [toxicidad del misoprostol sobre la gestación revisión de la literatura]
130. diagnosis and clinical criteria for chronic endometritis; [диагностика и клинические критерии хронического эндометрита]
131. disruptive congenital anomalies associated with misoprostol case report; [anomalías congénitas disruptivas asociadas con misoprostol reporte de un caso]
132. do the risks of a diagnostic xray during early pregnancy justify therapeutic abortion?; [les risques d'un examen de radiodiagnostic au debut de la gestation justifientils l'interruption de celleci?]
133. doctors speak of their experience in announcing a diagnosis announcing to parents seriouschronic disease in their child; [des médecins parlent de leur expérience de l'annonce l'annonce d'une maladie grave de l'enfant à ses parents]
134. economic cost of pregnancy care attributable to the failure of mexico's teenage pregnancy prevention policy; [costo económico de atención de embarazos atribuibles a la falla de la política de prevención del embarazo adolescente en méxico]
135. effects of growth hormone in downregulating hormone replacement cycle on the outcome of frozenthawed embryo transfer; [降调节后激素替代方案中应用生长激素 对冻融胚胎移植周期结局的影响]
136. effects of laserassisted hatching on the clinical outcome of freezethawedcleavage embryo transfer in patients with repeated implantation failure; [激光辅助孵化对反复种植失败患者行冻融卵裂期胚胎移植临床结局的影]
137. effects of neonatal intensive care on the mortality rate of idiopathic respiratory distress syndrome; [influencia de los cuidados intensivos neonatales en la mortalidad del sindrome de la dificultad respiratoria idiopatica]
138. efficacy and safety of uterine tamponade to control bleeding and reduce obstetric hysterectomy historic cohort in nuevo león, méxico, 2013; [eficacia y seguridad del taponamiento uterino para control de hemorragia y disminución de histerectomía obstétrica cohorte histórica en nuevo león, méxico, 2013]
139. efficacy, safety, and acceptability of misoprostol in the treatment of incomplete miscarriage a systematic review and metaanalysis; [eficacia, seguranca e aceitabilidade do misoprostol no tratamento do aborto incompleto uma revisao sistem tica e metanalise]
140. emergency contraception for adolescents; [la contraccezione d'emergenza in adolescenza considerazioni generali e proposte di un gruppo di lavoro]
141. evaluation of irreversible contraception; [zum stellenwert der irreversiblen kontrazeption]
142. evaluation of the success of medical abortion by a plasma hcg control threshold; [évaluation du succès de l'interruption volontaire de grossesse médicamenteuse par un seuil d'hcg sérique]
143. evaluation of the teratogenic risks in gestations exposed to misoprostol; [avaliação de riscos teratogênicos em gestações expostas ao misoprostol]
144. experiences and prophylactic of panic attacks the case of magnetic resonance imaging; [doświadczanie i proflaktyka ataku paniki casus obrazowania rezonansem magnetycznym]
145. factors associated with the failure of medical treatment for ectopic pregnancy case study conducted at the yaoundé gynaecology, obstetrics and pediatrics hospital; [facteurs associés à l’échec du traitement médical de la grossesse extrautérine cas de l’hôpital gynecoobstétrique et pédiatrique de yaoundé]
146. factors associated with the outcomes of preimplantation genetic testing in assisted reproduction; [胚胎种植前遗传学检测助孕妊娠结局的影响因素研究]
147. factors associated with the persistence of retained products following d and c is curettage still the treatment of choice?; [factores que se asocian a la persistencia de restos abortivos tras legrado evacuador sigue siendo el curetaje el tratamiento de eleccion?]
148. fertility, gestational and systemic problems in turner's syndrome two case reports; [turner sendromunda fertilite, gestasyonel ve sistemik sorunlar iki olgu sunumu]
149. fetal complications associated with unsuccessful attempts at termination of pregnancy; [fetale komplikationen nach misslungenem schwangerschaftsabbruch im ersten trimester]
150. four perspectives of women's health workshop participants talk about women's health issues in four countries [hong kong]
151. gnrh‐a combined fertility‐sparing re‐treatment in women with endometrial carcinoma or atypical endomertial hyperplasia who failed to oral progestin therapy; [gnrha 联合治疗用于口服孕激素治疗失败的子宫内膜非典型增生及子宫内膜癌患者的探讨]
152. gregory v pembrokeshire health authority [31 january 1989]
153. human rights, maternal mortality and reproductive health; [derechos humanos, mortalidad materna y salud reproductiva]
154. illegal abortion with misoprostol in guadeloupe; [avortements illégaux par le misoprostol en guadeloupe]
155. immunological aspects of pregnancy failures novel therapeutic approaches; [sikertelen terhességek immunológiai háttere újabb terápiás lehetoségek]
156. importance of a second dose of misoprostol in the management of medical abortion after failure of the initial treatment; [intérêt de l'administration d'une seconde dose de misoprostol dans la prise en charge de l'interruption volontaire de grossesse après échec du traitement médical initial]
157. improving the quality of care after spontaneous abortions in rural senegal; [amélioration de la qualité des soins après avortement (saa) en zone rurale au sénégal]
158. induced abortion a vulnerable public health problem; [el aborto inducido un problema de salud publica vulnerable]
159. induction of abortion during the second trimenon of pregnancy endocervical pge2 gel application, intramuscular application of sulproston and combined treatment (endocervical pge2 gel/intramuscular sulproston); [abortinduktion im zweiten schwangerschaftstrimenon endozervikale pge2gelapplikation, intramuskulare sulprostonapplikation und kombinierte (endozervikale pge2gel/intramuskulare sulproston) behandlung]
160. influence of two endometrial preparation programs independent of endogenous ovarian cycle on the pregnancy outcome of thin endometrial patients with freezethaw embryo transfer; [两种非依赖内源性卵巢周期的内膜准备方案对薄型子宫内膜冻融胚胎移植患者妊娠结局的影响研究]
161. interpretation to medication abortion up to 70 days of gestation • acog practice bulletin , number 225; [2020 agog实践简报孕龄<70 d药物 流产(no 225)》解读]
162. interregional project concerning abortion; [projet interregional sur l'ivg]
163. intrauterine devices in the immediate, early and late postabortion period; [dispositivos intrauterinos en el postaborto inmediato, precoz y tardío]
164. investigation of hepatitis b virus seroprevalence in hepatitis c infected patients; [hepatit c virüsü ile enfekte hastalarda hepatit b seroprevalansinin araştirilmasi]
165. is mifepristone 100mg an effective alternative to standard dose for medical abortion; [tıbbi abortus için 100 mg mifepriston standart doza etkili bir alternatif midir?]
166. male contraception; [la contraception masculine]
167. management of the uterine abnormalities on the reproductive outcomes in women with repeated implantation failure; [宫腔异常治疗对反复种植失败患者助孕结局的影响]
168. medical claims and women's experience physicianperformed abortions in the weimar republic; [heilanspruch und medizinische kunstfehler abtreibungen durch arzte in der weimarer republik offizielle beurteilung und weibliche erfahrung]
169. medical genetics in reproductive medicine; [vyuzití lékarské genetiky v reprodukcní medicíne]
170. medical responsibility in the practice of voluntary first trimester terminations of pregnancy preventive measures drawn from jurisprudence and from the study of 50 files of legal action or threatened legal action; [responsabilite medicale dans la pratique des interruptions volontaires de grossesse du premier trimestre mesures preventives tirees de la jurisprudence et de l'etude de 50 dossiers de plaintes ou de menaces de plaintes]
171. medical treatment of spontaneous abortion in the first trimester; [tratamiento médico del aborto espontáneo del primer trimestre]
172. medicallyinduced abortion and risk of reproductive failures in subsequent pregnancy; [medicinsk induceret abort og risiko for bivirkninger ved en efterfølgende graviditet sekundærpublikation]
173. medicalsocial aspects pregnancy, labor and puerperium in patients the solitary mother home in karwowo near szczecin; [medycznospołeczne aspekty przebiegu ciazy, porodu i połogu u pacjentekpensjonariuszek domu samotnej matki w karwowie koło szczecina]
174. modern techniques of medical pregnancy termination; [les techniques modernes d'interruption medicale de la grossesse]
175. moebius syndrome due to the use of misoprostol case report; [sindrome de moebius fetopatía por misoprostol reporte de un paciente]
176. on inhuman practices in gynaecology and their victims in germany during national socialist rule a study of concrete events; [uber inhumane praktiken der frauenheilkunde im nationalsozialismus und ihre opfer untersuchung zu konkreten ereignissen]
177. ovarian function in systemic lupus erythematosus patients undergoing the use of cyclophosphamide in two major rheumatologic care centers in curitiba, paraná state; [função ovariana em mulheres lúpicas submetidas ao uso de ciclofosfamida em dois grandes centros de atendimento reumatológico de curitiba, estado do paraná]
178. perinatal infection with the human immunodeficiency virus; [perinatalna infekcija virusom humane imunodeficijencije]
179. perinatal prophylaxis with immunoglobulin antid and the impact on rhd sensitizations among pregnant women in slovenia; [perinatalna zaščita z imunoglobulinom antid in vpliv senzibilizacije na antigen rhd med nosečnicami v sloveniji]
180. pharmacological voluntary interruption of pregnancy in a rural area; [seguridad en la interrupción voluntaria del embarazo farmacológica en un entorno rural]
181. postabortion counseling; [l'entretien postivg]
182. postnatal cardiac outcomes associated with the prenatal diagnosis of absence of ductus venosus; [evolución cardiológica postnatal y factores asociados a la agenesia de ductus venoso de diagnóstico prenatal]
183. postpartum contraception in china status, problems and coping strategies; [我国产后避孕现状和面临的问题及应对策略]
184. pregnancy and cardiac surgery with cardiopulmonary bypass; [terhesség és nyitott szívmutét]
185. prescribing of ergometrine for abortion without physical examination; [voorschrijven van ergometrine wegens abortus zonder onderzoek]
186. preterm infant born to a mother with severe pandemic h1n1 influenza; [aǧır pandemik h1n1 i̇nfluenzalı anneden erken doǧan bebek]
187. psychological decision making with regard to motherhood by women with highrisk pregnancy and normal pregnancy; [psychologiczne uwarunkowania decyzji o macierzyństwie kobiet w ciazy wyosokiego ryzyka i w ciazy prawidłowej]
188. psychopathology of recurrent spontaneous abortions; [fausses couches a repetition origine psychogene et/ou psychopathologie induite]
189. rendering follicle tubes permeable again after postpartum sterilization a report on 25 cases, should postpartum sterilization be performed or not?; [repermeabilisation tubaire apres sterilisation du postpartum a propos de 25 cas, fautil remettre en cause la sterilisation du postpartum?]
190. report of two cases of cervical pregnancy treated in a divergent way; [embarazo cervical tratado de forma divergente reporte de dos casos]
191. return of fertility after discontinuation of contraception according type of contraception, duration of use, age and body mass index; [kembalinya kesuburan setelah penghentian alat kontrasepsi berdasarkan jenis kontrasepsi, lama pemakaian, usia dan indeks massa tubuh]
192. rheumatoid arthritis and pregnancy a case report and literature review; [artritis reumatoide y embarazo reporte de un caso y revisión de la bibliografía]
193. ru 486 (mifepristone) from clinical trials to the perspectives for clinical use; [ru 486 (mifepristone) des essais cliniques aux perspectives d'utilisation]
194. sequelae of reentry of tachycardia during intrauterine development and in the newborn period; [dôsledky reentry tachykardie prítomnej počas vnútromaternicového vývoja a v novorodeneckom období]
195. seroprevalence of babesia caballi and theileria equi in horses in central germany; [vorkommen von antikörpern gegen babesia caballi und theileria equi bei pferden in mitteldeutschland]
196. sex education prerequisite for family planning; [education sexuelle prealable necessaire a la planification familiale]
197. sociocultural and obstetric risk factors associated with incomplete abortion in peruvian women; [factores de riesgo socioculturales y obstétricos asociados al aborto incompleto en mujeres peruanas]
198. sociopsychological aspects of voluntary abortion at the obstetrical and gynecological clinic of catania; [aspetti sociopsicologici dell'aborto volontario nella clinica ostetrica e ginecologica di catania]
199. std prevention and unplanned pregnancy a uk perspective; [prevention des mst et grossesses indesirees en grandebretagne]
200. the clinical aspects of acute renal failure, with particular reference to the postpartum period; [aspetti clinici dell'insufficienza renale acuta con particolare riguardo all'ira postpartum]
201. the development of medical law in the field of gynecology and the relationship with criminal law the applicability of the legal framework of the republic of kosovo; [rozwój prawa medycznego w dziedzinie ginekologii a jego związek z prawem karnym obowiązywanie ram prawnych republiki kosowa]
202. the effect of smoking on reproductive failures in couples examined in the genetic outpatient clinic at dr a jurasz university hospital in bydgoszcz; [rozpowszechnienie palenia tytoniu wśród par z niepowodzeniami cia̧żowymi z poradnigenetycznej szpitala uniwersyteckiego im dr a jurasza w bydgoszczy]
203. the effects of electromagnetic field on pregnancy; [elektromanyetik alanin gebelik üzerine etkileri]
204. the gynecologist and the problem of therapeutic abortion; [le medecin gynecologue face au problème de l'avortement thérapeutique]
205. the induction of abortion in the second trimester by combined administration of minprostin and sulproston, compared with the use of sulproston alone; [kombinierter einsatz von minprostin und sulproston bei der aborteinleitung im ii trimenon und bei der geburtseinleitung beim toten kind im vergleich zum alleinigen sulprostoneinsatz]
206. the usefulness of the chorionic villi technic in prenatal diagnosis; [korisnost korion frondozum tehnike u prenatalnoj dijagnozi]
207. ultrasound in republic of yemen; [echographie en republique du yemen]
208. use of misoprostol in gynecology and obstetrics; [utilisation du misoprostol en gynécologieobstétrique]
209. uterine rupture during pregnancy and delivery risk factors, symptoms and maternal and neonatal outcomes restrospective cohort; [ruptura d&#283;lohy v t&#283;hotenství a p&#345;i porodu rizikové faktory, p&#345;íznaky a perinatální výsledky retrospektivní analýza]
210. vaccination against influenza in pregnant women safety and effectiveness; [szczepienia przeciwko grypie u kobiet ciężarnych bezpieczeństwo i efektywność]
211. voluntary interruption of pregnancy not an insignificant act; [l'ivg un acte non anodin]
212. wish of pregnancy refusal of pregnancy ambivalence in family planning; [desir de grossesse refus de grossesse ou les embuches du desir dans le planning familial]

**3.4 Records excluded: Irrelevant records (n=983)**

1. ‘…a one stop shop in their own community’ medical abortion and the role of general practice
2. ‘a hope raised and then defeated’? the continuing harms of irish abortion law
3. “i did not plan to have a baby this is the outcome of our work” a qualitative study exploring unintended pregnancy among female sex workers
4. “nothing more or less than a discharged convict” the career of dr thomas millerchip of coventry, 18741912
5. …a one stop shop in their own community' medical abortion and the role of general practice
6. 10year evaluation of the use of medical abortion through telemedicine a retrospective cohort study
7. 201719 governmental decisions to allow home use of misoprostol for early medical abortion in the uk
8. 5 embryo manipulation and experimentation
9. a comparative study of manual vacuum aspiration with medical method of management for incomplete and missed abortion
10. a comparison of medical abortion (using mifepristone and gemeprost) with surgical vacuum aspiration efficacy and early medical sequelae
11. a comprehensive study of jaundice in pregnancy with emphasis on fetomaternal outcome
12. a depth study of a married woman; impact of induced abortions on fertility
13. a directtopatient telemedicine abortion service in australia retrospective analysis of the first 18 months
14. a kap study on mtp acceptors and their contraceptive practice
15. a maternal death from selfinduced medical abortion a call for action
16. a medical method of early pregnancy termination using tamoxifen and misoprostol
17. a medical record linkage analysis of abortion underreporting
18. a pilot study of acceptability of ru486 and ono 802 in a chinese population
19. a pilot study of the effect of methotrexate or combined oral contraceptive on bleeding patterns after induction of abortion with mifepristone and a prostaglandin pessary
20. a pilot study on the use of a 7day course of letrozole followed by misoprostol for the termination of early pregnancy up to 63 days
21. a pilotstudy of acceptability of ru486 and ono802 in a chinese population
22. a prospective economic evaluation comparing medical abortion (using mifepristone and gemeprost) and surgical vacuum aspiration
23. a prospective study of mifepristone and unlimited dosing of sublingual misoprostol for termination of secondtrimester pregnancy in uzbekistan and ukraine
24. a prospective study of spontaneous fetal losses after induced abortions
25. a randomised comparative study on sublingual versus vaginal administration of misoprostol for termination of pregnancy between 13 to 20 weeks
26. a randomised controlled trial of mifepristone in combination with misoprostol administered sublingually or vaginally for medical abortion up to 13 weeks of gestation
27. a randomised study of two doses of gemeprost in combination with mifepristone for induction of abortion in the second trimester of pregnancy
28. a randomized comparative study on vaginal administration of acetic acidmoistened versus dry misoprostol for midtrimester pregnancy termination
29. a randomized controlled study of sublingual vs oral misoprostol (400 mg) following mifepristone (200 mg) for medical abortion up to 63 days lmp in tunisia
30. a randomized trial of hospital vs home self administration of vaginal misoprostol for medical abortion
31. a randomized trial of mifepristone in combination with misoprostol administered sublingually or vaginally for medical abortion at 1320 weeks gestation
32. a retrospective cohort analysis of retained products of conception with vascularity after secondtrimester abortion incidence, outcomes, and ultrasonographic features leading to severe hemorrhage
33. a retrospective cohort analysis of retained products of conception with vascularity after secondtrimester abortion;incidence, outcomes in expectant management, and characteristic ultrasonographic features leading to future severe hemorrhage
34. a retrospective comparison between lamicel and gemeprost for cervical ripening before surgical interruption of firsttrimester pregnancy
35. a retrospective service evaluation of early medical abortion at home by a district general hospital during the covid19 pandemic 2020 to 2022
36. a safe and effective method of termination of midtrimester pregnancy
37. a shortscheme protocol of gemeprost for midtrimester termination of pregnancy with uterine scar
38. a stalled revolution? misoprostol and the pharmaceuticalization of reproductive health in francophone africa
39. a statistical analysis of women's reproductive health characteristics after ineffective reattempts of using art
40. a study of fertility and pregnancy outcomes in women after surgery for valvular heart disease
41. a study of incomplete abortion following medical method of abortion (mma)
42. a study of psychiatric morbidity during second trimester of pregnancy subsequent to abortion in the previous pregnancy
43. a study of the effects of folklore about the body on iud use by black american adolescents
44. a study of the prevalence of reproductive tract infections and its associated factors among married women of age 15 to 49 years in an adopted phc of adichunchanagiri institute of medical sciences, bg nagara, mandya
45. a successful twin pregnancy in a patient with hbeβthalassemia in western india
46. a thoroughly regressive law
47. ablebodied women killing disabled babieshow modern narratives on disability and abortion erase disabled people from the reproductive justice movement
48. abortamento espontâneo um estudo epidemiológico
49. abortion
50. abortion an attitude study of professional staff at ramathibodi hospital
51. abortion an attitude study of professional staff at ramathibodihospital
52. abortion and legal policy
53. abortion at gondar college hospital, ethiopia
54. abortion at the 1st trimester of pregnancy the risks of responsibility from jurisprudence and the study of 81 complaints
55. abortion attitudes in trinidad
56. abortion due to infection with chlamydia psittaci in a sheep farmer's wife
57. abortion education in canadian family medicine residency programs
58. abortion in brazil what do the official data say?
59. abortion in international human rights law missed opportunities in manuela v el salvador
60. abortion in minors
61. abortion in relation to fetal and maternal welfare
62. abortion in today's world
63. abortion incidence and services in the united states, 19951996
64. abortion induced with methotrexate and misoprostol a comparison of various protocols
65. abortion law in france
66. abortion law reform in india
67. abortion medical management between 1416 weeks' amenorrhea after french legislation deadline extension
68. abortion pill a boon or a curse?
69. abortion policies and practices in chile ambiguities and dilemmas
70. abortion policy implementation in ireland lessons from the community model of care
71. abortion practices in high school students in yamoussoukro, cote d'ivoire
72. abortion procedures in a tertiary care institution in india
73. abortion providers' experiences and views on selfmanaged medication abortion an exploratory study
74. abortion providers' resilience to antichoice tactics in the united states and canada
75. abortion related acute renal failure a study in dhaka medical college hospital
76. abortion reporting in the era of medical procedures why is it important?
77. abortion services and military medical facilities
78. abortion techniques in australia a history
79. abortion training at multiple sites an unexpected curriculum for teaching systemsbased practice
80. abortion, breast cancer, and informed consent
81. abortion, informed consent, and regulatory spillover
82. abortions in a hospital setting hidden realities in daressalaam, tanzania
83. aborto no brasil o que dizem os dados oficiais?
84. about analysis of and reflections on some abortion request statistic
85. acceptability of homeuse of misoprostol in medical abortion
86. acceptability of medical abortion in early pregnancy
87. accessibility, safety and outcomes in a united kingdom national health service nhs abortion clinic (walsall)
88. accessing abortion in a highly restrictive legal regime characteristics of women and pregnant people in malta selfmanaging their abortion through online telemedicine
89. accounting for time insights from a lifetable analysis of the efficacy of medical abortion
90. accuracy of anemia screening by pointofcare hemoglobin testing in patients seeking abortion
91. achievements of the figo initiative for the prevention of unsafe abortion and its consequences in southsoutheast asia
92. acquired uterine arteriovenous malformation management and treatment
93. acr appropriateness criteria® first trimester bleeding
94. acupuncture benefits to women with recurrent implantation failure a propensity scorematched cohort study
95. acute kidney injury in asia
96. acute renal failure following hexolinduced abortion
97. acute renal failure in blacks and indians in south africa comparison after 10 years
98. acute renal failure in obstetric patients treatment by hemodialysis
99. acute renal failure in obstetrics
100. acute renal failure in pregnancy
101. acute renal failure in pregnancy 1987
102. acute renal failure in pregnancy a decade of change
103. acute renal failure in pregnancy in a developing country twenty years of experience
104. acute uterine inversion following an induced abortion
105. acuterenalfailure in blacks and indians in southafrica comparison after 10 years
106. administration of depot medroxyprogesterone acetate on the day of mifepristone for medical abortion a pilot study
107. advanced abdominal pregnancy as a cause of symptomatic hemoperitoneum
108. advanced practice clinicians and medical abortion increasing access to care
109. advantages and disadvantages of medical abortion, according to brazilian residents in obstetrics and gynaecology
110. adverse pregnancy outcome and childhood malignancy with reference to paternal welding exposure
111. afrocaribbean children in local authority day care 0 3
112. age, parity, history of abortion and contraceptive choices affect the risk of repeat abortion
113. alteration of th1 and th2 cells by intracellular cytokine detection in patients with unexplained recurrent abortion before and after immunotherapy with the husband's mononuclear cells
114. ambulatory medical abortion performed in a family planning center
115. amniocentesis before 15 weeks gestation technical aspects and obstetric risks
116. an analysis of the cost of incomplete abortion to the public health sector in south africa 1994
117. an examination of loss to followup and potential bias in outcome ascertainment in a study of directtopatient telemedicine abortion in the united states
118. an experience with misoprostol for the induction of first trimester abortions in a secondary hospital in south africa
119. an overview of unsafe abortion in africa foreword
120. analysis of complications and management after selfadministration of medical termination of pregnancy pills
121. analysis of motivation of sterilised women wishing to be refertilised
122. analysis of pharmacodynamic components, targets and synergistic action mechanism of fuyuan shenghua granule for the treatment of medicalinduced incomplete abortion based on network pharmacology
123. analysis of severe adverse events related to the use of mifepristone as an abortifacient
124. angiographic transcatheter embolization in gynaecological practice
125. antifertility effect of continuous lowdosage oral progestogen therapy
126. antiprogesterone for medical abortion
127. application of multiplex quantitative fluorescent pcr with nonpolymorphic loci in prenatal diagnosis
128. are serum progesterone levels predictive of recurrent miscarriage in future pregnancies?
129. are us health professionals likely to prescribe mifepristone or methotrexate?
130. artificial insemination with fresh or frozen semen a comparative study
131. aspects of the significance of operatively corrected heartvalve failure on fertility
132. assessing postabortion care in health facilities in afghanistan a crosssectional study
133. assessing strength of preference for abortion method using 'willingness to pay' a useful research technique for measuring values
134. assessing survival time of outpatients with cervical cancer at the university of gondar referral hospital using the bayesian approach
135. assessment of completion of early medical abortion using a text questionnaire on mobile phones compared to a selfadministered paper questionnaire among women attending four clinics, cape town, south africa
136. assessment of efficacy and safety of medical treatment of nonviable first trimester pregnancy
137. assessment of rapid radioimmunoassay for urinary βhuman chorionic gonadotropin (βhcgria) in early pregnancy
138. assessment of the efficacy of different methods of second trimester abortion an initial experience in eastern nepal
139. association between the route of administration and efficacy of the misoprostol in the management of missed abortion a randomized clinical trial
140. association between ultrasonographic parameters of cesarean scar defect and outcome of early termination of pregnancy
141. attitudes to contraception in women seeking termination of pregnancy
142. attributes and perspectives of public providers related to provision of medical abortion at public health facilities in vietnam a crosssectional study in three provinces
143. autoantibodies in normal and abnormal pregnancy
144. azzolino v dingfelder
145. bacterial sacroiliitis and gluteal abscess after dilation and curettage for incomplete abortion
146. bacteroidaceae bacteremia effect of age and focus of infection upon clinical course
147. barriers and enablers to nurse practitioner provision of medication abortion in canada results from a national survey
148. bioartificial liver from cultured human foetal hepatocytes feasibility and prospects
149. birthcontrol and social group contraception, accidental pregnancies and abortion
150. bleeding after dilatation and curettage the efficacy of transcatheter uterine artery embolisation
151. blood loss with mifepristonemisoprostol abortion measures from a trial in china, cuba and india
152. body failure medical views of women, 19001950
153. brazil investigates drugs possible link with birthdefects
154. british abortion act
155. buxue yimu pills improve angiogenesis and blood flow in experimental zebrafish and rat models
156. california may become first state to mandate insurance coverage for contraception
157. can midlevel healthcare providers administer early medical abortion as safely and effectively as doctors? a randomised controlled equivalence trial in nepal
158. can the outcome of the next pregnancy be predicted at the time of induced abortion?
159. can women determine the success of early medical termination of pregnancy themselves?
160. canadian contraception consensus (part 1 of 4)
161. cannulation pitfalls in ecmo
162. cardiac surgical experience in northern nigeria
163. cardiovascular disease and associated comorbid conditions as determinants of adverse perinatal outcomes in pregnancy an analysis of the results of the register of pregnant bereg
164. catastrophic antiphospholipid syndrome
165. causes of acute renal failure in nishtar hospital multan
166. cervical ectopic pregnancy
167. cervical internal os cerclage description of a new technique and comparison with shirodkar operation
168. cervical twin heterotopic pregnancy overview of ectopic pregnancies and scanning detection algorithm
169. cesarean scar ectopic pregnancy the lurking danger in post cesarean failed medical abortion
170. cfilm a new local contraceptive
171. change in abortion services after implementation of a restrictive law in texas
172. changes in abortion service provision in bihar and jharkhand states, india between 2004 and 2013
173. changing trends in maternity care in sri lanka
174. characterisation of the molecular basis underlying chlamydial subversion of focal adhesion signaling
175. characteristics of maternal cardiac disease and pregnancy outcomes results from a 4year observational cohort survey in central vietnam
176. characteristics of medical termination of pregnancy acceptors in pondicherry state, 19721976
177. characteristics of women having abortion in china
178. chemical fingerprint analysis and ultraperformance liquid chromatography quadrupole timeofflight mass spectrometrybased metabolomics study of the protective effect of buxue yimu granule in medicalinduced incomplete abortion rats
179. choice at any cost
180. choice of analgesia or anesthesia for pain relief in suction curettage
181. choosing abortion teens who make the decision without parental involvement
182. chorionic villi sampling cytogenetic and clinical findings in 500 pregnancies
183. chronic bone marrow failure due to persistent b19 parvovirus infection
184. chronic myeloid leukemia comparison of survival between pregnant and nonpregnant women
185. clandestine abortion in port harcourt users' profile and motivation
186. client perspectives on choice of abortion method in england and wales
187. clinical analysis of ectopic pregnancies in a tertiary care centre in southern india a sixyear retrospective study
188. clinical analysis of secondtrimester pregnancy termination after previous caesarean delivery in 51 patients with placenta previa and placenta accreta spectrum a retrospective study
189. clinical and morphological differences in nondeveloping pregnancy and spontaneous abortion in early pregnancy
190. clinical approach and surgical strategy for spinal disorders in pregnant women
191. clinical course of hepatitis b virus infection during pregnancy
192. clinical diagnosis of completeness of medical abortion by nurses a reliability study in mozambique
193. clinical effects of myotonic dystrophy on pregnancy and the neonate
194. clinical experience with intramuscular norethisterone oenanthate as a contraceptive
195. clinical indication of medical ultrasound as a diagnostic tool among the outdoor patients of a selected hospital in bangladesh
196. clinical manifestations of pregnancy in patients with takayasu arteritis experience from a single tertiary center
197. clinical pattern of gynecological/early pregnancy complaints and the outcome of pelvic sonography in a private diagnostic center in ilorin
198. clinical significance of sub chorionic and retroplacental hematomas detected in the first trimester of pregnancy
199. clinical, surgical, and histopathologic outcomes following failed medical abortion
200. clinicianled transformation of sexual and reproductive healthcare in ni postdecriminalisation of abortion
201. clinton reform proposal faces congressional test on covered services, cost
202. cognitive bias in the management of a critically ill 29yearold patient
203. college women's experience with emergency contraception
204. commentary access to medicalassisted reproduction and pgd in italian law a deadly blow to an illiberal statute? commentary to the european court on human rights's decision costa and pavan v italy
205. comparative study of transdermal nitroglycerine patch and oral nifedipine in preterm labour
206. comparing pregnancy outcomes between symptomatic and asymptomatic covid19 positive unvaccinated women multicenter study in saudi arabia
207. comparison between the effectiveness of combination of letrozole with misoprostol and tamoxifen with misoprostol in medical termination of first trimester missed miscarriage
208. comparison of adolescent and adult experiences with norplant levonorgestrel contraceptive implants
209. comparison of clinical outcomes of vitrifiedthawed embryo transfer and fresh embryos transfer
210. comparison of intraamniotic prostaglandin f2αand hypertonic saline for induction of secondtrimester abortion
211. comparison of medical abortion with surgical vacuum aspiration women's preferences and acceptability of treatment
212. comparison of obstetric emergency clinical readiness a crosssectional analysis of hospitals in amhara, ethiopia
213. comparison of outcomes before and after ohio's law mandating use of the fdaapproved protocol for medication abortion a retrospective cohort study
214. comparison of rates of adverse events in adolescent and adult women undergoing medical abortion population register based study
215. comparison of the effects of voluntary termination of pregnancy and uterine evacuation for medical reasons on female sexual function
216. comparison of two lowsensitivity urine pregnancy tests for confirming the success of early medical abortion
217. compliance with new ban means no mention of abortion
218. complicated illegal induced abortions at a tertiary health institution in nigeria
219. complications during pregnancy and delivery in women with untreated rectovaginal deep infiltrating endometriosis
220. complications of second trimester induction for abortion or fetal demise for patients with and without prior cesarean delivery
221. complications related to induced abortion a combined retrospective and longitudinal followup study
222. comprehensive abortion care evidence of improvements in hospitallevel indicators in tigray, ethiopia
223. comprehensive genetic analysis of pregnancy loss by chromosomal microarrays outcomes, benefits, and challenges
224. concurrent copper t insertion with medical termination of pregnancy in women with previous caesarean section delivery
225. concurrent coppert insertion with medical termination of pregnancy in women with previous cesareansection delivery
226. conflicting recommendations between the french national authority for health (has) and clinical practice guidelines (cngof); focus on 200 late medical abortions, conducted outside marketing authorizations
227. congenital heart disease and acquired valvular lesions in pregnancy
228. conscientious objection to sexual and reproductive health services international human rights standards and european law and practice
229. conservative management of uterine necrosis following bilateral uterine vessels ligation and blynch suture long term follow up
230. constraints and optimization of the laser microwelding process of thin metal foils
231. construction and validation of a spatial database of providers of transgender hormone therapy in the us
232. continuation of pregnancy after medical advice in patients with desired abortion
233. continued clinical experience with an increasing dosage regimen of clomiphene citrate administration
234. contours of development
235. contraception failure in practice
236. contraception for the teenager
237. contraception from accessibility to efficiency
238. contraception utilization in black women via a reproductive justice lens
239. contraceptive behavior of jerusalem women seeking pregnancy counseling, 19801989
240. contraceptive coverage after medical termination of pregnancy
241. contraceptive discontinuation among white, black, and hispanic adolescents
242. contraceptive failure rates new estimates from the 1995 national survey of family growth
243. contraceptive practice of abortion patients
244. contraceptive practices of women attending for termination of pregnancy a study from south australia
245. contraceptive problems unique to the united states
246. contraceptive risktaking and contraceptive failure among users of female barrier methods
247. contraceptive use among women seeking repeat abortion in addis ababa, ethiopia
248. controversias en el tratamiento del aborto incompleto ameu versus tratamiento médico con misoprostol
249. controversy surrounds use of test for open spina bifida
250. correction management of septic chemical abortion with renal failure (n engl j med (1975) 292 (722725) (101056/nejm197504032921404))
251. corrections simultaneous compared with interval medical abortion regimens where home use is restricted (obstetrics and gynecology (2018) 131 (63541) doi 101097/aog0000000000002536)
252. cost effective & scalable community models for safe abortion in low resource countries in south asia
253. costeffectiveness of alternative strategies for firsttrimester abortion in mexico and nigeria
254. costeffectiveness of emergency contraception options over 1 year
255. costeffectiveness of levonorgestrel subdermal implants comparison with other contraceptive methods available in the united states
256. costeffectiveness of levonorgestrel subdermal implants comparison with other contraceptive methods available in the unitedstates
257. costeffectiveness of managing abortions manual vacuum aspiration (mva) compared to evacuation by curettage in tanzania
258. could american women use mifepristonemisoprostol pills safely with less medical supervision?
259. counseling women about childbearing and childrearing risks
260. criminality
261. crisis pregnancy centers in the united states lack of adherence to medical and ethical practice standards; a joint position statement of the society for adolescent health and medicine and the north american society for pediatric and adolescent gynecology
262. crisis pregnancy centers in the us lack of adherence to medical and ethical practice standards a joint position statement of the society for adolescent health and medicine and the north american society for pediatric and adolescent gynecology
263. critical appraisal of contraceptive research
264. cuban health system call it superior?
265. curettage and asherman's syndromelessons to (re) learn?
266. current state and future of ivf
267. cushing's syndrome during pregnancy secondary to adrenal adenoma metyrapone treatment and laparoscopic adrenalectomy
268. cutting unsafe abortions
269. cytogenetic studies of couples with reproductive failure in alexandria, egypt
270. cytomegalovirus and the expression of immunological markers in reproductive failure
271. dealing with the barriers to access to abortion
272. decentralising medical abortion services in victoria, australia
273. deciphering the voices in abortion care the woman's voice matters
274. decision making by single women seeking abortion
275. decreasing the need for abortion challenges and constraints
276. deformidad del principio de autonomía para sustentar actos éticamente ilícitos
277. delivering on the promise ippf's global action to safe abortion services
278. dengue fever during pregnancy maternal and fetal complications
279. dermatoglyphics associated with fetal wastage
280. design of a syringe extension device (chloe sed) for lowresource settings in subsaharan africa a circular economy approach
281. design of a syringe extension device (chloe sed®) for lowresource settings in subsaharan africa a circular economy approach
282. design options and methodological fallacies in the studies of reproductive failures
283. determinants and outcome of safe second trimester medical abortion at jimma university medical center, southwest ethiopia
284. determinants of abortion among women admitted to hospitals in fortaleza, north eastern brazil
285. determinants of low birthweight methodological assessment and metaanalysis
286. determination of pregnant women knowledge toward risk factors of vitamin d deficiency and measuring level during pregnancy in al nasiriyah city
287. development and evaluation of the abortion attributes questionnaire
288. development and pilot testing of the 2019 canadian abortion provider survey
289. developmental and reproductive risks of radiological procedures utilizing ionizing radiation during pregnancy
290. diagnosis of aneuploidy in archival, paraffinembedded pregnancyloss tissues by comparative genomic hybridization
291. diagnosis of aneuploidy in arrival, paraffinembedded pregnancyloss tissues by comparative genomic hybridization
292. diagnostic value of prenatal mr imaging in the detection of brain malformations in fetuses before the 26th week of gestational age
293. diaphragm method contraceptors implications for service organization and delivery
294. different types of serologic reactivity to bedsonia (psittacosis group) antigen in various hosts discussion of some related problems
295. direct effects of mifepristone on mice embryogenesis an in vitro evaluation by singleembryo rna sequencing analysis
296. discussing the best way of followup
297. disfunção tiroideia em grávidas de alto e baixo risco numa região com défice de iodo (beira interior)
298. district court finds proposed iowa women's health facility does not need state approval
299. does congenital heart disease severely jeopardise family life and pregnancies? obstetrical history of women with congenital heart disease in a single tertiary centre
300. does sublingual misoprostol reduce pain and facilitate iud insertion in women with no previous vaginal delivery? a randomized controlled trial
301. does supportive legislation guarantee access to pregnancy termination and postabortion care services? findings from a facility census in central province, zambia
302. doppler velocimetry of the uterine arteries an early screening test for miscarriage
303. drug interactions between oral contraceptives and antibiotics
304. early medical abortion first experiences
305. early pregnancy termination with a simplified mifepristone medical abortion outpatient regimen
306. early pregnancy termination with mifepristone and misoprostol in norway
307. early pregnancy termination with mifepristone and misoprostol in the united states
308. early pregnancy termination with oral mifepristone and vaginal misoprostol
309. early surface ablation on aborted lasik flaps
310. early termination of pregnancy with mifepristone (ru 486) and the orally active prostaglandin misoprostol
311. early versus delayed insertion of nexplanon at medical abortiona randomized controlled equivalence trial
312. economic cost of pregnancy care attributable to the failure of mexico's teenage pregnancy prevention policy
313. ectopic pregnancy in the era of medical abortion are we ready for it? spectrum of sonographic findings and our experience in a tertiary care service hospital of india
314. effect of abortion, in vitro fertilization, and other causes of prenatal death on life expectancy in the united states from 1925 to 2005
315. effect of endometrial stimulation on pregnancy outcome of in vitro fertilization patients
316. effect of folic acid supplementation on diminished ovarian reserve study protocol of a singlecentre, openlabel, randomised, placebocontrolled clinical trial
317. effect of immediate compared with delayed insertion of etonogestrel implants on medical abortion efficacy and repeat pregnancy
318. effect of immediate compared with delayed insertion of etonogestrel implants on medical abortion efficacy and repeat pregnancy a randomized controlled trial
319. effect of letrozole on uterine artery doppler flow indices prior to firsttrimester termination of pregnancy a randomized controlled trial
320. effect of pregnancy in arrhythmogenic right ventricular cardiomyopathy
321. effect of wujia shenghua capsule on uterine bleeding following medicallyinduced incomplete abortion in rats during early pregnancy
322. effective, lowcost regimens for medical termination of pregnancy at all gestations
323. effectiveness and safety of sublingual misoprostol in medical treatment of the 1st trimester miscarriage experience of offlabel use in korea
324. effectiveness of genetic consultation and genetic prevention
325. effectiveness of jianpiantai formula for the pregnancy outcome of in vitro fertilization and embryo transfer in infertile women protocol of a randomized controlled trial
326. effectiveness of the diaphragm, used continuously, without spermicide
327. effectiveness of the figo protocol for medical management of firsttrimester abortion
328. effects of leonurine hydrochloride on medically induced incomplete abortion in early pregnancy rats
329. effects of making emergency contraception available without a physician's prescription a populationbased study
330. effects of mifepristone on fibronectin in human villus and deciduas
331. effects of taking estrogen and progestogen after medical abortion on reducing vaginal hemorrhage time a randomizedcontrolled trial
332. efficacy and safety of the medical method in the voluntary interruption pregnancy over 8 yearsexperience of a portuguese medical centre
333. eficacia y seguridad del taponamiento uterino para control de hemorragia y disminución de histerectomía obstétrica cohorte histórica en nuevo león, méxico, 2013
334. eisenmenger syndrome factors relating to deterioration and death
335. elective abortion as a primary health service in rural india experience with manual vacuum aspiration
336. emergency contraception plan b
337. emergency contraceptive pills a simple proposal to reduce unintended pregnancies
338. employee benefits no abortion benefits, no contracts
339. encefalocele occipital gigante sin complicaciones neonatales inmediatas
340. enduring politics the culture of obstacles in legislating for assisted reproduction technologies in ireland
341. epidemiology of spontaneous pregnancy loss in kazakhstan a national populationbased cohort analysis during 20142019 using the national electronic healthcare system
342. errors in after medical abortion recovery period and their overcoming through the training of ob&gyn
343. estimates of demand for abortion among soviet immigrants in israel
344. estimates of pregnancies averted through california's family planning waiver program in 2002
345. estimating the costs for the treatment of abortion complications in two public referral hospitals a crosssectional study in ouagadougou, burkina faso
346. estimation of the adolescent pregnancy rate in thailand 20082013 an application of capturerecapture method
347. estudios citogenéticos en pacientes con fallas reproductivas pinar del río, 20152020
348. ethical issues in prenatal diagnosis
349. ethics and medical decisionmaking
350. ethics and reproductive health a principled approach
351. etiologies and subsequent reproductive performance of 100 couples with recurrent abortion
352. etonogestrel implant at the time of mifepristone for medical abortion
353. evaluating wellwoman clinics
354. evaluating women’s acceptability of treatment of incomplete second trimester abortion using misoprostol provided by midwives compared with physicians a mixed methods study
355. evaluation of a lower dose schedule of intramuscular 15(s)15methyl prostaglandin f2 alpha for induction of early midtrimester abortion
356. evaluation of a network of medical abortion providers in two districts of maharashtra, india
357. evaluation of abortion services and patient characteristics in a mexico city public hospital after legalization
358. evaluation of anticoagulant use during pregnancy in mothers with antiphospholipid syndrome an observational crosssectional study
359. evaluation of competitive elisa for detection of antibodies to rift valley fever virus in cattle and sheep sera
360. evaluation of different doses of femoston therapy for incomplete abortion a prospective observational trial
361. evaluation of the incidence and outcome of gestational diabetes mellitus using the current international consensus guidelines for diagnosing hyperglycaemia in pregnancy
362. evidencebased practicefact or fiction?
363. evolución cardiológica postnatal y factores asociados a la agenesia de ductus venoso de diagnóstico prenatal
364. exhaustion in myeloid lineage and very early defect in hspc pool an embryonic origin of fanconi haematological disorders
365. experience and acceptability of emergency hormonal contraception
366. experience of clandestine use of medical abortion among university students in chile a qualitative study
367. experience with midtrimester abortion
368. experiences of women living in hungary seeking a medical abortion online
369. exploring the costs and economic consequences of unsafe abortion in mexico city before legalisation
370. exposure to radiation therapy is associated with female reproductive health among childhood cancer survivors a metaanalysis study
371. factors affecting the success of repeated misoprostol course for the treatment of missed abortion
372. factors associated with cesarean operations of gestational diabetic mellitus and diabetes complications
373. factors associated with followup rates after medical abortion in adolescents at cook county hospital
374. factors associated with management outcome of incomplete abortion in yirgalem general hospital, sidama zone, southern ethiopia
375. factors associated with severe complications in unsafe abortion
376. factors hindering access to abortion services
377. factors influencing the delivery of abortion services in ontario a descriptive study
378. failure of sterilization after clip placement
379. failure to achieve the association of professors in gynecology and obstetrics objectives for abortion in thirdyear medical student curriculum
380. family planning clinics share blame for men's failure to use services
381. family planning in europe
382. farmacologico abortion by means of mifepristone and misoprostol
383. fatal hemorrhage from legal abortion in the united states
384. fatal necrotizing fasciitis in illegal abortion and the negligence tort
385. feasibility of a hospital outpatient day procedure for medication abortion at 1318 weeks gestation findings from nepal()
386. feasibility of a selfperformed urinary test for the followup on medical abortion the betina study
387. female rehabilitation after medical abortion
388. fertility and pregnancy outcomes following uterine devascularization for severe postpartum haemorrhage
389. fertility and pregnancy outcomes in females with dyskeratosis congenita
390. fertility following ectopic pregnancy
391. fertility trends in singapore
392. fetal cystic hygroma cause and natural history
393. fetal death ratios in a prospective study compared to state fetal death certificate reporting
394. fetal erythrocytes in maternal circulation after spontaneous abortion
395. filum terminale needle placement during caudal epidural steroid injection
396. first pregnancy abortion as an infectious complications risk factor
397. first trimester medical abortion practice in north east india
398. first trimester medical termination of pregnancy the nottingham experience
399. firsttrimester spontaneous abortions and the incidence of human immunodeficiency virus seropositivity
400. five years experience of haemodialysis at the lagos university teaching hospitalnovember 1981 to november 1986
401. followup after early medical abortion comparing clinical assessment with selfassessment in a rural hospital in northern norway
402. followup rates and contraceptive choices after medical abortion in adolescents at cook county hospital
403. forensic problems in bovine obstetrics and gynecology
404. formulation and noncontraceptive uses of the new, lowdose oral contraceptive
405. frequency and risk factors for repeat abortions after surgical compared with medical termination of pregnancy
406. fulminant postpartum cerebral vasoconstriction syndrome
407. future uncertain as congressional drive to block title x gag rule fails
408. garrison v medical center of delaware, inc, 12 december 1989
409. gastrojejunostomy as successful surgical treatment of acute duodenitis/proximal jejunitis in a mare
410. general practitioner knowledge and practice in relation to unintended pregnancy in the grampians region of victoria, australia
411. genetic findings in early miscarriage analysis by chromosomal microarray and whole exome sequencing
412. gestational trophoblastic disease treatment results at the brewer trophoblastic disease center
413. has mifepristone medical abortion expanded abortion access in new mexico? a survey of obgyn and family medicine physicians
414. heads up! potential legal obstacles to medical abortion
415. health benefits of legal abortion an analysis
416. healthcare students' knowledge and opinions about the argentinean abortion law
417. history of medicine volume 4
418. hodgkin’s disease in pregnancy
419. home selfadministration of vaginal misoprostol for medical abortion at 5063 days compared with gestation of below 50 days
420. homocysteine, folic acid and vitamin b12 concentration in patients with recurrent miscarriages
421. honour and interests medical ethics in britain, and the work of the british medical association's central ethical committee, 19021939
422. how antiabortion legislation would put doctors in a bind
423. how family planning can save lives in africa
424. how safe motherhood in india is
425. how the coronavirus disease 2019 pandemic is impacting sexual and reproductive health and rights and response results from a global survey of providers, researchers, and policymakers
426. human assisted reproduction
427. hummel v reiss
428. hydatidiform moles among patients with incomplete abortion in mwanza city, north western tanzania
429. hypoplastic left heart syndrome 19 years of prenatal diagnosis
430. hysteroscopic diagnosis of omentum incarceration subsequent to an iatrogenic uterine perforation
431. hysteroscopic findings after missed abortion
432. hysteroscopic removal of intrauterine contraceptive devices with missing threads
433. i did not plan to have a baby this is the outcome of our work a qualitative study exploring unintended pregnancy among female sex workers
434. i will never wish this pain to even my worst enemy lived experiences of pain associated with manual vacuum aspiration during postabortion care in kenya
435. identifying national availability of abortion care and distance from major us cities systematic online search
436. if you were to have another abortion, would you choose the same method? a study on 1032 patients’ level of satisfaction
437. illegal abortion with misoprostol in guadeloupe
438. imaging and imagining the fetus
439. immediate postabortion intrauterine contraception in nulliparous adolescents
440. immediate versus delayed insertion of an etonogestrel releasing implant at medical abortion a randomized controlled equivalence trial
441. immediate versus delayed insertion of an etonogestrel releasing implant at medical abortiona randomized controlled equivalence trial
442. impact of prolonged use of adjuvant tocolytics after cervical cerclage on late abortion and premature delivery
443. impact of the introduction of new medical methods on therapeutic abortions at the royal infirmary of edinburgh
444. impact of the supreme court decisions on the performance of abortions in the united states
445. impacto del estrés laboral en el anestesiólogo
446. impacts of delivery mode and maternal factors on neonatal oral microbiota
447. implantable cardioverterdefibrillators and pregnancy a safe combination?
448. implications for gynecological services of new medical methods of therapeutic abortion
449. improving safe postabortion care practices a study on interventions implemented by ipas pakistan
450. incidence of diandric triploidy (partial mole) in misscarriages
451. incidence of induced abortion determined by the randomised response technique
452. increased transient tachypnea of the newborn in infants of asthmatic mothers
453. increasing women's choices in medical abortion a study of misoprostol 400 μg swallowed immediately or held sublingually following 200 mg mifepristone
454. india's missing daughters an ominous sign for democracy
455. induced abortion in the first trimester of pregnancy and repeat pregnancy in adolescents
456. induced abortion physician training and practice patterns
457. induced abortions in khamano block of fatehgarh sahib district, punjab
458. inducedabortion among unmarried women in sichuan province, china
459. inducedabortion in maternal and child health centers in a general familyplanning program in cap bon, tunisia
460. induction of abortion in the second trimester of pregnancy by intramuscular 15methyl prostaglandin f 2 alpha (prostin 15m)
461. induction of abortion with mifepristone and misoprostol in earlypregnancy
462. inequalities in access to and quality of abortion services in mexico can tasksharing be an opportunity to increase legal and safe abortion care?
463. infectious agents identified in aborted swine fetuses in a highdensity breeding area a threeyear study
464. infertility today the management of female medical causes
465. injectable contraception
466. instructiononly versus demonstration of a low sensitivity pregnancy test for selfassessment of medical abortion in south africa; a multicentre noninferiority randomised controlled trial
467. integrating medical abortion into safe abortion services experience from three pilot sites in south africa
468. integrating mobile phones into medical abortion provision intervention development, use, and lessons learned from a randomized controlled trial
469. integrative transcriptomics and proteomics analyses to reveal the therapeutic effect and mechanism of buxue yimu pills in medicalinduced incomplete abortion rats
470. interrupção da gestação por opção na póvoa de varzim análise e perspectivas
471. intraabdominal hemorrhage induced by medical abortion in midtrimester pregnancy an unusual presentation of placenta percreta
472. intraamniotic injection of hypertonic solution as a method for termination of pregnancy
473. intrauterine administration of hcg immediately after oocyte retrieval and the outcome of icsi a randomized controlled trial
474. intrauterine contraception after medical abortion factors affecting success of early insertion
475. intravaginal misoprostol for medical evacuation of first trimester missed abortion
476. introducing medical abortion in turkey perceptions of physicians
477. introducing medical abortion in turkey perspectives of nurses/midwives
478. introducing medical abortion in turkey perspectives of physicians
479. introduction menstrual regulation the method and the issues
480. invasive management of proximal ureteral calculi during pregnancy
481. investigating the reasons of preterm labor among visitors of shariati hospital in bandar abbas during 2012 and 2013
482. is it safe to provide abortion pills over the counter? a study on outcome following selfmedication with abortion pills
483. is selfassessment of medical abortion using a lowsensitivity pregnancy test combined with a checklist and phone text messages feasible in south african primary healthcare settings? a randomized trial
484. is selfmedication with over the counter abortion pills really safe? an experience at a tertiary care centre
485. is sexselective abortion against the law?
486. is therapeutic abortion preventable?
487. is ward evacuation for uncomplicated incomplete abortion under systemic analgesia safe and effective a randomized clinicaltrial
488. it’s a small bit of advice, but actually on the day, made such a difference… perceptions of quality in abortion care in england and wales
489. judgment, 28 january 1987
490. kisspeptin a potential factor for unexplained infertility and impaired embryo implantation
491. knowledge and use of secondary contraception among patients requesting termination of pregnancy
492. lamicel is more effective in preparing the cervix for midtrimester termination of pregnancy than laminaria japonicum
493. laparoscopic sterilisation opinion and practice among gynaecologists in scotland
494. laparoscopic tubal sterilization coincident with therapeutic abortion by suction curettage
495. laparoscopic tubalsterilization using yoons rings the technique and the psychological effects
496. large variation in clinical regimens used to induce medical abortion in denmark
497. late midtrimester medical pregnancy terminations 3 different procedures with prostaglandinf2alpha and laminaria tents
498. lateterm abortion and medical necessity a failure of science
499. latin american hospitals improve postabortion care maternal health
500. legal duties to respect abortion choices
501. legal limits relaxed time to look at other barriers faced by women seeking termination of pregnancy for fetal anomalies
502. leptospirosis seropositivity and its serovars among cattle in northeastern malaysia
503. lessons learned from introducing dilation and evacuation in tigray region of ethiopia
504. level of realization of reproductive potential in women of different age
505. life and death issues in bioethics abortion, persistent vegetative state, and the definition of death
506. lifethreatening complications of mtp/abortion
507. longterm outcome of lupus nephritis a single center study
508. lownganonglevine syndrome in a 3monthold infant with isolated left ventricular noncompaction
509. malaria in pregnancy
510. malaria in pregnancy clinicalfeatures and outcome of treatment
511. male experiences of unintended pregnancy characteristics and prevalence
512. management of gestational trophoblastic tumours a fiveyear clinical experience
513. management of incomplete abortion with manual vacuum aspiration in comparison to sharp metallic curette in an ethiopian setting
514. management of incomplete and missed spontaneous abortions a cohort study of trends in calgary emergency departments
515. management of incomplete spontaneous abortion with suction curettage in the pediatric emergency department
516. management of pregnancy and delivery in women with transposition of the great arteries after atrial switch operation a 16year singlecenter experience
517. management of septic abortion
518. mandatory parental involvement/judicial bypass laws do they promote adolescents' health?
519. manual vacuum aspiration a safe and costeffective substitute of electric vacuum aspiration for the surgical management of early pregnancy loss
520. manual vacuum aspiration saves lives
521. manual vacuum aspiration technique draws interest
522. massachusetts medical society
523. maternal activity in relation to birth size in rural india the pune maternal nutrition study
524. maternal and fetal outcomes of subsequent pregnancies in women with peripartum cardiomyopathy
525. maternal and fetal outcomes with aortic dissection in pregnant patients with marfan syndrome
526. maternal and fetal prognosis of subsequent pregnancy in black african women with peripartum cardiomyopathy
527. maternal and infant followup after total cervical occlusion
528. maternal and perinatal outcome of maternal obesity at rscm in 20142019
529. maternal fontan procedure is a predictor of a smallforgestationalage neonate a 10year retrospective study
530. maternal heart disease and pregnancy outcome a single
531. maternal level of pregnancyassociated plasma protein a as a predictor of pregnancy failure in threatened abortion
532. maternal mortality and morbidity associated with clandestine abortions
533. maternal mortality at the university of benin teaching hospital benin city, nigeria
534. maternal mortality at the university of nigeria teaching hospital, enugu a 10year survey
535. maternal mortality in a semiurban nigerian community
536. maternal mortality in hospitals in zululand, july 1993 june 1994
537. matsubarayano suture a simple uterine compression suture for postpartum hemorrhage during cesarean section
538. medical abortion
539. medical abortion as an alternative to vacuum aspiration first experiences with the 'abortion pill' in the netherlands
540. medical abortion at home and at hospital a trial of efficacy and acceptability
541. medical abortion clinical aspects
542. medical abortion complications an epidemiologic study at a midmissouri clinic
543. medical abortion in rural tamil nadu, south india a quiet transformation
544. medical abortion in second trimester missed abortion pregnancies
545. medical abortion in women of ≤56 days amenorrhoea; a comparison between gemeprost (a pge1 analogue) alone and mifepristone and gemeprost
546. medical abortion in women of less than or equal to 56 days amenorrhoea a comparison between gemeprost (a pge1 analogue) alone and mifepristone and gemeprost
547. medical abortion in women of lessthanorequalto56 days amenorrhea a comparison between gemeprost (a pge(1) analog) alone and mifepristone and gemeprost
548. medical abortion outcomes after a seconddose of misoprostol is the presence of cardiac motion on followup ultrasonography predictive of failure?
549. medical abortion outcomes and repeat pregnancy following quickstart of contraceptive implants and depotmedroxyprogesterone acetate
550. medical abortion outcomes following quickstart of contraceptive implants and depotmedroxyprogesterone acetate
551. medical abortion provided by nursemidwives or physicians in a high resource setting a costeffectiveness analysis
552. medical abortion public health and private lives
553. medical abortion self use in kenya results from a process evaluation of women's experiences
554. medical abortion what does the research tell us?
555. medical abortion with home administration of misoprostol up to 63 days gestation
556. medical abortion with mifepristone and home administration of misoprostol up to 63 days' gestation
557. medical abortion with mifepristone and misoprostol a clinical trial in taiwanese women
558. medical abortionlet us give quality care! making medical abortion safer and more effective in low resource population a comparative study from private hospital, nagpur india
559. medical and social aspects of adolescent pregnancies i adolescents applying for termination of an illegitimate pregnancy
560. medical and social problems and attitudes to optional abortions in bulgaria
561. medical developments and religious belief with special reference to europe in the 18th and 19th centuries
562. medical evacuation of 1st trimester (12 weeks gestation) incomplete abortion and missed abortion
563. medical evacuation of first trimester (twelve weeks gestation) incomplete abortion and missed abortion
564. medical management of firsttrimester abortion
565. medical management of missed abortion a randomized clinical trial
566. medical referral for abortion and freedom of conscience in australian law
567. medical responsibility in the practice of voluntary first trimester terminations of pregnancy preventive measures drawn from jurisprudence and from the study of 50 files of legal action or threatened legal action
568. medical student awareness of sexual health is poor
569. medical students and controversial ethical issues results from the multicenter study sbrame
570. medical students' intentions to seek abortion training and to provide abortion services in future practice
571. medical termination of pregnancy a study of acceptor characteristics
572. medical termination of pregnancy and concurrent contraceptive adoption in a tertiary referral hospital in delhi
573. medical termination of pregnancy in the second trimester
574. medical termination of pregnancy with mifepristone initial experience at the sheba medical center
575. medical treatment for missed abortion difference in gestational age based on menstrual dating and on ultrasound criteria, and the correlation to failure
576. medicalmanagement of miscarriage nonsurgical uterine evacuation of incomplete and inevitable spontaneousabortion (vol 306, pg 894, 1993)
577. meeting women's need for a flexible abortion service retrospective study of a specialist daycare unit
578. menstrual induction with mifepristone and misoprostol
579. menstrual regulation in family planning services
580. menstrual regulation using medication is acceptable and feasible in bangladesh
581. methotrexate and misoprostol teratogenicity further expansion of the clinical manifestations
582. methotrexate compared with mercaptopurine for early induced abortion
583. microbiology relevant to recurrent miscarriage
584. midgestational abortion for medical or genetic indications
585. midtrimester abortion by dilatation and evacuation a safe and practical alternative
586. midtrimester abortion with intramuscular injection of 15methylprostaglandin f2alpha
587. midtrimester pregnancy termination in teenage women
588. midwives, nurses and doctors performing mva in vietnam and south africa and early medical abortion in nepal evidence from randomized, controlled trials
589. mifepristone (ru 486) — an abortifacient to prevent abortion?
590. mifepristone (ru 486) compared with highdose estrogen and progestogen for emergency postcoital contraception
591. mifepristone (ru486) compared with highdose estrogen and progestogen for emergency postcoital contraception
592. mifepristone abortion in minors
593. mifepristone followed by home administration of buccal misoprostol for medical abortion up to 70 days of amenorrhoea in a general practice in curaçao
594. mifepristone in mid trimester termination of pregnancyvalue for money?
595. mifepristone in midtrimester termination of pregnancy value for money
596. mifepristonemisoprostol abortion a trial in rural and urban maharashtra, india
597. minors' behavioral responses to parental involvement laws delaying abortion until age 18
598. misconceptions monstrosity and the politics of interpretation in american culture from the antinomian controversy to biotechnology
599. misdiagnosis of ectopic pregnancy importance of ultrasonography prior to early termination of pregnancy
600. mish publishes new framework for fearbased, abstinenceonly education
601. misoprostol (cytotec8)exposure pregnancy a french coijaborative study
602. misoprostol and pregnancy risk of malformations
603. misoprostol for termination of second trimester pregnancy in a scarred uterus
604. misoprostol use as a method of medical abortion
605. misoprostol use in obstetrics and gynecology in brazil, jamaica, and the united states
606. misoprostolinduced fever and unnecessary antibiotic prescribing a retrospective study
607. modern indications for abortion
608. morbidity of therapeutic abortion in auckland
609. morbidity that is associated with curettage for the management of spontaneous and induced abortion in women who are infected with hiv
610. mortality associated with hypertonic saline abortion
611. motives for selfinduced abortion among women in alexandria an exploratory study
612. must a catholic hospital inform a rape victim of the availability of the morningafter pill
613. must a catholic hospital inform a rape victim of the availability of the 'morningafter pill'?
614. myometritis with pelvic septic vein thrombophlebitis secondary to fusobacterium necrophorum sepsis
615. new evidence on induced abortion in tehran, iran rates, causes, and changes
616. new frontiers in abortion care
617. nondeveloping pregnancy histological and immunohistochemical markers of endocrine disorders in endometrial scrapes
618. nonphysician clinicians can safely provide first trimester medical abortion
619. north florida women's health services v state
620. not every subseptate uterus requires surgical correction to reduce poor reproductive outcome
621. nurse versus physicianprovision of early medical abortion in mexico a randomized controlled noninferiority trial
622. observations on abortion in zambia
623. observations on female sterilization in chile
624. observations on trypanosomiasis in the belgian congo
625. obstetric and gynecologic dysfunction in the ehlersdanlos syndrome
626. obstetric and perinatal outcome of teenage pregnancy
627. obstetric determinants of neonatal survival antenatal predictors of neonatal survival and morbidity in extremely low birth weight infants
628. occurrence and determinants of psychological distress among women undergoing abortion/medical termination of pregnancy
629. of human rights and women's health
630. office versus telephone followup after medical abortion
631. on inhuman practices in gynecology and their victims in germany during nationalsocialist rule a study of concrete events
632. oncologic and fertility outcomes after simple trachelectomy in women with early cervical cancer
633. online availability of mifepristone and misoprostol
634. opinion and use of contraceptives among medical students of the university of nigeria, enugu campus
635. options in making use of pregnancy history in planning and analysing studies of reproductive failure
636. oral contraception noncompliance the extent of the problem
637. orientame preventing and solving problems related to unwanted pregnancy for 25 years in colombia
638. oriéntame preventing and solving problems related to unwanted pregnancy for 25 years in colombia
639. outbreaks of porcine reproductive failure report on a collaborative field investigation
640. outcome of firsttrimester exposure to lowdose methotrexate in eight patients with rheumatic disease
641. outcome of noac exposure during pregnancy ( and the problem of event reporting )
642. outcome of pregnancy in takayasu arteritis
643. outcomes of legal induced abortion at 910 weeks ' gestation in adult women
644. outpatient mifepristonemisoprostolmedical abortion through 77 days of gestation
645. pain assessment during medical abortion up to 14 weeks a 1year prospective comparative study
646. parsing the qmarkers of baoyin jian to treat abnormal uterine bleeding by highthroughput chinmedomics strategy
647. partialbirth abortion, congress, and the constitution
648. pathopharmacology of excessive hemorrhage in mifepristone abortions
649. pattern of congenital heart disease among egyptian children a 3year retrospective study
650. pediatrics
651. perampanel and pregnancy
652. pérdida recurrente del embarazo e hipertensión pulmonar idiopática
653. periabortion care when is it too much and when too little?
654. pericentric inversion of human chromosome 9 epidemiology study in czech males and females
655. perinatal outcomes after organ preserving surgery in patient with ovarian cancer
656. personal accounts of 'nearmiss' maternal mortalities in kampala, uganda
657. pharmacy dispensing of abortion pills in ghana experiences of pharmacy workers and users
658. pharmacy workers in nepal can provide the correct information about using mifepristone and misoprostol to women seeking medication to induce abortion
659. phase iii clinical trial with norplant ii (two covered rods) report on five years of use
660. pitfalls in the diagnosis of infection following medical termination of pregnancy
661. placenta previa; prevalence, risk factor and outcome
662. placental abnormalities in equine pregnancies generated by scnt from one donor horse
663. placental polyp an unusual hysteroscopic finding
664. planned parenthood continues prescribing birth control for teens
665. planned parenthood of rocky mountains v owens
666. planned parenthood of southern arizona v lawall
667. polidocanol foam for nonsurgical permanent female contraception initial trial in baboons
668. post abortion syndrome myth or reality?
669. post legalisation challenge minimizing complications of abortion
670. postabortion care ethical and legal duties
671. postabortion contraception method mix and uptake in ipassupported public health facilities in ethiopia
672. postcoital contraception a delicate political issue
673. postcoital contraception a report from the london brook advisory centres, united kingdom
674. postcoital contraception some characteristics of women who use this method
675. potential use of single measurement of serum progesterone in detecting early pregnancy failure
676. practice bulletin no 143 medical management of firsttrimester abortion
677. practice of unsafe abortion in pakistan characteristics and outcome
678. predicting poor compliance with followup and intrauterine contraception services after medical termination of pregnancy
679. prediction of late failure after medical abortion from serial βhcg measurements and ultrasonography
680. predictors of pregnancy outcome in essential thrombocythemia a single institution study of 63 pregnancies
681. preemptive effect of ibuprofen versus placebo on pain relief and success rates of medical abortion a doubleblind, randomized, controlled study
682. pregnancies, growth and development of children conceived by subzonal injection of spermatozoa
683. pregnancy after heart valve replacement
684. pregnancy after heartvalve replacement
685. pregnancy and lupus nephritis
686. pregnancy and systemic lupuserythematosus
687. pregnancy complications in sickle cell disease are more prevalent in women with vasoocclusion related organ damage than hemolysis related organ damage
688. pregnancy course in patients with interstitial lung diseases
689. pregnancy following uterine artery embolization with polyvinyl alcohol particles for patients with uterine fibroid or adenomyosis
690. pregnancy in antiphospholipid syndrome outcomes and risk factorsdata from a portuguese multidisclinary unit
691. pregnancy in pateints with exstrophyepispadias complex are higher rates of complications and spontaneous abortion inevitable?
692. pregnancy in patients with prosthetic and homograft heart valves maternal and fetal outcome
693. pregnancy in wilson's disease management and outcome
694. pregnancy outcome after suicide attempt by drug use a danish populationbased study
695. pregnancy outcome in women exposed to diethylstilbestrol inutero
696. pregnancy outcomes in antiphospholipid syndrome 8 yearexperience from a multidisciplinary unit
697. pregnancy outcomes in multiple sclerosis patients previously treated with cyclophosphamide
698. pregnancy rates and outcomes of hivinfected women in korea
699. pregnancy related acute renal failure still a major problem
700. pregnancy termination techniques, risks, and complications and their management
701. pregnancy toxemia and lipid mobilization syndrome in two alpaca (vicugna pacos) at 6 and 10 months of gestation
702. pregnancy, fertility, and recurrence risk in corrected tetralogy of fallot
703. preimplantation genetic screening among women experiencing recurrent failure of in vitro fertilization
704. prenatal diagnosis and late termination a legal perspective
705. prenatal risk factors for optic nerve hypoplasia
706. prescription for profit how doctors defraud medicaid
707. presenting features of women with uterine arteriovenous malformations
708. prevalence of anaemia in women with unsupervised medical abortion an observational study
709. prevalence of and attitudes to abortion among migrant women in sydney
710. prevalence of morbidity associated with abortion before and after legalisation in south africa
711. prevalence of self induced abortion by selfadministration of abortive pills among abortionrelated admissions in a tertiary care centre
712. prevalence of unprofessional social media content among young vascular surgeons (retracted article see vol 72, pg 1514, 2020)
713. prevention of rh haemolytic disease
714. primary cause of death in extremely low birth weight infants
715. problems loom ahead
716. profile of women receiving secondtrimester safe abortion service at paropakar maternity and women’s hospital
717. progesterone implantation in habitual abortion
718. progesteronemediated reversal of mifepristoneinduced pregnancy termination in a rat model an exploratory investigation
719. progestinbased contraceptive on the same day as medical abortion
720. prophylactic compared with therapeutic ibuprofen analgesia in firsttrimester medical abortion a randomized controlled trial
721. prospective study of home use of mifepristone and misoprostol for medical abortion up to 10 weeks of pregnancy in kazakhstan
722. prospective study of home use of mifepristone and misoprostol for medical abortion up to 10weeks of pregnancy in kazakhstan
723. prospective study of medical abortion in nepal medical college teaching hospital (nmcth) a one year experience
724. prostacyclin deficiency in a young woman with recurrent thrombosis
725. prostaglandine2 induction of abortion and fetal demise
726. prostaglandins and mifepriston in stimulation of medical abortion
727. protecting safe abortion in humanitarian settings overcoming legal and policy barriers
728. protective effect of taohong siwu decoction on abnormal uterine bleeding induced by incomplete medical abortion in rats during early pregnancy
729. providing accessible medical abortion services in a victorian rural community a description and audit of service delivery and contraception follow up
730. providing mifepristonemisoprostol medical abortion the view from the clinic
731. provision of medical abortion by midlevel healthcare providers in kyrgyzstan testing an intervention to expand safe abortion services to underserved rural and periurban areas
732. provision of medical abortion using telemedicine in brazil
733. psychiatric morbidity and acceptability following medical and surgical methods of induced abortion
734. psychiatric morbidity and acceptibility following medical and surgical methods of induced abortion
735. psychological responses following medical abortion (using mifepristone and gemeprost and surgical vacuum aspiration a patientcentered, partially randomised prospective study
736. psychological responses following medical abortion (using mifepristone and gemeprost) and surgical vacuum aspiration a patientcentered, partially randomised prospective study
737. public law no 262, 25 march 1988
738. putting the icpd plan of action to work turkey women's health tops the agenda
739. pxr mediates mifepristoneinduced hepatomegaly in mice
740. quality focuses on clients' needs
741. radiology rounds intrauterine contraceptive device
742. randomized study on the effect of adding oxytocin to ethacridine lactate or misoprostol for secondtrimester termination of pregnancy
743. reasons due to which, women resort to illegally induced abortions
744. recent developments of medical law
745. recours à l'avortement provoqué à lomé (togo) évolution, facteurs associés et perceptions
746. reduced fertility among women employed as dental assistants exposed to high levels of nitrous oxide
747. religion, conscience, and controversial clinical practices
748. replacement of dilation and curettage/evacuation by manual vacuum aspiration and medical abortion, and the introduction of postabortion contraception in pakistan
749. replacing the dead the politics of reproduction in the postwar soviet union, 19441955
750. reply of the authors setting up a medical abortion service for failure
751. report of survey pregnancy outcomes in medical laboratory technologists
752. representations and uses of emergency contraception in west africa a social anthropological reading of a northern medicinal product
753. reproductive health and blurred professional boundaries
754. reproductive health crisis during waves one and two of the covid19 pandemic in india incidence and deaths from severe maternal complications in more than 202,000 hospital births
755. reproductive health information and abortion services standards developed by the european court of human rights
756. reproductive patterns as environmental markers in rural morocco
757. reproductive performance of patients treated with clomiphene citrate
758. researches in aetiology of nonspecific urethritis
759. restrictions in access to abortion the italian situation
760. restrictive state abortion bansa reproductive injustice
761. results and lessons learned from a small medical abortion clinical study in turkey
762. results of first trimester medical abortion care
763. resurgence of congenital rubella syndrome in the 1990s report on missed opportunities and failed prevention policies among women of childbearing age
764. retraction notice (journal of vascular surgery (2020) 72(2) (667671), (s074152141932587x), (101016/jjvs201910069))
765. retrospective analysis of prognostic value of the neutrophiltolymphocyte ratio in early miscarriages a 8year survey
766. retrospective observational assessment of the hazards of otc availability of mtp pills
767. right tool, wrong job manual vacuum aspiration, postabortion care and transnational population politics in senegal
768. risk factors and the choice of longacting reversible contraception following medical abortion effect on subsequent induced abortion and unwanted pregnancy
769. risk factors for intrauterine device embedment in postmenopausal women an analysis of 731 participants undergoing hysteroscopy
770. risk management in pregnancy termination
771. risk of complications during pregnancy in women with congenital aortic stenosis
772. risks and costs of illegally induced abortion in bangladesh
773. risks and costs of illegally inducedabortion in bangladesh
774. rocking the cradle the uncertain policy of withholding treatment from anomalous newborns (infanticide, mercykilling, defective infants, selective nontreatment, euthanasia)
775. role of isosorbide mononitrate as an agent for cervical ripening in secondtrimester abortions
776. role of ultrasound in first trimester bleeding
777. routine followup versus selfassessment of complete abortion following medical abortion, effect on its success and acceptability a randomized controlled trial
778. ru486 misconceptions, myths and morals a critique of the criticisms
779. rubella vaccine in postpubertal women experience in western washington state
780. safety and efficacy of medical abortion in a pediatric population
781. safety and efficacy of outpatient mifepristonemisoprostol medical abortion through 76 days of gestational ageportuguese experience in a tertiary hospital
782. safety and feasibility of office hysteroscopy in removing retained products of conception
783. safety of induced abortions at less than 12 weeks of pregnancy in japan
784. scar ectopic pregnancy type ii / endogenic with impending scar rupture
785. scenario of self medication for medical abortion in a tertiary care centre
786. second and thirdtrimester termination of pregnancy in women with uterine scar a retrospective analysis of 111 gemeprostinduced terminations of pregnancy after previous cesarean delivery
787. second trimester abortions in india
788. second trimester medical abortion in a primigravida with lupus nephritis and rapidly progressive renal failure challenges and outcome
789. secondtrimester medication abortion outside the clinic setting an analysis of electronic client records from a safe abortion hotline in indonesia
790. secondtrimester surgical and medical abortion practice in canada in 2012 a national survey
791. secular trends in uncertainsex births and proportion of male births in norway, 19671998
792. self administration versus supervised use of medical abortion pills impact on women’s health
793. self reported outcomes and adverse events after medical abortion through online telemedicine population based study in the republic of ireland and northern ireland
794. selfadministered multilevel pregnancy tests in simplified followup of medical abortion in tunisia
795. selfassessment of the outcome of early medical abortion versus clinic followup in india a randomised, controlled, noninferiority trial
796. septic abortion a 5year experience at siriraj hospital
797. septic abortion personal experience and management
798. septic pelvic thrombophlebitis of unknown origin an ever threatening entity
799. sequential use of foley catheter with misoprostol for second trimester pregnancy termination in women with and without caesarean scars a prospective cohort study
800. seroprevalence of babesia caballi and theileria equi in horses in central germany
801. service delivery correlates of choosing shortacting contraceptives at the time of uterine evacuation in bangladesh
802. setting up a medical abortion service for failure
803. setting up a medical abortion service for failure reply
804. severe acute maternal morbidity a pilot study of a definition for a nearmiss
805. sexual activity in girls under 16 years of age
806. sexual knowledge and attitudes of adolescents relationship to contraceptive use
807. sexualactivity in girls under 16 years of age
808. sexuality, words and familyplanning
809. short and long term mortality rates associated with first pregnancy outcome population register based study for denmark 19802004
810. should organs from patients in permanent vegetative state be used for transplantation?
811. side effects of induced abortion results from a populationbased survey
812. sífilis congénita auditorías en tiempo real
813. significance of clinical and anamnestic risk factors for the prediction of negative outcomes in assisted reproductive technology programs
814. simplified medical abortion screening a demonstration project
815. simultaneous administration of mifepristone and misoprostol increases access to early medical abortion where homeuse of misoprostol is not permitted
816. simultaneous compared with interval medical abortion regimens where home use is restricted
817. síndrome de hellp en el hospital nacional guillermo almenara irigoyen presentación clínica y complicaciones de una emergencia obstétrica
818. situation analysis of patients attending tu teaching hospital after medical abortion with problems and complications
819. social and medical implications of teenage motherhood
820. social questions, medical answers contesting british abortion law
821. soluble mhc class i chainrelated molecule serum levels are predictive markers of implantation failure and successful term pregnancies following ivf
822. some errors after the medical abortion recovery period and contraception choice
823. some psychiatric aspects of abortion
824. spanish abortion reform faces opposition
825. sperm proteins as targets for contraception and fertility biomarkers
826. spontaneous foetal losses in women using different contraceptives around the time of conception
827. standardized second trimester medical abortion training and service provision in ethiopia, october 2010september 2014
828. status of male contraception
829. strategies for nursing uterine rupture in primiparas during pregnancy
830. structural barriers or patient preference? a mixed methods appraisal of medical abortion use in england and wales
831. structural equation modelling analysis determining causal role among methyltransferases, methylation, and apoptosis during human pregnancy and abortion
832. structure of reproductive losses of adolescent girls and women of reproductive age in the tyumen
833. study of pregnancy outcome over a period of five years in a postgraduate institute of west bengal
834. study of spontaneous abortion in thailand
835. study of 'spontaneous' abortion in thailand
836. study of the relation between preterm delivery and periodontitis the role of prophylactic dental therapy in the prevention of preterm delivery
837. study on clinical presentation and outcome of septic abortion and its relationship with person inducing abortion
838. study results translate to better services
839. successful medical abortion of a pregnancy within a noncommunicating rudimentary uterine horn
840. successful surgical treatment of invasive pancreatic ductal adenocarcinoma during pregnancy
841. successful termdelivery following medically managed 9 weeks live caesarean scar pregnancy
842. suggested fda restrictions might curtail access to mifepristone
843. suggestions on revision regarding abortion laws in korea an empirical study using qualitative research method
844. surgical management of early pregnancy failure history, politics, and safe, costeffective care
845. surgical management of incomplete abortion by manual vacuum aspiration (mva)
846. survey of the attitude to, knowledge and practice of contraception and medical abortion in women attending a family planning clinic
847. survey of the attitude to, the knowledge and the practice of contraception and medical abortion in women who attended a family planning clinic
848. survey women educational needs about routine ocps
849. syphilitic endometritis causing first trimester abortion a potential infectious cause of fetal morbidity in early gestation
850. systemic inflammatory response syndrome, organ failure, and outcome in critically ill obstetric patients treated in an icu
851. tanzanian lessons in using nonphysician clinicians to scale up comprehensive emergency obstetric care in remote and rural areas
852. teen pregnancy effect on family wellbeing
853. telemedicine medical abortion at home under 12 weeks' gestation a prospective observational cohort study during the covid19 pandemic
854. telephone followup after early medical abortion using australia's first low sensitivity urine pregnancy test
855. tenminute abortions
856. termination of a pregnancy located in one horn of a bicornuate uterus challenges and solution
857. termination of early pregnancy by the 3βhydroxysteroid dehydrogenase inhibitor epostane
858. termination of early pregnancy by two regimens of mifepristone with misoprostol and mifepristone with pg05a multicentre randomized clinical trial in china
859. termination of pregnancy in papua new guinea the traditional and contemporary position
860. termination of pregnancy in papuanewguinea the traditional and contemporary position
861. the abortion battle the canadian scene
862. the abortion paradox
863. the abortion pill a solution for unsafe abortions in developing countries?
864. the applied of computer for analysis on contraceptive efficacy of iud of rural women in guangdong province
865. the association between history of retained placenta and success rate of misoprostol treatment for early pregnancy failure
866. the characteristics of and the contraceptive practice among women seeking therapeutic termination of pregnancy in the scottish highlands
867. the clinical management of abortion
868. the clinical use of karyotyping spontaneous abortions
869. the contraceptive choice at the time of a surgical and pharmacological abortion a possible and effective option thanks to a dedicate counselling
870. the contraceptive practice of abortion patients
871. the cost of child rearing for wrongful conception
872. the counseling component of medical abortion
873. the culture of illegal abortion in south africa
874. the determinants and health consequences of unsafe abortion in rio de janeiro, brazil
875. the diagnosis of early pregnancy failure by sonar
876. the difficulties of contraception conflicts and paradoxes
877. the effect of hysteroscopic metroplasty on fertility in tshaped and yshaped dysmorphic uterus
878. the effect of smoking on reproductive failures in couples examined in the genetic outpatient clinic at dr a jurasz university hospital in bydgoszcz
879. the effects of religious beliefs on the health care practices of the amish
880. the effects of the antihormones ru486 and tamoxifen on fetoplacental development and placental bed vascularisation in the rat a model for intrauterine fetal growth retardation
881. the ethical legacy of hippocrates
882. the experience of carrying out the technical responsibility system in the birth control of peng county, sichuan province
883. the experiences of women accessing legal abortion in mexico city
884. the feasibility of a modified method of laparoscopic transabdominal cervicoisthmic cerclage during pregnancy
885. the feasibility of early 2nd trimester pregnancy (12 to 14 weeks) termination with single intraamniotic injection of 15methyl pgf2alpha
886. the feasibility of offering medical abortions by tele medicine 20 months of experience
887. the fight for reproductive rights in central and eastern europe poland catholic backlash
888. the frequency of chromosomal abnormalities in patients with reproductive failure
889. the frequency of chromosomal, abnormalities in patients with reproductive failure
890. the gag rule revisited physicians as abortion gatekeepers
891. the hemostatic system with medical abortion
892. the hospital's role in contraception
893. the immunobiology of abortion
894. the impact of the covid19 pandemic on the course of miscarriages
895. the impact of the prenatal ultrasonography on birth of babies with korean pediatric surgical index diseases
896. the influence of contraceptive practice upon maternal and child health
897. the introduction of first trimester medical abortion in armenia
898. the invisible pregnant athlete and the promise of title ix
899. the language of firsttrimester nonviable pregnancy patientreported preferences and clarity
900. the legal implications of prenatal diagnosis in malaysia
901. the legal status of the fetus as a patient in europe
902. the midwife's role in abortion care
903. the modern abortion jurisprudence under article 8 of the european convention on human rights
904. the morphology, typical combinations of polymorphic genes of hemostasis, and specific features of the pathogenesis of retrochorial hematoma in missed abortion
905. the outcome of home medical abortions provided through telemedicine
906. the outcome of in vitro fertilization in unexplained habitual aborters concurrent with secondary infertility
907. the outcome of septic abortion a tertiary care hospital experience
908. the pattern of maternal mortality at maternity hospital kuala lumpur
909. the political challenges and educational opportunities around very early abortion
910. the portrayal and perceptions of cesarean section in mexican media facebook pages a mixedmethods study
911. the position of general practitioner midwifery
912. the preemptive effect of ibuprofen versus placebo on pain relief and success rates of medical abortion a doubleblind randomised controlled study
913. the pregnant plastic surgical resident results of a survey of women plastic surgeons and plasticsurgery residency directors
914. the problem of teenage pregnancy
915. the profile of women who seek emergency contraception from the family planning service
916. the relationship between insecurity and the quality of hospital care provided to women with abortionrelated complications in the democratic republic of congo a crosssectional analysis
917. the risk of birth defects jacobs v theimer and parents' right to know
918. the role of auxiliary nursemidwives and community health volunteers in expanding access to medical abortion in rural nepal
919. the role of medical abortion in the implementation of the law on voluntary termination of pregnancy in uruguay
920. the safety and efficacy of chorionic villus sampling for early prenatal diagnosis of cytogenetic abnormalities
921. the spectrum of relapsing fever in the rocky mountains
922. the telabortion project delivering the abortion pill to your doorstep by telemedicine and mail
923. the theory and practice of self ownership
924. the trophoblastic blood flow during the course of medical abortion
925. the unwanted child
926. the use of manual vacuum aspiration in the treatment of incomplete abortions a descriptive study from three public hospitals in malawi
927. the use of prostaglandins in obstetrics
928. the value of posterior cervical angle as a predictor of vaginal delivery a preliminary study
929. thelper 1type immunity to trophoblast in women with recurrent spontaneous abortion
930. thelper 1type immunity to trophoblast in women with recurrent spontaneousabortion
931. therapeutic abortion by intraamniotic injection of prostaglandins
932. third time unlucky a study of women who have three or more legal abortions
933. thrombophilia, anticoagulant therapy, and pregnancy outcome in women with poor obstetric history
934. timing and indication for curettage after medical abortion in early pregnant women with prior uterine incision
935. tissue distribution of cocaine in a pregnant woman
936. toward a standard measure of abortion service qualitya stakeholder first approach
937. toxic shock associated with clostridium sordellii and clostridium perfringens after medical and spontaneous abortion
938. transplacental passage of fetal red cells in abortion; increased incidence after curettage and effect of oxytocic drugs
939. trauma in pregnancy predicting pregnancy outcome
940. treatment of epilepsy in women of childbearing age
941. triangular assessment of the etiology of induced abortion in iran a qtualitative study
942. triangular assessment of the etiology of induced abortion in iran a qualitative study
943. tucson woman's clinic v eden
944. ulipristal acetate and pregnancy outcomean observational study
945. ultrasonographic characteristics in patients clinically diagnosed with threatened abortion
946. ultrasonographic evaluation of endometrial changes using computer assisted image analysis
947. umbilical cord lesions in early intrauterine fetal demise
948. uncomplicated abortion with mifepristone and misoprostol in a hemophilia a carrier
949. understanding of changes in abortion rate following liberalization of abortion law among brazilian residents in obstetrics and gynecology
950. unsuccessful prior attempts to terminate pregnancy among women seeking first trimester abortion at registered facilities in bihar and jharkhand, india
951. unusual intrauterine objects potential pitfalls in ultrasonographic identification
952. uptake and outcome of artificial reproductive techniques following allogeneic stem cell tranplantation a single centre experience
953. usa victory over gag rule for family planning groups
954. use of an athome multilevel pregnancy test and an automated telephone system to simplify medical abortion followup
955. use of misoprostol during pregnancy and mobius syndrome in infants
956. use of misoprostol in gynecology and obstetrics
957. use of platelet rich plasma in human infertility
958. use of prostaglandin for induction of second trimester abortions in high risk pregnancy
959. use of quinine for selfinduced abortion
960. using telemedicine to improve early medical abortion at home (utah) a randomised controlled trial to compare telemedicine with inperson consultation for early medical abortion
961. utah using telemedicine to improve early medical abortion at home a protocol for a randomised controlled trial comparing facetoface with telephone consultations for women seeking early medical abortion
962. uterine artery embolization in the treatment and prevention of postpartum hemorrhage
963. uterine aspiration for evacuation of the pregnant uterus
964. uterine preservation surgery for placental polyp
965. uterine rupture during pregnancy and delivery risk factors, symptoms and maternal and neonatal outcomes restrospective cohort
966. utility of manual vaccum aspiration (mva) in a tunisian tertiary referral center
967. vaccination against influenza in pregnant women safety and effectiveness
968. vaginal pge2 and intraamniotic pgf2 alpha for the termination of second trimester pregnancy
969. validation of a spanishlanguage scale for evaluating perceived quality of care of medical abortions before 9 weeks gestation
970. validation of icd9 codes for stable miscarriage in the emergency department
971. value of hysteroscopy and laparoscopy in differential diagnosis of gestational trophoblastic neoplasia
972. vasectomy on request still a moot point for discussion
973. what is needed to ensure the health and survival of mother and baby?
974. when is emergency contraception the right name for postcoital treatment?
975. which evacuation method for missed abortion is preferred by women with recurrent pregnancy loss?
976. who will do the abortions?
977. why is access to medical abortion limited in the russian federation?
978. womens reproductive health
979. women's selfreported experiences using misoprostol obtained from drug sellers a prospective cohort study in lagos state, nigeria
980. y to x translocation in a woman with reproductive failure a new rearrangement
981. yoruba traditional healers' knowledge of contraception, abortion and infertility
982. your conscience, your right a history of efforts to violate prolife medical conscience, and the laws that stand in the way
983. youth often risk unsafe abortions

**4. Full-text articles assessed for eligibility (n=224)**

1. 10 year evaluation of the use of medical abortion through telemedicine a retrospective cohort study
2. 200 mg mifepristone was as effective as 600 mg when used with oral misoprostol for early medical abortion
3. 2017 19 governmental decisions to allow home use of misoprostol for early medical abortion in the uk
4. 346 efficacy and safety of mifepristone and sublingual misoprostol for second trimester medical abortion a prospective study
5. a clinical evaluation of the safety and efficacy of mefepristone and misoprostol in medical abortions
6. a comparative study on sublingual versus oral and vaginal administration of misoprostol for late first and early second trimester abortion
7. a comparison of misoprostol with and without laminaria tents for induction of secondtrimester abortion
8. a comparison of misoprostol with and without methylergometrine and oxytocin in outpatient medical abortion a phase iii randomized controlled trial
9. a comparison of surgical vacuum aspiration abortion with medical abortion using mifepristone (ru 486) and gemeprost implications for nursing staff
10. a comparison of the costs of manual vacuum aspiration (mva) and evacuation and curettage (e and c) in the treatment of early incomplete abortions in kenya
11. a comparison of the efficacy of sublingual and oral misoprostol 400 microgram in the management of early pregnancy failure a randomized controlled trial
12. a comparison of the safety and efficacy of three medical abortion protocols
13. a comparison of the safety and efficacy of two medical abortion protocols
14. a comparitive study of use of intravaginal misoprostol after 48 hours and 4 hours of oral mifepristone for medical termination of pregnancy
15. a noninferiority study of outpatient mifepristonemisoprostol medical abortion at 6470 days and 7177 days of gestation
16. a phase iii multicentre study on medical termination of early pregnancy with two regimens of mifepristone followed by pg05
17. a pilot study of mifepristone and misoprostol administered at the same time for abortion up to 49 days gestation
18. a prospective randomized, doubleblinded, placebocontrolled trial comparing mifepristone and vaginal misoprostol to vaginal misoprostol alone for elective termination of early pregnancy
19. a prospective trial using mifepristone and vaginal misoprostol in termination of pregnancies up to 63 days of gestation
20. a prospective, comparative study of clinical outcomes following clinicbased versus selfuse of medical abortion
21. a twopill sublingual misoprostol outpatient regimen following mifepristone for medical abortion through 70 days' lmp a prospective comparative openlabel trial
22. a viable alternative to surgical vacuum aspiration repeated doses of intravaginal misoprostol over 9 hours for medical termination of pregnancies up to eight weeks
23. abortion in four asian countries patient characteristics, morbidity and contraceptive acceptance
24. abortion in the first trimester guideline of the dggg (s2klevel, awmf registry no 015094, december 2022) part 1 with recommendations on care structures, information and advice on decisionmaking, measures before abortion and medical abortion
25. abortion policy and womens health in developingcountries
26. acceptability and feasibility of early pregnancy termination by mifepristonemisoprostol results of a large multicenter trial in the united states mifepristone clinical trials group
27. acceptability and feasibility of outpatient medical abortion with mifepristone and misoprostol up to 70 days gestation in singapore
28. adolescent girls undergoing medical abortion have lower risk of haemorrhage, incomplete evacuation or surgical evacuation than women above 18 years old
29. association between uterine position and transvaginal misoprostol treatment for early pregnancy failure
30. association of the g2014g genotype in estrogen receptor 1 gene with failure of the mifepristoneinduced termination of early pregnancy
31. bleeding patterns after vaginal misoprostol for treatment of early pregnancy failure
32. can women accurately assess the outcome of medical abortion based on symptoms alone?
33. change in serum betahuman chorionic gonadotropin after abortion with methotrexate and misoprostol
34. change in serum βhuman chorionic gonadotropin after abortion with methotrexate and misoprostol
35. clinical followup compared with selfassessment of outcome after medical abortion a multicentre, noninferiority, randomised, controlled trial
36. clinical study of early pregnancy termination by administration of dl15methyl prostaglandin f2 alpha combined with tamoxifen or norethisterone
37. clinical study of terminating biochemical pregnancy and early clinical pregnancy with mifepristone and misoprostol
38. clinical study of termination of early pregnancy by combination of dl15methylprostaglandin f2 alpha and ru 486
39. clinical trial on termination of early pregnancy with ru486 in combination with prostaglandin
40. clinical use and safety of medical method of first trimester abortion
41. clinicaltrial on termination of earlypregnancy with ru486 in combination with prostaglandin
42. comparative study of efficacy and acceptability of two regimens for 1st trimester mtp
43. comparative study of mifepristone with vaginal misoprostol for first trimester termination of pregnancy at different gestational ages
44. comparative study of regimen mifepristone and misoprostol with misoprostol alone at first trimester termination of pregnancy
45. comparing the effects of misoprostol/letrozole and misoprostol/placebo on medical abortion success rate a randomized clinical trial
46. comparison of misoprostolonly and combined mifepristonemisoprostol regimens for homebased early medical abortion in tunisia and vietnam
47. Comparison of rates of adverse events in adolescent and adult women undergoing medical abortion: population register based study
48. comparison of sublingual, oral and vaginal misopristol for cervical ripening 12 hours before hysteroscopy
49. comparison of the efficacy and safety of two combined misoprostol regimens for second trimester medical abortion
50. comparison of two doses of mifepristone in combination with misoprostol for early medical abortion a randomised trial
51. comparison of vaginal and sublingual misoprostol for second trimester abortion randomized controlled equivalence trial
52. comparitive study of early (24 hours) versus late (48 hours) misoprostol administration after mifepristone for termination of early pregnancy
53. consistency of medical abortion efficacy from 5 through 14 weeks' gestation
54. declining risk of surgical intervention following early medical abortion a time trend analysis
55. depot medroxyprogesterone acetate (dmpa) administration at the time of mifepristone for medical abortion a pilot study
56. does followup with serum hcg simplify medical abortion? a randomized controlled trial
57. does methotrexate confer a significant advantage over misoprostol alone for early medical abortion? a retrospective analysis of 8678 abortions
58. doubleblind randomized trial of mifepristone in combination with vaginal gemeprost or misoprostol for induction of abortion up to 63 days gestation
59. early abortion with buccal versus sublingual misoprostol alone a multicenter, randomized trial
60. early medical abortion using lowdose mifepristone followed by buccal misoprostol a large australian observational study
61. early medical abortion with selfadministered lowdose mifepristone in combination with misoprostol
62. early versus late misoprostol administration after mifepristone for medical abortion
63. Effect of Previous Live Birth and Prior Route of Delivery on the Outcome of Early Medical Abortion
64. effectiveness and acceptability of home use of misoprostol for medical abortion up to 10 weeks of pregnancy
65. effectiveness and safety of early medication abortion provided in pharmacies by auxiliary nursemidwives a noninferiority study in nepal
66. effectiveness of early medical abortion using lowdose mifepristone and buccal misoprostol in women with no defined intrauterine gestational sac
67. effectiveness of selfmanaged medication abortion between 9 and 16 weeks of gestation
68. efficacy and acceptability of a mifepristonemisoprostol combined regimen for early induced abortion among women in mexico city
69. efficacy and safety of intravaginal misoprostol for midtrimester medical termination of pregnancy
70. efficacy and safety of mifepristone and buccal misoprostol versus buccal misoprostol alone for medical abortion
71. efficacy and safety of mifepristonebuccal misoprostol for early medical abortion in an australian clinical setting
72. efficacy of combination therapy with methotrexate and misoprostol in termination of pregnancy in the first trimester
73. efficacy of concurrent administration of mifepristone and misoprostol for termination of pregnancy
74. efficacy of mifepristone and misoprostol in late first trimester medical abortion, missed abortion and blighted ovum
75. efficacy of misoprostol administration 24 hours after mifepristone for termination of early pregnancy
76. efficacy of misoprostol in relation to uterine position in the treatment of early pregnancy failure
77. efficacy of single dose of mifepristone combined with two doses of misoprostol in early medical abortions
78. efficacy of single dose vaginal misoprostol 800μg in i trimester abortion
79. elevated mrna expression of pgf2 receptor splice variant 2(fpv2) in human decidua is associated with incomplete mifepristonemisoprostolinduced early medical abortion by regulation of interleukin8
80. endometrial thickness and serum betahcg as predictors of the effectiveness of oral misoprostol in early pregnancy failure
81. endometrial thickness and serum βhcg as predictors of the effectiveness of oral misoprostol in early pregnancy failure
82. endometrial thickness as predictor of the effectiveness of intracervical misoprostol in early pregnancy failure
83. endometrial thickness following medical abortion is not predictive of subsequent surgical intervention
84. evaluation of effect of letrozole prior to misoprostol in comparison with misoprostol alone in success rate of induced abortion
85. evaluation of the success of medical abortion by a plasma hcg control threshold
86. evaluation of ultrasound needs in first trimester medical abortion with misoprostol
87. expanding a woman's options to include home use of misoprostol for medical abortion up until 76 days an observational study of efficacy and safety
88. experiences with gemeprost in 1sttrimester termination of pregnancy
89. experiences with the progesterone antagonist mifepristone (ru 486) for termination of early pregnancy
90. extending outpatient medical abortion services through 70 days of gestational age
91. factors affecting gestational age at termination of pregnancy
92. Factors affecting the outcome of early medical abortion: a review of 4132 consecutive cases
93. factors influencing the abortion interval of second trimester pregnancy termination using misoprostol
94. factors related to completeness of medical abortion with mifepristone and misoprostol
95. Failure Rate of Medical Treatment for Miscarriage Correlated with the Difference between Gestational Age According to Last Menstrual Period and Gestational Size Calculated via Ultrasound
96. first trimester abortion with mifepristone and vaginal misoprostol
97. firsttrimester pregnancy failure
98. further acceptability evaluation of ru486 and ono 802 as abortifacient agents in a chinese population
99. high failure rates of medical termination of pregnancy after introduction to a large teaching hospital
100. highdose misoprostol as an alternative therapy after failed medical abortion
101. home selfadministration of misoprostol for medical abortion up to 56 days' gestation
102. home selfadministration of vaginal misoprostol for medical abortion at 50 to 63 days compared with gestation of below 50 days
103. ibuprofen and paracetamol for pain relief during medical abortion a doubleblind randomized controlled study
104. importance of a second dose of misoprostol in the management of medical abortion after failure of the initial treatment
105. Increased risk for medical abortion failure for multiparous women
106. increasing women's choices in medical abortion a study of misoprostol 400 microg swallowed immediately or held sublingually following 200 mg mifepristone
107. induced abortion incidence and trends worldwide from 1995 to 2008
108. induction of abortion during the 2nd trimenon of pregnancy endocervical pge2 gel application, intramuscular application of sulproston and combined treatment (endocervical pge2 gel intramuscular sulproston)
109. induction of abortion in earlypregnancy with mifepristone in conjunction with gemeprost
110. induction of abortion with mifepristone and misoprostol in early pregnancy
111. initiating intramuscular depot medroxyprogesterone acetate 2448 hours after mifepristone administration does not affect success of early medical abortion
112. is mifepristone 100mg an effective alternative to standard dose for medical abortion
113. issues in second trimester induced abortion (medical/surgical methods)
114. lowdose mifepristone followed by vaginal misoprostol at 48 hours for abortion up to 63 days
115. lowering the doses of mifepristone and gameprost for early abortion a randomised controlled trial world health organization task force on postovulatory methods for fertility regulation
116. lowering the doses of mifepristone and gemeprost for early abortion a randomised controlled trial
117. medical abortion a life saving technology
118. medical abortion a practical quick reference algorithm to support the gynecologists
119. medical abortion after 9 weeks
120. medical abortion at 57 to 63 days' gestation with a lower dose of mifepristone and gemeprost a randomized controlled trial
121. medical abortion at 63 to 90 days of gestation
122. medical abortion at 912 weeks
123. medical abortion defining success and categorizing failures
124. medical abortion followup with serum human chorionic gonadotropin compared with ultrasonography a randomized controlled trial
125. medical abortion in china
126. medical abortion in the second trimester an update
127. medical abortion in women of ≤56 days amenorrhoea a comparison between gemeprost (a pge(1) analogue) alone and mifepristone and gemeprost
128. medical abortion use of mifepristone and misoprostol in first and second trimesters of pregnancy
129. medical abortion using methotrexate and misoprostol efficacy and tolerability
130. medical abortion with methotrexate 75 mg intramuscularly and vaginal misoprostol
131. medical abortion with mifepristone and home administration of misoprostol up to 63 days' gestation
132. medical abortion with mifepristone and vaginal misoprostol between 64 and 70 days' gestation
133. medical termination of pregnancy during the second versus the first trimester and its effects on subsequent pregnancy
134. medical treatment with misoprostol for early failure of pregnancies after assisted reproductive technology a promising treatment option
135. methotrexate and misoprostol for early abortion in adolescent women
136. methotrexate and misoprostol used alone or in combination for early abortion
137. methotrexate and misoprostol vs misoprostol alone for early abortion a randomized controlled trial
138. mifepristone 100mg for early medical abortion
139. mifepristone and misoprostol administered simultaneously versus 24 hours apart for abortion a randomized controlled trial
140. mifepristone followed by home administration of buccal misoprostol for medical abortion up to 70 days of amenorrhoea in a general practice in curacao
141. mifepristone plus vaginal misoprostol vs vaginal misoprostol alone for medical abortion in gestation 63 days or less in nepalese women a quasirandomized controlled trial
142. minimal effective dose of mifepristone for medical abortion
143. misoprostol abortion ultrasonography versus betahcg testing for verification of effectiveness
144. misoprostol alonea new method of medical abortion?
145. misoprostol as the primary agent for medical abortion in a lowincome urban setting
146. misoprostol dose and route after mifepristone for early medical abortion a randomised controlled noninferiority trial
147. misoprostol for second trimester medical abortion a comparison of three routes of administration
148. monitoring medical abortion using mifepristone/misoprostol combination with ultrasonogram and serum human chorionic gonadotropin
149. multicenter trial of a simplified mifepristone medical abortion regimen
150. options in making use of pregnancy history in planning and analyzing studies of reproductive failure
151. oral methotrexate and vaginal misoprostol for early abortion
152. oral misoprostol administration in the first and second trimester for termination
153. outcome of first trimester induced abortions using misoprostol by buccal and vaginal routes
154. outcomes during early implementation of mifepristonebuccal misoprostol abortions up to 63 days of gestation in a canadian clinical setting
155. outpatient mifepristonemisoprostol medical abortion through 77 days of gestation
156. Parity is a major determinant of success rate in medical abortion: a retrospective analysis of 3161 consecutive cases of early medical abortion treated with reduced doses of mifepristone and vaginal gemeprost
157. placenta accreta a cause of failed medical abortion
158. prediction of late failure after medical abortion from serial betahcg measurements and ultrasonography
159. predictive value of peripheral blood α1acid glycoprotein in medical abortion outcomes with mifepristone and relativity of concentration
160. Predictors of uterine evacuation following early medical abortion with mifepristone and misoprostol
161. pregnancy the effect of dose of mifepristone and gestation on the efficacy of medical abortion with mifepristone and misoprostol
162. preliminary results of the role of semiquantitative pregnancy tests in medical abortion provision
163. randomised controlled trial comparing efficacy of same day administration of mifepristone and misoprostol for termination of pregnancy with the standard 36 to 48hour protocol
164. randomised controlled trial comparing the efficacy of sameday administration of mifepristone and misoprostol for termination of pregnancy with the standard 36 to 48 hour protocol
165. randomized trial of mifepristone and buccal misoprostol vs misoprostol alone for medical abortion
166. randomized trial of mifepristone and buccal or vaginal misoprostol for abortion through 56 days of last menstrual period
167. randomized trial of oral versus sublingual misoprostol 24 h after mifepristone for medical abortion
168. randomized trial of oral versus vaginal misoprostol 2 days after mifepristone 200 mg for abortion up to 63 days of pregnancy
169. randomized trial of oral versus vaginal misoprostol at one day after mifepristone for early medical abortion
170. randomized, doubleblind, controlled trial of mifepristone in capsule versus tablet form followed by misoprostol for early medical abortion
171. reducing the buccal dose of misoprostol in mifepristone medical abortion up to 63 days lmp
172. retrospective assessment of the influence of gestational age on the first trimester medical abortion efficiency
173. Risk factors for surgical intervention of early medical abortion
174. risk factors for unsuccessful medical abortion with mifepristone and misoprostol
175. risk of surgical evacuation and risk of major surgery following secondtrimester medical abortion in denmark a nationwide cohort study
176. role of routine ultrasonography in monitoring the outcome of medical abortion in a clinical setting
177. role of ultrasonography in evaluation of bleeding per vagina in first trimester of pregnancy
178. role of ultrasonography in the evaluation of causes of bleeding per vaginum in the first trimester of pregnancy
179. ru 486 (mifepristone) from clinicaltrials to the perspectives for clinical use
180. safe medical abortion in the second trimester of pregnancy
181. selfadministration of abortion pills and its maternal outcome in tertiary care center
182. selfassessment of medical abortion outcome using symptoms and home pregnancy testing
183. serum biomarkers may help predict successful misoprostol management of early pregnancy failure
184. serum βhcg concentration is a predictive factor for successful early medical abortion with vaginal misoprostol within 24 hours
185. simultaneous administration of mifepristone and misoprostol for early termination of pregnancy a randomized controlled trial
186. sonographic patterns of the endometrium in assessment of medical abortion outcomes
187. sonographic quantification of endometrial changes after abortion with computerassisted image analysis
188. sublingual v/s vaginal misoprostol for second trimester termination of pregnancy a comparative study
189. surgical intervention after medical abortion
190. tamoxifen compared to methotrexate when used with misoprostol for abortion
191. termination of 2nd trimester pregnancy with sulprostone and mifepristone a randomized doubleblind placebocontrolled trial
192. termination of early pregnancy by two regimens of mifepristone with misoprostol a multicentre clinical trial
193. termination of early pregnancy by two regimens of mifepristone with misoprostol and mifepristone with pg05 a multicentre randomized clinical trial in china
194. termination of early pregnancy using flexible, lowdose mifepristonemisoprostol regimens
195. termination of early pregnancy using ru 486 alone or in combination with prostaglandin the ru 486 collaboration group
196. termination of earlypregnancy by 2 regimens of mifepristone with misoprostol and mifepristone with pg05 a multicenter randomized clinicaltrial in china
197. termination of pregnancy at very early gestation without visible yolk sac on ultrasound
198. the effect of dose of mifepristone and gestation on the efficacy of medical abortion with mifepristone and misoprostol
199. the effect of misoprostol with and without letrozole in inducing successful medical abortion
200. the effect of nonsteroidal antiinflammatory drugs on medical abortion with mifepristone and misoprostol at 1322 weeks gestation
201. the effectiveness of home medical abortions provided through telemedicine
202. the efficacy and acceptability of mifepristone medical abortion with home administration misoprostol provided by private providers linked with the hospital a prospective study of 433 patients
203. the extended gestational age medical abortion study the effectiveness of medical abortion with mifepristone and misoprostol at 5763 days versus 6470 days gestation
204. the feasibility of early second trimester pregnancy (12 to 14 weeks) termination with single intra‐amniotic injection of 15‐methyl pgf2α
205. the induction of abortion in the 2nd trimester by combined administration of minprostin and sulproston, compared with the use of sulproston alone
206. the induction of abortion with prostaglandins
207. the medical management of incomplete abortion
208. the role of parity in medical abortion up to 49 days of amenorrhoea
209. therapeutic termination of second trimester pregnancies with low dose misoprostol
210. trend of serum betahuman chorionic gonadotropin levels after medical abortion in the early first trimester of pregnancy
211. trends in elective medical and surgical abortions in the united states during the covid19 pandemic
212. two mifepristone doses and two intervals of misoprostol administration for termination of early pregnancy a randomised factorial controlled equivalence trial
213. two prophylactic pain management regimens for medical abortion ≤63 days' gestation with mifepristone and misoprostol a multicenter, randomized, placebocontrolled trial
214. twopill regimens of misoprostol after mifepristone medical abortion through 63 days' lmp a randomized controlled trial of buccal and sublingual misoprostol
215. ultrasonographic features of the endometrium following successful medical termination of early pregnancy
216. ultrasound evaluation of the endometrium after medical termination of pregnancy
217. use of mifepristone and sublingual misoprostol for early medical abortion
218. usercentred design and development of a patient decision aid for choice of first trimester abortion method
219. using oral and intravaginal misoprostol for medical abortion
220. vaginal misoprostol administered 1, 2, or 3 days after mifepristone for early medical abortion a randomized trial
221. vaginal misoprostol alone for medical abortion up to 9 weeks of gestation efficacy and acceptability
222. vaginal misoprostol for salvage therapy after failed medical abortion
223. who multinational study of three misoprostol regimens after mifepristone for early medical abortion i efficacy
224. women's evaluation of three early abortion methods

**5. Articles excluded (n=215): No related data (n=152), Low quality studies (n=24), Absence of control group (n=18), Studies on second-trimester medical abortions (n=17), Insufficient data (n=4)**

**5.1 Articles excluded: No related data (n=152)**

1. 10 year evaluation of the use of medical abortion through telemedicine a retrospective cohort study
2. 2017 19 governmental decisions to allow home use of misoprostol for early medical abortion in the uk
3. a clinical evaluation of the safety and efficacy of mefepristone and misoprostol in medical abortions
4. a comparison of misoprostol with and without methylergometrine and oxytocin in outpatient medical abortion a phase iii randomized controlled trial
5. a comparison of the costs of manual vacuum aspiration (mva) and evacuation and curettage (e and c) in the treatment of early incomplete abortions in kenya
6. a comparison of the safety and efficacy of three medical abortion protocols
7. a comparison of the safety and efficacy of two medical abortion protocols
8. a phase iii multicentre study on medical termination of early pregnancy with two regimens of mifepristone followed by pg05
9. a prospective randomized, doubleblinded, placebocontrolled trial comparing mifepristone and vaginal misoprostol to vaginal misoprostol alone for elective termination of early pregnancy
10. a prospective, comparative study of clinical outcomes following clinicbased versus selfuse of medical abortion
11. a twopill sublingual misoprostol outpatient regimen following mifepristone for medical abortion through 70 days' lmp a prospective comparative openlabel trial
12. a viable alternative to surgical vacuum aspiration repeated doses of intravaginal misoprostol over 9 hours for medical termination of pregnancies up to eight weeks
13. abortion in four asian countries patient characteristics, morbidity and contraceptive acceptance
14. abortion in the first trimester guideline of the dggg (s2klevel, awmf registry no 015094, december 2022) part 1 with recommendations on care structures, information and advice on decisionmaking, measures before abortion and medical abortion
15. abortion policy and womens health in developingcountries
16. acceptability and feasibility of early pregnancy termination by mifepristonemisoprostol results of a large multicenter trial in the united states mifepristone clinical trials group
17. acceptability and feasibility of outpatient medical abortion with mifepristone and misoprostol up to 70 days gestation in singapore
18. adolescent girls undergoing medical abortion have lower risk of haemorrhage, incomplete evacuation or surgical evacuation than women above 18 years old
19. association between uterine position and transvaginal misoprostol treatment for early pregnancy failure
20. association of the g2014g genotype in estrogen receptor 1 gene with failure of the mifepristoneinduced termination of early pregnancy
21. bleeding patterns after vaginal misoprostol for treatment of early pregnancy failure
22. can women accurately assess the outcome of medical abortion based on symptoms alone?
23. change in serum betahuman chorionic gonadotropin after abortion with methotrexate and misoprostol
24. change in serum βhuman chorionic gonadotropin after abortion with methotrexate and misoprostol
25. clinical followup compared with selfassessment of outcome after medical abortion a multicentre, noninferiority, randomised, controlled trial
26. clinical study of early pregnancy termination by administration of dl15methyl prostaglandin f2 alpha combined with tamoxifen or norethisterone
27. clinical study of terminating biochemical pregnancy and early clinical pregnancy with mifepristone and misoprostol
28. clinical study of termination of early pregnancy by combination of dl15methylprostaglandin f2 alpha and ru 486
29. clinical trial on termination of early pregnancy with ru486 in combination with prostaglandin
30. clinical use and safety of medical method of first trimester abortion
31. clinicaltrial on termination of earlypregnancy with ru486 in combination with prostaglandin
32. comparative study of efficacy and acceptability of two regimens for 1st trimester mtp
33. comparative study of regimen mifepristone and misoprostol with misoprostol alone at first trimester termination of pregnancy
34. comparing the effects of misoprostol/letrozole and misoprostol/placebo on medical abortion success rate a randomized clinical trial
35. comparison of misoprostolonly and combined mifepristonemisoprostol regimens for homebased early medical abortion in tunisia and vietnam
36. comparison of sublingual, oral and vaginal misopristol for cervical ripening 12 hours before hysteroscopy
37. comparitive study of early (24 hours) versus late (48 hours) misoprostol administration after mifepristone for termination of early pregnancy
38. consistency of medical abortion efficacy from 5 through 14 weeks' gestation
39. declining risk of surgical intervention following early medical abortion a time trend analysis
40. depot medroxyprogesterone acetate (dmpa) administration at the time of mifepristone for medical abortion a pilot study
41. does followup with serum hcg simplify medical abortion? a randomized controlled trial
42. does methotrexate confer a significant advantage over misoprostol alone for early medical abortion? a retrospective analysis of 8678 abortions
43. doubleblind randomized trial of mifepristone in combination with vaginal gemeprost or misoprostol for induction of abortion up to 63 days gestation
44. early abortion with buccal versus sublingual misoprostol alone a multicenter, randomized trial
45. early medical abortion using lowdose mifepristone followed by buccal misoprostol a large australian observational study
46. early medical abortion with selfadministered lowdose mifepristone in combination with misoprostol
47. early versus late misoprostol administration after mifepristone for medical abortion
48. effectiveness and acceptability of home use of misoprostol for medical abortion up to 10 weeks of pregnancy
49. effectiveness and safety of early medication abortion provided in pharmacies by auxiliary nursemidwives a noninferiority study in nepal
50. effectiveness of early medical abortion using lowdose mifepristone and buccal misoprostol in women with no defined intrauterine gestational sac
51. effectiveness of selfmanaged medication abortion between 9 and 16 weeks of gestation
52. efficacy and acceptability of a mifepristonemisoprostol combined regimen for early induced abortion among women in mexico city
53. efficacy and safety of mifepristone and buccal misoprostol versus buccal misoprostol alone for medical abortion
54. efficacy and safety of mifepristonebuccal misoprostol for early medical abortion in an australian clinical setting
55. efficacy of combination therapy with methotrexate and misoprostol in termination of pregnancy in the first trimester
56. efficacy of concurrent administration of mifepristone and misoprostol for termination of pregnancy
57. efficacy of mifepristone and misoprostol in late first trimester medical abortion, missed abortion and blighted ovum
58. efficacy of misoprostol in relation to uterine position in the treatment of early pregnancy failure
59. elevated mrna expression of pgf2 receptor splice variant 2(fpv2) in human decidua is associated with incomplete mifepristonemisoprostolinduced early medical abortion by regulation of interleukin8
60. endometrial thickness and serum betahcg as predictors of the effectiveness of oral misoprostol in early pregnancy failure
61. endometrial thickness and serum βhcg as predictors of the effectiveness of oral misoprostol in early pregnancy failure
62. endometrial thickness as predictor of the effectiveness of intracervical misoprostol in early pregnancy failure
63. endometrial thickness following medical abortion is not predictive of subsequent surgical intervention
64. evaluation of effect of letrozole prior to misoprostol in comparison with misoprostol alone in success rate of induced abortion
65. evaluation of the success of medical abortion by a plasma hcg control threshold
66. evaluation of ultrasound needs in first trimester medical abortion with misoprostol
67. expanding a woman's options to include home use of misoprostol for medical abortion up until 76 days an observational study of efficacy and safety
68. extending outpatient medical abortion services through 70 days of gestational age
69. factors affecting gestational age at termination of pregnancy
70. factors related to completeness of medical abortion with mifepristone and misoprostol
71. firsttrimester pregnancy failure
72. further acceptability evaluation of ru486 and ono 802 as abortifacient agents in a chinese population
73. high failure rates of medical termination of pregnancy after introduction to a large teaching hospital
74. highdose misoprostol as an alternative therapy after failed medical abortion
75. home selfadministration of misoprostol for medical abortion up to 56 days' gestation
76. ibuprofen and paracetamol for pain relief during medical abortion a doubleblind randomized controlled study
77. importance of a second dose of misoprostol in the management of medical abortion after failure of the initial treatment
78. increasing women's choices in medical abortion a study of misoprostol 400 microg swallowed immediately or held sublingually following 200 mg mifepristone
79. induced abortion incidence and trends worldwide from 1995 to 2008
80. initiating intramuscular depot medroxyprogesterone acetate 2448 hours after mifepristone administration does not affect success of early medical abortion
81. is mifepristone 100mg an effective alternative to standard dose for medical abortion
82. lowdose mifepristone followed by vaginal misoprostol at 48 hours for abortion up to 63 days
83. lowering the doses of mifepristone and gameprost for early abortion a randomised controlled trial world health organization task force on postovulatory methods for fertility regulation
84. medical abortion a life saving technology
85. medical abortion a practical quick reference algorithm to support the gynecologists
86. medical abortion after 9 weeks
87. medical abortion at 57 to 63 days' gestation with a lower dose of mifepristone and gemeprost a randomized controlled trial
88. medical abortion at 63 to 90 days of gestation
89. medical abortion at 912 weeks
90. medical abortion defining success and categorizing failures
91. medical abortion followup with serum human chorionic gonadotropin compared with ultrasonography a randomized controlled trial
92. medical abortion in china
93. medical abortion with methotrexate 75 mg intramuscularly and vaginal misoprostol
94. medical abortion with mifepristone and home administration of misoprostol up to 63 days' gestation
95. medical abortion with mifepristone and vaginal misoprostol between 64 and 70 days' gestation
96. medical treatment with misoprostol for early failure of pregnancies after assisted reproductive technology a promising treatment option
97. methotrexate and misoprostol for early abortion in adolescent women
98. mifepristone 100mg for early medical abortion
99. mifepristone followed by home administration of buccal misoprostol for medical abortion up to 70 days of amenorrhoea in a general practice in curacao
100. minimal effective dose of mifepristone for medical abortion
101. misoprostol abortion ultrasonography versus betahcg testing for verification of effectiveness
102. misoprostol alonea new method of medical abortion?
103. misoprostol as the primary agent for medical abortion in a lowincome urban setting
104. monitoring medical abortion using mifepristone/misoprostol combination with ultrasonogram and serum human chorionic gonadotropin
105. multicenter trial of a simplified mifepristone medical abortion regimen
106. options in making use of pregnancy history in planning and analyzing studies of reproductive failure
107. prediction of late failure after medical abortion from serial betahcg measurements and ultrasonography
108. predictive value of peripheral blood α1acid glycoprotein in medical abortion outcomes with mifepristone and relativity of concentration
109. preliminary results of the role of semiquantitative pregnancy tests in medical abortion provision
110. randomized trial of oral versus sublingual misoprostol 24 h after mifepristone for medical abortion
111. randomized trial of oral versus vaginal misoprostol 2 days after mifepristone 200 mg for abortion up to 63 days of pregnancy
112. randomized trial of oral versus vaginal misoprostol at one day after mifepristone for early medical abortion
113. randomized, doubleblind, controlled trial of mifepristone in capsule versus tablet form followed by misoprostol for early medical abortion
114. reducing the buccal dose of misoprostol in mifepristone medical abortion up to 63 days lmp
115. role of routine ultrasonography in monitoring the outcome of medical abortion in a clinical setting
116. role of ultrasonography in evaluation of bleeding per vagina in first trimester of pregnancy
117. role of ultrasonography in the evaluation of causes of bleeding per vaginum in the first trimester of pregnancy
118. ru 486 (mifepristone) from clinicaltrials to the perspectives for clinical use
119. selfadministration of abortion pills and its maternal outcome in tertiary care center
120. selfassessment of medical abortion outcome using symptoms and home pregnancy testing
121. serum biomarkers may help predict successful misoprostol management of early pregnancy failure
122. serum βhcg concentration is a predictive factor for successful early medical abortion with vaginal misoprostol within 24 hours
123. sonographic patterns of the endometrium in assessment of medical abortion outcomes
124. sonographic quantification of endometrial changes after abortion with computerassisted image analysis
125. tamoxifen compared to methotrexate when used with misoprostol for abortion
126. termination of 2nd trimester pregnancy with sulprostone and mifepristone a randomized doubleblind placebocontrolled trial
127. termination of early pregnancy by two regimens of mifepristone with misoprostol a multicentre clinical trial
128. termination of early pregnancy by two regimens of mifepristone with misoprostol and mifepristone with pg05 a multicentre randomized clinical trial in china
129. termination of early pregnancy using flexible, lowdose mifepristonemisoprostol regimens
130. termination of early pregnancy using ru 486 alone or in combination with prostaglandin the ru 486 collaboration group
131. termination of earlypregnancy by 2 regimens of mifepristone with misoprostol and mifepristone with pg05 a multicenter randomized clinicaltrial in china
132. termination of pregnancy at very early gestation without visible yolk sac on ultrasound
133. the effect of misoprostol with and without letrozole in inducing successful medical abortion
134. the effect of nonsteroidal antiinflammatory drugs on medical abortion with mifepristone and misoprostol at 1322 weeks gestation
135. the effectiveness of home medical abortions provided through telemedicine
136. the efficacy and acceptability of mifepristone medical abortion with home administration misoprostol provided by private providers linked with the hospital a prospective study of 433 patients
137. the extended gestational age medical abortion study the effectiveness of medical abortion with mifepristone and misoprostol at 5763 days versus 6470 days gestation
138. the feasibility of early second trimester pregnancy (12 to 14 weeks) termination with single intra‐amniotic injection of 15‐methyl pgf2α
139. the induction of abortion in the 2nd trimester by combined administration of minprostin and sulproston, compared with the use of sulproston alone
140. the induction of abortion with prostaglandins
141. the medical management of incomplete abortion
142. trend of serum betahuman chorionic gonadotropin levels after medical abortion in the early first trimester of pregnancy
143. trends in elective medical and surgical abortions in the united states during the covid19 pandemic
144. two mifepristone doses and two intervals of misoprostol administration for termination of early pregnancy a randomised factorial controlled equivalence trial
145. two prophylactic pain management regimens for medical abortion ≤63 days' gestation with mifepristone and misoprostol a multicenter, randomized, placebocontrolled trial
146. twopill regimens of misoprostol after mifepristone medical abortion through 63 days' lmp a randomized controlled trial of buccal and sublingual misoprostol
147. ultrasonographic features of the endometrium following successful medical termination of early pregnancy
148. ultrasound evaluation of the endometrium after medical termination of pregnancy
149. usercentred design and development of a patient decision aid for choice of first trimester abortion method
150. vaginal misoprostol alone for medical abortion up to 9 weeks of gestation efficacy and acceptability
151. vaginal misoprostol for salvage therapy after failed medical abortion
152. women's evaluation of three early abortion methods

**5.2 Articles excluded: Low quality studies (n=24)**

1. a comparitive study of use of intravaginal misoprostol after 48 hours and 4 hours of oral mifepristone for medical termination of pregnancy
2. a noninferiority study of outpatient mifepristonemisoprostol medical abortion at 6470 days and 7177 days of gestation
3. comparative study of mifepristone with vaginal misoprostol for first trimester termination of pregnancy at different gestational ages
4. comparison of two doses of mifepristone in combination with misoprostol for early medical abortion a randomised trial
5. efficacy of single dose vaginal misoprostol 800μg in i trimester abortion
6. induction of abortion with mifepristone and misoprostol in early pregnancy
7. medical abortion in women of ≤56 days amenorrhoea a comparison between gemeprost (a pge(1) analogue) alone and mifepristone and gemeprost
8. methotrexate and misoprostol used alone or in combination for early abortion
9. methotrexate and misoprostol vs misoprostol alone for early abortion a randomized controlled trial
10. mifepristone and misoprostol administered simultaneously versus 24 hours apart for abortion a randomized controlled trial
11. mifepristone plus vaginal misoprostol vs vaginal misoprostol alone for medical abortion in gestation 63 days or less in nepalese women a quasirandomized controlled trial
12. misoprostol dose and route after mifepristone for early medical abortion a randomised controlled noninferiority trial
13. outcome of first trimester induced abortions using misoprostol by buccal and vaginal routes
14. pregnancy the effect of dose of mifepristone and gestation on the efficacy of medical abortion with mifepristone and misoprostol
15. randomised controlled trial comparing efficacy of same day administration of mifepristone and misoprostol for termination of pregnancy with the standard 36 to 48hour protocol
16. randomised controlled trial comparing the efficacy of sameday administration of mifepristone and misoprostol for termination of pregnancy with the standard 36 to 48 hour protocol
17. randomized trial of mifepristone and buccal misoprostol vs misoprostol alone for medical abortion
18. randomized trial of mifepristone and buccal or vaginal misoprostol for abortion through 56 days of last menstrual period
19. retrospective assessment of the influence of gestational age on the first trimester medical abortion efficiency
20. risk factors for unsuccessful medical abortion with mifepristone and misoprostol
21. sublingual v/s vaginal misoprostol for second trimester termination of pregnancy a comparative study
22. the effect of dose of mifepristone and gestation on the efficacy of medical abortion with mifepristone and misoprostol
23. vaginal misoprostol administered 1, 2, or 3 days after mifepristone for early medical abortion a randomized trial
24. who multinational study of three misoprostol regimens after mifepristone for early medical abortion i efficacy

**5.3 Articles excluded: Absence of control group (n=18)**

1. 200 mg mifepristone was as effective as 600 mg when used with oral misoprostol for early medical abortion
2. a comparison of surgical vacuum aspiration abortion with medical abortion using mifepristone (ru 486) and gemeprost implications for nursing staff
3. a comparison of the efficacy of sublingual and oral misoprostol 400 microgram in the management of early pregnancy failure a randomized controlled trial
4. a pilot study of mifepristone and misoprostol administered at the same time for abortion up to 49 days gestation
5. a prospective trial using mifepristone and vaginal misoprostol in termination of pregnancies up to 63 days of gestation
6. efficacy of misoprostol administration 24 hours after mifepristone for termination of early pregnancy
7. efficacy of single dose of mifepristone combined with two doses of misoprostol in early medical abortions
8. experiences with gemeprost in 1sttrimester termination of pregnancy
9. experiences with the progesterone antagonist mifepristone (ru 486) for termination of early pregnancy
10. first trimester abortion with mifepristone and vaginal misoprostol
11. induction of abortion in earlypregnancy with mifepristone in conjunction with gemeprost
12. lowering the doses of mifepristone and gemeprost for early abortion a randomised controlled trial
13. oral methotrexate and vaginal misoprostol for early abortion
14. outcomes during early implementation of mifepristonebuccal misoprostol abortions up to 63 days of gestation in a canadian clinical setting
15. outpatient mifepristonemisoprostol medical abortion through 77 days of gestation
16. simultaneous administration of mifepristone and misoprostol for early termination of pregnancy a randomized controlled trial
17. use of mifepristone and sublingual misoprostol for early medical abortion
18. using oral and intravaginal misoprostol for medical abortion

**5.4 Articles excluded: Studies on second-trimester medical abortions (n=17)**

1. 346 efficacy and safety of mifepristone and sublingual misoprostol for second trimester medical abortion a prospective study
2. a comparative study on sublingual versus oral and vaginal administration of misoprostol for late first and early second trimester abortion
3. a comparison of misoprostol with and without laminaria tents for induction of secondtrimester abortion
4. comparison of the efficacy and safety of two combined misoprostol regimens for second trimester medical abortion
5. comparison of vaginal and sublingual misoprostol for second trimester abortion randomized controlled equivalence trial
6. efficacy and safety of intravaginal misoprostol for midtrimester medical termination of pregnancy
7. factors influencing the abortion interval of second trimester pregnancy termination using misoprostol
8. induction of abortion during the 2nd trimenon of pregnancy endocervical pge2 gel application, intramuscular application of sulproston and combined treatment (endocervical pge2 gel intramuscular sulproston)
9. issues in second trimester induced abortion (medical/surgical methods)
10. medical abortion in the second trimester an update
11. medical abortion use of mifepristone and misoprostol in first and second trimesters of pregnancy
12. medical termination of pregnancy during the second versus the first trimester and its effects on subsequent pregnancy
13. misoprostol for second trimester medical abortion a comparison of three routes of administration
14. oral misoprostol administration in the first and second trimester for termination
15. risk of surgical evacuation and risk of major surgery following secondtrimester medical abortion in denmark a nationwide cohort study
16. safe medical abortion in the second trimester of pregnancy
17. therapeutic termination of second trimester pregnancies with low dose misoprostol

**5.5 Articles excluded: Insufficient data (n=4)**

1. home selfadministration of vaginal misoprostol for medical abortion at 50 to 63 days compared with gestation of below 50 days
2. medical abortion using methotrexate and misoprostol efficacy and tolerability
3. placenta accreta a cause of failed medical abortion
4. surgical intervention after medical abortion

**6. Studies included in quality assessment (n=9)**

1. Effect of previous live birth and prior route of delivery on the outcome of early medical abortion
2. Risk factors for surgical intervention of early medical abortion
3. Comparison of rates of adverse events in adolescent and adult women undergoing medical abortion: population register based study
4. Factors affecting the outcome of early medical abortion: a review of 4132 consecutive cases
5. Parity is a major determinant of success rate in medical abortion: a retrospective analysis of 3161 consecutive cases of early medical abortion treated with reduced doses of mifepristone and vaginal gemeprost
6. Failure Rate of Medical Treatment for Miscarriage Correlated with the Difference between Gestational Age According to Last Menstrual Period and Gestational Size Calculated via Ultrasound
7. The role of parity in medical abortion up to 49 days of amenorrhoea
8. Increased risk for medical abortion failure for multiparous women
9. Predictors of uterine evacuation following early medical abortion with mifepristone and misoprostol

**7. Studies included in the systematic review and meta-analysis (n=9)**

1.	Effect of previous live birth and prior route of delivery on the outcome of early medical abortion

2.	Risk factors for surgical intervention of early medical abortion

3.	Comparison of rates of adverse events in adolescent and adult women undergoing medical abortion: population register based study

4.	Factors affecting the outcome of early medical abortion: a review of 4132 consecutive cases

5.	Parity is a major determinant of success rate in medical abortion: a retrospective analysis of 3161 consecutive cases of early medical abortion treated with reduced doses of mifepristone and vaginal gemeprost

6.	Failure Rate of Medical Treatment for Miscarriage Correlated with the Difference between Gestational Age According to Last Menstrual Period and Gestational Size Calculated via Ultrasound

7.	The role of parity in medical abortion up to 49 days of amenorrhoea

8.	Increased risk for medical abortion failure for multiparous women

9.	Predictors of uterine evacuation following early medical abortion with mifepristone and misoprostol
