# Supplementary material for: Development and validation of a risk assessment model for predicting the failure of early medical abortions: A clinical prediction model study based on a systematic review and meta-analysis
Source: PLoS One. 2024 Dec 20;19(12):e0315025. doi: 10.1371/journal.pone.0315025 (PMC11661585; doi:10.1371/journal.pone.0315025)
Supplement: S3 Appendix — (DOCX) [file pone.0315025.s006.docx]

**S3 Appendix. The inclusion criteria, exclusion criteria, data extraction, and quality assessment of systematic review.**

**1. Inclusion criteria**

We included studies that met the following criteria: (1) case-control studies, cohort studies, and randomized controlled trials; (2) studies comparing the early medical abortion (EMA) failure rates of patients with risk factors versus those without risk factors; (3) studies reporting outcomes (success or failure) of EMAs; and (4) sample size of at least 20 patients in each group.

**2. Exclusion criteria**

We excluded studies that met the following criteria: (1) case reports, case series, reviews, and in vivo/vitro studies; (2) studies lacking a control group; (3) studies without available data on outcomes (success or failure) of EMAs; (4) duplicate studies.

**3. Data extraction**

The data from eligible studies were independently extracted by two authors (AHL and XWL) and organized into a two-by-two table. Any discrepancies were resolved through discussions with another author (YWY). The baseline information collected included the author's name, region, period, design, number of medical abortions, number and proportion of failed medical abortions, procedure of medical abortions (dose and route), gestational age, and maternal age in each group.

**4. Quality assessment**

The quality of the observational studies was assessed using the Newcastle-Ottawa Scale (NOS) [1]. A star rating system of items in the NOS was utilized to evaluate each study. An observational study was considered to be of high quality when it received a rating of at least 6 stars [2].

**References**

[1] Ottawa Hospital Research Institute 2021. The Newcastle-Ottawa Scale (NOS) for assessing the quality of nonrandomized studies in meta-analyses. 2024. Available from: https://www.ohri.ca/programs/clinical_epidemiology/oxford.asp

[2] Duan J, Ma D, Wen X, Guo Q, Gao J, Zhang G, et al. Hydroxychloroquine prophylaxis for preeclampsia, hypertension and prematurity in pregnant patients with systemic lupus erythematosus: A meta-analysis. Lupus. 2021;30:1163–1174. https://doi.org/10.1177/09612033211007199 PMID: 33853420
