# Supplementary material for: Development and validation of a risk assessment model for predicting the failure of early medical abortions: A clinical prediction model study based on a systematic review and meta-analysis
Source: PLoS One. 2024 Dec 20;19(12):e0315025. doi: 10.1371/journal.pone.0315025 (PMC11661585; doi:10.1371/journal.pone.0315025)
Supplement: S4 Appendix — (DOCX) [file pone.0315025.s007.docx]

**S4 Appendix. All data extracted from the primary research sources for the systematic review and meta-analysis.**

All the data were extracted from the primary research sources (Medline, Embase, Scopus, Web of Science, and Cochrane Library) for the systematic review and meta-analysis. There were no data or supporting information obtained from another source.

| **S4 Appendix-1. Name of data extractors and date of data extraction.** | | |
| --- | --- | --- |
| **Study** | **Name of data extractors** | **Date of data extraction** |
| Ashok 2002 [14] | An-Hao Liu | January 19th, 2024 |
| Bartley 2000 [15] | An-Hao Liu | January 19th, 2024 |
| Chien 2009 [11] | An-Hao Liu | January 19th, 2024 |
| Gluck 2023 [16] | An-Hao Liu | January 19th, 2024 |
| Lefebvre 2008 [17] | An-Hao Liu | January 19th, 2024 |
| Meaidi 2019 [12] | An-Hao Liu | January 19th, 2024 |
| Niinimaki 2004 [18] | An-Hao Liu | January 19th, 2024 |
| Niinimaki 2011 [13] | An-Hao Liu | January 19th, 2024 |
| Reeves 2015 [19] | An-Hao Liu | January 19th, 2024 |

| **S4 Appendix-2. The confirmation of the eligibility of nine included studies (inclusion criteria).** | | | | | | |
| --- | --- | --- | --- | --- | --- | --- |
| **Study** | **Study design** | **Did this study compare EMA failure rates of patients with risk factors versus those without risk factors?** | | **Did this study report outcomes of EMAs?** | **Did this study have a sample size of at least 20 patients in each group?** | |
| Ashok 2002 [14] | Prospective cohort study | Yes |  | Yes | Yes |  |
| Bartley 2000 [15] | Retrospective cohort study | Yes |  | Yes | Yes |  |
| Chien 2009 [11] | Retrospective cohort study | Yes |  | Yes | Yes |  |
| Gluck 2023 [16] | Retrospective cohort study | Yes |  | Yes | Yes |  |
| Lefebvre 2008 [17] | Retrospective cohort study | Yes |  | Yes | Yes |  |
| Meaidi 2019 [12] | Retrospective cohort study | Yes |  | Yes | Yes |  |
| Niinimaki 2004 [18] | Retrospective cohort study | Yes |  | Yes | Yes |  |
| Niinimaki 2011 [13] | Retrospective cohort study | Yes |  | Yes | Yes |  |
| Reeves 2015 [19] | Prospective cohort study | Yes |  | Yes | Yes |  |
| The inclusion criteria: (1) case-control studies, cohort studies, and randomized controlled trials; (2) studies comparing the early medical abortion (EMA) failure rates of patients with risk factors versus those without risk factors; (3) studies reporting outcomes (success or failure) of EMAs; and (4) sample size of at least 20 patients in each group. | | | | | | |

| **S4 Appendix-2. The confirmation of the eligibility of nine included studies (exclusion criteria).** | | | | | | |
| --- | --- | --- | --- | --- | --- | --- |
| **Study** | **Study design** | **Did this study lack a control group?** | | **Is this study without available data on outcomes of EMAs?** | **Is this study a duplicate study?** | |
| Ashok 2002 [14] | Prospective cohort study | No |  | No | No |  |
| Bartley 2000 [15] | Retrospective cohort study | No |  | No | No |  |
| Chien 2009 [11] | Retrospective cohort study | No |  | No | No |  |
| Gluck 2023 [16] | Retrospective cohort study | No |  | No | No |  |
| Lefebvre 2008 [17] | Retrospective cohort study | No |  | No | No |  |
| Meaidi 2019 [12] | Retrospective cohort study | No |  | No | No |  |
| Niinimaki 2004 [18] | Retrospective cohort study | No |  | No | No |  |
| Niinimaki 2011 [13] | Retrospective cohort study | No |  | No | No |  |
| Reeves 2015 [19] | Prospective cohort study | No |  | No | No |  |
| The exclusion criteria: (1) case reports, case series, reviews, and in vivo/vitro studies; (2) studies lacking a control group; (3) studies without available data on outcomes (success or failure) of EMAs; (4) duplicate studies. | | | | | | |

| **S4 Appendix-3. The quality assessment of the included studies based on the Newcastle-Ottawa scale.** | | | | | | | | | |
| --- | --- | --- | --- | --- | --- | --- | --- | --- | --- |
| **Study/Assessment** | **Item 1** | **Item 2** | **Item 3** | **Item 4** | **Item 5** | **Item 6** | **Item 7** | **Item 8** | **Total scores** |
| ▲/△ | A* | B | C | D | E | F | G | H | NOS scores for cohort studies |
| Ashok 2002▲ | ★ | ★ | ★ | ☆ | ★☆ | ★ | ★ | ★ | **7^†^** |
| Bartley 2000△ | ★ | ★ | ★ | ☆ | ★☆ | ★ | ★ | ★ | **7^†^** |
| Chien 2009△ | ★ | ★ | ★ | ☆ | ★★ | ★ | ★ | ★ | **8^†^** |
| Gluck 2023△ | ★ | ★ | ★ | ☆ | ★★ | ★ | ★ | ★ | **8^†^** |
| Lefebvre 2008△ | ★ | ★ | ★ | ☆ | ★☆ | ★ | ★ | ★ | **7^†^** |
| Meaidi 2019△ | ★ | ★ | ★ | ☆ | ★★ | ★ | ★ | ★ | **8^†^** |
| Niinimaki 2004△ | ★ | ★ | ★ | ☆ | ★★ | ★ | ★ | ★ | **8^†^** |
| Niinimaki 2011△ | ★ | ★ | ★ | ☆ | ★★ | ★ | ★ | ★ | **8^†^** |
| Reeves 2015▲ | ★ | ★ | ★ | ☆ | ★★ | ★ | ★ | ★ | **8^†^** |
| *Items of quality assessment methods. A, representativeness of the exposed cohort. B, selection of the non-exposed cohort. C, ascertainment of exposure. D, demonstration that outcome of interest was not present at start of study. E, comparability of cohorts on the basis of the design or analysis. F, assessment of outcome. G, was follow-up long enough for outcomes to occur. H, adequacy of follow up of cohorts. NOS, the Newcastle-Ottawa scale. △, retrospective cohort study. ▲, prospective cohort study. ★, was awarded when the respective information was available. ☆, was awarded when the respective information was unavailable. †Studies receiving 6 stars or more were considered to be of high quality in NOS. | | | | | | | | | |

| **S4 Appendix-4. All data extracted from each study for the reported systematic review and meta-analysis.** | | | | | | | |
| --- | --- | --- | --- | --- | --- | --- | --- |
| **Study** | **No. of medical**  **abortions** | **No. of failed**  **abortions (%)** | **Selected risk factors** | **Patients with PT≥1 (No. of failed/total medical abortions)** |  | **Patients with PT=0 (No. of failed/total medical abortions)** |  |
| Ashok 2002 [14] | 4131 | 94 (2.3) | PT | 38/1045 |  | 53/3019 |  |
| Bartley 2000 [15] | 2839 | 102 (3.6) | PA, PT | 31/580 |  | 70/2224 |  |
| Chien 2009 [11] | 879 | 82 (9.3) | PA | — |  | — |  |
| Gluck 2023 [16] | 778 | 196 (25.1) | LU | — |  | — |  |
| Lefebvre 2008 [17] | 1850 | 54 (2.9) | PA | — |  | — |  |
| Meaidi 2019 [12] | 86437 | 5320 (6.2) | GA, MA, VD, CS, MR, PM, PS | — |  | — |  |
| Niinimaki 2004 [18] | 316 | 29 (9.2) | PA | — |  | — |  |
| Niinimaki 2011 [13] | 27030 | 1447 (5.4) | TR, MS, GA | — |  | — |  |
| Reeves 2015 [19] | 2160 | 75 (3.5) | PA, PT | 38/996 |  | 37/1153 |  |
| PT, previous termination of pregnancy. PA, parity. VD, only vaginal deliveries and spontaneous delivery of placenta. CS, ≥1 caesarean section. MR, ≥1 manual removal of placenta. GA, gestational age. MA, maternal age. PM, previous medical abortions. PS, previous surgical abortions. MS, marital status. TR, type of residence. LU, differences between gestational age calculated using the last menstrual period and gestational age calculated via ultrasound. | | | | | | | |

| **S4 Appendix-4. All data extracted from each study for the reported systematic review and meta-analysis.** | | | | | | | |
| --- | --- | --- | --- | --- | --- | --- | --- |
| **Study** | **Patients with PA≥1 (No. of failed/total medical abortions)** | **Patients with PA=0 (No. of failed/total medical abortions)** | **The OR value of LU≥1** | **The OR value of MC** |  | **The OR value of RU** |  |
| Ashok 2002 [14] | — | — | — | — |  | — |  |
| Bartley 2000 [15] | 70/1277 | 29/1383 | — | — |  | — |  |
| Chien 2009 [11] | 68/523 | 14/356 | — | — |  | — |  |
| Gluck 2023 [16] | — | — | 1.24 (95%CI 1.01-1.51) | — |  | — |  |
| Lefebvre 2008 [17] | 42/977 | 12/873 | — | — |  | — |  |
| Meaidi 2019 [12] | — | — | — | — |  | — |  |
| Niinimaki 2004 [18] | 19/149 | 10/167 | — | — |  | — |  |
| Niinimaki 2011 [13] | — | — | — | 1.22 (95%CI 1.04-1.42) |  | 1.39 (95%CI 1.16-1.68) |  |
| Reeves 2015 [19] | 55/1334 | 20/826 | — | — |  | — |  |
| EMA, early medical abortion. OR, odds ratio. GA7, gestational age of 42–48 days. GA8, gestational age of 49–55 days. GA9, gestational age of 56–62 days. GA12, gestational age of 9–12 weeks. MA1, maternal age of 20–24 years. MA2, maternal age of 25–29 years. MA3, maternal age of 30–34 years. MA4, maternal age of 35–39 years. MA5, maternal age of 40–49 years. PA, parity. VD, only vaginal deliveries and spontaneous delivery of placenta. CS, ≥1 caesarean section. MR, ≥1 manual removal of placenta. PT, previous termination of pregnancy. PM, previous medical abortions. PS1, ≥1 previous surgical abortion: ≥56 days of gestation. PS2, ≥1 previous surgical abortion: <56 days of gestation. PS3, ≥2 previous surgical abortion: both <56 and ≥56 days of gestation. MC, married or cohabiting. RU, rural areas. LU, differences between gestational age calculated using the last menstrual period and gestational age calculated via ultrasound. | | | | | | | |

| **S4 Appendix-4. All data extracted from each study for the reported systematic review and meta-analysis.** | | | | | | | |
| --- | --- | --- | --- | --- | --- | --- | --- |
| **Study** | **The OR value of GA7** | **The OR value of GA8** | **The OR value of GA9** | **The OR value of GA12** |  | **The OR value of MA1** |  |
| Ashok 2002 [14] | — | — | — | — |  | — |  |
| Bartley 2000 [15] | — | — | — | — |  | — |  |
| Chien 2009 [11] | — | — | — | — |  | — |  |
| Gluck 2023 [16] | — | — | — | — |  | — |  |
| Lefebvre 2008 [17] | — | — | — | — |  | — |  |
| Meaidi 2019 [12] | 1.67 (95%CI 1.53-1.82) | 2.35 (95%CI 2.15-2.57) | 3.22 (95%CI 2.92-3.55) | 6.47 (95%CI 5.06-8.26) |  | 1.31 (95%CI 1.17-1.47) |  |
| Niinimaki 2004 [18] | — | — | — | — |  | — |  |
| Niinimaki 2011 [13] | — | — | — | — |  | — |  |
| Reeves 2015 [19] | — | — | — | — |  | — |  |
| EMA, early medical abortion. OR, odds ratio. GA7, gestational age of 42–48 days. GA8, gestational age of 49–55 days. GA9, gestational age of 56–62 days. GA12, gestational age of 9–12 weeks. MA1, maternal age of 20–24 years. MA2, maternal age of 25–29 years. MA3, maternal age of 30–34 years. MA4, maternal age of 35–39 years. MA5, maternal age of 40–49 years. PA, parity. VD, only vaginal deliveries and spontaneous delivery of placenta. CS, ≥1 caesarean section. MR, ≥1 manual removal of placenta. PT, previous termination of pregnancy. PM, previous medical abortions. PS1, ≥1 previous surgical abortion: ≥56 days of gestation. PS2, ≥1 previous surgical abortion: <56 days of gestation. PS3, ≥2 previous surgical abortion: both <56 and ≥56 days of gestation. MC, married or cohabiting. RU, rural areas. LU, differences between gestational age calculated using the last menstrual period and gestational age calculated via ultrasound. | | | | | | | |

| **S4 Appendix-4. All data extracted from each study for the reported systematic review and meta-analysis.** | | | | | | | |
| --- | --- | --- | --- | --- | --- | --- | --- |
| **Study** | **The OR value of MA2** | **The OR value of MA3** | **The OR value of MA4** | **The OR value of MA5** |  | **The OR value of VD** |  |
| Ashok 2002 [14] | — | — | — | — |  | — |  |
| Bartley 2000 [15] | — | — | — | — |  | — |  |
| Chien 2009 [11] | — | — | — | — |  | — |  |
| Gluck 2023 [16] | — | — | — | — |  | — |  |
| Lefebvre 2008 [17] | — | — | — | — |  | — |  |
| Meaidi 2019 [12] | 1.52 (95%CI 1.35-1.71) | 1.67 (95%CI 1.48-1.89) | 1.47 (95%CI 1.30-1.67) | 1.16 (95%CI 1.00-1.35) |  | 1.08 (95%CI 1.00-1.61) |  |
| Niinimaki 2004 [18] | — | — | — | — |  | — |  |
| Niinimaki 2011 [13] | — | — | — | — |  | — |  |
| Reeves 2015 [19] | — | — | — | — |  | — |  |
| EMA, early medical abortion. OR, odds ratio. GA7, gestational age of 42–48 days. GA8, gestational age of 49–55 days. GA9, gestational age of 56–62 days. GA12, gestational age of 9–12 weeks. MA1, maternal age of 20–24 years. MA2, maternal age of 25–29 years. MA3, maternal age of 30–34 years. MA4, maternal age of 35–39 years. MA5, maternal age of 40–49 years. PA, parity. VD, only vaginal deliveries and spontaneous delivery of placenta. CS, ≥1 caesarean section. MR, ≥1 manual removal of placenta. PT, previous termination of pregnancy. PM, previous medical abortions. PS1, ≥1 previous surgical abortion: ≥56 days of gestation. PS2, ≥1 previous surgical abortion: <56 days of gestation. PS3, ≥2 previous surgical abortion: both <56 and ≥56 days of gestation. MC, married or cohabiting. RU, rural areas. LU, differences between gestational age calculated using the last menstrual period and gestational age calculated via ultrasound. | | | | | | | |

| **S4 Appendix-4. All data extracted from each study for the reported systematic review and meta-analysis.** | | | | | | | |
| --- | --- | --- | --- | --- | --- | --- | --- |
| **Study** | **The OR value of CS** | **The OR value of MR** | **The OR value of PM** | **The OR value of PS1** | **The OR value of PS2** | **The OR value of PS3** |  |
| Ashok 2002 [14] | — | — | — | — | — | — |  |
| Bartley 2000 [15] | — | — | — | — | — | — |  |
| Chien 2009 [11] | — | — | — | — | — | — |  |
| Gluck 2023 [16] | — | — | — | — | — | — |  |
| Lefebvre 2008 [17] | — | — | — | — | — | — |  |
| Meaidi 2019 [12] | 1.48 (95%CI 1.33-1.64) | 2.16 (95%CI 1.75-2.67) | 0.84 (95%CI 0.78-0.91) | 1.17 (95%CI 1.08-1.27) | 1.53 (95%CI 1.35-1.74) | 1.64 (95%CI 1.41-1.90) |  |
| Niinimaki 2004 [18] | — | — | — | — | — | — |  |
| Niinimaki 2011 [13] | — | — | — | — | — | — |  |
| Reeves 2015 [19] | — | — | — | — | — | — |  |
| EMA, early medical abortion. OR, odds ratio. GA7, gestational age of 42–48 days. GA8, gestational age of 49–55 days. GA9, gestational age of 56–62 days. GA12, gestational age of 9–12 weeks. MA1, maternal age of 20–24 years. MA2, maternal age of 25–29 years. MA3, maternal age of 30–34 years. MA4, maternal age of 35–39 years. MA5, maternal age of 40–49 years. PA, parity. VD, only vaginal deliveries and spontaneous delivery of placenta. CS, ≥1 caesarean section. MR, ≥1 manual removal of placenta. PT, previous termination of pregnancy. PM, previous medical abortions. PS1, ≥1 previous surgical abortion: ≥56 days of gestation. PS2, ≥1 previous surgical abortion: <56 days of gestation. PS3, ≥2 previous surgical abortion: both <56 and ≥56 days of gestation. MC, married or cohabiting. RU, rural areas. LU, differences between gestational age calculated using the last menstrual period and gestational age calculated via ultrasound. | | | | | | | |
