# Supplementary material for: Development and validation of a risk assessment model for predicting the failure of early medical abortions: A clinical prediction model study based on a systematic review and meta-analysis
Source: PLoS One. 2024 Dec 20;19(12):e0315025. doi: 10.1371/journal.pone.0315025 (PMC11661585; doi:10.1371/journal.pone.0315025)
Supplement: S5 Appendix — (DOCX) [file pone.0315025.s008.docx]

| **S5 Appendix. Original data of the validation cohort patients.** | | | | | | | | | | | | | | | | | |
| --- | --- | --- | --- | --- | --- | --- | --- | --- | --- | --- | --- | --- | --- | --- | --- | --- | --- |
| Number | Name | PA* | GA | MA | PT | MS | TR | LU | PA | GA | MA | PT | MS | TR | LU | Total score | Outcome |
|  |  | (number) | (wks+days) | (yrs) | (number) |  |  | (wks) | (pts) | (pts) | (pts) | (pts) | (pts) | (pts) | (pts) | (pts) |  |
| 1 | WLP | VD(2) | 6 | 29 | 0 | MC | UB | 2 | 1 | 5 | 4 | 0 | 2 | 0 | 2 | 14 | failure |
| 2 | LXH | VD(2) | 7+3 | 34 | PM(1) | MC | UB | 0 | 1 | 9 | 5 | -2 | 2 | 0 | 0 | 15 | failure |
| 3 | HPQ | 0 | 7+3 | 30 | PS(3)PM(1) | SG | UB | 0 | 0 | 9 | 5 | 5 | 0 | 0 | 0 | 19 | failure |
| 4 | ZHY | VD(1)CS(1) | 6+5 | 28 | PS(1) | MC | RU | 0 | 4 | 5 | 4 | 5 | 2 | 3 | 0 | 23 | failure |
| 5 | ZLX | 0 | 6+2 | 22 | 0 | SG | RU | 1 | 0 | 5 | 3 | 0 | 0 | 3 | 2 | 13 | failure |
| 6 | ZCY | CS(2) | 6+2 | 34 | 0 | MC | UB | 1 | 4 | 5 | 5 | 0 | 2 | 0 | 2 | 18 | failure |
| 7 | SJR | 0 | 6 | 29 | PS(1) | MC | RU | 1 | 0 | 5 | 4 | 5 | 2 | 3 | 2 | 21 | failure |
| 8 | CEF | VD(1) | 6+2 | 32 | PS(3)PM(3) | MC | UB | 0 | 1 | 5 | 5 | 5 | 2 | 0 | 0 | 18 | failure |
| 9 | YIL | VD(3) | 6+1 | 34 | PS(1)PM(1) | MC | RU | 1 | 1 | 5 | 5 | 5 | 2 | 3 | 2 | 23 | failure |
| 10 | QBH | 0 | 6+6 | 32 | PM(2) | MC | UB | 1 | 0 | 5 | 5 | -2 | 2 | 0 | 2 | 12 | failure |
| 11 | ZXF | VD(1) | 6+2 | 23 | PS(1) | MC | UB | 0 | 1 | 5 | 3 | 5 | 2 | 0 | 0 | 16 | failure |
| 12 | YAJ | VD(1) | 6+4 | 26 | PS(1) | SG | RU | 1 | 1 | 5 | 4 | 5 | 0 | 3 | 2 | 20 | failure |
| 13 | DHL | 0 | 6 | 21 | 0 | SG | RU | 2 | 0 | 5 | 3 | 0 | 0 | 3 | 2 | 13 | failure |
| 14 | HLB | VD(2) | 6+5 | 39 | PS(1) | MC | RU | 0 | 1 | 5 | 4 | 5 | 2 | 3 | 0 | 20 | failure |
| 15 | WBR | 0 | 6 | 23 | PS(2) | SG | RU | 2 | 0 | 5 | 3 | 5 | 0 | 3 | 2 | 18 | failure |
| 16 | LCL | 0 | 6+3 | 24 | PS(1) | SG | RU | 0 | 0 | 5 | 3 | 5 | 0 | 3 | 0 | 16 | failure |
| 17 | ZHY | VD(2) | 6+2 | 35 | PS(1) | MC | UB | 0 | 1 | 5 | 4 | 5 | 2 | 0 | 0 | 17 | failure |
| 18 | YMZ | 0 | 9+3 | 18 | 0 | SG | RU | 1 | 0 | 19 | 0 | 0 | 0 | 3 | 2 | 24 | failure |
| 19 | LXZ | VD(2) | 6+1 | 41 | PS(2) | MC | UB | 0 | 1 | 5 | 1 | 5 | 2 | 0 | 0 | 14 | failure |
| 20 | HUS | VD(2) | 6 | 33 | 0 | MC | UB | 1 | 1 | 5 | 5 | 0 | 2 | 0 | 2 | 15 | failure |
| 21 | CXL | VD(2) | 5+5 | 32 | PS(1) | MC | UB | 4 | 1 | 0 | 5 | 5 | 2 | 0 | 2 | 15 | failure |
| 22 | FYJ | CS(2) | 5 | 37 | PS(1) | MC | UB | 2 | 4 | 0 | 4 | 5 | 2 | 0 | 2 | 17 | failure |
| 23 | CHM | CS(2) | 6+1 | 35 | PS(1) | MC | UB | 0 | 4 | 5 | 4 | 5 | 2 | 0 | 0 | 20 | failure |
| 24 | LYW | 0 | 5+1 | 24 | PS(2) | SG | RU | 1 | 0 | 0 | 3 | 5 | 0 | 3 | 2 | 13 | failure |
| 25 | QMJ | VD(3) | 6+6 | 34 | PS(1) | MC | UB | 0 | 1 | 5 | 5 | 5 | 2 | 0 | 0 | 18 | failure |
| 26 | ZLH | VD(2) | 6 | 35 | PS(2) | MC | RU | 2 | 1 | 5 | 4 | 5 | 2 | 3 | 2 | 22 | failure |
| 27 | CUN | 0 | 5+1 | 20 | PS(2) | SG | UB | 1 | 0 | 0 | 3 | 5 | 0 | 0 | 2 | 10 | failure |
| 28 | WJH | VD(3) | 6+2 | 34 | PS(5) | MC | UB | 1 | 1 | 5 | 5 | 5 | 2 | 0 | 2 | 20 | failure |
| 29 | HHH | 0 | 7+1 | 35 | 0 | MC | RU | 0 | 0 | 9 | 4 | 0 | 2 | 3 | 0 | 18 | failure |
| 30 | WBB | 0 | 7+1 | 27 | 0 | SG | UB | 1 | 0 | 9 | 4 | 0 | 0 | 0 | 2 | 15 | failure |
| 31 | XIQ | CS(1) | 6+2 | 28 | PS(1)PM(1) | MC | UB | 0 | 4 | 5 | 4 | 5 | 2 | 0 | 0 | 20 | failure |
| 32 | ZYH | CS(2) | 5+6 | 32 | PS(1) | MC | UB | 1 | 4 | 0 | 5 | 5 | 2 | 0 | 2 | 18 | failure |
| 33 | DUJ | CS(1) | 6+6 | 28 | 0 | MC | RU | 1 | 4 | 5 | 4 | 0 | 2 | 3 | 2 | 20 | failure |
| 34 | CYP | 0 | 6+6 | 26 | 0 | SG | UB | 1 | 0 | 5 | 4 | 0 | 0 | 0 | 2 | 11 | failure |
| 35 | GWJ | 0 | 6 | 25 | 0 | SG | RU | 3 | 0 | 5 | 4 | 0 | 0 | 3 | 2 | 14 | failure |
| 36 | YLL | CS(2) | 7+1 | 38 | PS(1)PM(1) | MC | UB | 1 | 4 | 9 | 4 | 5 | 2 | 0 | 2 | 26 | failure |
| 37 | WUM | 0 | 6+4 | 26 | 0 | SG | RU | 0 | 0 | 5 | 4 | 0 | 0 | 3 | 0 | 12 | failure |
| 38 | ZQM | 0 | 7+4 | 31 | PS(1) | SG | RU | 1 | 0 | 9 | 5 | 5 | 0 | 3 | 2 | 24 | failure |
| 39 | MDF | VD(2) | 6+3 | 33 | PS(2) | MC | UB | 0 | 1 | 5 | 5 | 5 | 2 | 0 | 0 | 18 | failure |
| 40 | LXM | 0 | 5 | 33 | 0 | SG | UB | 0 | 0 | 0 | 5 | 0 | 0 | 0 | 0 | 5 | failure |
| 41 | ZJT | 0 | 8+1 | 27 | 0 | MC | RU | 0 | 0 | 12 | 4 | 0 | 2 | 3 | 0 | 21 | failure |
| 42 | ZLX | VD(2) | 6 | 42 | PS(2)PM(1) | MC | UB | 0 | 1 | 5 | 1 | 5 | 2 | 0 | 0 | 14 | failure |
| 43 | KSL | CS(2) | 8+2 | 39 | PS(1) | MC | UB | 0 | 4 | 12 | 4 | 5 | 2 | 0 | 0 | 27 | failure |
| 44 | ZLM | VD(2) | 6+6 | 25 | PS(2) | MC | RU | 1 | 1 | 5 | 4 | 5 | 2 | 3 | 2 | 22 | failure |
| 45 | WAL | CS(1) | 6+5 | 25 | PS(3)PM(1) | MC | RU | 0 | 4 | 5 | 4 | 5 | 2 | 3 | 0 | 23 | failure |
| 46 | HSC | VD(1) | 8+1 | 31 | 0 | MC | UB | 0 | 1 | 12 | 5 | 0 | 2 | 0 | 0 | 20 | failure |
| 47 | CHM | VD(1) | 9+3 | 29 | PS(2) | MC | UB | 0 | 1 | 19 | 4 | 5 | 2 | 0 | 0 | 31 | failure |
| 48 | WJY | VD(2) | 6+1 | 26 | PS(1) | MC | RU | 1 | 1 | 5 | 4 | 5 | 2 | 3 | 2 | 22 | failure |
| 49 | HYY | 0 | 5+2 | 30 | PS(3) | MC | UB | 0 | 0 | 0 | 5 | 5 | 2 | 0 | 0 | 12 | success |
| 50 | WBB | CS(2) | 5+5 | 34 | PM(1) | MC | UB | 1 | 4 | 0 | 5 | -2 | 2 | 0 | 2 | 11 | success |
| 51 | LSJ | VD(2) | 6+1 | 37 | 0 | MC | UB | 0 | 1 | 5 | 4 | 0 | 2 | 0 | 0 | 12 | success |
| 52 | GSX | CS(2) | 5+1 | 33 | 0 | SG | UB | 0 | 4 | 0 | 5 | 0 | 0 | 0 | 0 | 9 | success |
| 53 | YEX | VD(2) | 5 | 40 | PS(2) | MC | RU | 0 | 1 | 0 | 1 | 5 | 2 | 3 | 0 | 12 | success |
| 54 | CEJ | VD(1) | 5+2 | 27 | PS(1)PM(1) | MC | UB | 0 | 1 | 0 | 4 | 5 | 2 | 0 | 0 | 12 | success |
| 55 | LGD | VD(2) | 6+4 | 29 | 0 | MC | UB | 0 | 1 | 5 | 4 | 0 | 2 | 0 | 0 | 12 | success |
| 56 | HYY | CS(2) | 6+5 | 32 | 0 | MC | RU | 0 | 4 | 5 | 5 | 0 | 2 | 3 | 0 | 19 | success |
| 57 | ZLY | 0 | 6 | 18 | 0 | SG | UB | 1 | 0 | 5 | 0 | 0 | 0 | 0 | 2 | 7 | success |
| 58 | ZHN | VD(1) | 6 | 27 | 0 | MC | UB | 1 | 1 | 5 | 4 | 0 | 2 | 0 | 2 | 14 | success |
| 59 | LHY | 0 | 5+4 | 21 | PS(1) | SG | RU | 1 | 0 | 0 | 3 | 5 | 0 | 3 | 2 | 13 | success |
| 60 | ZYC | 0 | 6+1 | 25 | 0 | MC | UB | 2 | 0 | 5 | 4 | 0 | 2 | 0 | 2 | 13 | success |
| 61 | LXY | 0 | 6+2 | 21 | 0 | SG | UB | 1 | 0 | 5 | 3 | 0 | 0 | 0 | 2 | 10 | success |
| 62 | LXT | 0 | 5 | 28 | 0 | SG | UB | 2 | 0 | 0 | 4 | 0 | 0 | 0 | 2 | 6 | success |
| 63 | HAY | VD(1) | 7 | 36 | PS(1) | MC | UB | 0 | 1 | 9 | 4 | 5 | 2 | 0 | 0 | 21 | success |
| 64 | ZHQ | 0 | 6+1 | 20 | 0 | SG | UB | 1 | 0 | 5 | 3 | 0 | 0 | 0 | 2 | 10 | success |
| 65 | HWY | VD(1) | 6+6 | 24 | PM(1) | MC | RU | 0 | 1 | 5 | 3 | -2 | 2 | 3 | 0 | 12 | success |
| 66 | XWJ | 0 | 5+3 | 29 | PS(1) | SG | UB | 1 | 0 | 0 | 4 | 5 | 0 | 0 | 2 | 11 | success |
| 67 | XXY | VD(1)CS(1) | 5 | 32 | PS(2) | MC | UB | 1 | 4 | 0 | 5 | 5 | 2 | 0 | 2 | 18 | success |
| 68 | ZPL | VD(1) | 6+3 | 28 | 0 | MC | UB | 0 | 1 | 5 | 4 | 0 | 2 | 0 | 0 | 12 | success |
| 69 | SUJ | 0 | 6+1 | 23 | 0 | SG | RU | 1 | 0 | 5 | 3 | 0 | 0 | 3 | 2 | 13 | success |
| 70 | CSS | VD(1) | 5+1 | 34 | PS(3) | MC | UB | 1 | 1 | 0 | 5 | 5 | 2 | 0 | 2 | 15 | success |
| 71 | YJY | 0 | 6+1 | 21 | 0 | SG | UB | 1 | 0 | 5 | 3 | 0 | 0 | 0 | 2 | 10 | success |
| 72 | KJJ | VD(1) | 5 | 34 | PM(2) | MC | RU | 1 | 1 | 0 | 5 | -2 | 2 | 3 | 2 | 11 | success |
| 73 | CCC | VD(1)CS(1) | 6 | 36 | PS(3)PM(1) | MC | UB | 1 | 4 | 5 | 4 | 5 | 2 | 0 | 2 | 22 | success |
| 74 | LMR | 0 | 5+1 | 19 | 0 | SG | RU | 3 | 0 | 0 | 0 | 0 | 0 | 3 | 2 | 5 | success |
| 75 | GJY | 0 | 5+ | 31 | 0 | MC | RU | 2 | 0 | 0 | 5 | 0 | 2 | 3 | 2 | 12 | success |
| 76 | CFL | 0 | 6+1 | 38 | 0 | SG | RU | 0 | 0 | 5 | 4 | 0 | 0 | 3 | 0 | 12 | success |
| 77 | WYQ | 0 | 6 | 26 | PS(1) | SG | UB | 1 | 0 | 5 | 4 | 5 | 0 | 0 | 2 | 16 | success |
| 78 | HQL | 0 | 6 | 22 | 0 | SG | RU | 1 | 0 | 5 | 3 | 0 | 0 | 3 | 2 | 13 | success |
| 79 | CMX | VD(2) | 5 | 36 | 0 | MC | UB | 1 | 1 | 0 | 4 | 0 | 2 | 0 | 2 | 9 | success |
| 80 | LKK | 0 | 6+3 | 23 | PS(1) | SG | UB | 1 | 0 | 5 | 3 | 5 | 0 | 0 | 2 | 15 | success |
| 81 | CYZ | 0 | 5+6 | 23 | 0 | SG | RU | 1 | 0 | 0 | 3 | 0 | 0 | 3 | 2 | 8 | success |
| 82 | LLZ | VD(1) | 7 | 19 | PM(1) | SG | RU | 0 | 1 | 9 | 0 | -2 | 0 | 3 | 0 | 11 | success |
| 83 | ZCY | 0 | 6+4 | 23 | 0 | SG | RU | 1 | 0 | 5 | 3 | 0 | 0 | 3 | 2 | 13 | success |
| 84 | LIM | 0 | 5+1 | 25 | 0 | SG | RU | 1 | 0 | 0 | 4 | 0 | 0 | 3 | 2 | 9 | success |
| 85 | ZJJ | 0 | 6+5 | 22 | 0 | SG | UB | 0 | 0 | 5 | 3 | 0 | 0 | 0 | 0 | 8 | success |
| 86 | ZHX | 0 | 6+2 | 20 | 0 | SG | RU | 1 | 0 | 5 | 3 | 0 | 0 | 3 | 2 | 13 | success |
| 87 | GQL | 0 | 6+3 | 26 | 0 | SG | RU | 0 | 0 | 5 | 4 | 0 | 0 | 3 | 0 | 12 | success |
| 88 | WZX | 0 | 6 | 26 | 0 | SG | UB | 1 | 0 | 5 | 4 | 0 | 0 | 0 | 2 | 11 | success |
| 89 | FZT | 0 | 5+4 | 23 | 0 | SG | UB | 0 | 0 | 0 | 3 | 0 | 0 | 0 | 0 | 3 | success |
| 90 | FXT | 0 | 6+3 | 22 | 0 | SG | UB | 0 | 0 | 5 | 3 | 0 | 0 | 0 | 0 | 8 | success |
| 91 | WAL | CS(1) | 6+3 | 38 | PM(2) | MC | UB | 0 | 4 | 5 | 4 | -2 | 2 | 0 | 0 | 13 | success |
| 92 | LIP | 0 | 5+5 | 32 | 0 | SG | UB | 1 | 0 | 0 | 5 | 0 | 0 | 0 | 2 | 7 | success |
| 93 | JYW | 0 | 6+2 | 25 | 0 | SG | UB | 1 | 0 | 5 | 4 | 0 | 0 | 0 | 2 | 11 | success |
| 94 | ZMT | 0 | 6+2 | 18 | 0 | SG | UB | 1 | 0 | 5 | 0 | 0 | 0 | 0 | 2 | 7 | success |
| 95 | QLF | CS(2) | 6+4 | 39 | PM(1) | MC | UB | 0 | 4 | 5 | 4 | -2 | 2 | 0 | 0 | 13 | success |
| 96 | ZHH | VD(1) | 5+6 | 33 | PS(3) | MC | UB | 0 | 1 | 0 | 5 | 5 | 2 | 0 | 0 | 13 | success |
| 97 | GUM | 0 | 6 | 25 | 0 | SG | UB | 1 | 0 | 5 | 4 | 0 | 0 | 0 | 2 | 11 | success |
| 98 | JXH | VD(2) | 5+6 | 31 | PS(3) | MC | UB | 2 | 1 | 0 | 5 | 5 | 2 | 0 | 2 | 15 | success |
| 99 | DYF | 0 | 5 | 28 | 0 | MC | UB | 1 | 0 | 0 | 4 | 0 | 2 | 0 | 2 | 8 | success |
| 100 | LXL | 0 | 6+1 | 20 | 0 | SG | UB | 0 | 0 | 5 | 3 | 0 | 0 | 0 | 0 | 8 | success |
| 101 | WQI | 0 | 6+4 | 30 | 0 | SG | UB | 0 | 0 | 5 | 5 | 0 | 0 | 0 | 0 | 10 | success |
| 102 | XFY | VD(2) | 6+4 | 36 | 0 | MC | UB | 1 | 1 | 5 | 4 | 0 | 2 | 0 | 2 | 14 | success |
| 103 | YHL | 0 | 7+3 | 24 | 0 | SG | UB | 1 | 0 | 9 | 3 | 0 | 0 | 0 | 2 | 14 | success |
| 104 | ZHY | 0 | 4+5 | 18 | 0 | SG | UB | 0 | 0 | 0 | 0 | 0 | 0 | 0 | 0 | 0 | success |
| 105 | TSS | 0 | 5+2 | 24 | 0 | SG | UB | 1 | 0 | 0 | 3 | 0 | 0 | 0 | 2 | 5 | success |
| 106 | LNS | VD(2) | 5+5 | 35 | 0 | MC | UB | 0 | 1 | 0 | 4 | 0 | 2 | 0 | 0 | 7 | success |
| 107 | ZSY | 0 | 5+3 | 29 | PM(1) | MC | UB | 0 | 0 | 0 | 4 | -2 | 2 | 0 | 0 | 4 | success |
| 108 | PYX | 0 | 5+4 | 24 | 0 | SG | UB | 0 | 0 | 0 | 3 | 0 | 0 | 0 | 0 | 3 | success |
| 109 | HXL | 0 | 5+6 | 16 | 0 | SG | UB | 1 | 0 | 0 | 0 | 0 | 0 | 0 | 2 | 2 | success |
| 110 | WAY | 0 | 5 | 25 | 0 | SG | UB | 2 | 0 | 0 | 4 | 0 | 0 | 0 | 2 | 6 | success |
| 111 | LHA | 0 | 5+6 | 34 | 0 | SG | RU | 1 | 0 | 0 | 5 | 0 | 0 | 3 | 2 | 10 | success |
| 112 | YXL | CS(1) | 6+5 | 37 | PM(1) | MC | RU | 0 | 4 | 5 | 4 | -2 | 2 | 3 | 0 | 16 | success |
| 113 | WAR | CS(1) | 6 | 33 | 0 | MC | RU | 0 | 4 | 5 | 5 | 0 | 2 | 3 | 0 | 19 | success |
| 114 | HLL | 0 | 6 | 24 | 0 | SG | UB | 1 | 0 | 5 | 3 | 0 | 0 | 0 | 2 | 10 | success |
| 115 | LWT | VD(2) | 6+1 | 26 | 0 | MC | UB | 0 | 1 | 5 | 4 | 0 | 2 | 0 | 0 | 12 | success |
| 116 | LXJ | 0 | 5+6 | 28 | 0 | SG | UB | 1 | 0 | 0 | 4 | 0 | 0 | 0 | 2 | 6 | success |
| 117 | JYH | 0 | 6 | 32 | PM(1) | SG | UB | 0 | 0 | 5 | 5 | -2 | 0 | 0 | 0 | 8 | success |
| 118 | WYY | 0 | 6+1 | 24 | 0 | SG | UB | 0 | 0 | 5 | 3 | 0 | 0 | 0 | 0 | 8 | success |
| 119 | YWJ | 0 | 6+1 | 20 | 0 | SG | RU | 0 | 0 | 5 | 3 | 0 | 0 | 3 | 0 | 11 | success |
| 120 | LXZ | 0 | 5+3 | 18 | PM(1) | SG | UB | 1 | 0 | 0 | 0 | -2 | 0 | 0 | 2 | 0 | success |
| 121 | ZMM | VD(2) | 5 | 35 | 0 | MC | UB | 0 | 1 | 0 | 4 | 0 | 2 | 0 | 0 | 7 | success |
| 122 | ZJL | CS(1) | 5+2 | 36 | PS(2) | MC | UB | 0 | 4 | 0 | 4 | 5 | 2 | 0 | 0 | 15 | success |
| 123 | LDY | VD(2) | 5+3 | 35 | 0 | MC | RU | 1 | 1 | 0 | 4 | 0 | 2 | 3 | 2 | 12 | success |
| 124 | CWJ | VD(1) | 5 | 27 | PS(3) | MC | UB | 2 | 1 | 0 | 4 | 5 | 2 | 0 | 2 | 14 | success |
| 125 | YYM | VD(2) | 5 | 37 | 0 | MC | UB | 3 | 1 | 0 | 4 | 0 | 2 | 0 | 2 | 9 | success |
| 126 | LIH | 0 | 5 | 34 | 0 | SG | UB | 1 | 0 | 0 | 5 | 0 | 0 | 0 | 2 | 7 | success |
| 127 | ZAX | CS(3) | 5 | 34 | PS(1) | MC | UB | 1 | 4 | 0 | 5 | 5 | 2 | 0 | 2 | 18 | success |
| 128 | LZF | VD(2) | 6+2 | 32 | PS(1) | MC | RU | 0 | 1 | 5 | 5 | 5 | 2 | 3 | 0 | 21 | success |
| 129 | XQH | 0 | 5 | 23 | 0 | SG | UB | 1 | 0 | 0 | 3 | 0 | 0 | 0 | 2 | 5 | success |
| 130 | YMX | 0 | 6 | 17 | 0 | SG | UB | 1 | 0 | 5 | 0 | 0 | 0 | 0 | 2 | 7 | success |
| 131 | CSQ | 0 | 5 | 28 | PM(1) | SG | UB | 2 | 0 | 0 | 4 | -2 | 0 | 0 | 2 | 4 | success |
| 132 | JJJ | 0 | 6+1 | 35 | 0 | SG | UB | 1 | 0 | 5 | 4 | 0 | 0 | 0 | 2 | 11 | success |
| 133 | ZXL | 0 | 6+1 | 31 | 0 | SG | UB | 2 | 0 | 5 | 5 | 0 | 0 | 0 | 2 | 12 | success |
| 134 | WLP | VD(1) | 6+3 | 35 | 0 | MC | UB | 0 | 1 | 5 | 4 | 0 | 2 | 0 | 0 | 12 | success |
| 135 | HKX | 0 | 6+2 | 19 | 0 | SG | RU | 0 | 0 | 5 | 0 | 0 | 0 | 3 | 0 | 8 | success |
| 136 | LNJ | 0 | 5+2 | 21 | 0 | SG | RU | 0 | 0 | 0 | 3 | 0 | 0 | 3 | 0 | 6 | success |
| 137 | WYT | VD(1) | 6+6 | 35 | 0 | MC | UB | 0 | 1 | 5 | 4 | 0 | 2 | 0 | 0 | 12 | success |
| 138 | WHM | 0 | 6+1 | 33 | 0 | MC | UB | 0 | 0 | 5 | 5 | 0 | 2 | 0 | 0 | 12 | success |
| 139 | WYP | VD(2) | 5+2 | 33 | PS(1) | SG | UB | 0 | 1 | 0 | 5 | 5 | 0 | 0 | 0 | 11 | success |
| 140 | LQY | VD(1) | 6 | 21 | 0 | SG | RU | 2 | 1 | 5 | 3 | 0 | 0 | 3 | 2 | 14 | success |
| 141 | CYX | 0 | 6+2 | 24 | PS(1) | SG | UB | 2 | 0 | 5 | 3 | 5 | 0 | 0 | 2 | 15 | success |
| 142 | WAJ | 0 | 6 | 26 | 0 | SG | RU | 2 | 0 | 5 | 4 | 0 | 0 | 3 | 2 | 14 | success |
| 143 | ZML | 0 | 6 | 25 | 0 | SG | RU | 0 | 0 | 5 | 4 | 0 | 0 | 3 | 0 | 12 | success |
| 144 | HYP | 0 | 6+3 | 25 | 0 | SG | RU | 1 | 0 | 5 | 4 | 0 | 0 | 3 | 2 | 14 | success |
| 145 | WAY | 0 | 5+5 | 23 | 0 | SG | UB | 1 | 0 | 0 | 3 | 0 | 0 | 0 | 2 | 5 | success |
| 146 | LQF | VD(2) | 6+4 | 40 | PS(1) | MC | UB | 1 | 1 | 5 | 1 | 5 | 2 | 0 | 2 | 16 | success |
| 147 | WLS | VD(1)CS(1) | 6 | 38 | PS(1) | MC | UB | 0 | 4 | 5 | 4 | 5 | 2 | 0 | 0 | 20 | success |
| 148 | LZR | VD(2) | 6+2 | 26 | 0 | MC | UB | 1 | 1 | 5 | 4 | 0 | 2 | 0 | 2 | 14 | success |
| 149 | ZWP | 0 | 5+2 | 21 | PM(1) | SG | RU | 3 | 0 | 0 | 3 | -2 | 0 | 3 | 2 | 6 | success |
| 150 | LHZ | CS(1) | 5 | 27 | 0 | MC | UB | 1 | 4 | 0 | 4 | 0 | 2 | 0 | 2 | 12 | success |
| 151 | ZXY | 0 | 5+1 | 21 | 0 | SG | RU | 1 | 0 | 0 | 3 | 0 | 0 | 3 | 2 | 8 | success |
| 152 | ZYA | 0 | 5 | 18 | 0 | SG | UB | 0 | 0 | 0 | 0 | 0 | 0 | 0 | 0 | 0 | success |
| 153 | CQR | VD(2) | 5+3 | 31 | PS(2) | MC | UB | 0 | 1 | 0 | 5 | 5 | 2 | 0 | 0 | 13 | success |
| 154 | LSQ | 0 | 5+6 | 27 | 0 | SG | RU | 0 | 0 | 0 | 4 | 0 | 0 | 3 | 0 | 7 | success |
| 155 | LWT | 0 | 5 | 20 | 0 | SG | UB | 1 | 0 | 0 | 3 | 0 | 0 | 0 | 2 | 5 | success |
| 156 | YAY | CS(1) | 6+4 | 28 | 0 | SG | UB | 0 | 4 | 5 | 4 | 0 | 0 | 0 | 0 | 13 | success |
| 157 | CYF | VD(1) | 6+4 | 34 | PM(1) | MC | UB | 0 | 1 | 5 | 5 | -2 | 2 | 0 | 0 | 11 | success |
| 158 | TUS | 0 | 5+2 | 24 | 0 | SG | UB | 1 | 0 | 0 | 3 | 0 | 0 | 0 | 2 | 5 | success |
| 159 | LIJ | 0 | 6+1 | 21 | PS(1) | SG | UB | 0 | 0 | 5 | 3 | 5 | 0 | 0 | 0 | 13 | success |
| 160 | LUM | VD(2) | 5 | 30 | PS(2) | SG | UB | 1 | 1 | 0 | 5 | 5 | 0 | 0 | 2 | 13 | success |
| 161 | HJQ | CS(1) | 5+5 | 28 | 0 | MC | RU | 0 | 4 | 0 | 4 | 0 | 2 | 3 | 0 | 13 | success |
| 162 | WAL | VD(1) | 6 | 37 | PS(1) | MC | UB | 1 | 1 | 5 | 4 | 5 | 2 | 0 | 2 | 19 | success |
| 163 | WAK | VD(2) | 5 | 30 | 0 | SG | UB | 2 | 1 | 0 | 5 | 0 | 0 | 0 | 2 | 8 | success |
| 164 | LXR | VD(2) | 5+5 | 39 | PS(1) | MC | UB | 0 | 1 | 0 | 4 | 5 | 2 | 0 | 0 | 12 | success |
| 165 | WAY | VD(2) | 6 | 36 | PM(1) | MC | RU | 0 | 1 | 0 | 4 | -2 | 2 | 3 | 0 | 8 | success |
| 166 | ZWP | VD(1) | 6+5 | 20 | 0 | SG | UB | 1 | 1 | 5 | 3 | 0 | 0 | 0 | 2 | 11 | success |
| 167 | YCQ | VD(2) | 5+6 | 37 | PM(1) | MC | UB | 1 | 1 | 0 | 4 | -2 | 2 | 0 | 2 | 7 | success |
| 168 | HYL | VD(1) | 5 | 27 | 0 | MC | UB | 3 | 1 | 0 | 4 | 0 | 2 | 0 | 2 | 9 | success |
| 169 | ZXY | 0 | 5 | 23 | 0 | SG | UB | 0 | 0 | 0 | 3 | 0 | 0 | 0 | 0 | 3 | success |
| 170 | XZY | 0 | 5+3 | 20 | 0 | SG | UB | 1 | 0 | 0 | 3 | 0 | 0 | 0 | 2 | 5 | success |
| 171 | ZDD | 0 | 6 | 25 | 0 | SG | UB | 1 | 0 | 5 | 4 | 0 | 0 | 0 | 2 | 11 | success |
| 172 | CSY | 0 | 5+1 | 22 | 0 | SG | RU | 1 | 0 | 0 | 3 | 0 | 0 | 3 | 2 | 8 | success |
| 173 | YYQ | CS(1) | 5+5 | 26 | 0 | SG | UB | 1 | 4 | 0 | 4 | 0 | 0 | 0 | 2 | 10 | success |
| 174 | HXL | 0 | 5+6 | 16 | 0 | SG | UB | 1 | 0 | 0 | 0 | 0 | 0 | 0 | 2 | 2 | success |
| 175 | HJR | 0 | 5 | 21 | 0 | SG | UB | 1 | 0 | 0 | 3 | 0 | 0 | 0 | 2 | 5 | success |
| 176 | HJN | 0 | 5 | 26 | 0 | SG | UB | 0 | 0 | 0 | 4 | 0 | 0 | 0 | 0 | 4 | success |
| 177 | HYQ | VD(1) | 6+5 | 25 | PM(2) | SG | RU | 0 | 1 | 5 | 4 | -2 | 0 | 3 | 0 | 11 | success |
| 178 | CHL | VD(1) | 6+2 | 42 | PM(3) | MC | UB | 0 | 1 | 5 | 1 | -2 | 2 | 0 | 0 | 7 | success |
| 179 | ZWJ | 0 | 6+3 | 29 | 0 | SG | UB | 0 | 0 | 5 | 4 | 0 | 0 | 0 | 0 | 9 | success |
| 180 | WAT | 0 | 5+1 | 18 | 0 | SG | RU | 1 | 0 | 0 | 0 | 0 | 0 | 3 | 2 | 5 | success |
| 181 | MST | 0 | 5+6 | 23 | 0 | SG | UB | 1 | 0 | 0 | 3 | 0 | 0 | 0 | 2 | 5 | success |
| 182 | SZY | 0 | 5+5 | 23 | 0 | SG | UB | 2 | 0 | 0 | 3 | 0 | 0 | 0 | 2 | 5 | success |
| 183 | HBB | 0 | 6+2 | 20 | 0 | SG | UB | 1 | 0 | 5 | 3 | 0 | 0 | 0 | 2 | 10 | success |
| 184 | LJX | 0 | 5 | 26 | PS(1) | SG | UB | 0 | 0 | 0 | 4 | 5 | 0 | 0 | 0 | 9 | success |
| 185 | WKY | 0 | 5+3 | 21 | PS(1) | SG | RU | 2 | 0 | 0 | 3 | 5 | 0 | 3 | 2 | 13 | success |
| 186 | SHY | 0 | 5+1 | 35 | 0 | SG | RU | 0 | 0 | 0 | 4 | 0 | 0 | 3 | 0 | 7 | success |
| 187 | XYF | 0 | 6 | 26 | 0 | SG | UB | 0 | 0 | 5 | 4 | 0 | 0 | 0 | 0 | 9 | success |
| 188 | FYY | 0 | 5+6 | 22 | PM(1) | SG | RU | 1 | 0 | 0 | 3 | -2 | 0 | 3 | 2 | 6 | success |
| 189 | YGJ | 0 | 5 | 26 | PS(1) | SG | UB | 2 | 0 | 0 | 4 | 5 | 0 | 0 | 2 | 11 | success |
| 190 | CSY | 0 | 5+1 | 23 | 0 | SG | UB | 1 | 0 | 0 | 3 | 0 | 0 | 0 | 2 | 5 | success |
| 191 | HAX | 0 | 6+1 | 31 | 0 | SG | UB | 1 | 0 | 5 | 5 | 0 | 0 | 0 | 2 | 12 | success |
| 192 | LYF | 0 | 5 | 21 | PM(1) | SG | RU | 2 | 0 | 0 | 3 | -2 | 0 | 3 | 2 | 6 | success |
| 193 | LJX | 0 | 5+3 | 20 | 0 | SG | UB | 2 | 0 | 0 | 3 | 0 | 0 | 0 | 2 | 5 | success |
| 194 | HXL | VD(1) | 5+4 | 25 | PM(3) | MC | UB | 1 | 1 | 0 | 4 | -2 | 2 | 0 | 2 | 7 | success |
| 195 | CLS | 0 | 6+5 | 26 | 0 | SG | UB | 4 | 0 | 5 | 4 | 0 | 0 | 0 | 2 | 11 | success |
| 196 | WQH | 0 | 5+2 | 21 | 0 | SG | UB | 1 | 0 | 0 | 3 | 0 | 0 | 0 | 2 | 5 | success |
| 197 | LYJ | 0 | 6 | 23 | 0 | SG | UB | 0 | 0 | 5 | 3 | 0 | 0 | 0 | 0 | 8 | success |
| 198 | WSL | VD(2) | 5+5 | 38 | 0 | MC | UB | 0 | 1 | 0 | 4 | 0 | 2 | 0 | 0 | 7 | success |
| 199 | LIY | 0 | 6+1 | 28 | PM(1) | SG | UB | 1 | 0 | 5 | 4 | -2 | 0 | 0 | 2 | 9 | success |
| 200 | LYL | VD(1) | 5 | 32 | 0 | MC | UB | 1 | 1 | 0 | 5 | 0 | 2 | 0 | 2 | 10 | success |
| 201 | RXC | 0 | 5+5 | 27 | PM(1) | SG | UB | 0 | 0 | 0 | 4 | -2 | 0 | 0 | 0 | 2 | success |
| 202 | WSQ | 0 | 6+3 | 20 | 0 | SG | UB | 0 | 0 | 5 | 3 | 0 | 0 | 0 | 0 | 8 | success |
| 203 | KAQ | VD(1) | 5+1 | 26 | 0 | MC | UB | 1 | 1 | 0 | 4 | 0 | 2 | 0 | 2 | 9 | success |
| 204 | LIM | VD(1) | 5+2 | 29 | 0 | MC | UB | 0 | 1 | 0 | 4 | 0 | 2 | 0 | 0 | 7 | success |
| 205 | ZYN | 0 | 6 | 23 | 0 | SG | UB | 1 | 0 | 5 | 3 | 0 | 0 | 0 | 2 | 10 | success |
| 206 | LYY | VD(1) | 6+5 | 33 | PS(2) | MC | UB | 0 | 1 | 5 | 5 | 5 | 2 | 0 | 0 | 18 | success |
| 207 | WDB | 0 | 5+3 | 22 | 0 | SG | UB | 1 | 0 | 0 | 3 | 0 | 0 | 0 | 2 | 5 | success |
| 208 | ZJH | 0 | 6+4 | 21 | 0 | SG | UB | 1 | 0 | 5 | 3 | 0 | 0 | 0 | 2 | 10 | success |
| 209 | YWY | 0 | 6+3 | 25 | 0 | SG | UB | 0 | 0 | 5 | 4 | 0 | 0 | 0 | 0 | 9 | success |
| 210 | LJM | 0 | 4 | 18 | 0 | SG | RU | 1 | 0 | 0 | 0 | 0 | 0 | 3 | 2 | 5 | success |
| 211 | WNQ | VD(2) | 6+3 | 33 | 0 | MC | UB | 0 | 1 | 5 | 5 | 0 | 2 | 0 | 0 | 13 | success |
| 212 | WRM | VD(2) | 5+6 | 38 | PM(2) | MC | UB | 0 | 1 | 0 | 4 | -2 | 2 | 0 | 0 | 5 | success |
| 213 | XSC | 0 | 6+5 | 24 | 0 | SG | UB | 0 | 0 | 5 | 3 | 0 | 0 | 0 | 0 | 8 | success |
| 214 | ZLY | 0 | 6+4 | 20 | 0 | SG | UB | 0 | 0 | 5 | 3 | 0 | 0 | 0 | 0 | 8 | success |
| 215 | WRM | VD(2) | 6+1 | 37 | PM(1) | MC | UB | 0 | 1 | 5 | 4 | -2 | 2 | 0 | 0 | 10 | success |
| 216 | LPM | 0 | 6+2 | 22 | 0 | SG | RU | 1 | 0 | 5 | 3 | 0 | 0 | 3 | 2 | 13 | success |
| 217 | LHY | 0 | 6 | 21 | 0 | SG | UB | 1 | 0 | 5 | 3 | 0 | 0 | 0 | 2 | 10 | success |
| 218 | HYL | VD(1) | 6+1 | 35 | 0 | MC | UB | 0 | 1 | 5 | 4 | 0 | 2 | 0 | 0 | 12 | success |
| 219 | MJY | VD(1) | 6 | 23 | 0 | MC | RU | 3 | 1 | 5 | 3 | 0 | 2 | 3 | 2 | 16 | success |
| 220 | SMM | 0 | 5 | 28 | PS(1) | SG | UB | 2 | 0 | 0 | 4 | 5 | 0 | 0 | 2 | 11 | success |
| 221 | DST | 0 | 5 | 26 | 0 | SG | UB | 1 | 0 | 0 | 4 | 0 | 0 | 0 | 2 | 6 | success |
| 222 | HYL | 0 | 6 | 21 | 0 | SG | UB | 0 | 0 | 5 | 3 | 0 | 0 | 0 | 0 | 8 | success |
| 223 | JYY | 0 | 6+1 | 20 | 0 | SG | RU | 0 | 0 | 5 | 3 | 0 | 0 | 3 | 0 | 11 | success |
| 224 | WMR | 0 | 5+2 | 19 | PS(1)PM(1) | SG | RU | 0 | 0 | 0 | 0 | 5 | 0 | 3 | 0 | 8 | success |
| 225 | CBL | VD(2) | 5+3 | 34 | 0 | MC | UB | 2 | 1 | 0 | 5 | 0 | 2 | 0 | 2 | 10 | success |
| 226 | XJM | 0 | 6+2 | 19 | 0 | SG | UB | 1 | 0 | 5 | 0 | 0 | 0 | 0 | 2 | 7 | success |
| 227 | ZCB | 0 | 6+1 | 29 | 0 | SG | RU | 1 | 0 | 5 | 4 | 0 | 0 | 3 | 2 | 14 | success |
| 228 | LML | 0 | 6 | 28 | PS(1) | SG | RU | 0 | 0 | 5 | 4 | 5 | 0 | 3 | 0 | 17 | success |
| 229 | ZMJ | 0 | 6 | 26 | PM(2) | SG | UB | 2 | 0 | 5 | 4 | -2 | 0 | 0 | 2 | 9 | success |
| 230 | ZHY | 0 | 5 | 23 | 0 | SG | UB | 0 | 0 | 0 | 3 | 0 | 0 | 0 | 0 | 3 | success |
| 231 | YYR | 0 | 6+2 | 25 | 0 | SG | UB | 1 | 0 | 5 | 4 | 0 | 0 | 0 | 2 | 11 | success |
| 232 | XMX | 0 | 5+4 | 27 | 0 | SG | RU | 1 | 0 | 0 | 4 | 0 | 0 | 3 | 2 | 9 | success |
| 233 | GXZ | 0 | 5+4 | 18 | 0 | SG | UB | 0 | 0 | 0 | 0 | 0 | 0 | 0 | 0 | 0 | success |
| 234 | CHX | 0 | 6+1 | 26 | PS(1) | SG | UB | 2 | 0 | 5 | 4 | 5 | 0 | 0 | 2 | 16 | success |
| 235 | YYJ | 0 | 5+6 | 21 | PM(1) | SG | RU | 1 | 0 | 0 | 3 | -2 | 0 | 3 | 2 | 6 | success |
| 236 | NMK | 0 | 5 | 23 | 0 | SG | RU | 2 | 0 | 0 | 3 | 0 | 0 | 3 | 2 | 8 | success |
| 237 | LJL | 0 | 5+6 | 21 | PS(1) | SG | UB | 3 | 0 | 0 | 3 | 5 | 0 | 0 | 2 | 10 | success |
| 238 | ZXH | 0 | 6+3 | 27 | 0 | SG | UB | 2 | 0 | 5 | 4 | 0 | 0 | 0 | 2 | 11 | success |
| 239 | ZXY | 0 | 6+1 | 27 | 0 | SG | UB | 0 | 0 | 5 | 4 | 0 | 0 | 0 | 0 | 9 | success |
| 240 | CXY | 0 | 5+3 | 19 | PS(1) | SG | RU | 1 | 0 | 0 | 0 | 5 | 0 | 3 | 2 | 10 | success |
| 241 | SHX | 0 | 5 | 27 | 0 | SG | UB | 1 | 0 | 0 | 4 | 0 | 0 | 0 | 2 | 6 | success |
| 242 | BXF | 0 | 6+5 | 26 | 0 | SG | UB | 1 | 0 | 5 | 4 | 0 | 0 | 0 | 2 | 11 | success |
| 243 | CXX | 0 | 5+6 | 25 | 0 | SG | UB | 1 | 0 | 0 | 4 | 0 | 0 | 0 | 2 | 6 | success |
| 244 | LIY | 0 | 5+2 | 27 | 0 | SG | UB | 2 | 0 | 0 | 4 | 0 | 0 | 0 | 2 | 6 | success |
| 245 | HSF | VD(1) | 5+3 | 35 | 0 | MC | UB | 0 | 1 | 0 | 4 | 0 | 2 | 0 | 0 | 7 | success |
| 246 | ZCB | 0 | 6 | 29 | 0 | SG | RU | 1 | 0 | 5 | 4 | 0 | 0 | 3 | 2 | 14 | success |
| 247 | LJY | 0 | 6 | 29 | PM(1) | MC | UB | 0 | 0 | 0 | 4 | -2 | 2 | 0 | 0 | 4 | success |
| 248 | YHY | VD(1) | 5+1 | 24 | 0 | SG | UB | 3 | 1 | 0 | 3 | 0 | 0 | 0 | 2 | 6 | success |
| 249 | GXR | 0 | 5+2 | 21 | 0 | SG | UB | 1 | 0 | 0 | 3 | 0 | 0 | 0 | 2 | 5 | success |
| 250 | YHL | 0 | 7+3 | 24 | 0 | SG | UB | 1 | 0 | 9 | 3 | 0 | 0 | 0 | 2 | 14 | success |
| 251 | ZYN | 0 | 6 | 23 | 0 | SG | UB | 1 | 0 | 5 | 3 | 0 | 0 | 0 | 2 | 10 | success |
| 252 | GWJ | 0 | 6 | 25 | 0 | SG | RU | 2 | 0 | 5 | 4 | 0 | 0 | 3 | 2 | 14 | success |
| 253 | WZX | 0 | 6 | 26 | 0 | SG | UB | 0 | 0 | 5 | 4 | 0 | 0 | 0 | 0 | 9 | success |
| 254 | LXT | 0 | 5 | 28 | 0 | SG | UB | 2 | 0 | 0 | 4 | 0 | 0 | 0 | 2 | 6 | success |
| 255 | HXY | VD(2) | 6+3 | 36 | PS(1) | MC | UB | 0 | 1 | 5 | 4 | 5 | 2 | 0 | 0 | 17 | success |
| 256 | ZHY | VD(2) | 6+2 | 35 | 0 | SG | UB | 0 | 1 | 5 | 4 | 0 | 0 | 0 | 0 | 10 | success |
| 257 | LMY | VD(2) | 5 | 39 | PS(3) | MC | UB | 3 | 1 | 0 | 4 | 5 | 2 | 0 | 2 | 14 | success |
| 258 | WBB | CS(2) | 5 | 33 | 0 | MC | UB | 0 | 4 | 0 | 5 | 0 | 2 | 0 | 0 | 11 | success |
| 259 | YLL | CS(2) | 7+1 | 38 | PS(1)PM(1) | MC | UB | 1 | 4 | 9 | 4 | 5 | 2 | 0 | 2 | 26 | success |
| 260 | ZXL | VD(3) | 5 | 35 | PS(1) | MC | UB | 1 | 1 | 0 | 4 | 5 | 2 | 0 | 2 | 14 | success |
| 261 | DHL | 0 | 6 | 21 | 0 | SG | RU | 2 | 0 | 5 | 3 | 0 | 0 | 3 | 2 | 13 | success |
| 262 | HJR | 0 | 5 | 21 | 0 | SG | UB | 1 | 0 | 0 | 3 | 0 | 0 | 0 | 2 | 5 | success |
| 263 | WFN | 0 | 5 | 21 | PS(1) | SG | RU | 1 | 0 | 0 | 3 | 5 | 0 | 3 | 2 | 13 | success |
| 264 | YMZ | 0 | 8 | 18 | 0 | SG | RU | 1 | 0 | 12 | 0 | 0 | 0 | 3 | 2 | 17 | success |
| 265 | YAB | VD(1) | 5 | 32 | PS(2) | MC | RU | 1 | 1 | 0 | 5 | 5 | 2 | 3 | 2 | 18 | success |
| 266 | WXT | 0 | 6+5 | 20 | 0 | SG | RU | 0 | 0 | 5 | 3 | 0 | 0 | 3 | 0 | 11 | success |
| 267 | CTT | VD(1) | 7 | 23 | 0 | SG | RU | 2 | 1 | 9 | 3 | 0 | 0 | 3 | 2 | 18 | success |
| 268 | HLB | VD(2) | 6 | 38 | 0 | MC | RU | 3 | 1 | 5 | 4 | 0 | 2 | 3 | 2 | 17 | success |
| 269 | WTG | 0 | 5 | 18 | 0 | SG | RU | 1 | 0 | 0 | 0 | 0 | 0 | 3 | 2 | 5 | success |
| 270 | CYY | VD(1) | 5 | 27 | 0 | MC | UB | 1 | 1 | 0 | 4 | 0 | 2 | 0 | 2 | 9 | success |
| 271 | CYF | 0 | 7+4 | 18 | 0 | SG | RU | 0 | 0 | 9 | 0 | 0 | 0 | 3 | 0 | 12 | success |
| 272 | CYZ | 0 | 5+2 | 23 | 0 | SG | RU | 1 | 0 | 0 | 3 | 0 | 0 | 3 | 2 | 8 | success |
| 273 | CQY | CS(2) | 6+6 | 33 | PM(1) | MC | UB | 1 | 4 | 5 | 5 | -2 | 2 | 0 | 2 | 16 | success |
| 274 | GBB | VD(1)CS(1) | 5+4 | 35 | PS(2) | MC | UB | 0 | 4 | 0 | 4 | 5 | 2 | 0 | 0 | 15 | success |
| 275 | JGC | VD(1) | 6 | 32 | 0 | MC | UB | 6 | 1 | 5 | 5 | 0 | 2 | 0 | 2 | 15 | success |
| 276 | LCL | VD(1) | 7+1 | 23 | PM(1) | MC | UB | 0 | 1 | 9 | 3 | -2 | 2 | 0 | 0 | 13 | success |
| 277 | LDT | 0 | 6+2 | 22 | PM(1) | SG | UB | 1 | 0 | 5 | 3 | -2 | 0 | 0 | 2 | 8 | success |
| 278 | LXN | VD(1) | 5 | 29 | 0 | SG | UB | 2 | 1 | 0 | 4 | 0 | 0 | 0 | 2 | 7 | success |
| 279 | LWL | 0 | 6+3 | 24 | PS(1) | SG | RU | 0 | 0 | 5 | 3 | 5 | 0 | 3 | 0 | 16 | success |
| 280 | LQQ | 0 | 5+3 | 25 | 0 | SG | UB | 0 | 0 | 0 | 4 | 0 | 0 | 0 | 0 | 4 | success |
| 281 | LQY | 0 | 6+1 | 33 | 0 | SG | UB | 0 | 0 | 5 | 5 | 0 | 0 | 0 | 0 | 10 | success |
| 282 | LUZ | VD(1) | 10+4 | 39 | PS(3) | MC | UB | 0 | 1 | 19 | 4 | 5 | 2 | 0 | 0 | 31 | success |
| 283 | LLJ | CS(1) | 5 | 28 | PS(1) | SG | RU | 1 | 4 | 0 | 4 | 5 | 0 | 3 | 2 | 18 | success |
| 284 | SIJ | VD(2) | 6+4 | 34 | PM(2) | MC | UB | 0 | 1 | 5 | 5 | -2 | 2 | 0 | 0 | 11 | success |
| 285 | TQW | VD(2) | 6 | 38 | PM(2) | MC | UB | 0 | 1 | 5 | 4 | -2 | 2 | 0 | 0 | 10 | success |
| 286 | LUJ | CS(1) | 5 | 28 | PM(1) | SG | RU | 1 | 4 | 0 | 4 | -2 | 0 | 3 | 2 | 11 | success |
| 287 | TAB | CS(1) | 6+6 | 26 | 0 | MC | RU | 1 | 4 | 5 | 4 | 0 | 2 | 3 | 2 | 20 | success |
| 288 | WFN | 0 | 5 | 21 | PS(1) | SG | RU | 1 | 0 | 0 | 3 | 5 | 0 | 3 | 2 | 13 | success |
| 289 | XZJ | VD(1) | 5 | 29 | PS(1) | MC | UB | 1 | 1 | 0 | 4 | 5 | 2 | 0 | 2 | 14 | success |
| 290 | XWJ | 0 | 5+3 | 29 | PM(1) | SG | UB | 1 | 0 | 0 | 4 | -2 | 0 | 0 | 2 | 4 | success |
| 291 | ZQY | CS(1) | 9 | 34 | PS(1) | MC | UB | 0 | 4 | 19 | 5 | 5 | 2 | 0 | 0 | 35 | success |
| 292 | ZJG | CS(2) | 6+1 | 39 | PS(2) | MC | UB | 0 | 4 | 5 | 4 | 5 | 2 | 0 | 0 | 20 | success |
| 293 | ZYS | 0 | 6+1 | 25 | PS(1) | SG | RU | 0 | 0 | 5 | 4 | 5 | 0 | 3 | 0 | 17 | success |
| 294 | ZMS | 0 | 6+3 | 21 | 0 | SG | RU | 1 | 0 | 5 | 3 | 0 | 0 | 3 | 2 | 13 | success |
| 295 | CSS | 0 | 6+2 | 25 | 0 | SG | RU | 0 | 0 | 5 | 4 | 0 | 0 | 3 | 0 | 12 | success |
| 296 | CYL | 0 | 6+5 | 24 | 0 | SG | UB | 0 | 0 | 5 | 3 | 0 | 0 | 0 | 0 | 8 | success |
| 297 | GLX | CS(2) | 5+4 | 34 | 0 | MC | UB | 0 | 4 | 0 | 5 | 0 | 2 | 0 | 0 | 11 | success |
| 298 | HMX | 0 | 6 | 25 | PS(1) | SG | UB | 1 | 0 | 5 | 4 | 5 | 0 | 0 | 2 | 16 | success |
| 299 | HPX | 0 | 5+2 | 22 | 0 | SG | UB | 2 | 0 | 0 | 3 | 0 | 0 | 0 | 2 | 5 | success |
| 300 | KXX | CS(1) | 5+1 | 38 | PM(1) | MC | UB | 0 | 4 | 0 | 4 | -2 | 2 | 0 | 0 | 8 | success |
| 301 | LXY | 0 | 6+1 | 30 | 0 | SG | UB | 0 | 0 | 5 | 5 | 0 | 0 | 0 | 0 | 10 | success |
| 302 | LXH | 0 | 5+1 | 35 | PS(2) | SG | UB | 1 | 0 | 0 | 4 | 5 | 0 | 0 | 2 | 11 | success |
| 303 | LDM | 0 | 5+3 | 28 | PS(1) | SG | RU | 2 | 0 | 0 | 4 | 5 | 0 | 3 | 2 | 14 | success |
| 304 | WYU | 0 | 6 | 31 | 0 | SG | UB | 0 | 0 | 5 | 5 | 0 | 0 | 0 | 0 | 10 | success |
| 305 | WLI | 0 | 6+3 | 26 | 0 | MC | UB | 0 | 0 | 5 | 4 | 0 | 2 | 0 | 0 | 11 | success |
| 306 | WXY | VD(2) | 6+2 | 39 | PM(3) | MC | UB | 0 | 1 | 5 | 4 | -2 | 2 | 0 | 0 | 10 | success |
| 307 | WMH | VD(1) | 6+2 | 29 | 0 | MC | UB | 0 | 1 | 5 | 4 | 0 | 2 | 0 | 0 | 12 | success |
| 308 | XMN | VD(1) | 6+3 | 31 | 0 | MC | UB | 0 | 1 | 5 | 5 | 0 | 2 | 0 | 0 | 13 | success |
| 309 | YXH | 0 | 9 | 20 | 0 | SG | RU | 2 | 0 | 19 | 3 | 0 | 0 | 3 | 2 | 27 | success |
| 310 | ZZH | 0 | 6 | 27 | 0 | SG | UB | 2 | 0 | 5 | 4 | 0 | 0 | 0 | 2 | 11 | success |
| 311 | ZYL | CS(2) | 6+4 | 32 | PS(2) | MC | UB | 0 | 4 | 5 | 5 | 5 | 2 | 0 | 0 | 21 | success |
| 312 | CAM | 0 | 5+1 | 24 | PS(1) | MC | RU | 2 | 0 | 0 | 3 | 5 | 2 | 3 | 2 | 15 | success |
| 313 | CMZ | 0 | 6+5 | 23 | 0 | SG | UB | 3 | 0 | 5 | 3 | 0 | 0 | 0 | 2 | 10 | success |
| 314 | CSJ | 0 | 6+1 | 30 | 0 | SG | RU | 1 | 0 | 5 | 5 | 0 | 0 | 3 | 2 | 15 | success |
| 315 | CSM | 0 | 6 | 20 | 0 | SG | RU | 1 | 0 | 5 | 3 | 0 | 0 | 3 | 2 | 13 | success |
| 316 | CYJ | VD(2) | 5+2 | 36 | PS(2) | MC | UB | 0 | 1 | 0 | 4 | 5 | 2 | 0 | 0 | 12 | success |
| 317 | CWT | CS(1) | 6 | 24 | 0 | SG | RU | 0 | 4 | 5 | 3 | 0 | 0 | 3 | 0 | 15 | success |
| 318 | FXQ | 0 | 6+3 | 34 | PS(1) | MC | RU | 0 | 0 | 5 | 5 | 5 | 2 | 3 | 0 | 20 | success |
| 319 | GYL | VD(2) | 6 | 29 | 0 | MC | UB | 1 | 1 | 5 | 4 | 0 | 2 | 0 | 2 | 14 | success |
| 320 | GLH | CS(1) | 5+6 | 36 | PS(2) | MC | UB | 1 | 4 | 0 | 4 | 5 | 2 | 0 | 2 | 17 | success |
| 321 | GFF | 0 | 5+5 | 28 | PS(1) | SG | RU | 0 | 0 | 0 | 4 | 5 | 0 | 3 | 0 | 12 | success |
| 322 | HSS | 0 | 6+5 | 24 | 0 | SG | RU | 0 | 0 | 5 | 3 | 0 | 0 | 3 | 0 | 11 | success |
| 323 | HXB | VD(2) | 5+2 | 27 | 0 | MC | UB | 1 | 1 | 0 | 4 | 0 | 2 | 0 | 2 | 9 | success |
| 324 | JJJ | 0 | 6+2 | 24 | 0 | SG | UB | 0 | 0 | 5 | 3 | 0 | 0 | 0 | 0 | 8 | success |
| 325 | LDT | 0 | 6+2 | 22 | PS(1) | SG | UB | 1 | 0 | 5 | 3 | 5 | 0 | 0 | 2 | 15 | success |
| 326 | LDL | CS(2) | 6 | 43 | PS(1)PM(1) | MC | UB | 1 | 4 | 5 | 1 | 5 | 2 | 0 | 2 | 19 | success |
| 327 | LLF | VD(2) | 6+5 | 31 | 0 | MC | UB | 1 | 1 | 5 | 5 | 0 | 2 | 0 | 2 | 15 | success |
| 328 | LMQ | CS(1) | 6 | 33 | 0 | MC | UB | 0 | 4 | 5 | 5 | 0 | 2 | 0 | 0 | 16 | success |
| 329 | LDA | 0 | 5 | 34 | 0 | SG | UB | 0 | 0 | 0 | 5 | 0 | 0 | 0 | 0 | 5 | success |
| 330 | LQY | 0 | 6+1 | 33 | 0 | SG | UB | 0 | 0 | 5 | 5 | 0 | 0 | 0 | 0 | 10 | success |
| 331 | LRJ | 0 | 6+2 | 19 | 0 | SG | RU | 0 | 0 | 5 | 0 | 0 | 0 | 3 | 0 | 8 | success |
| 332 | LXQ | VD(1) | 5 | 31 | 0 | MC | UB | 1 | 1 | 0 | 5 | 0 | 2 | 0 | 2 | 10 | success |
| 333 | LXX | CS(1) | 6+1 | 31 | PS(1) | SG | RU | 4 | 4 | 5 | 5 | 5 | 0 | 3 | 2 | 24 | success |
| 334 | LYX | 0 | 5+1 | 27 | 0 | MC | UB | 1 | 0 | 0 | 4 | 0 | 2 | 0 | 2 | 8 | success |
| 335 | SXR | VD(2) | 6 | 39 | PS(1)PM(1) | MC | UB | 0 | 1 | 5 | 4 | 5 | 2 | 0 | 0 | 17 | success |
| 336 | TYC | VD(1) | 5+3 | 26 | 0 | SG | RU | 0 | 1 | 0 | 4 | 0 | 0 | 3 | 0 | 8 | success |
| 337 | WFN | 0 | 5+1 | 21 | PS(1) | SG | RU | 1 | 0 | 0 | 3 | 5 | 0 | 3 | 2 | 13 | success |
| 338 | WJH | 0 | 5+5 | 24 | PM(1) | SG | UB | 2 | 0 | 0 | 3 | -2 | 0 | 0 | 2 | 3 | success |
| 339 | WMY | CS(2) | 5+3 | 32 | 0 | MC | UB | 3 | 4 | 0 | 5 | 0 | 2 | 0 | 2 | 13 | success |
| 340 | WJH | CS(1) | 5+1 | 30 | 0 | MC | UB | 2 | 4 | 0 | 5 | 0 | 2 | 0 | 2 | 13 | success |
| 341 | WYS | 0 | 5+4 | 25 | 0 | SG | RU | 0 | 0 | 0 | 4 | 0 | 0 | 3 | 0 | 7 | success |
| 342 | XYS | 0 | 6 | 25 | 0 | SG | UB | 0 | 0 | 5 | 4 | 0 | 0 | 0 | 0 | 9 | success |
| 343 | XLU | 0 | 6+2 | 26 | 0 | SG | UB | 0 | 0 | 5 | 4 | 0 | 0 | 0 | 0 | 9 | success |
| 344 | XYA | CS(2) | 6+1 | 38 | PS(1) | MC | UB | 0 | 4 | 5 | 4 | 5 | 2 | 0 | 0 | 20 | success |
| 345 | YYM | CS(1) | 6 | 29 | 0 | MC | UB | 1 | 4 | 5 | 4 | 0 | 2 | 0 | 2 | 17 | success |
| 346 | ZLM | VD(2) | 6 | 24 | PS(1) | MC | RU | 1 | 1 | 5 | 3 | 5 | 2 | 3 | 2 | 21 | success |
| 347 | WYZ | 0 | 6 | 23 | 0 | SG | UB | 1 | 0 | 5 | 3 | 0 | 0 | 0 | 2 | 10 | success |
| 348 | ZZF | VD(2) | 5+1 | 37 | 0 | MC | UB | 0 | 1 | 0 | 4 | 0 | 2 | 0 | 0 | 7 | success |
| 349 | ZCX | VD(1) | 5 | 33 | 0 | MC | UB | 2 | 1 | 0 | 5 | 0 | 2 | 0 | 2 | 10 | success |
| 350 | ZYT | CS(2)VD(1) | 5 | 31 | PM(1) | MC | UB | 3 | 4 | 0 | 5 | -2 | 2 | 0 | 2 | 11 | success |
| 351 | ZSS | 0 | 6+1 | 28 | 0 | SG | RU | 0 | 0 | 5 | 4 | 0 | 0 | 3 | 0 | 12 | success |
| 352 | BLM | CS(1) | 7+1 | 34 | PS(1)PM(1) | SG | UB | 0 | 4 | 9 | 5 | 5 | 0 | 0 | 0 | 23 | success |
| 353 | ZJY | VD(1) | 6 | 34 | PS(4) | MC | UB | 1 | 1 | 5 | 5 | 5 | 2 | 0 | 2 | 20 | success |
| 354 | ZSY | CS(1) | 6+1 | 25 | 0 | SG | RU | 2 | 4 | 5 | 4 | 0 | 0 | 3 | 2 | 18 | success |
| 355 | CFF | 0 | 5 | 30 | PS(1) | SG | RU | 1 | 0 | 0 | 5 | 5 | 0 | 3 | 2 | 15 | success |
| 356 | CJH | 0 | 6+3 | 23 | 0 | SG | UB | 0 | 0 | 5 | 3 | 0 | 0 | 0 | 0 | 8 | success |
| 357 | CYH | VD(2) | 5 | 41 | PS(2) | MC | UB | 1 | 1 | 0 | 1 | 5 | 2 | 0 | 2 | 11 | success |
| 358 | CYQ | 0 | 6 | 24 | 0 | SG | RU | 0 | 0 | 5 | 3 | 0 | 0 | 3 | 0 | 11 | success |
| 359 | CJL | 0 | 6+1 | 21 | 0 | SG | UB | 1 | 0 | 5 | 3 | 0 | 0 | 0 | 2 | 10 | success |
| 360 | FCL | 0 | 4+1 | 28 | 0 | SG | UB | 3 | 0 | 0 | 4 | 0 | 0 | 0 | 2 | 6 | success |
| 361 | GYD | 0 | 5+5 | 21 | PS(1)PM(1) | SG | UB | 5 | 0 | 0 | 3 | 5 | 0 | 0 | 2 | 10 | success |
| 362 | HYH | VD(2) | 5+1 | 33 | 0 | MC | RU | 1 | 1 | 0 | 5 | 0 | 2 | 3 | 2 | 13 | success |
| 363 | HZP | 0 | 6 | 27 | 0 | SG | UB | 0 | 0 | 5 | 4 | 0 | 0 | 0 | 0 | 9 | success |
| 364 | HLY | CS(1) | 6+1 | 31 | 0 | MC | UB | 4 | 4 | 5 | 5 | 0 | 2 | 0 | 2 | 18 | success |
| 365 | JYW | CS(1) | 5 | 24 | PS(1) | MC | UB | 2 | 4 | 0 | 3 | 5 | 2 | 0 | 2 | 16 | success |
| 366 | JJH | CS(2) | 6+4 | 34 | PM(1) | MC | RU | 0 | 4 | 5 | 5 | -2 | 2 | 3 | 0 | 17 | success |
| 367 | JJX | 0 | 5+6 | 23 | 0 | SG | RU | 1 | 0 | 0 | 3 | 0 | 0 | 3 | 2 | 8 | success |
| 368 | LXP | 0 | 6+1 | 22 | 0 | SG | RU | 0 | 0 | 5 | 3 | 0 | 0 | 3 | 0 | 11 | success |
| 369 | LYD | CS(1) | 6+1 | 23 | 0 | MC | RU | 2 | 4 | 5 | 3 | 0 | 2 | 3 | 2 | 19 | success |
| 370 | LMQ | 0 | 5 | 25 | 0 | SG | UB | 0 | 0 | 0 | 4 | 0 | 0 | 0 | 0 | 4 | success |
| 371 | LYX | 0 | 6+3 | 33 | PS(1) | SG | UB | 1 | 0 | 5 | 5 | 5 | 0 | 0 | 2 | 17 | success |
| 372 | LYM | VD(2) | 6+3 | 38 | PS(1)PM(3) | MC | RU | 0 | 1 | 5 | 4 | 5 | 2 | 3 | 0 | 20 | success |
| 373 | LZR | 0 | 6+3 | 22 | 0 | SG | RU | 0 | 0 | 5 | 3 | 0 | 0 | 3 | 0 | 11 | success |
| 374 | LCM | CS(2) | 5+1 | 30 | PS(2) | MC | UB | 3 | 4 | 0 | 5 | 5 | 2 | 0 | 2 | 18 | success |
| 375 | LLH | VD(1) | 5+5 | 32 | PS(1) | MC | UB | 0 | 1 | 0 | 5 | 5 | 2 | 0 | 0 | 13 | success |
| 376 | MMM | 0 | 6+4 | 23 | 0 | SG | RU | 0 | 0 | 5 | 3 | 0 | 0 | 3 | 0 | 11 | success |
| 377 | PHL | 0 | 5+1 | 27 | PS(1) | SG | UB | 2 | 0 | 0 | 4 | 5 | 0 | 0 | 2 | 11 | success |
| 378 | PWL | 0 | 6+2 | 20 | 0 | SG | RU | 1 | 0 | 5 | 3 | 0 | 0 | 3 | 2 | 13 | success |
| 379 | SHM | 0 | 5+2 | 20 | 0 | SG | UB | 2 | 0 | 0 | 3 | 0 | 0 | 0 | 2 | 5 | success |
| 380 | SQF | 0 | 6+4 | 26 | PS(1) | MC | RU | 0 | 0 | 5 | 4 | 5 | 2 | 3 | 0 | 19 | success |
| 381 | SXD | 0 | 5+2 | 25 | 0 | SG | UB | 1 | 0 | 0 | 4 | 0 | 0 | 0 | 2 | 6 | success |
| 382 | WLY | CS(1) | 5+4 | 23 | 0 | MC | UB | 1 | 4 | 0 | 3 | 0 | 2 | 0 | 2 | 11 | success |
| 383 | WQH | VD(2) | 5+1 | 37 | PS(2) | MC | UB | 0 | 1 | 0 | 4 | 5 | 2 | 0 | 0 | 12 | success |
| 384 | WLS | 0 | 5+2 | 27 | PS(1) | SG | UB | 1 | 0 | 0 | 4 | 5 | 0 | 0 | 2 | 11 | success |
| 385 | WSB | VD(2) | 6 | 27 | PM(1) | SG | RU | 1 | 1 | 5 | 4 | -2 | 0 | 3 | 2 | 13 | success |
| 386 | WYQ | CS(1) | 5+3 | 32 | 0 | MC | UB | 0 | 4 | 0 | 5 | 0 | 2 | 0 | 0 | 11 | success |
| 387 | XHL | 0 | 7+2 | 21 | 0 | SG | UB | 0 | 0 | 9 | 3 | 0 | 0 | 0 | 0 | 12 | success |
| 388 | XYP | 0 | 6+4 | 23 | 0 | SG | UB | 0 | 0 | 5 | 3 | 0 | 0 | 0 | 0 | 8 | success |
| 389 | XQH | 0 | 5 | 25 | 0 | SG | UB | 1 | 0 | 0 | 4 | 0 | 0 | 0 | 2 | 6 | success |
| 390 | XJR | VD(2) | 5+2 | 21 | 0 | MC | UB | 1 | 1 | 0 | 3 | 0 | 2 | 0 | 2 | 8 | success |
| 391 | XXQ | 0 | 5+2 | 27 | 0 | SG | RU | 1 | 0 | 0 | 4 | 0 | 0 | 3 | 2 | 9 | success |
| 392 | XYC | VD(2) | 6 | 37 | PM(1) | MC | UB | 3 | 1 | 5 | 4 | -2 | 2 | 0 | 2 | 12 | success |
| 393 | YHC | 0 | 5+6 | 22 | 0 | SG | RU | 1 | 0 | 0 | 3 | 0 | 0 | 3 | 2 | 8 | success |
| 394 | YFH | VD(1) | 6 | 35 | PS(2) | MC | UB | 0 | 1 | 5 | 4 | 5 | 2 | 0 | 0 | 17 | success |
| 395 | YJY | 0 | 6+6 | 22 | 0 | SG | RU | 1 | 0 | 5 | 3 | 0 | 0 | 3 | 2 | 13 | success |
| 396 | YLM | 0 | 5+3 | 22 | 0 | SG | RU | 1 | 0 | 0 | 3 | 0 | 0 | 3 | 2 | 8 | success |
| 397 | YSW | 0 | 6+2 | 20 | 0 | SG | UB | 1 | 0 | 5 | 3 | 0 | 0 | 0 | 2 | 10 | success |
| 398 | YLZ | VD(1) | 5+3 | 35 | PS(1) | MC | UB | 1 | 1 | 0 | 4 | 5 | 2 | 0 | 2 | 14 | success |
| 399 | YQQ | 0 | 6+3 | 23 | PS(1) | SG | RU | 2 | 0 | 5 | 3 | 5 | 0 | 3 | 2 | 18 | success |
| 400 | ZZY | 0 | 6+4 | 19 | 0 | SG | UB | 1 | 0 | 5 | 0 | 0 | 0 | 0 | 2 | 7 | success |
| 401 | ZHL | CS(2) | 6 | 35 | PS(3) | MC | UB | 0 | 4 | 5 | 4 | 5 | 2 | 0 | 0 | 20 | success |
| 402 | ZLL | 0 | 5+6 | 31 | 0 | SG | UB | 0 | 0 | 0 | 5 | 0 | 0 | 0 | 0 | 5 | success |
| ****PA*** parity. ***VD*** only vaginal deliveries and spontaneous delivery of placenta. ***CS*** ≥1 caesarean section. ***GA*** gestational age. ***MA*** maternal age. ***PT*** previous termination of pregnancy. ***PM*** previous medical abortions. ***PS*** ≥1 previous surgical abortion. ***MS*** marital status. ***SG*** single. ***MC*** married or cohabiting. ***TR*** type of residence. ***UB*** urban areas. ***RU*** rural areas. ***LU*** differences between gestational age calculated using the last menstrual period and gestational age calculated via ultrasound. ***wks*** weeks. ***yrs*** years. ***pts*** points. | | | | | | | | | | | | | | | | | |
