# Supplementary material for: Development and validation of a risk assessment model for predicting the failure of early medical abortions: A clinical prediction model study based on a systematic review and meta-analysis
Source: PLoS One. 2024 Dec 20;19(12):e0315025. doi: 10.1371/journal.pone.0315025 (PMC11661585; doi:10.1371/journal.pone.0315025)
Supplement: S1 Fig — PRISMA, preferred reporting items for systematic reviews and meta-analyses. (PPTX) [file pone.0315025.s009.pptx]

## Slide 1
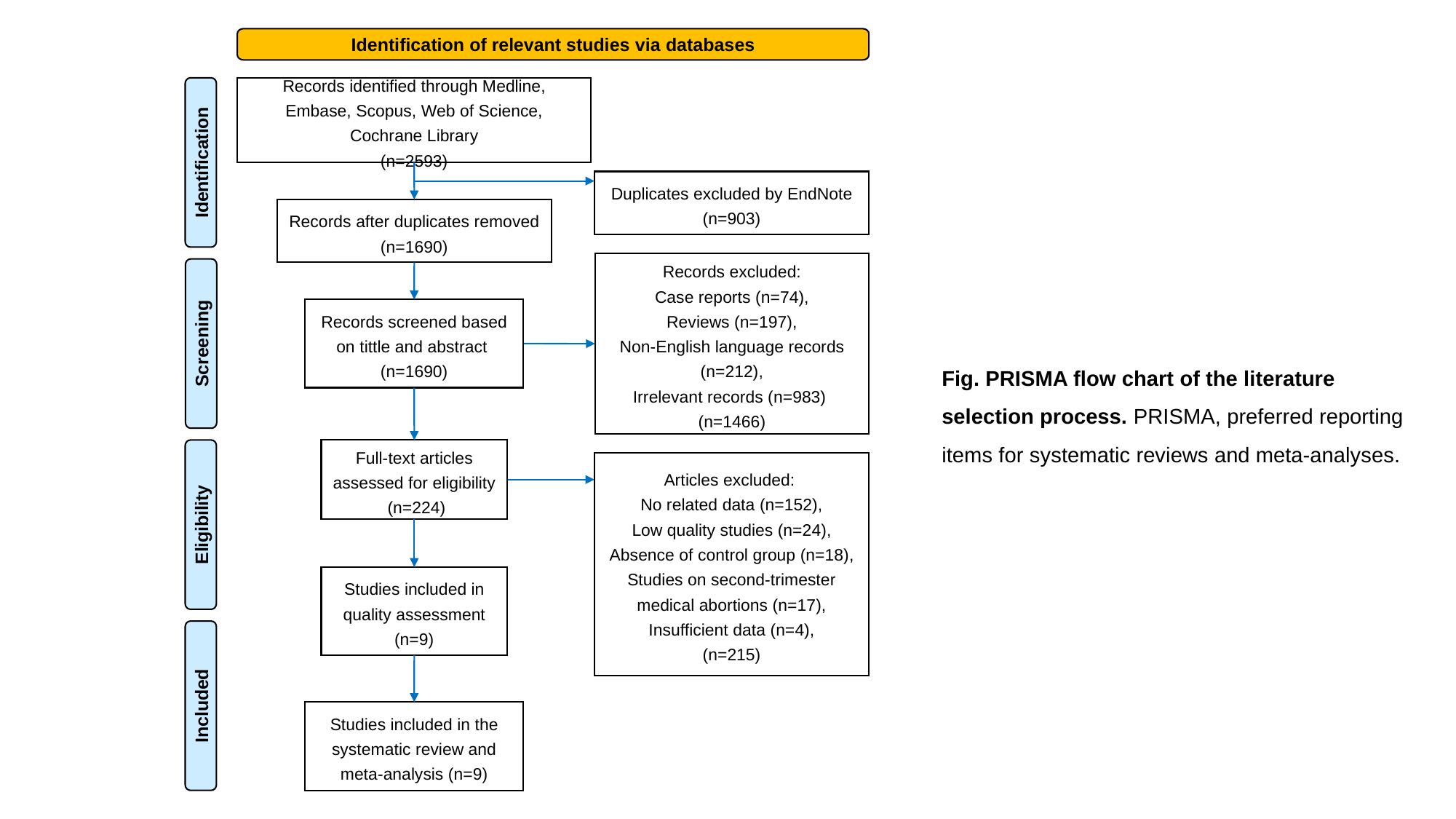

Identification of relevant studies via databases
Identification
Records identified through Medline, Embase, Scopus, Web of Science, Cochrane Library
(n=2593)
Duplicates excluded by EndNote
(n=903)
Records after duplicates removed (n=1690)
Records excluded:
Case reports (n=74),
Reviews (n=197),
Non-English language records (n=212),
Irrelevant records (n=983)
(n=1466)
Screening
Records screened based on tittle and abstract
(n=1690)
Eligibility
Full-text articles assessed for eligibility
 (n=224)
Articles excluded:
No related data (n=152),
Low quality studies (n=24),
Absence of control group (n=18),
Studies on second-trimester medical abortions (n=17),
Insufficient data (n=4),
(n=215)
Studies included in quality assessment
(n=9)
Included
Studies included in the systematic review and meta-analysis (n=9)
Fig. PRISMA flow chart of the literature selection process. PRISMA, preferred reporting items for systematic reviews and meta-analyses.
